# Reactions of B_2_(*o*‐tolyl)_4_ with Boranes: Assembly of the Pentaborane(9), HB[B(*o*‐tolyl)(μ‐H)]_4_

**DOI:** 10.1002/anie.202101054

**Published:** 2021-03-04

**Authors:** Karlee L. Bamford, Zheng‐Wang Qu, Douglas W. Stephan

**Affiliations:** ^1^ Department of Chemistry University of Toronto 80 St. George St. Toronto Ontario M5S3H6 Canada; ^2^ Mulliken Center for Theoretical Chemistry Institut für Physikalische und Theoretische Chemie Rheinische Friedrich-Wilhelms-Universität Bonn Beringstrasse 4 53115 Bonn Germany

**Keywords:** boron, cluster, diborane(4), metathesis, pentaborane(9)

## Abstract

Reactions of the diborane(4) B_2_(*o*‐tolyl)_4_ and monohydridoboranes are shown to give B(*o*‐tolyl)_3_ and (o‐tolyl)BR_2_ (R_2_=(C_8_H_14_) **3**, cat **4**, pin **5**, (C_6_F_5_)_2_ **6**) as the major products. The corresponding reaction with BH_3_‐sources gives complex mixtures, resulting from hydride/aryl exchange, dimerization and borane elimination. This led to the isolation of the first tetra‐substituted pentaborane(9) HB[B(*o*‐tolyl)(μ‐H)]_4_ **8**. The reaction pathways are probed experimentally and by computations.

## Graphical abstract

The diborane(4) B_2_(o‐tolyl)_4_ reacts with species with B−H bonds giving a complex mixture arising from hydride/aryl exchange, dimerization and borane elimination. Use of BH_3_ sources led to the isolation of the first tetra‐substituted pentaborane(9) HB[B(*o*‐tolyl)(μ‐H)]_4_.

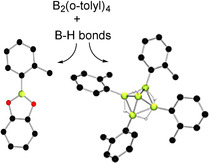

The chemistry of boron reagents continues to be of widespread interest, affording applications in complex organic syntheses,^[1]^ optoelectronics,^[2]^ materials,^[3]^ and boron cluster chemistry.^[4]^ Our interest in boron compounds, stems from their utility in “frustrated Lewis pair” (FLP) chemistry.^[5]^ While the Lewis acidity of boranes can be directly exploited in intermolecular FLPs, boron hydrides can also be employed as synthons, including in the synthesis of the now classic intramolecular FLP Mes_2_PCH_2_CH_2_B(C_6_F_5_)_2_ from the Erker group.^[6]^ In our own work, 9‐BBN served as a precursor to a N‐heterocyclic carbene stabilized borenium cation.^[7]^ In a similar vein, Crudden and co‐workers^[8]^ expanded such borenium cations to include those employing mesoionic carbenes. Among our more recent efforts to increase the diversity of main group Lewis acids,^[9]^ we have explored other electron‐deficient boron reagents in small‐molecule activation. For example, we used the borinium cation [Mes_2_B][B(C_6_F_5_)_4_], originally described by Shoji and co‐workers,^[10]^ in reactions with H_2_, hydridoborane and silane, leading to the first diboranium cation [B_2_(μ‐H)_2_(μ‐Mes)Mes_3_][B(C_6_F_5_)_4_].^[11]^ This species was also derived from Mes_2_BH and Brønsted acid. Interestingly, the corresponding protonation of (MesBH_2_)_2_ yielded the triboron cation [H_2_B(μ‐H)(μ‐Mes)B(μ‐Mes)(μ‐H)BH_2_]^+^.

Targeting new avenues to unique boron reagents, our interest focuses on the potential of diboranes(4). Though alkoxydiboranes(4) are exploited extensively in the construction of C−B bonds,^[12]^ aryldiboranes(4) have drawn much less attention. While Berndt and co‐workers reported the first aryldiborane(4) in 1988,^[13]^ earlier reduction chemistry on Mes_2_BF failed to generate B_2_Mes_4_,^[14]^ perhaps as a consequence of steric crowding. Nonetheless, in 1992 Power and co‐workers^[15]^ isolated the diboranes(4), B_2_(R)(Mes)_3_ (R=OMe, Ph, CH_2_SiMe_3_). Such tetraaryl‐substituted species (Figure 1 a) remained largely unexplored, until 2017 when Yamashita and co‐workers developed a one‐pot synthesis of the tetraaryldiborane(4), B_2_(*o*‐tolyl)_4_ and demonstrated its ability to activate H_2_.^[16]^ Later that same year, Yamaguchi and Piers^[17]^ described the ability of the dithieno‐diborin to similarly react with H_2_. In 2018, Erker and co‐woorkers^[18]^ reported the synthesis of the dissymmetric tetrasubstituted diborane(4) Ph(C_6_HR(C_6_F_5_)(SiMe_3_))BB(C_6_F_5_)_2_, while the Yamashita group reported the reactions of B_2_(*o*‐tolyl)_4_ with CO, nitriles, azobenzene, and pyridazine.^[19]^ Most recently, Yamashita and co‐workers have also reported the reduction of B_2_(*o*‐tolyl)_4_ affording a dianion which behaves as a diarylboryl anion equivalent.^[20]^ In related work on boron nucleophiles and diboranes(4), Yamashita's group also reported a doubly hydride‐bridged tetraborane(6) species.^[21]^

**Figure 1:**
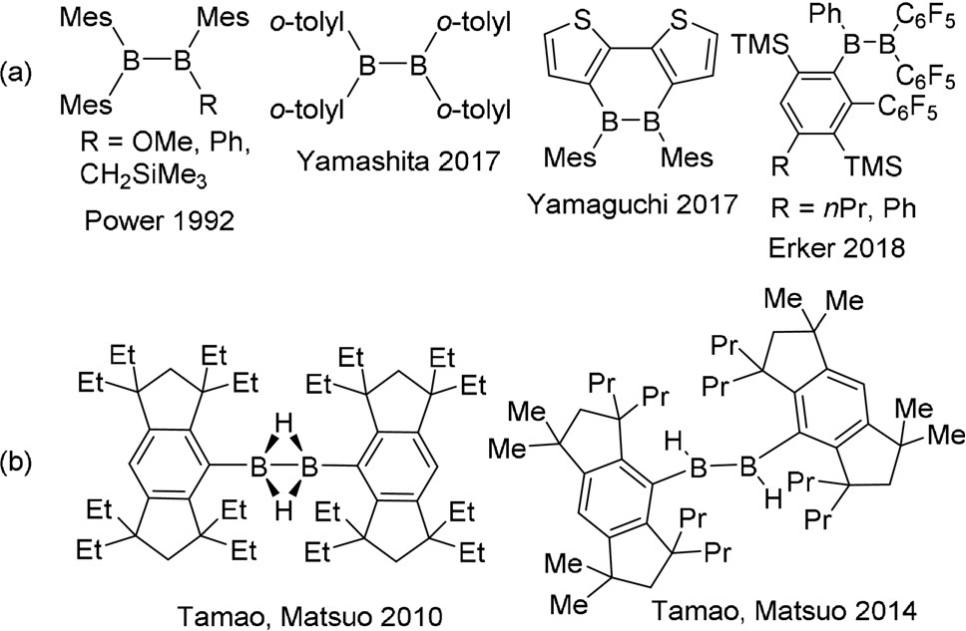
a) Known tetraaryl‐substituted diboranes(4), and b) hydrido‐substituted aryldiboranes(4).

An even more elusive subset of aryldiboranes(4) are hydrido‐substituted derivatives. Tamao and Matsuo used extreme steric demands^[22]^ to prepare the butterfly and twisted geometries of dihydridodiboranes(4) (Figure 1 b). The paucity of sterically unencumbered hydridodiboranes,^[23]^ suggests species of formulae B_2_HAr_3_ or B_2_H_2_Ar_2_ are reactive. Herein we specifically target the generation of hydridodiboranes via reactions of B_2_(*o*‐tolyl)_4_ (**1**) with secondary boranes and BH_3_‐sources. These reactions are shown to proceed via aryl/hydride exchange while subsequent reactions of the generated hydridodiboranes(4) prompt boron cluster formation. In the case of BH_3_⋅SMe_2_, the reaction with **1** gives an unprecedented tetraaryl‐pentaborane(9). The reaction pathways are probed both experimentally and computationally.

The combination of **1** and one of the monohydridoboranes, (HB(C_8_H_14_), HBcat, HBpin, or HB(C_6_F_5_)_2_), in a 1:1 ratio in benzene afforded two major products after 12 h as evidenced by NMR spectroscopy.^[24]^ An ^11^B NMR signal at 72.6 ppm, common to all reactions, was unambiguously confirmed to arise from B(*o*‐tolyl)_3_ **2** via independent synthesis and crystallographic characterization (Figure 2 a). The second products were identified as (*o*‐tolyl)B(C_8_H_14_) (**3**),^[25]^ (*o*‐tolyl)Bcat (**4**),^[26]^ (*o*‐tolyl)Bpin (**5**),^[27]^ and (*o*‐tolyl)B(C_6_F_5_)_2_ (**6**),^[28]^ respectively, based on known spectroscopic data. In the case of **4**, this was also confirmed by X‐ray crystallography (Figure 2 b).

**Figure 2:**
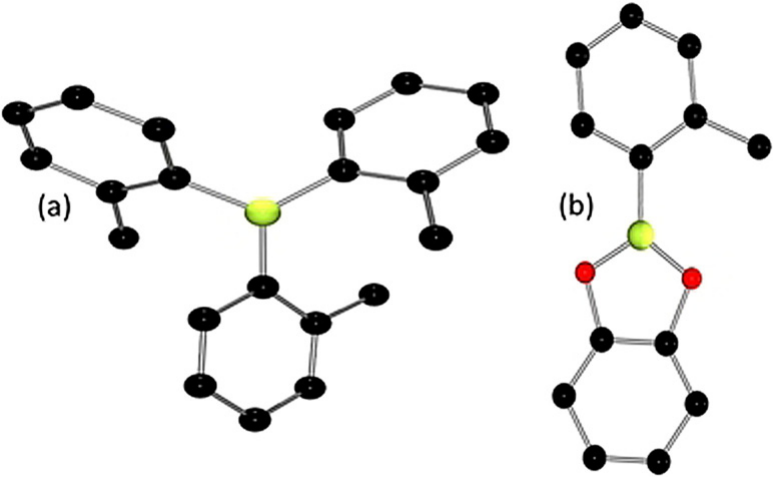
POV‐ray depiction of a) **2**, b) **4**. B: yellow‐green; C: black; O: red. All hydrogen atoms have been omitted for clarity.

In a similar fashion, reactions of **1** with two equivalents of either HBcat or HBpin gave two major products. The product common to both reactions is [H_2_B(*o*‐tolyl)]_2_ (**7**) which gives a ^11^B signal at 19 ppm (vide infra). In addition, **4** or **5** are observed as respective products (Scheme 1).

**Scheme 1:**
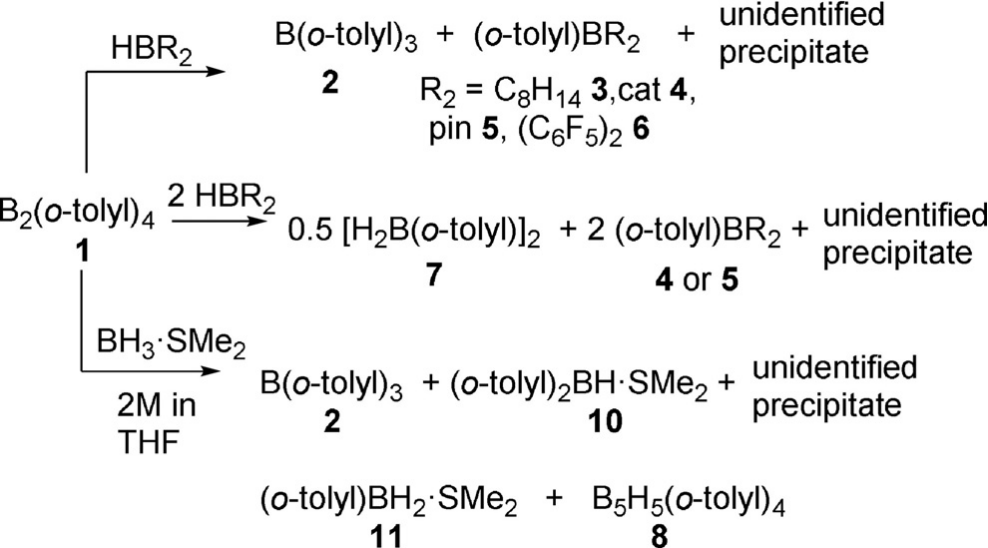
Reactions of **1** with hydridoboranes.

The formation of **3**–**6** demonstrates substituent/hydride redistribution upon combination of **1** with a monohydridoborane. However, as the corresponding diborane(4) product B_2_H(*o*‐tolyl)_3_ is not observed, further reactivity should account for the formation of **2** and **7**.

The corresponding reaction of **1** with one equivalent of BH_3_⋅SMe_2_ (2 M in THF) in toluene was monitored by NMR spectroscopy. After 16 h, **1** was consumed and major ^11^B signals at 72.4, 3.0, −0.3, −4.6, −8.3 and −46.8 ppm were observed. While the first of these resonances arises from **2**, workup afforded the isolation of a product **8** in 24 % relative yield,^[29]^ which accounts for the ^11^B NMR signals at −4.6 and −46.8 (d, ^1^ *J*_BH_=167 Hz) ppm.

A crystallographic study of **8** revealed it is a square‐pyramidal 2,3,4,5‐substituted pentaborane(9), B_5_H_5_(*o*‐tolyl)_4_ (Figure 3). The basal boron atoms have terminal *o*‐tolyl substituents with bridging hydrides, while the apical boron bears a terminal hydride. The four equivalent B–B distances in the basal plane are each 1.834(2) Å, while those to the apical boron are 1.691(3) Å, resulting in a displacement of the apical boron from the basal plane of 1.086 Å. This structure and the strongly shielded ^11^B chemical shift of the apical boron are consistent with the three‐dimensional aromaticity^[30]^ of *nido*‐pentaboranes(9), presumably accounting for the high stability of **8**. Indeed, compound **8** shows no evidence of reaction after prolonged heating at 110 °C, in toluene solution (Figures S30, S31). These observations are consistent with the known stability of the parent pentaborane(9), B_5_H_9_.^[31]^ In a related sense, compound **8** showed no reaction with D_2_ (1 atm) even after heating to 110 °C for 24 hours (Figures S32, S33). This behavior is parallel to that of B_5_H_9_ under base‐free thermolysis.[^31^, ^32^]

**Figure 3:**
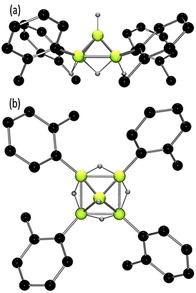
POV‐ray depiction of **8** as viewed from a) side‐on, and b) top‐down. B: yellow‐green, C: dark grey, H: light grey. All hydrogen atoms except those bound to boron centers have been omitted for clarity.

Compound **8** is, to our knowledge, a unique example of a tetrasubstituted pentaborane(9)^[33]^ and the first example in which arylation exists on the basal boron atoms of a pentaborane(9).^[34]^ Perhaps more importantly, we note that prior pentaborane(9) derivatives have been exclusively derived from functionalization of B_5_H_9_ or higher clusters,^[35]^ whereas here we assemble the B5 cluster from substituted borane and diborane species (i.e. B1 and B2 synthons).

Seeking to identify the remaining products in the reaction mixture of **1** and BH_3_⋅SMe_2_, we speculated that [H_2_B(*o*‐tolyl)]_2_ (**7**) and [HB(*o*‐tolyl)_2_]_2_ (**9**) were among them. Efforts to generate these species selectively by redistribution reactions^[36]^ of **2** and BH_3_⋅L (L=SMe_2_ or THF) failed. However, we noted that in describing the formation of **9**, HB(*o*‐tolyl)_2_⋅C_6_D_6,_ and HB(*o*‐tolyl)_2_, Yamashita and co‐workers^[16]^ had ascribed them to the ^11^B signals at 28.5, 18.6 and 72.4 ppm, respectively, in the reaction of **1** and H_2._ Noting that our data unambiguously affirmed the downfield resonance arises from **2**, we re‐examined this reaction in both hexane and C_6_D_6_, finding no spectroscopic difference.^[37]^ Given the propensity of diaryl(hydrido)boranes to dimerize,^[38]^ we suggest **7** and **9** are indeed formed from reaction of **1** and H_2_ (4 atm) and this accounts for the ^11^B signals at 18.6 and 28.5 ppm, respectively (Figures S16, S17). This view was further supported by our DFT‐computed^[39]^ ^11^B chemical shifts (see Supporting Information) for **2**, **4**, **7**, **8**, and **9** (*δ*_calc_=73.0; 37.0; 21.3; −5.6, −44.3; 28.7 ppm) that agree well with experimental values. These revised assignments indicate that neither **7** nor **9** are present in the original reaction mixture of **1**/BH_3_⋅SMe_2_. However, addition of excess SMe_2_ to the **1**/H_2_ reaction mixture showed loss of the ^11^B signals at 18.6 and 28.5 ppm and the appearance of signals at −0.3 and −8.3 ppm analogous to those seen in the reaction mixture of **1** and BH_3_⋅SMe_2_. Thus, we attribute these respective signals to (*o*‐tolyl)_2_BH⋅SMe_2_ (**10**) and (*o*‐tolyl)BH_2_⋅SMe_2_ (**11**), a view consistent with our DFT‐computed ^11^B chemical shifts (*δ*_calc_=−1.5, −5.6 ppm).

Performing the reaction of **1** with neat BH_3_⋅SMe_2_ in THF afforded no trace of **2**, rather **8** and HB(*o*‐tolyl)_2_⋅THF are formed.^[16]^ In contrast, repeating the reaction of **1** with neat BH_3_⋅SMe_2_, in the total absence of THF, afforded no trace of **8**. Instead, ^11^B NMR data reveal a mixture of **2** in addition to two new strong signals at 2.3 and −22.6 ppm (see Supporting Information). Interestingly, addition of THF to this mixture reduces the intensity of these peaks and affords **8** after 24 h, suggesting the unassigned signals arise from species that act as precursor(s) to **8**. Collectively, these data suggest that intermediate borane/SMe_2_ adducts are kinetically reactive in the presence of THF, prompting *o*‐tolyl/hydride exchange.

These reactions are unexpectedly complex given the simplicity of the reagents involved. Nonetheless, the ability of sterically unhindered aryl(hydrido)boranes^[40]^ and diboranes(4) to scramble substituents or aggregate via hydride bridges, results in complex mixtures. In addition, the presence of THF or SMe_2_ also induces equilibria for Lewis adduct formations with less encumbered boron centers. Despite these complexities, dispersion‐corrected DFT calculations were performed at the PW6B95‐D3 + COSMO‐RS// TPSS‐D3 + COSMO level (see Supporting Information)^[41]^ to garner some insight into the reactions of **1** with hydridoboranes. In the case of **1** and HBcat in toluene (Scheme 2), initial aryl/hydride exchange is 1.9 kcal mol^−1^ endergonic over a moderate free energy barrier of 20.0 kcal mol^−1^ (via transition structure **TSA**) affording the product **4** and the transient hydridodiborane(4) H(*o*‐tolyl)BB(*o*‐tolyl)_2_ (**A**). Dimerization of **A** giving (**A**)_2_ is −16.6 kcal mol^−1^ exergonic over a barrier of only 5.7 kcal mol^−1^ (via **TSAd**). This dimer needs only 7.6 kcal mol^−1^ to eliminate the experimentally observed species **2** and the computed by‐product, H_2_B_3_(*o*‐tolyl)_3_ (**Ad**). While the precise fate of **Ad** is uncertain, further reaction with borane or diborane(4) species in solution could account for the minor unidentified by‐products in the reaction mixture.

**Scheme 2:**
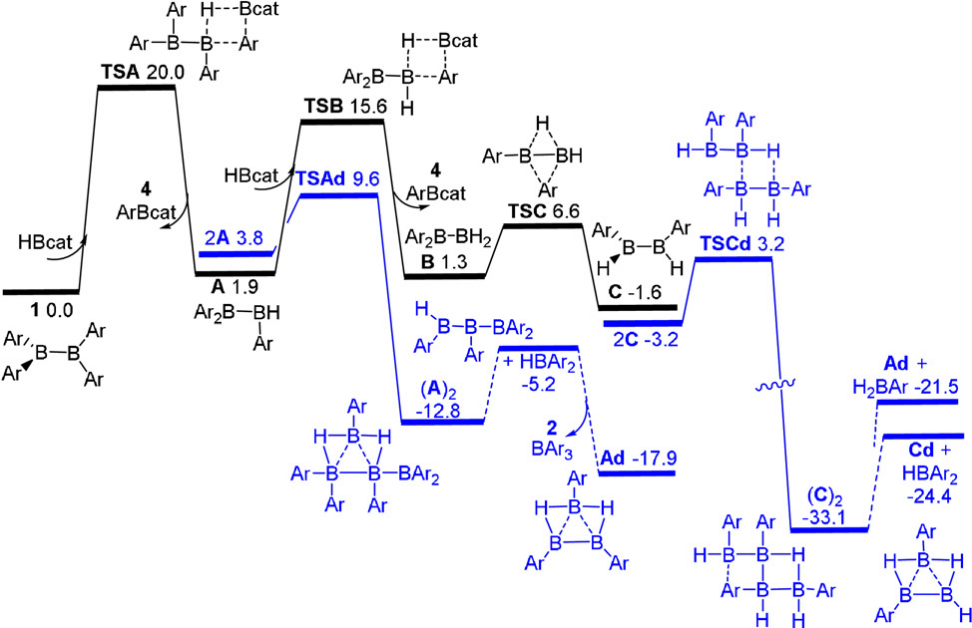
DFT‐computed free energy paths (in kcal mol^−1^, at 298 K temperature and 1 M concentration) for the reactions of **1** (Ar=*o*‐tolyl) in toluene with HBcat.

Given that reactions of **1** and hydridoboranes are computed to provide access to triboron species, it is tempting to suggest such species react with hydridodiboranes(4) to give the observed pentaborane(9) species where the degree of substitution is under thermodynamic control. Alternatively, the established nucleophilicity of sp^2^–sp^3^ diboranes^[33]^ suggests THF or SMe_2_ enhances disproportionation of hydridodiboranes(4), prompting delivery of “BH” to (**C**)_2_ affording **8**. This latter view is consistent with reports by Kodama and Perry that the sp^3^–sp^3^ diborane B_2_H_4_⋅(PMe_3_)_2_ effects expansion of boron hydride clusters by nominal diborane cleavage into BH_3_⋅(PMe_3_) and “BH⋅(PMe_3_)”.^[42]^

Analogous computations for the reaction of **1** and BH_3_⋅SMe_2_ showed an even more complex array of possibilities (see Supporting Information), such as aryl/hydride exchange reactions, dimerization of hydrido‐boron species and subsequent elimination of boranes. Nonetheless it is interesting to note that our DFT calculations infer triboron intermediates may react with diboranes, affording further thermodynamically favored aggregates such as the observed pentaborane(9) (see Supporting Information). Certainly, we can infer that the availability of additional hydrides in the reactions of BH_3_ sources favors the generation of reactive intermediates that are central to the formation of **8**.

In summary, we have shown that transient hydridodiboranes generated via reactions of the diborane(4) **1** with secondary boranes are highly reactive, providing a complex mixture of products including the known species **2**–**7**, in addition to higher boron‐aggregates. In the corresponding reaction of **1** and BH_3_‐sources, the borane **2**, the hydridoboranes **10** and **11** and the pentaborane **8** were identified among the products. These reactions demonstrated that hydride/aryl exchange, dimerization, and borane elimination reactions unlock avenues to the pentaborane(9) species **8**. This latter product represents the only known polyaryl pentaborane(9) and the first to be assembled from borane and diborane(4) components.

Supporting information for this article is given via a link at the end of the document and crystallographic data is deposited in CCDC 2049552, 2049553 and 2049554.

## Conflict of interest

The authors declare no conflict of interest.

## Supporting information

As a service to our authors and readers, this journal provides supporting information supplied by the authors. Such materials are peer reviewed and may be re‐organized for online delivery, but are not copy‐edited or typeset. Technical support issues arising from supporting information (other than missing files) should be addressed to the authors.

Supplementary

Supplementary
