# Supplementary material for: Reactions of B2(o‐tolyl)4 with Boranes: Assembly of the Pentaborane(9), HB[B(o‐tolyl)(μ‐H)]4
Source: Angew Chem Int Ed Engl. 2021 Mar 4;60(15):8532–6. doi: 10.1002/anie.202101054 (PMC8048642; doi:10.1002/anie.202101054)

checkCIF/PLATON report

Structure factors have been supplied for datablock(s) ortho_a

THIS REPORT IS FOR GUIDANCE ONLY. IF USED AS PART OF A REVIEW PROCEDURE FOR PUBLICATION, IT SHOULD NOT REPLACE THE EXPERTISE OF AN EXPERIENCED CRYSTALLOGRAPHIC REFEREE.

No syntax errors found. CIF dictionary Interpreting this report

Datablock: ortho_a

```
Bond precision:  C-C = 0.0045 A          Wavelength=0.71073

Cell:             a=4.6682(7)     b=12.4280(18)      c=18.112(2)
                  alpha=90        beta=90            gamma=90
Temperature:      150 K

                Calculated                Reported
Volume          1050.8(2)                 1050.8(3)
Space group     P 21 21 21                P 21 21 21
Hall group      P 2ac 2ab                 P 2ac 2ab
Moiety formula  C13 H11 B O2              ?
Sum formula     C13 H11 B O2              C13 H11 B O2
Mr              210.03                    210.03
Dx,g cm-3       1.328                     1.328
Z               4                         4
Mu (mm-1)       0.087                     0.087
F000            440.0                     440.0
F000'           440.21
h,k,lmax        5,15,22                   5,15,22
Nref            2132[ 1279]               2128
Tmin,Tmax       0.997,0.998               0.593,0.745
Tmin'           0.974

Correction method= # Reported T Limits: Tmin=0.593 Tmax=0.745
AbsCorr = MULTI-SCAN

Data completeness= 1.66/1.00      Theta(max)= 26.313

R(reflections)= 0.0464( 1602)     wR2(reflections)= 0.0993( 2128)

S = 0.989                  Npar= 146
```

The following ALERTS were generated. Each ALERT has the format
test-name_ALERT_alert-type_alert-level.
Click on the hyperlinks for more details of the test.

Alert level C

```
PLAT340_ALERT_3_C Low Bond Precision on  C-C Bonds ...............    0.00446 Ang.
PLAT906_ALERT_3_C Large K Value in the Analysis of Variance ......      2.495 Check
```

Alert level G

```
PLAT395_ALERT_2_G Deviating  X-O-Y    Angle From 120 for O01            105.2 Degree
PLAT395_ALERT_2_G Deviating  X-O-Y    Angle From 120 for O02            106.1 Degree
PLAT720_ALERT_4_G Number of Unusual/Non-Standard Labels ..........          3 Note
PLAT912_ALERT_4_G Missing # of FCF Reflections Above STh/L=  0.600          1 Note
PLAT933_ALERT_2_G Number of OMIT Records in Embedded .res File ...          1 Note
PLAT978_ALERT_2_G Number C-C Bonds with Positive Residual Density.          1 Info
```

```
   0 ALERT level A = Most likely a serious problem - resolve or explain
   0 ALERT level B = A potentially serious problem, consider carefully
   2 ALERT level C = Check. Ensure it is not caused by an omission or oversight
   6 ALERT level G = General information/check it is not something unexpected

   0 ALERT type 1 CIF construction/syntax error, inconsistent or missing data
   4 ALERT type 2 Indicator that the structure model may be wrong or deficient
   2 ALERT type 3 Indicator that the structure quality may be low
   2 ALERT type 4 Improvement, methodology, query or suggestion
   0 ALERT type 5 Informative message, check
```

It is advisable to attempt to resolve as many as possible of the alerts in all categories. Often the minor alerts point to easily fixed oversights, errors and omissions in your CIF or refinement strategy, so attention to these fine details can be worthwhile. In order to resolve some of the more serious problems it may be necessary to carry out additional measurements or structure refinements. However, the purpose of your study may justify the reported deviations and the more serious of these should normally be commented upon in the discussion or experimental section of a paper or in the "special_details" fields of the CIF. checkCIF was carefully designed to identify outliers and unusual parameters, but every test has its limitations and alerts that are not important in a particular case may appear. Conversely, the absence of alerts does not guarantee there are no aspects of the results needing attention. It is up to the individual to critically assess their own results and, if necessary, seek expert advice.

Publication of your CIF in IUCr journals

A basic structural check has been run on your CIF. These basic checks will be run on all CIFs submitted for publication in IUCr journals (*Acta Crystallographica*, *Journal of Applied Crystallography*, *Journal of Synchrotron Radiation*); however, if you intend to submit to *Acta Crystallographica Section C* or *E* or *IUCrData*, you should make sure that full publication checks are run on the final version of your CIF prior to submission.

Publication of your CIF in other journals

Please refer to the *Notes for Authors* of the relevant journal for any special instructions relating to CIF submission.

PLATON version of 26/06/2020; check.def file version of 17/06/2020

Datablock ortho_a - ellipsoid plot

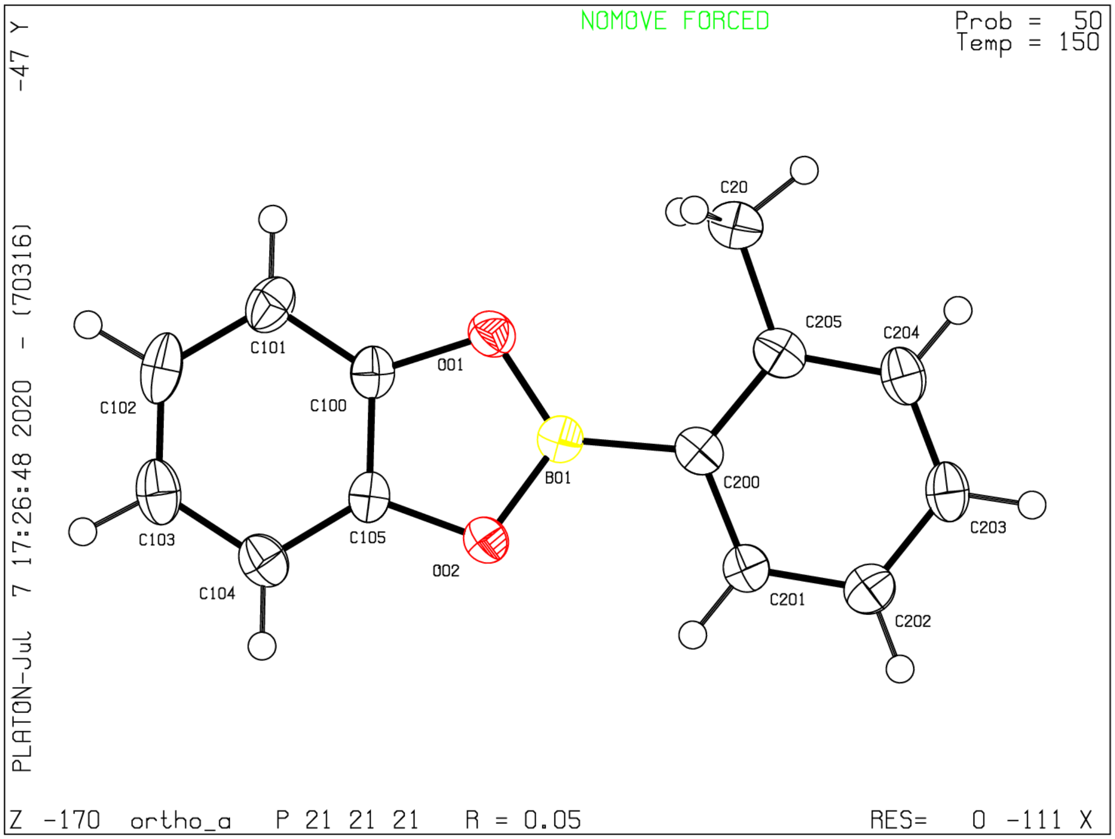

Supplement: Supplementary file 1 — Supplementary [file ANIE-60-8532-s001.zip › checkcif-o-tolylBcat.pdf]
