# Supplementary material for: Reactions of B2(o‐tolyl)4 with Boranes: Assembly of the Pentaborane(9), HB[B(o‐tolyl)(μ‐H)]4
Source: Angew Chem Int Ed Engl. 2021 Mar 4;60(15):8532–6. doi: 10.1002/anie.202101054 (PMC8048642; doi:10.1002/anie.202101054)
Supplement: Supplementary file 2 — Supplementary [file ANIE-60-8532-s002.pdf]

Angewandte Chemie
Eine Zeitschrift der Gesellschaft Deutscher Chemiker

Supporting Information

Reactions of B_2(*o*-tolyl)$_4$ with Boranes: Assembly of the Pentaborane(9), HB[B(*o*-tolyl)(μ-H)]$_4$

Karlee L. Bamford, Zheng-Wang Qu, and Douglas W. Stephan**

anie_202101054_sm_miscellaneous_information.pdf
anie_202101054_sm_cif.zip

Supporting Information
©Wiley-VCH 2019
Weinheim, Germany

Reactions of the B_2(*o*-tolyl)$_4$ with Boranes: Assembly of the Pentaborane(9), HB(B(*o*-tolyl)(μ-H))$_4$

Karlee L. Bamford[a], Zheng-Wang Qu[b]*, and Douglas W. Stephan*[a]

Abstract: Reactions of the diborane(4) B_2(*o*-tolyl)$_4$ and monohydridoboranes are shown to give B(*o*-tolyl)$_3$ and (o-tolyl)BR_2 (R_2 = (C_8H_{14}) **3**, cat **4**, pin **5**, (C_6F_5)$_2$ **6**) as the major products. The corresponding reaction with BH_3-sources complex mixtures, resulting from hydride/aryl exchange, dimerization and borane elimination. This led to the isolation of the first tetra-substituted pentaborane(9) HB(B(*o*-tolyl)(μ-H))$_4$ **8**. The reaction pathways are probed experimentally and by computations.

DOI: 10.1002/anie.2020XXXXX

Table of Contents

Experimental Procedures 3

Chemicals and Materials 3

Physical Methods 4

Test Reactions of Compound 1 4

Preparation of Compound 2 4

Generation and Isolation of (*o*-tolyl)Bcat (Compound 4) 5

Preparation of Compound 8 5

Characterization 6

Compound 2 6

Compound 4 8

Compound 8 10

Compound 1/BH_3·L Reaction Precipitate 14

Reaction Monitoring NMR Spectra 17

Compound 1 and H_2 17

Compound 1 and BH_3-Sources 20

Compound 1 and Hydridoboranes 24

Compound 8 Stability and Attempted Reaction with D_2 25

Single-Crystal X-Ray Crystallography 27

Computational Details 29

References 106

Experimental Procedures

Chemicals and Materials

All preparative procedures were performed in an inert atmosphere of dry, deoxygenated (O_2 < 0.5 ppm) nitrogen, using glovebox techniques or standard Schlenk techniques unless otherwise specified. Solvents were stored over activated 3 Å molecular sieves following drying procedures. Anhydrous toluene (Sigma Aldrich) and HPLC-grade pentane, *n*-hexane, and diethyl ether (Caledon Laboratories Ltd.) were dried using a Grubbs-type Innovative Technologies solvent purification system. Anhydrous benzene (Sigma Aldrich) was stored over 3Å molecular sieves prior to use. Tetrahydrofuran (THF) was purchased from Caledon Laboratories Ltd. and distilled from sodium-benzophenone. Deuterated solvents were purchased from Cambridge Isotope Laboratories Inc. (C_6D_6, toluene-d_8, THF-d_8), distilled from CaH_2 or sodium-benzophenone, and stored over 3Å molecular sieves prior to use. A Nanochem Weldassure purifier column was used to dry H_2 gas (grade 5.0, supplied by Linde) used in hydrogenation reactions (4 atm) of compound **1**. The attempted reaction of compound **8** with D_2 (Sigma Aldrich) at 1 atm was conducted at Dalhousie University, courtesy of Prof. Saurabh Chitnis.

Anhydrous dimethylsulfide (SMe_2), borane dimethylsulfide ($BH_3{\cdot}SMe_2$; neat or 2M in THF), borane tetrahydrofuran ($BH_3{\cdot}$THF; 1M in THF), 9-borabicyclo[3.3.1]nonane (9-BBN), and boron trifluoride diethyl etherate ($BF_3{\cdot}OEt_2$) were purchased from Sigma Aldrich and used without further purification. Catecholborane (HBcat) and pinacolborane (HBin) were purchased from Sigma Aldrich and Strem Chemicals, respectively, and were distilled prior to use. Trimethylsilyl chloride was purchased from Sigma Aldrich and distilled from CaH_2. Mesityllithium (MesLi) was prepared according to literature procedure,[1] using commercially available 2-bromomesitylene (TCI America) and *n*-butyllithium (2.5 M in *n*-hexane; Sigma Aldrich). Tris(pentafluorophenyl)borane ($B(C_6F_5)_3$; Boulder Scientific) was used as received in the reported synthesis of $HB(C_6F_5)_2$ ("Piers' borane").[2] The reported synthesis of **1** (B_2(*o*-tolyl)$_4$) was followed using commercial *o*-tolylmagnesium bromide (2M in Et_2O; Sigma Aldrich) and bis(catecholato)diboron (B_2cat_2; Strem Chemicals).[3] The same Grignard reagent was used in the preparation of **2**. Hamilton micro-syringes were used to transfer neat $BH_3{\cdot}SMe_2$ and THF solutions of $BH_3{\cdot}L$ (L = THF or SMe_2). Plastic syringes and disposable needles used to dispense solvents for NMR-scale reactions were prepared prior to use by evacuation in the antechamber of the glovebox overnight. Celite used in filtrations as well as molecular sieves were similarly dried at 300 °C under vacuum for 24–48 hours.

Physical Methods

All NMR spectra were collected at 298 K on Bruker Avance III 400, Agilent DD2 500, or Agilent DD2 600 spectrometers in 3 or 5 mm diameter NMR tubes. ^{1}H and ^{13}C{^{1}H} chemical shifts are reported relative to protio-solvent signals (C_6D_6: δ(^{1}H) = 7.16 ppm, δ(^{13}C{^{1}H}) = 128.1 ppm; toluene-d_8: δ(^{1}H) = 7.09, 7.01, 6.97, and 2.08 ppm, δ(^{13}C{^{1}H}) = 137.5, 128.9, 127.9, 125.1, and 20.4 ppm; THF-d_8: δ(^{1}H) =3.58 and 1.72 ppm, δ(^{13}C{^{1}H}) = 67.2 and 25.3 ppm), while ^{11}B and ^{19}F chemical shifts are reported relative to $(Et_2O)\cdot BF_3$ and $CFCl_3$ external standards, respectively. All chemical shifts (δ) are reported in ppm and coupling constants are given in Hz. Complete NMR assignment of compounds **2** and **8** was accomplished using HSQC and HMBC heteronuclear correlation techniques. High pressure NMR tubes were used for H_2 reactions (4 atm) conducted at the University of Toronto, while low pressure NMR tubes were used for D_2 reactions (1 atm) conducted at Dalhousie University.

Departmental facilities were used for high resolution mass spectrometry (DART: JEOL AccuTOF) and elemental analysis (Perkin Elmer 2400 Series II CHNS Analyser).

Test Reactions of Compound 1

In an inert atmosphere glovebox, $B_2(o\text{-tolyl})_4$ (**1**; 19.3 mg, 0.05 mmol) in a 1 dr vial was dissolved in 0.2 mL C_6D_6 and was either a) transferred to 1 dr vial containing a monohydridoborane reagent (0.05 or 0.1 mmol) dissolved in 0.2 mL of the same solvent, or b) added to it by an aliquot of $BH_3\cdot L$ reagent (L = THF or SMe_2; THF solutions or neat; 0.017–0.05 mmol). For monohydridoborane reactions, an additional 0.2 mL of solvent was used to quantitatively combine the reagents and transfer the reaction mixture by pipette to a 5 mm NMR tube, bringing the total reaction volume to approximately 0.6 mL. For $BH_3\cdot L$ reactions, an additional 0.4 mL of solvent was used to quantitively transfer the reaction mixture to a 5 mm NMR tube. NMR tubes were capped and sealed with Teflon tape while still inside the glovebox and then additionally sealed with Parafilm outside of the glovebox. Samples were typically monitored by ^{1}H and ^{11}B NMR spectroscopy over the course of 24 hours.

Preparation of Compound 2

A similar preparation to that used for BPh_3 was followed on a 2 mmol scale,[4] using *o*-tolylmagnesium bromide in place of phenylmagnesium bromide. After the addition of the Grignard reagent, the reaction was allowed to stir at room temperature overnight. The following day, volatiles were removed *in vacuo* and the product was extracted with excess pentane (5 x 2 mL washes). The extract was filtered, concentrated under reduced pressure, and

recrystallized at –30 °C. The product was obtained as colourless crystals and dried *in vacuo* to give a white powder (Yield: 358.1 mg, 63%).

Generation and Isolation of (*o*-tolyl)Bcat (Compound 4)

Following the procedure described above for NMR-scale test reactions involving compound **1**, the crude reaction mixture of HBcat and compound **1** was returned to the glovebox and dried *in vacuo*. The reaction residue, a white solid, was extracted with 3 x 0.3 mL of pentane and passed through a short plug of celite into a fresh 1 dr vial. The pentane extract was cooled to –30 °C for 24 hours, furnishing crystals of **4**. The mother liquor was decanted and the crystals were dried *in vacuo* for spectroscopic study.

Preparation of Compound 8

In an inert atmosphere glovebox, **1** (28.0 mg, 0.07 mmol) was weighed into a 2 dr vial and dissolved in 1.5 mL benzene or toluene. The vial was equipped with a stir bar and $BH_3{\cdot}SMe_2$ (2 M in THF; 25 μL, 0.05 mmol) was subsequently added by syringe. The resulting near-colourless solution was allowed to stir for 24 hours before an aliquot of the then yellow coloured solution was taken for multinuclear NMR spectroscopic analysis in a 5 mm NMR tube sealed with Teflon tape and Parafilm. Isolation of the product was achieved by removal of volatiles *in vacuo* from the crude reaction mixture and extraction with pentane (3 x 0.5 mL washes), leaving a white precipitate*. The extract was filtered through a short celite plug into a fresh 1 dr vial, concentrated, and stored in a freezer cooled to –30 °C for 24 hours to give colourless needles crystals suitable for X-ray diffraction studies. The mother liquor was decanted and the crystals were dried *in vacuo* to give a white powder (Yield: 7.3 mg, 24%**).

*The identity of the precipitate is presently unknown. The solid is readily soluble in benzene and toluene, but insoluble in pentane or hexane. The solid is ^{11}B and ^{13}C NMR-silent, however mass spectrometric studies (see Characterization section) suggest the presence of species containing multiple boron atoms.

As the reaction stoichiometry is not presently understood, the percentage yield was calculated assuming starting material **1 is the limiting reagent and that **1** and product **8** are in 1:1 stoichiometry.

Characterization

Compound 2

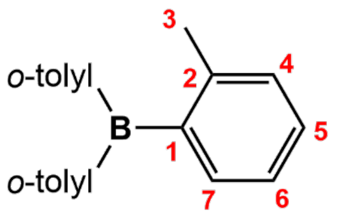

^{1}H NMR (500 MHz, C_6D_6, 298 K): δ (ppm) 7.26 (dd, $^{3}J_{HH}$ = 7.4 Hz, $^{4}J_{HH}$ = 1.6 Hz, 3H, H_7), 7.17 (td, $^{3}J_{HH}$ = 7.5 Hz, $^{4}J_{HH}$ = 1.6 Hz, 3H, H_5), 7.04 (br d, $^{3}J_{HH}$ = 7.0 Hz, 3H, H_4), 7.01 (tm, $^{3}J_{HH}$ = 7.4 Hz, 3H, H_6), 2.09 (s, 9H, H_3).
^{11}B NMR (128 MHz, C_6D_6, 298 K): δ (ppm) 72.6 (br s).
^{13}C{^{1}H} NMR (126 MHz, C_6D_6, 298 K): δ (ppm) 146.7 (br s, C_1), 142.5 (s, C_2), 135.1 (s, C_7), 130.8 (s, C_5), 130.1 (s, C_4), 125.4 (s, C_6), 23.4 (apparent d, C_3).

MS (DART(+) Ionization, 200 °C, *m/z*): 302.2 ($[M+NH_4]^+$).
HRMS (DART-TOF(+) Ionization, *m/z*): Calcd. for $C_{21}H_{25}BN$, ($[M+NH_4]^+$): 302.20746; Found: 302.20750.

Elemental analysis: calcd (%) $C_{21}H_{21}B$: C 88.75, H 7.45, N 0; Found: C 87.10*, H 7.40, N 0. *Low carbon values are frequently obtained for compounds featuring electropositive elements due to the ready formation of carbides.[5]

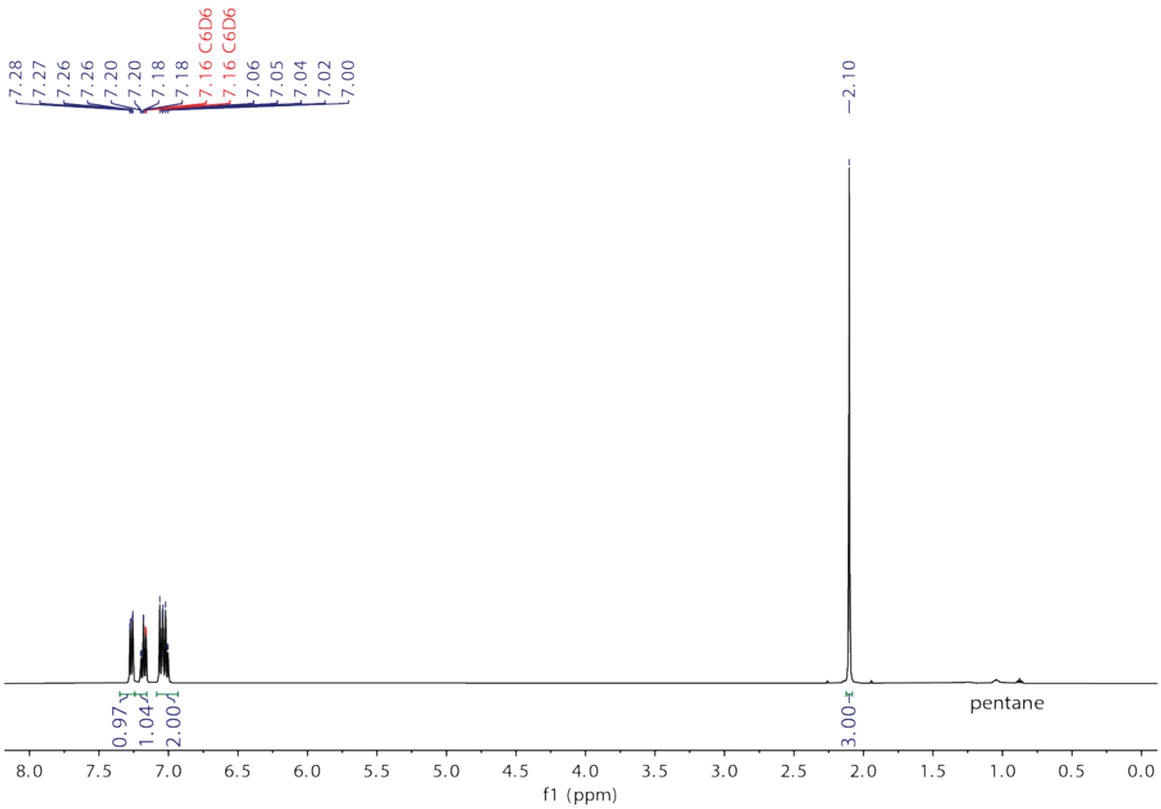

Figure S1. ^{1}H NMR spectrum (500 MHz, C_6D_6, 298 K) of **2**.

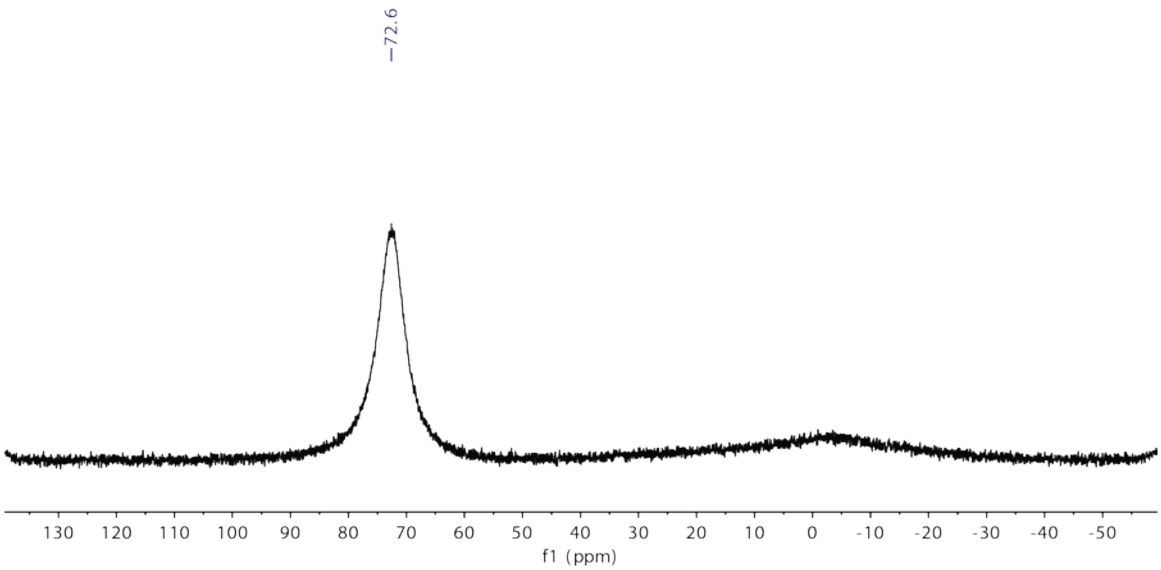

Figure S2. ^{11}B NMR spectrum (128 MHz, C_6D_6, 298 K) of **2**.

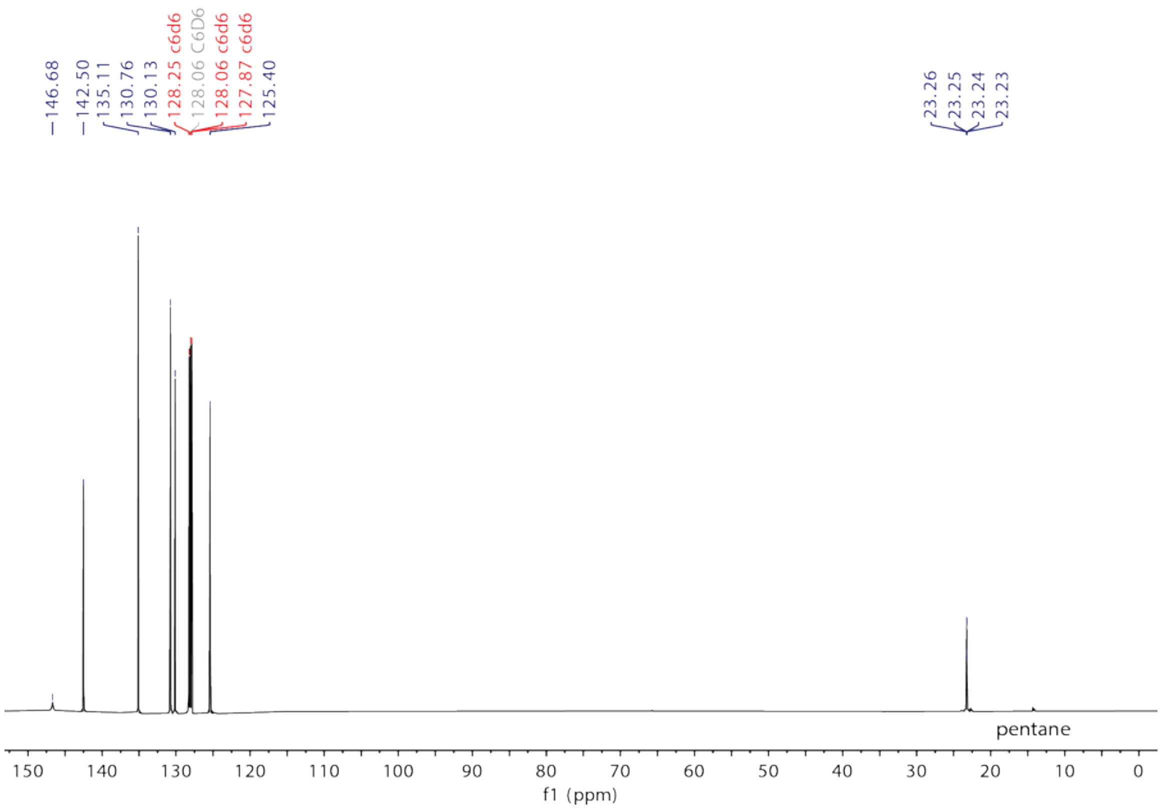

Figure S3. $^{13}C\{^1H\}$ NMR spectrum (126 MHz, C_6D_6, 298 K) of **2**.

Compound 4

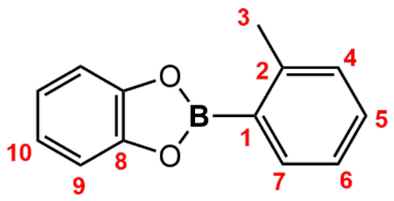

Data are in agreement with those previously reported for (*o*-tolyl)Bcat.[6]

^{1}H NMR (400 MHz, C_6D_6, 298 K): δ (ppm) 8.23 (dd, J_{HH} = 7.4, 1.6 Hz, 1H, H_7), 7.24–7.03 (m, 5H*, H_4 & H_5 & H_6 & H_{10}), 6.84–6.81 (m, 2H, H_9), 2.66 (s, 3H, H_3). *Overlaps with benzene solvent signal.

^{11}B NMR (128 MHz, C_6D_6, 298 K): δ (ppm) 32.6 (br s).

$^{13}C\{^1H\}$ NMR (100 MHz, C_6D_6, 298 K): δ (ppm) 148.9 (s, C_8), 145.8 (s, C_2), 137.1 (s, C_7), 132.4 (s, C_5), 130.6 (s, C_4), 125.6 (s, C_6), 122.9 (s, C_{10}), 112.8 (s, C_9), 22.6 (s, C_3). The signal for C_1 was not observed.

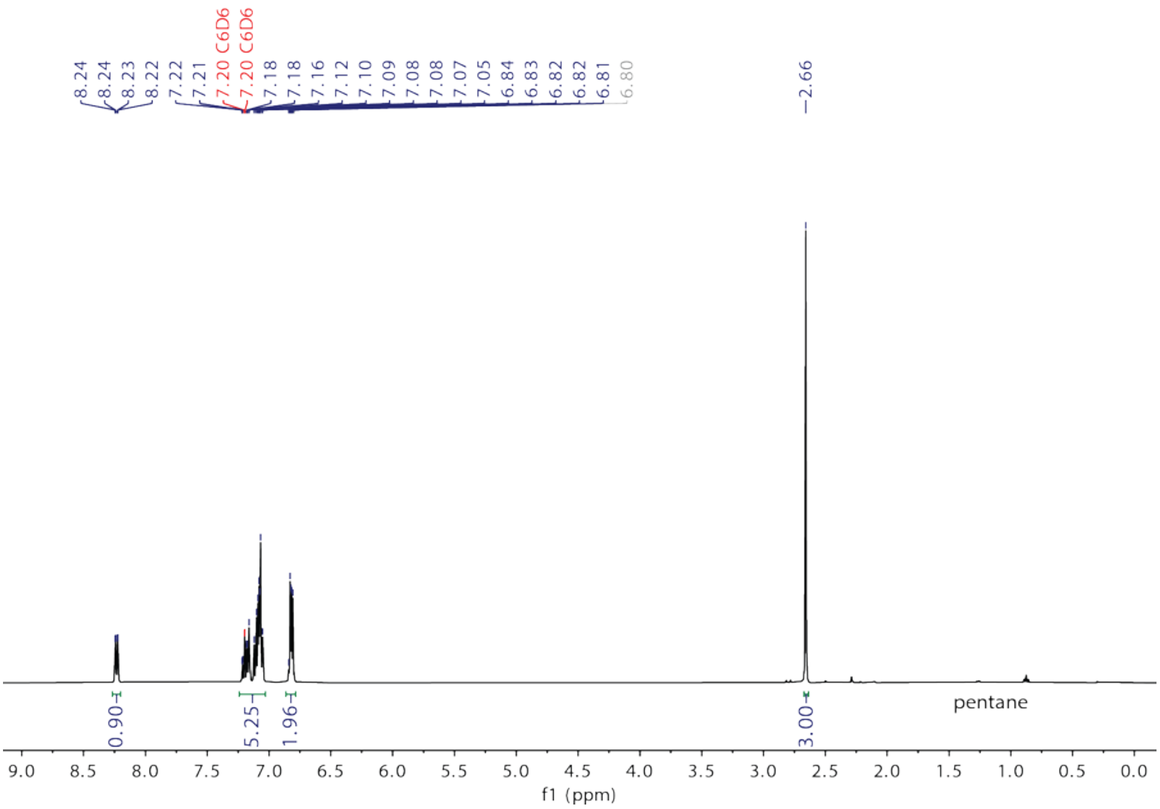

Figure S4. ^{1}H NMR spectrum (400 MHz, C_6D_6, 298 K) of **4**.

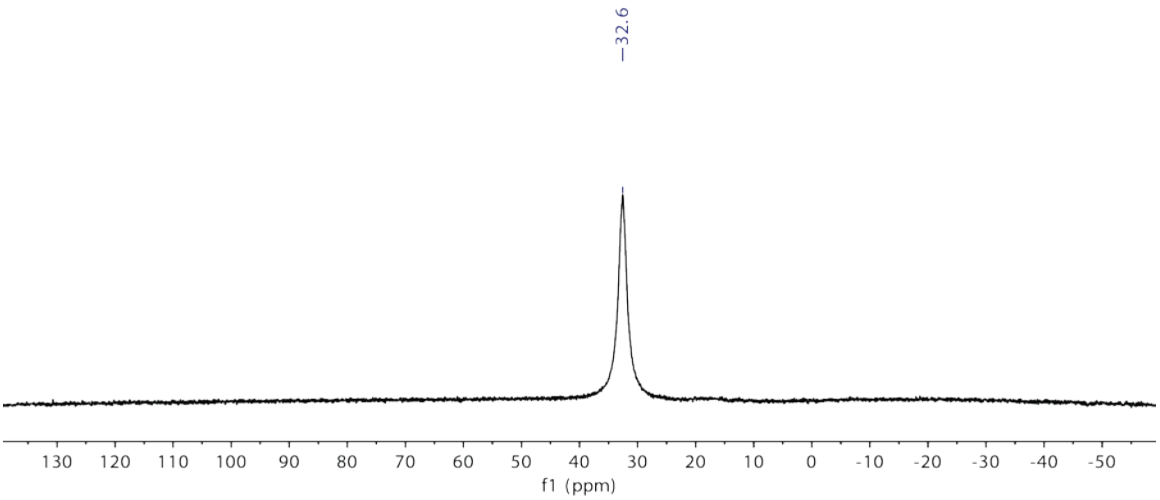

Figure S5. ^{11}B NMR spectrum (128 MHz, C_6D_6, 298 K) of **4**.

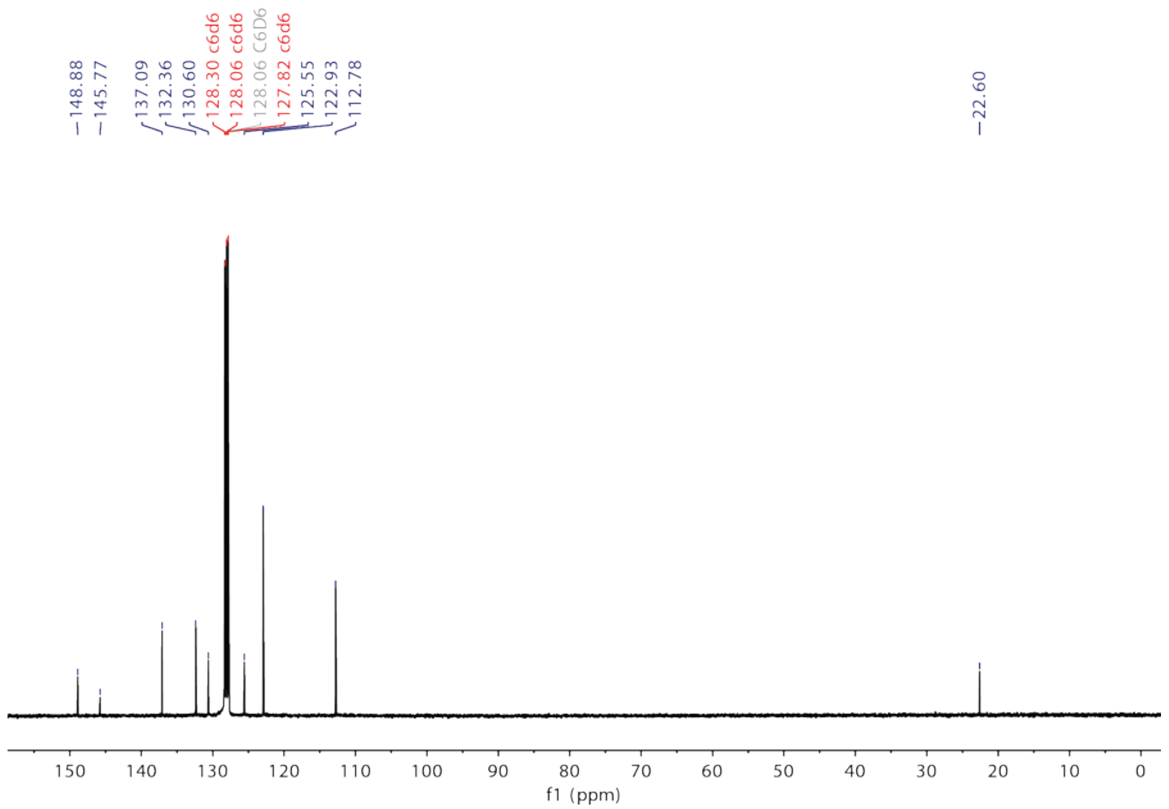

Figure S6. $^{13}C\{^1H\}$ NMR spectrum (100 MHz, C_6D_6, 298 K) of **4**.

Compound 8

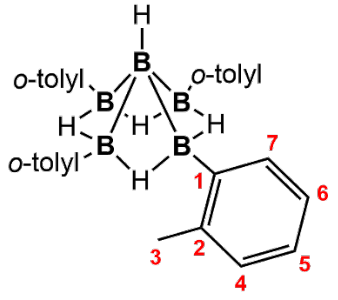

Characterization in C_6D_6 solvent:

^{1}H NMR (400 MHz, C_6D_6, 298 K): δ (ppm) 7.66 (dd, $^3J_{HH}$ = 7.4 Hz, $^2J_{HH}$ = 1.5 Hz, 4H, H_7), 7.04 (tm, $^3J_{HH}$ = 7.6 Hz, 4H, H_5), 6.98–6.90 (m, 8H, H_4 & H_6), 2.25 (s, 12H, H_3), 2.16 (br s, 1H, B–$H_{terminal}$), 1.30 (br s, 4H, B–H_{bridge}).
^{11}B NMR (128 MHz, C_6D_6, 298 K): δ (ppm) –4.8 (br s, B-basal) , –45.9 (d, $^1J_{BH}$ = 167 Hz, B-apical).
$^{13}C\{^1H\}$ NMR (126 MHz, C_6D_6, 298 K): δ (ppm) 141.9 (s, C_2), 137.7 (s, C_7), 130.4 (br, C_1), 129.9 (s, C_5), 129.5 (s, C_4), 125.9 (s, C_6), 23.1 (m*, C_3). *The signal is a second order multiplet.

Characterization in THF-d_8 solvent:

^{1}H NMR (400 MHz, THF-d_8, 298 K): δ (ppm) 7.47 (dd, $^{3}J_{HH}$ = 7.4 Hz, $^{2}J_{HH}$ = 1.5 Hz, 4H, H_7), 7.13 (td, $^{3}J_{HH}$ = 7.4 Hz, $^{2}J_{HH}$ = 1.5 Hz, 4H, H_5), 7.07 (d, $^{3}J_{HH}$ = 7.5 Hz, 4H, H_4), 7.03–6.95 (m, 4H, H_6), 2.35 (s, 12H, H_3), 1.70 (br s*, 1H, B–$H_{terminal}$), 1.38 (br s, 4H, B–H_{bridge}). *Overlaps with THF solvent signal.

^{11}B NMR (128 MHz, THF-d_8, 298 K): δ (ppm) –4.9 (br s, B-basal), –46.4 (d, $^{1}J_{BH}$ = 167 Hz, B-apical).

^{13}C{^{1}H} NMR (126 MHz, THF-d_8, 298 K): δ (ppm) 142.7 (s, C_2), 138.2 (s, C_7), 131.3 (br, C_1), 130.2 (s, C_5), 129.8 (s, C_4), 126.2 (s, C_6), 23.3 (m*, C_3). *The signal is a second order multiplet.

MS (DART(+) Ionization, 400 °C, *m/z*): 442.3 ($[M+NH_4]^+$).

HRMS (DART-TOF(+) Ionization, *m/z*): Calcd. for $C_{28}H_{37}B_5N$, ($[M+NH_4]^+$): 442.33858; Found: 442.34056.

Elemental analysis: calcd (%) $C_{28}H_{33}B_5$: C 79.39, H 7.85, N 0; Found: C 73.52*, H 6.59, N 0. *Low carbon values are frequently obtained for compounds featuring electropositive elements due to the ready formation of carbides.[5]

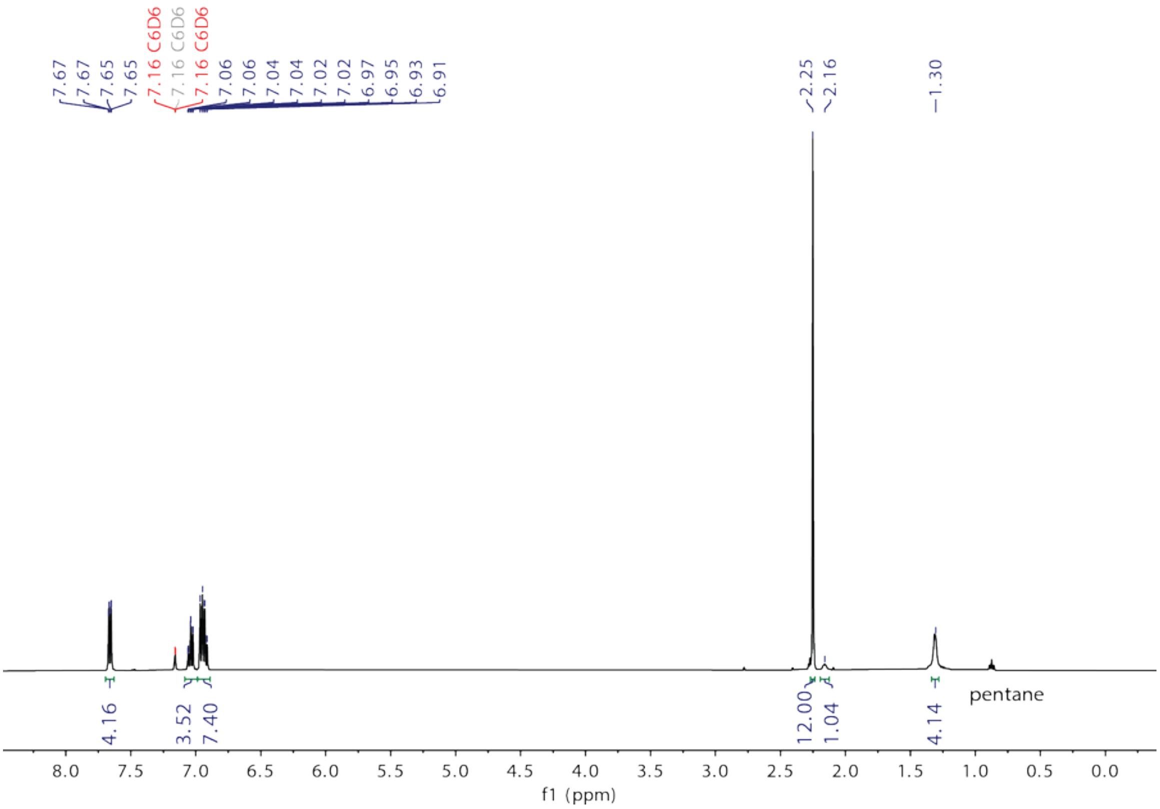

Figure S7. $^{1}H\{^{11}B\}$ NMR spectrum (500 MHz, C_6D_6, 298 K) of **8**.

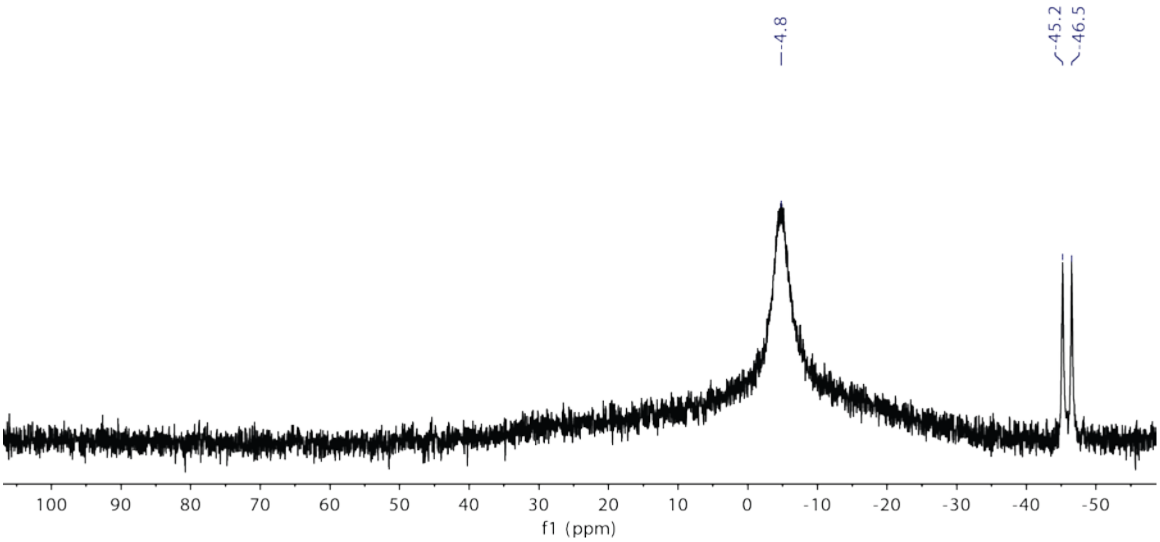

Figure S8. ^{11}B NMR spectrum (128 MHz, C_6D_6, 298 K) of **8**.

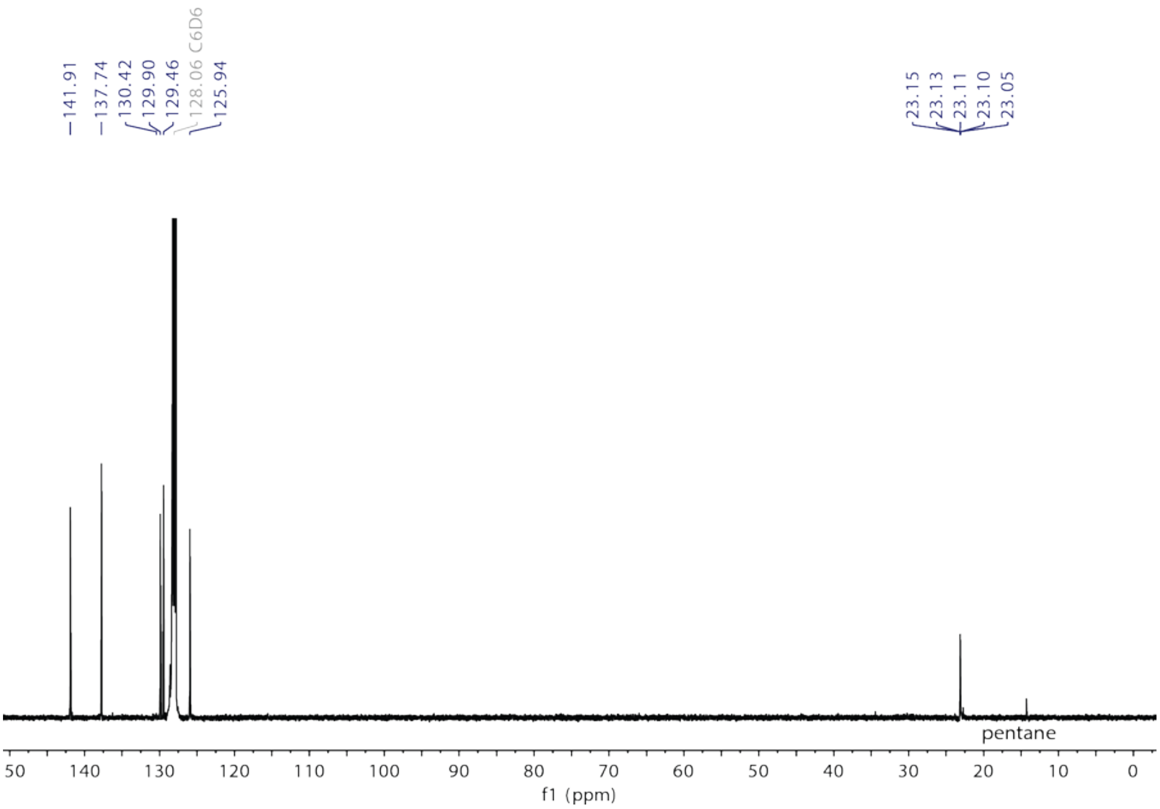

Figure S9. $^{13}C\{^{1}H\}$ NMR spectrum (126 MHz, C_6D_6, 298 K) of **8**.

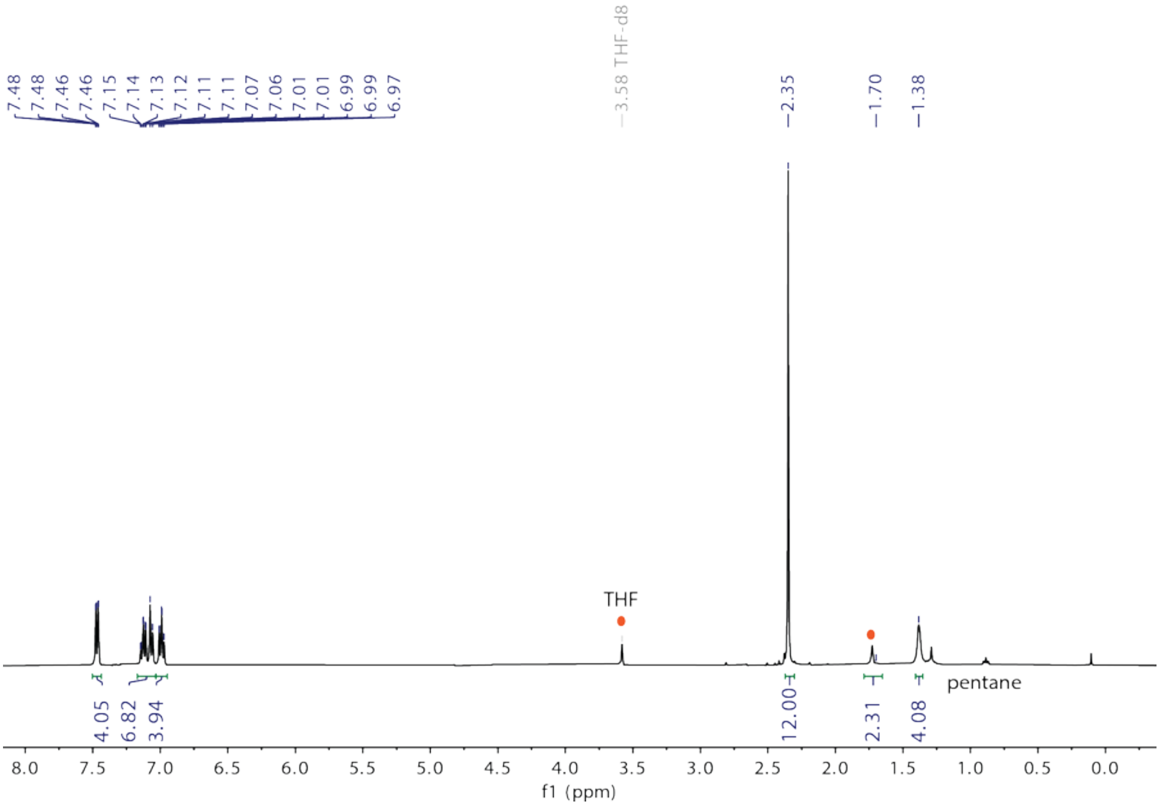

Figure S10. $^{1}H\{^{11}B\}$ NMR spectrum (500 MHz, THF-d_8, 298 K) of **8**.

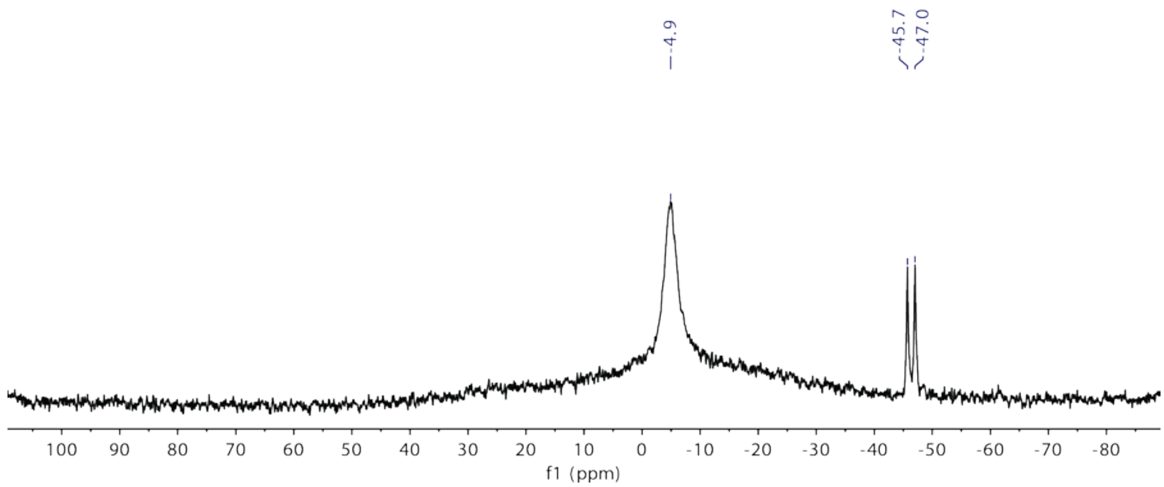

Figure S11. ^{11}B NMR spectrum (128 MHz, THF-d_8, 298 K) of **8**.

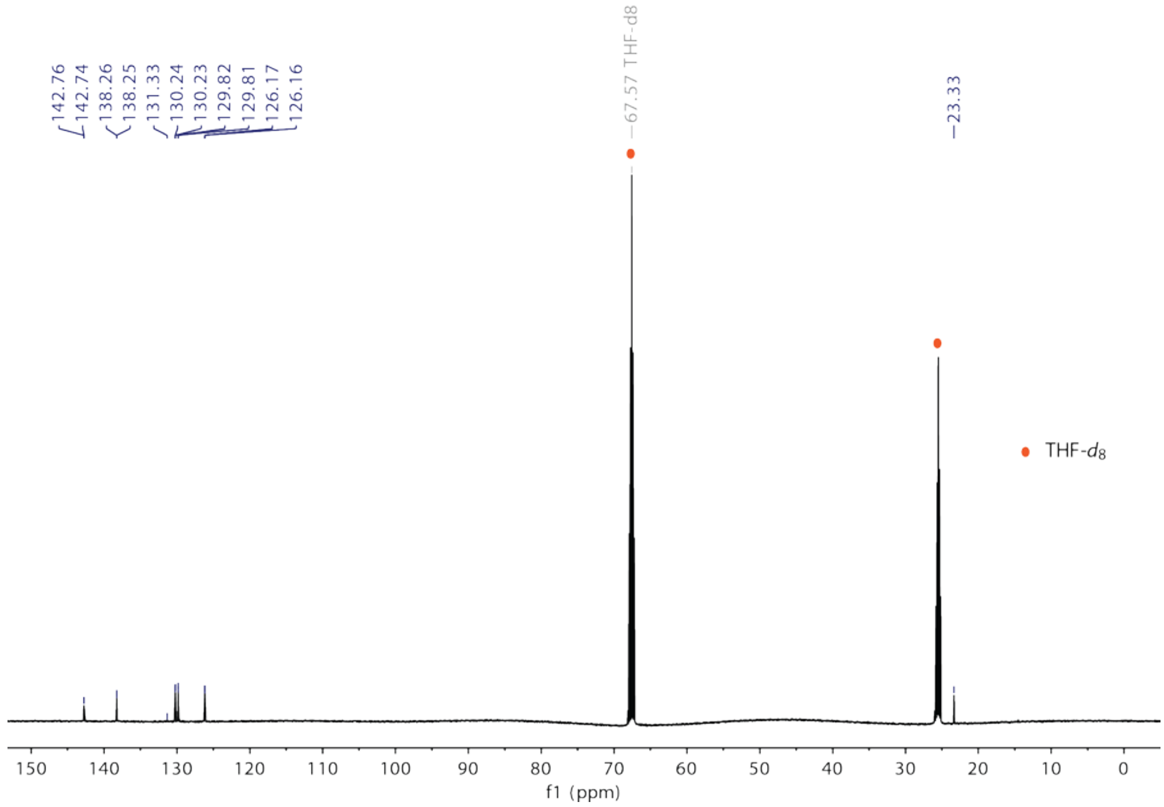

Figure S12. $^{13}C\{^1H\}$ NMR spectrum (126 MHz, THF-d_8, 298 K) of **8**.

Compound 1/BH_3·L Reaction Precipitate

Elemental analysis found: C 14.33, H 2.25, N 0.

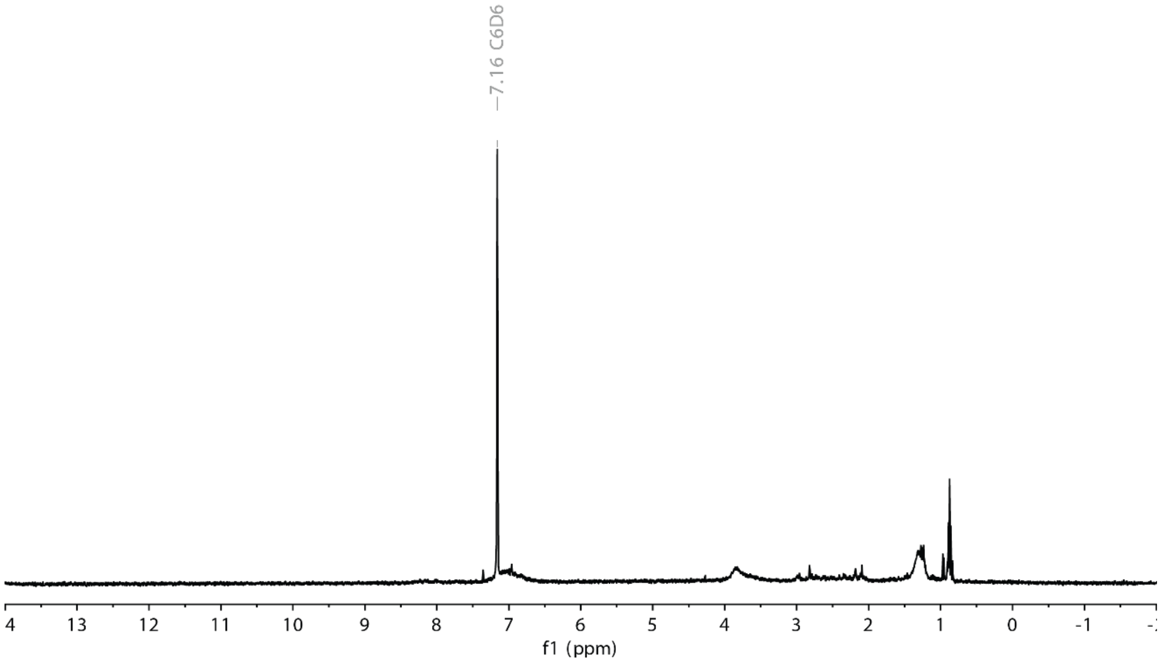

Figure S13. ^{1}H NMR spectrum (400 MHz, C_6D_6, 298 K) of unknown precipitate from the reaction of **1** and $BH_3{\cdot}SMe_2$ or $BH_3{\cdot}THF$.

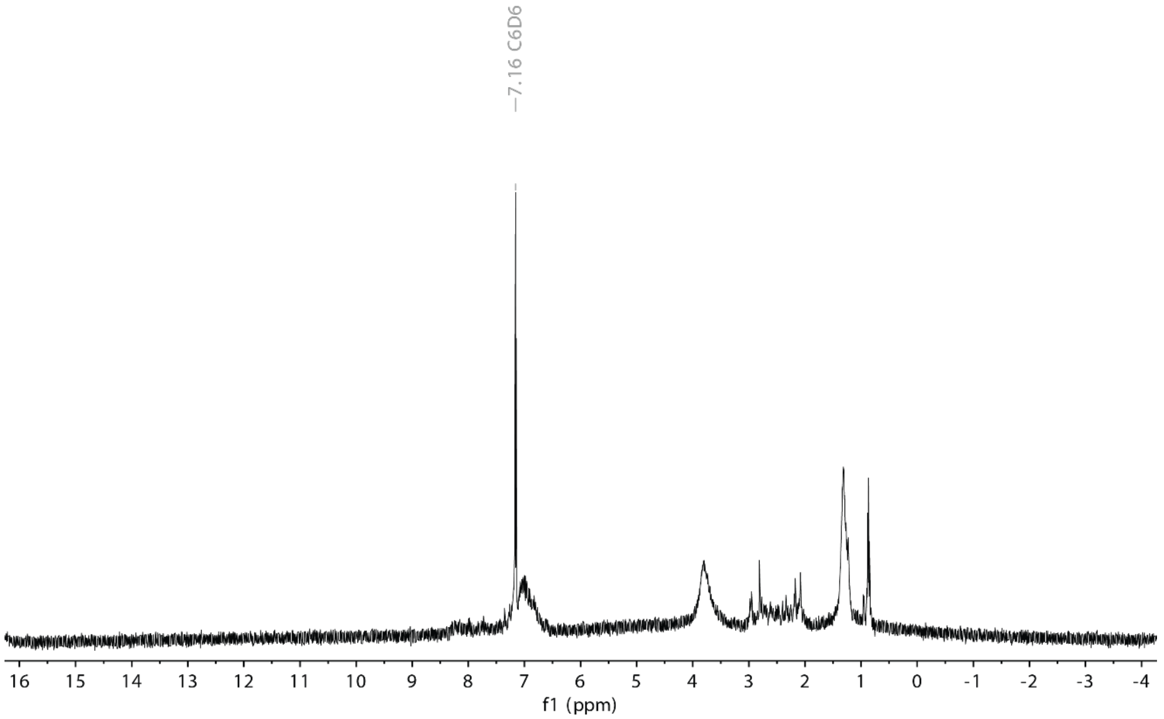

Figure S14. ^{1}H{^{11}B} NMR spectrum (400 MHz, C_6D_6, 298 K) of unknown precipitate from the reaction of **1** and $BH_3{\cdot}SMe_2$ or $BH_3{\cdot}THF$.

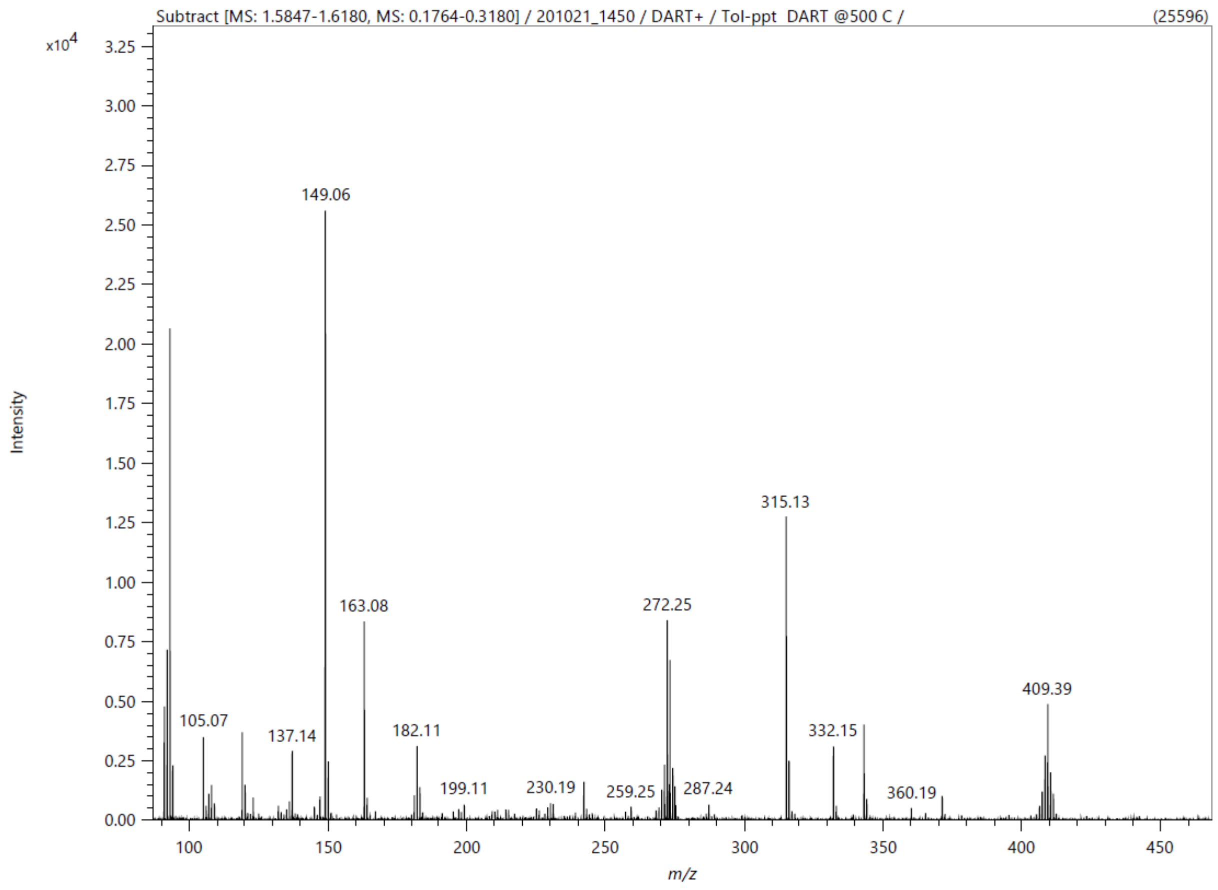

Figure S15. Mass spectrum (DART(+), 500 °C) of the unknown precipitate from the reaction of **1** and $BH_3 \cdot SMe_2$ or $BH_3 \cdot THF$.

Reaction Monitoring NMR Spectra

Compound 1 and H_2

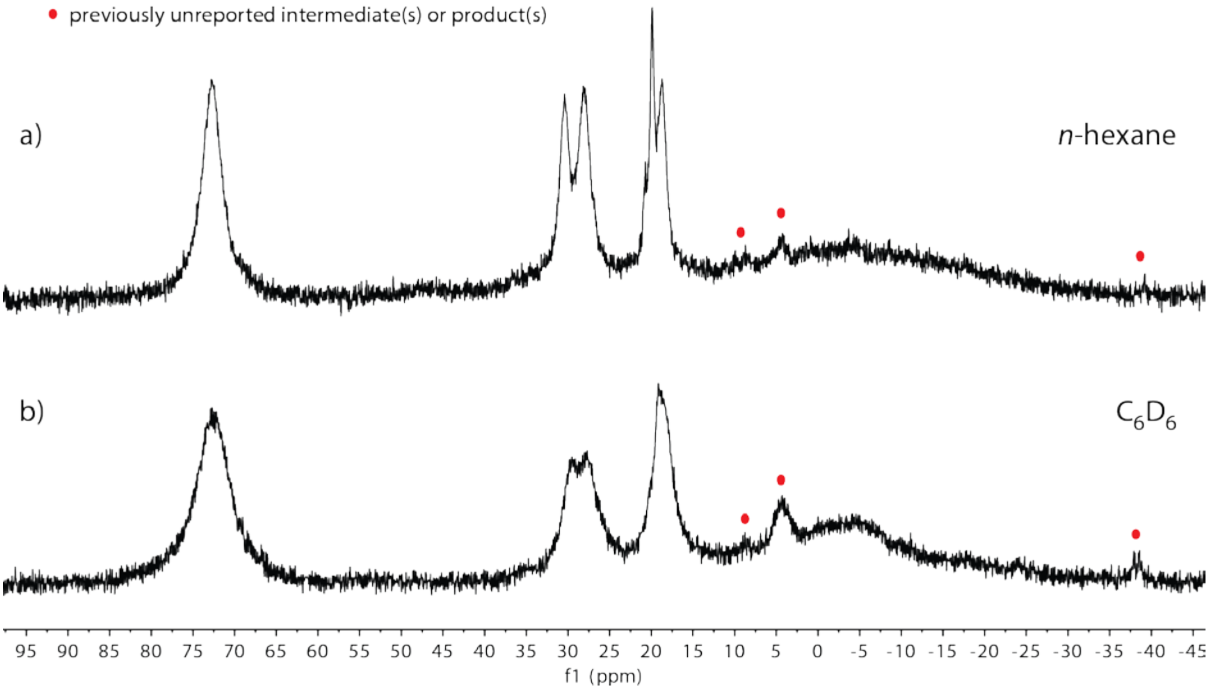

Figure S16. $^{11}B\{^1H\}$ NMR spectra (128 MHz, 298 K) of the reaction of **1** and H_2 (4 atm) in a) n-hexane or b) C_6D_6 solution, after 4 hours.

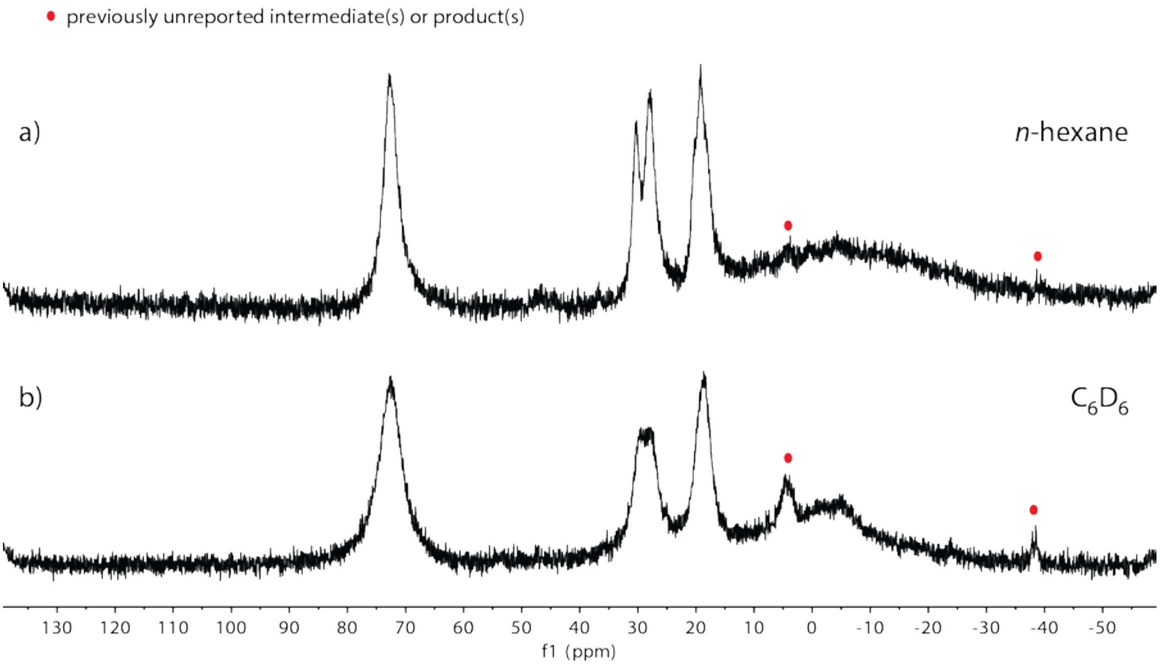

Figure S17. ^{11}B NMR spectra (128 MHz, 298 K) of the reaction of **1** and H_2 (4 atm) in a) n-hexane or b) C_6D_6 solution, after 4 hours.

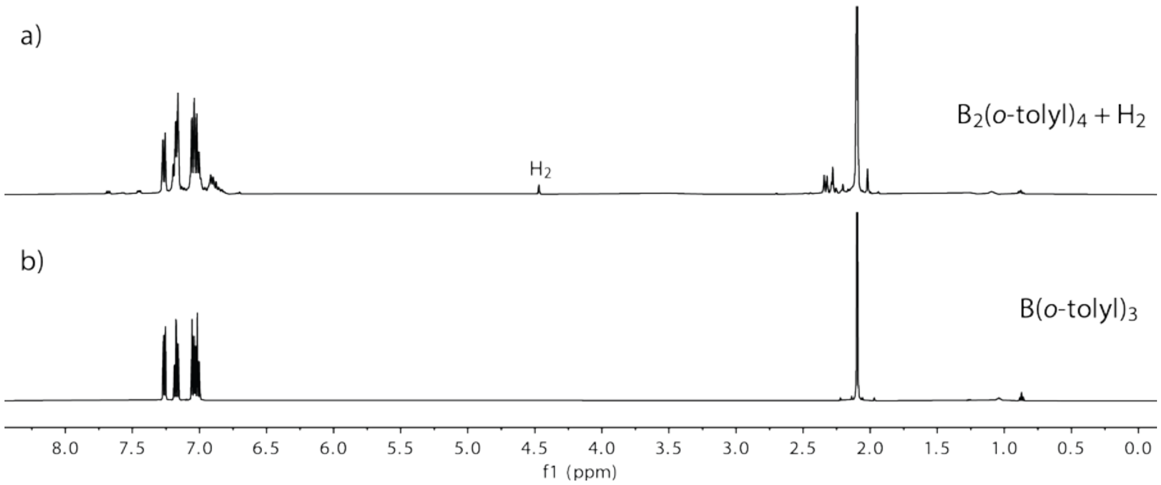

Figure S18. Comparison of the mixture of **1**/H_2 *vs*. **2** by ^{1}H NMR (400 MHz, 298 K, C_6D_6) spectroscopy.

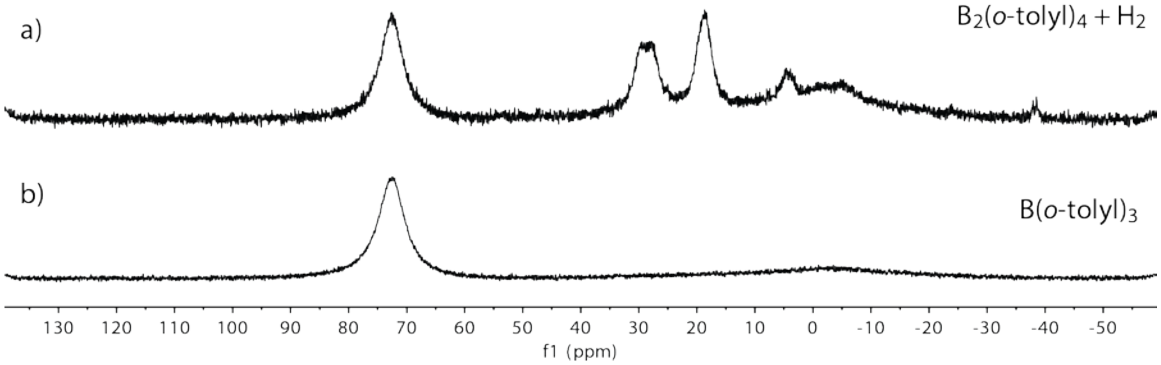

Figure S19. Comparison of the mixture of **1**/H_2 *vs*. **2** by ^{11}B NMR (128 MHz, 298 K, C_6D_6) spectroscopy.

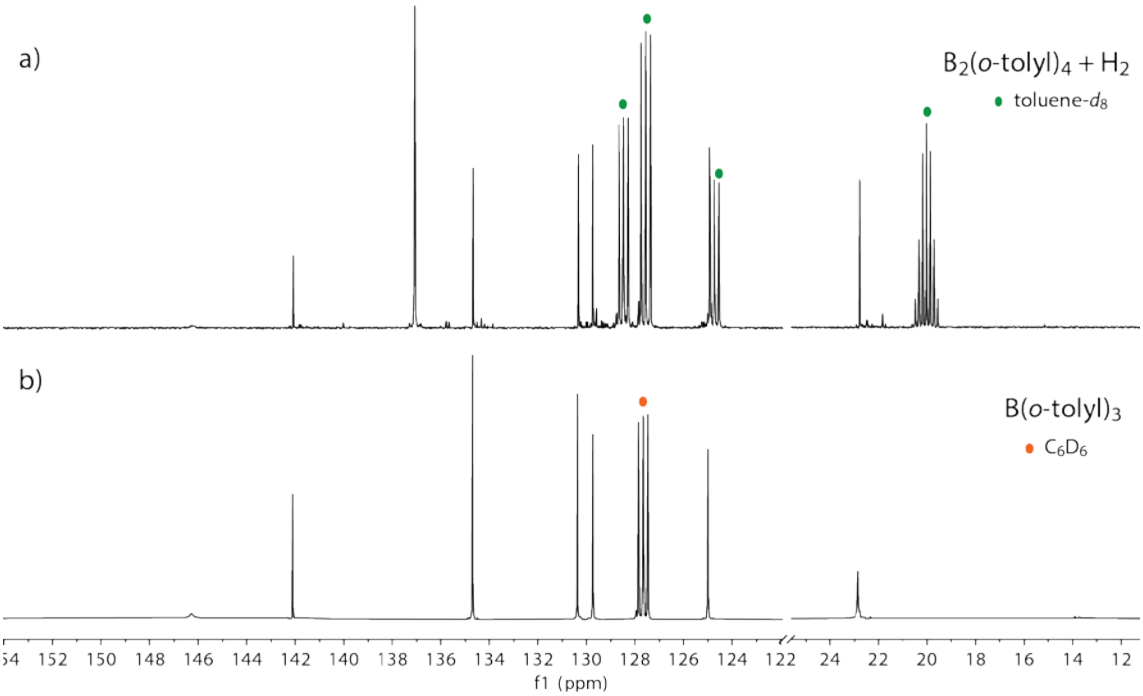

Figure S20. Comparison of the mixture of **1**/H_2 *vs*. **2** by $^{13}C\{^1H\}$ NMR (128 MHz, 298 K, toluene-d_8 or C_6D_6) spectroscopy.

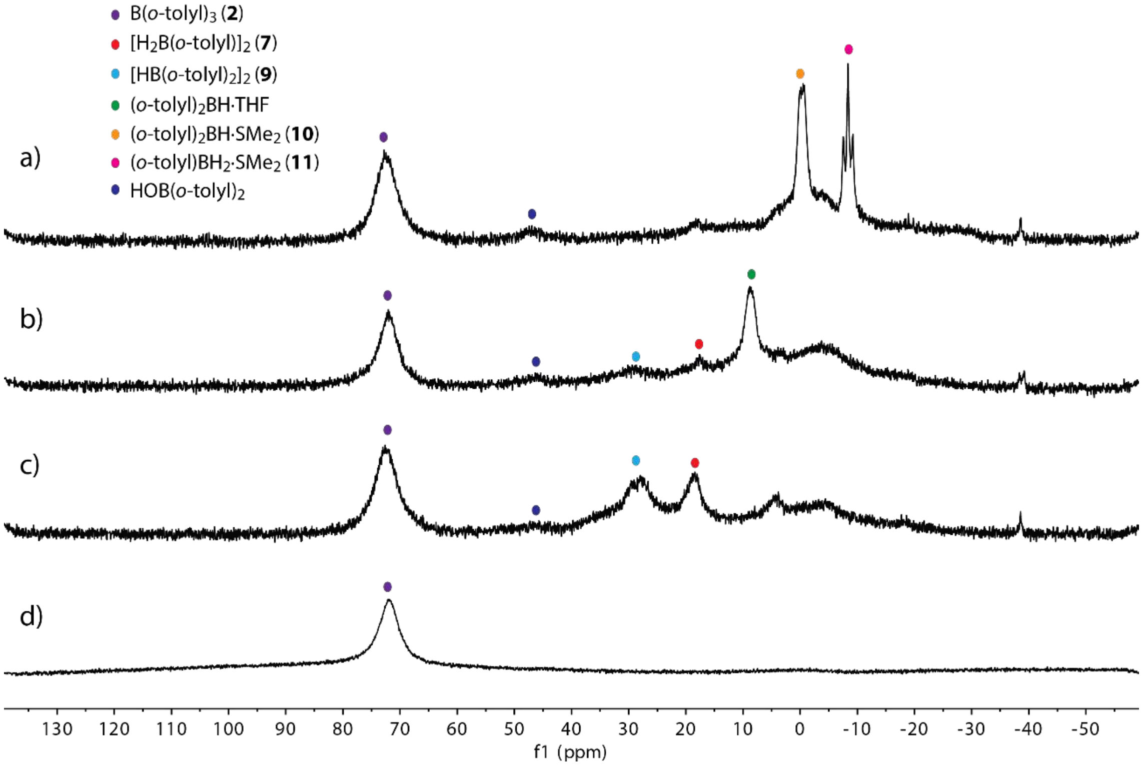

Figure S21. ^{11}B NMR (128 MHz, 298 K, C_6D_6) spectroscopic comparison of: a) **1**/H_2 after 4 hours, with excess SMe_2 added; b) **1**/H_2 after 4 hours, with excess THF added; c) **1**/H_2 after 4 hours; and d) an authentic sample of **2**

Compound 1 and BH_3-Sources

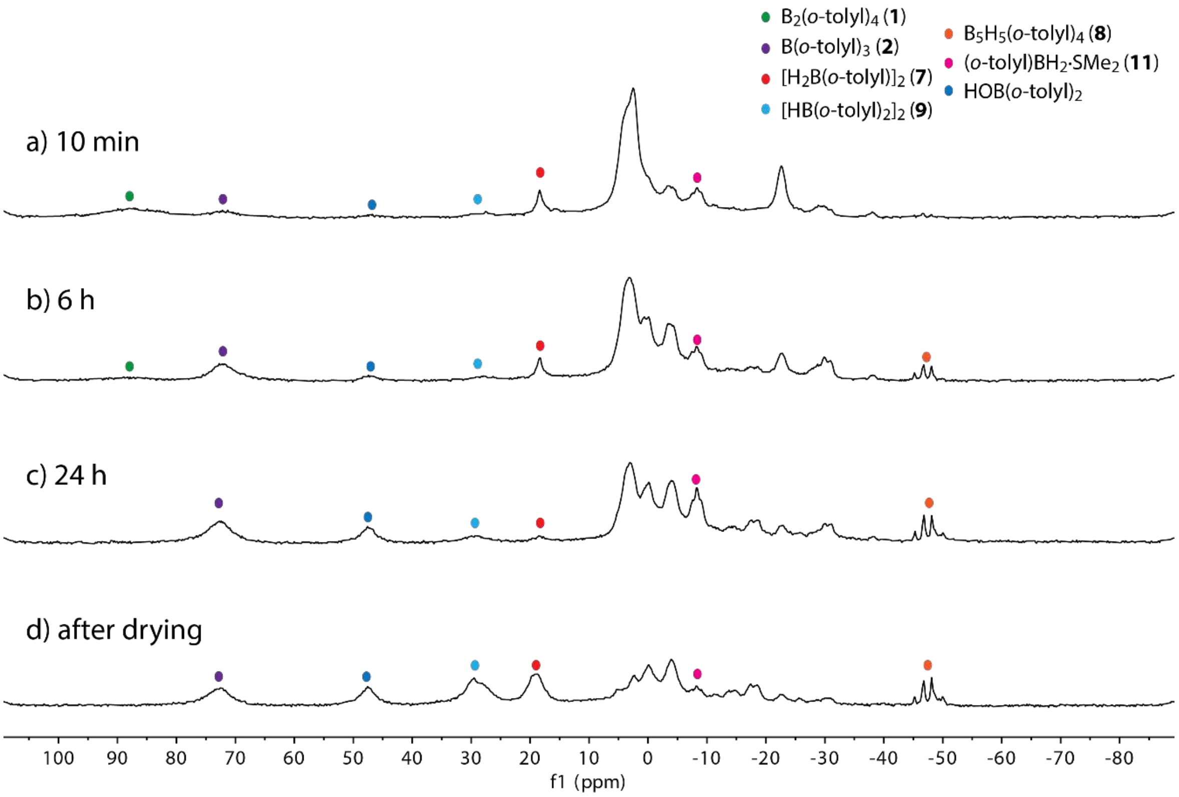

Figure S22. ^{11}B NMR spectra (128 MHz, 298 K) of the 1:1 reaction of **1** and $BH_3{\cdot}SMe_2$ (2M in THF) in C_6D_6, obtained a) 10 minutes, b) 6 hours, and c) 24 hours after initial mixing, and d) after drying *in vacuo*.

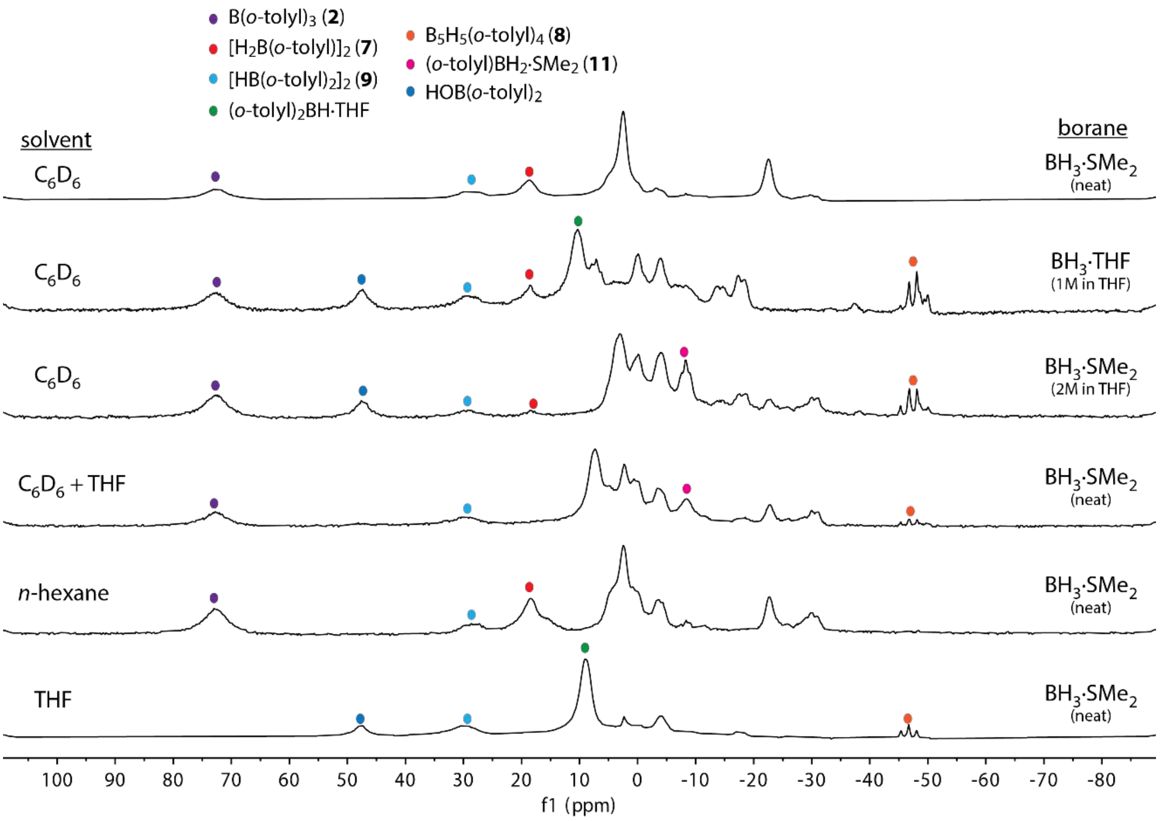

Figure S23. ^{11}B NMR spectra (128 MHz, 298 K) of the 1:1 reactions of **1** and $BH_3{\cdot}L$ (L = SMe_2, THF, or none) in C_6D_6, *n*-hexane or THF reaction solvent, obtained 24 hours after initial mixing.

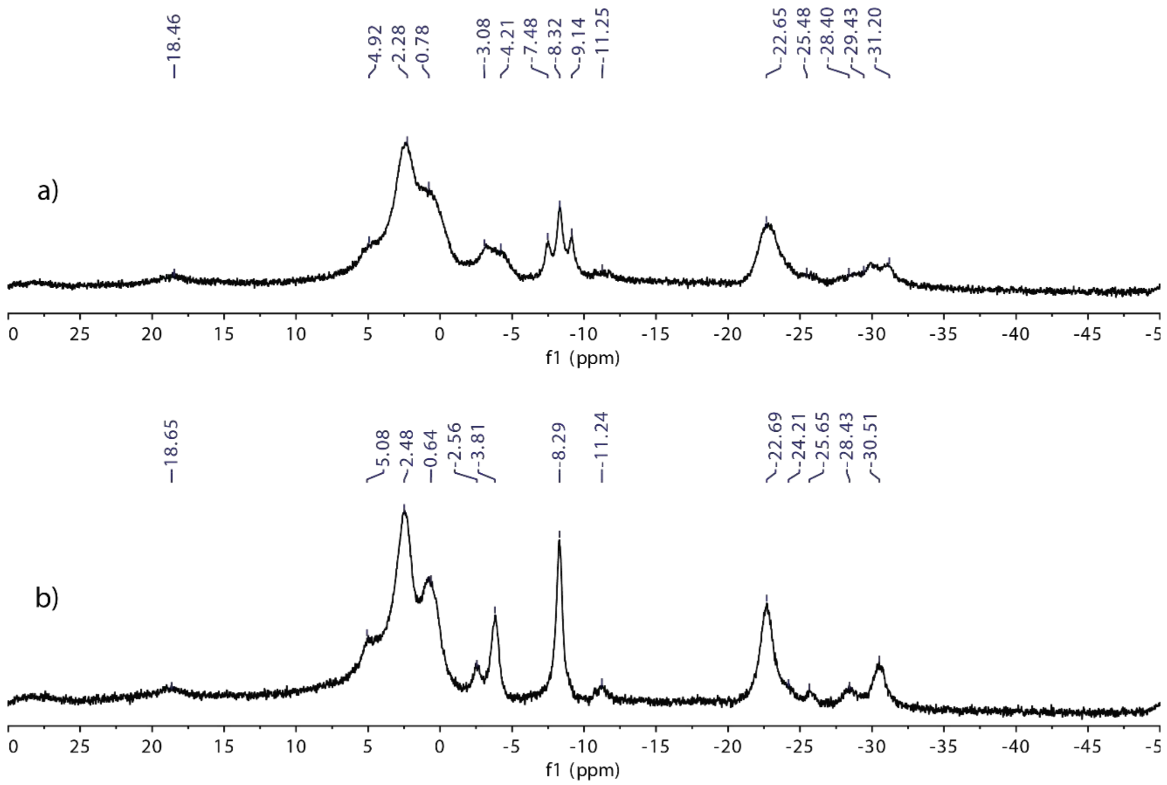

Figure S24. Expansion of the a) ^{11}B and b) $^{11}B\{^1H\}$ NMR spectra for the 1:1 reaction of **1** and neat $BH_3{\cdot}SMe_2$, recorded 24 hours after initial mixing.

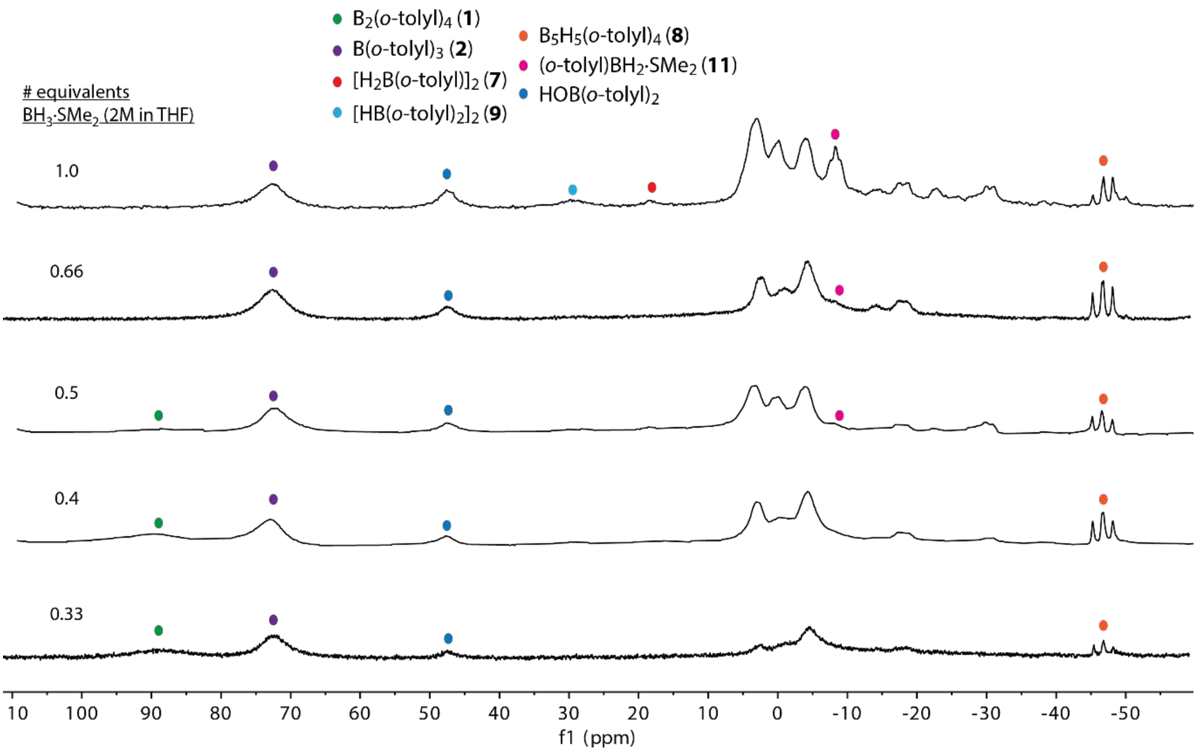

Figure S25. ^{11}B NMR spectra (128 MHz, 298 K) of the 1:x reactions of **1** and $BH_3{\cdot}SMe_2$ (2M in THF) in C_6D_6 or toluene solvent, obtained 24 hours after initial mixing (x = number of equivalents of $BH_3{\cdot}SMe_2$).

Compound 1 and Hydridoboranes

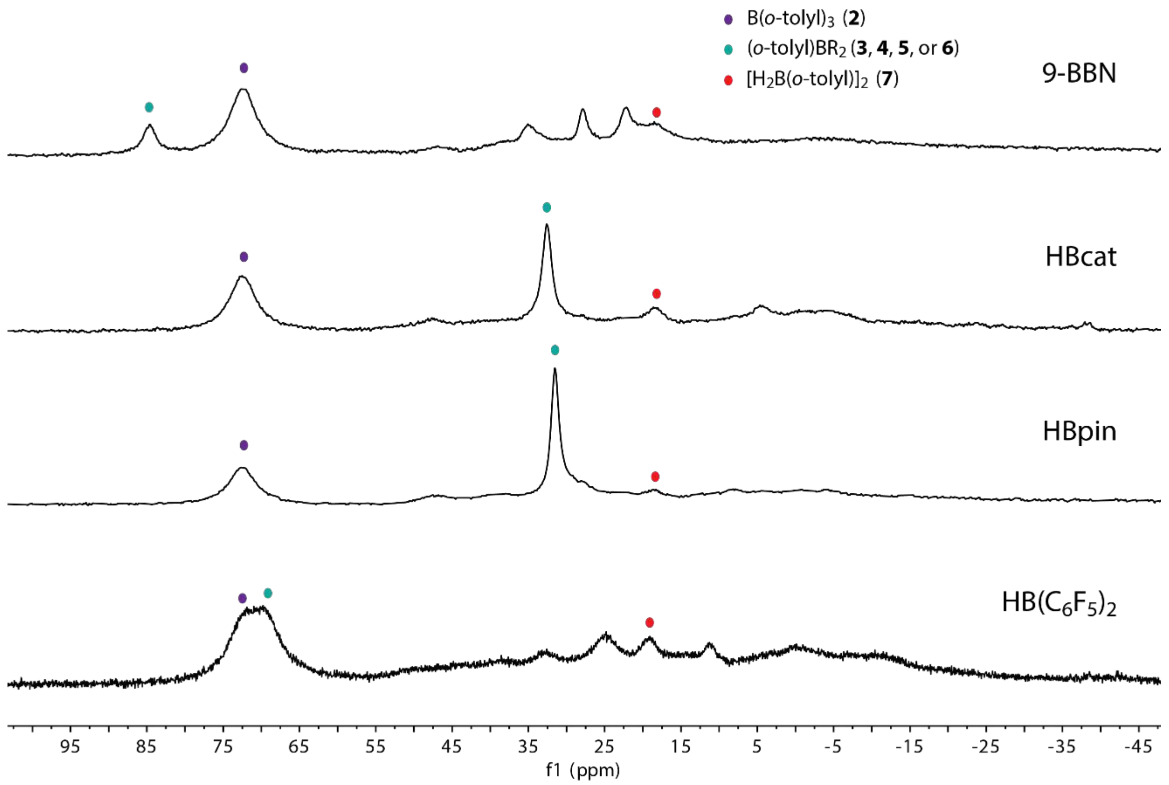

Figure S26. ^{11}B NMR spectra (128 MHz, 298 K) of the 1:1 reactions of **1** and 9-BBN, HBcat, HBpin, and $HB(C_6F_5)_2$.

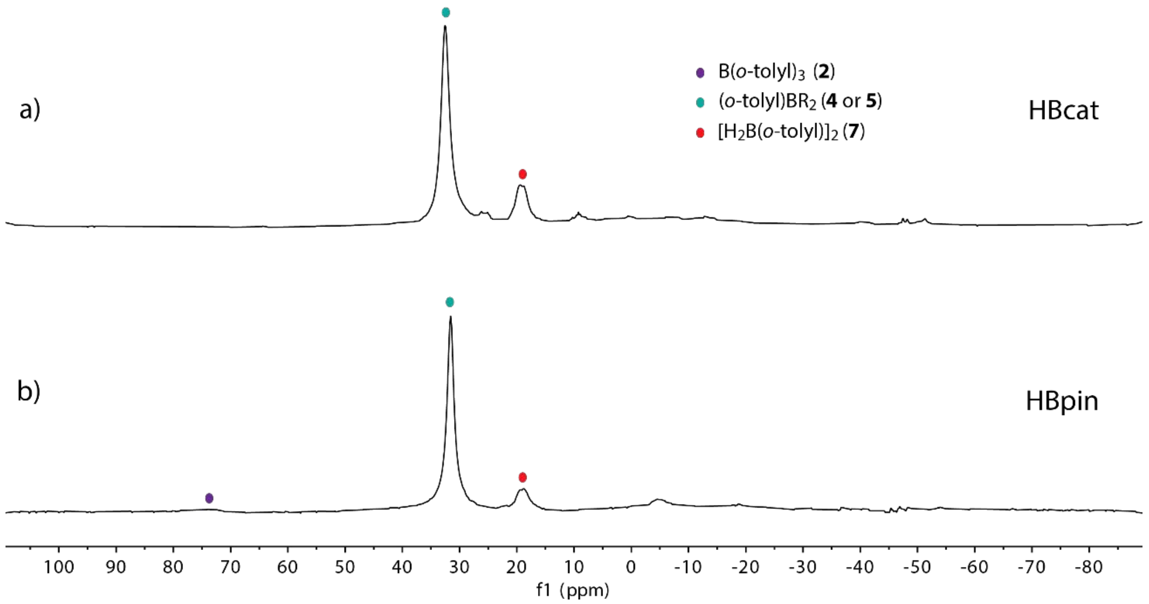

Figure S27. ^{11}B NMR spectra (128 MHz, 298 K, C_6D_6) of the 1:2 reactions of **1** and a) HBcat, or b) HBpin.

Compound 8 Stability and Attempted Reaction with D_2

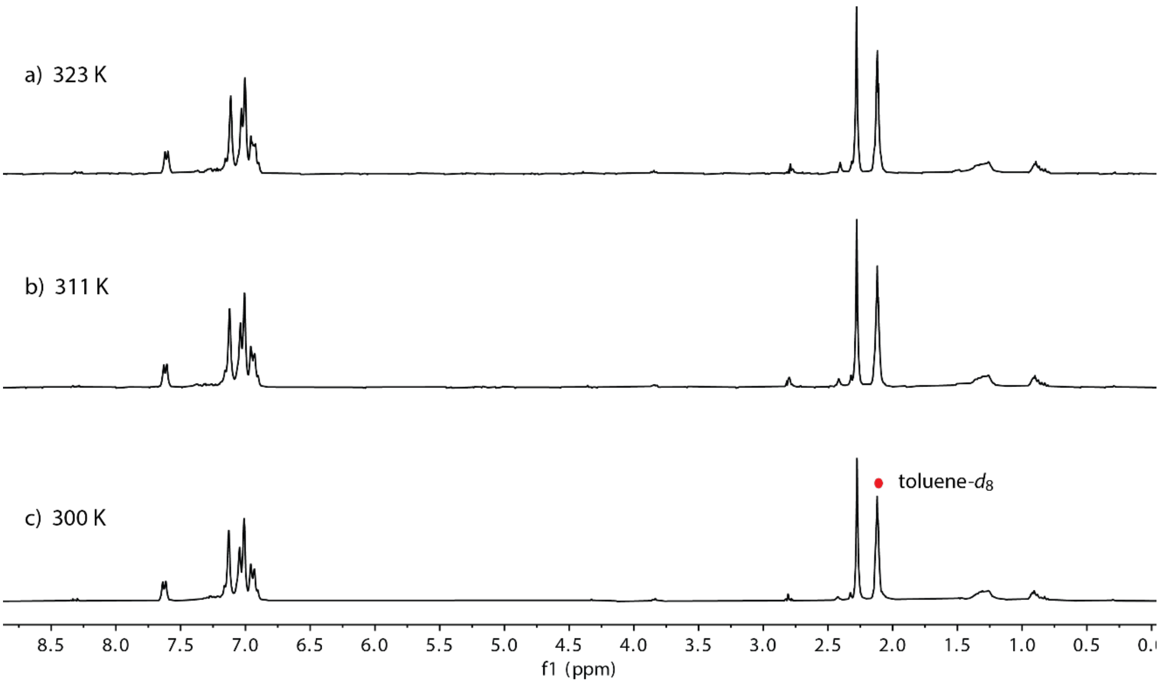

Figure S28. Variable temperature ^{1}H NMR spectra (300 MHz, toluene-d_8) of **8** a) 323 K, b) 311 K, and c) 300 K.

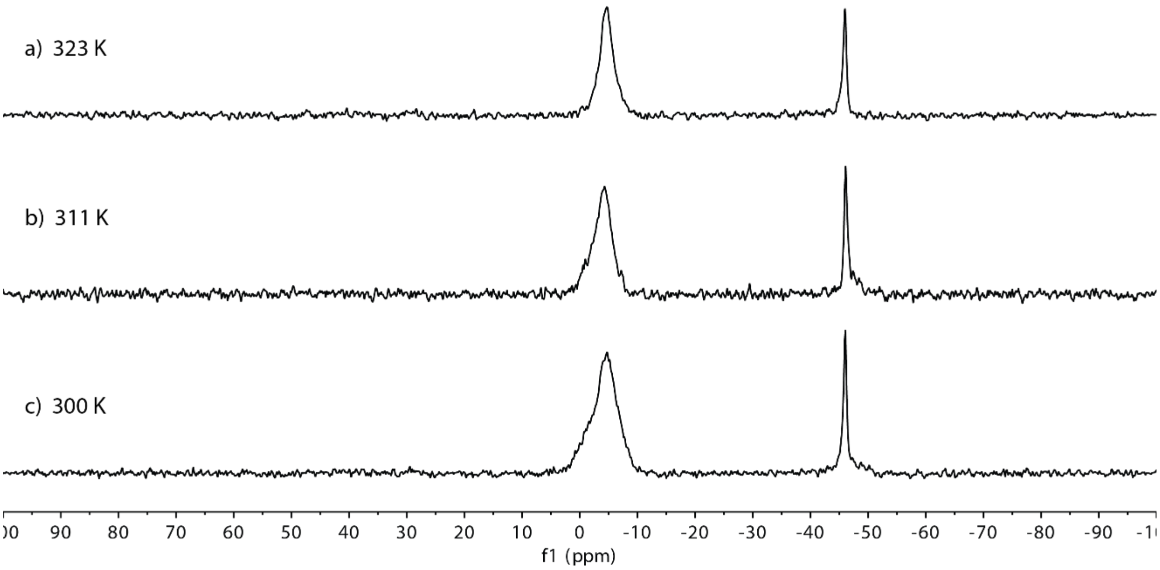

Figure S29. Variable temperature ^{11}B{^{1}H} NMR spectra (96 MHz, toluene-d_8) of **8** a) 323 K, b) 311 K, and c) 300 K.

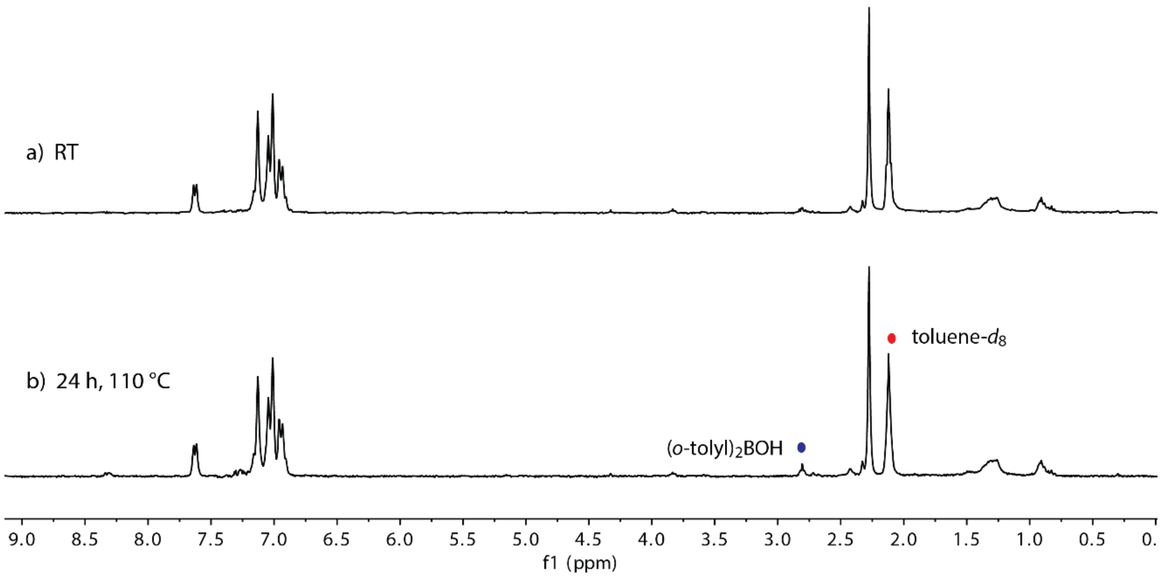

Figure S30. ^{1}H NMR spectra (300 MHz, 298 K, toluene-d_8) of **8** a) at room temperature and b) after heating to 110 °C for 24 hours.

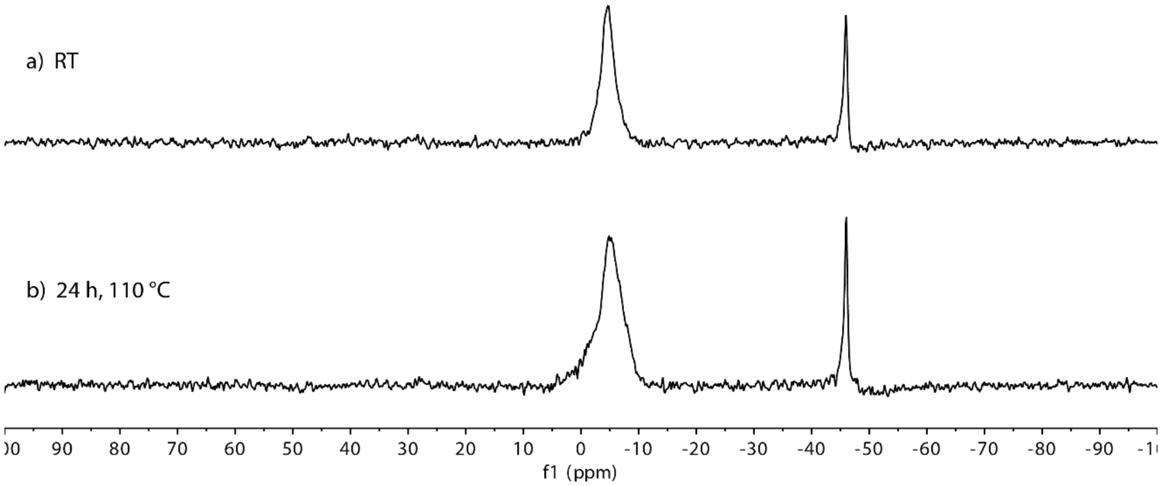

Figure S31. $^{11}B\{^1H\}$ NMR spectra (96 MHz, 298 K, toluene-d_8) of **8** a) at room temperature and b) after heating to 110 °C for 24 hours.

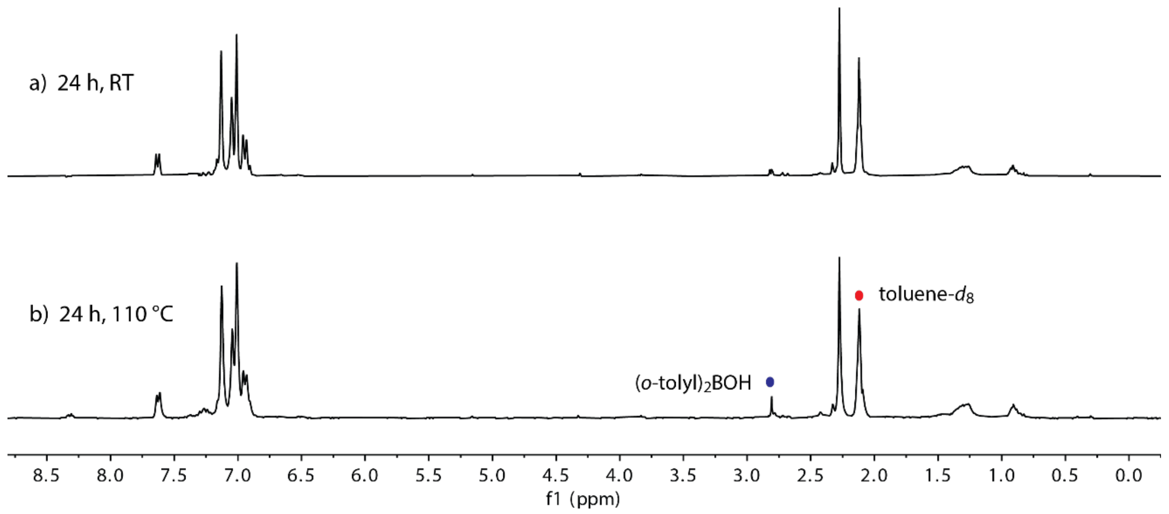

Figure S32. ^{1}H NMR spectra (300 MHz, 298 K, toluene-d_8) of the attempted reaction of **8** and D_2 (1 atm), a) at room temperature for 24 hours and b) after heating to 110 °C for 24 hours.

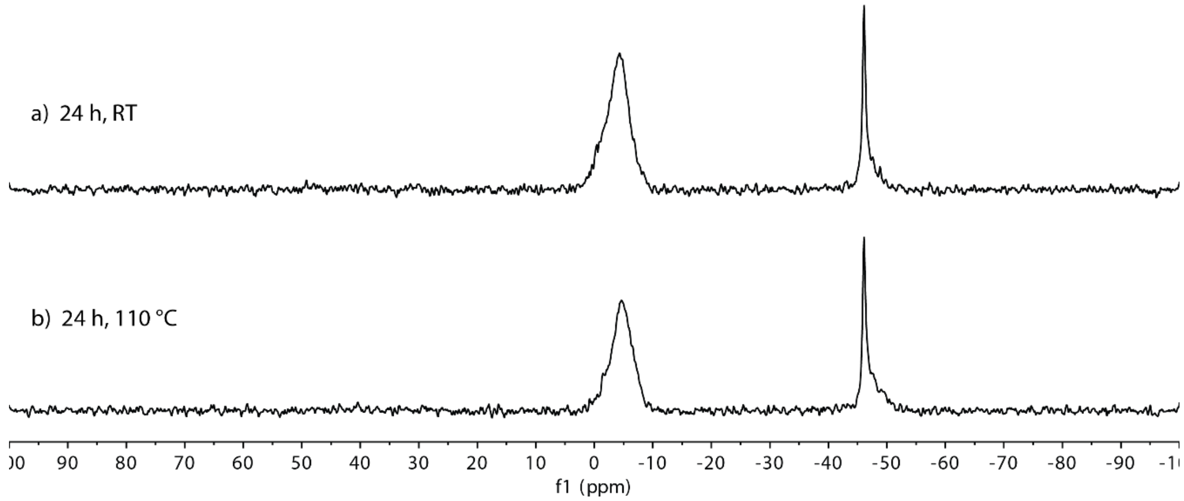

Figure S33. ^{11}B{^{1}H} NMR spectra (96 MHz, 298 K, toluene-d_8) of the attempted reaction of **8** and D_2 (1 atm), a) at room temperature for 24 hours and b) after heating to 110 °C for 24 hours.

Single-Crystal X-Ray Crystallography

X-ray crystallographic data for compounds **2** and **4** were collected on a Bruker Apex2 X-ray diffractometer at 150±2 K using a graphite monochromator with MoK(α) radiation (λ = 0.71073 Å) and the Bruker APEX3 software[7] package. Suitable crystals were selected and mounted in Paratone-N oil on a MiTeGen cryoloop, then placed in the cold (N2) stream of the diffractometer. Unit cell parameters were determined from consecutive scans at different orientations. The data were integrated using the SAINT software package[8] and a multi-scan absorption correction was applied using SADABS.[9] All structures were solved by intrinsic phasing in the SHELXTL suite of programs using XT and refined by full-matrix least-squares

on F^2 using XL.[10] All non-hydrogen atoms were subjected to anisotropic refinement and carbon-bound hydrogen atoms were placed in calculated positions using an appropriate riding model and coupled isotropic temperature factors (U_{iso}(H) = 1.2U_{eq}(C)).

X-ray crystallographic data for compound **8** were collected on a Bruker Kappa APEX-DUO CMOS PHOTO II diffractometer at 150±2 K using CuK(α) radiation (λ = 1.54178 Å) and the Bruker APEX3 software[7] package. The data were integrated and corrected as described above. The boron-bound hydrogen atoms in compound **8** were located in the difference Fourier map and were freely refined.

For all compounds, peaks remaining in the difference Fourier map after final refinements were deemed to be chemically insignificant based on their position and magnitude of electron density.

Reflections 0 0 2 and 0 1 1 in the data set for compound **2** were potentially affected by the beamstop in systematic error (alerts PLAT919 and PLAT934) and were therefore excluded using the OMIT instruction in final refinements.

The absolute structure parameter for **4** is meaningless because the compound is both achiral and a weak anomalous scatterer. The absolute structure parameter is therefore not included herein. Due to a systematic error associated with beamstop obstruction, reflection 0 0 2 was omitted in final structure refinements.

Crystallographic data (CCDC 2049552-2049554) can be obtained free of charge from the Cambridge Crystallography Data Center at www.ccdc.cam.ac.uk/data_request/cif.

Table S1. Crystal and refinement data

	**2**	**4**	**8**
CCDC #	2049554	2049552	2049553
empirical formula	$C_{21}H_{21}B$	$C_{13}H_{11}BO_2$	$C_{28}H_{33}B_5$
formula weight	284.19	210.03	423.59
crystal colour	colourless	colourless	colourless
crystal habit	block	rod	plate
crystal system	monoclinic	orthorhombic	tetragonal
space group	$P2_1/n$	$P2_12_12_1$	$P4/n$
a /Å	9.284(1)	4.6682(7)	15.5007(6)
b /Å	10.434(1)	12.428(2)	15.5007(6)
c /Å	17.729(3)	18.112(2)	5.1190(3)
α /°	90	90	90
β /°	103.808(4)	90	90

γ /°	90	90	90
V /Å^3	1667.8(4)	1050.8(3)	1229.95(12)
Z	4	4	2
D_{calc} /g·cm^{-3}	1.132	1.328	1.144
μ /mm^{-1}	0.063	0.087	0.447
θ_{min} /°	2.286	1.987	4.033
θ_{max} /°	27.538	26.313	68.327
reflections measured	23450	8000	10318
unique reflections (N_{ref})	3840	2128	1129
R_{int}	0.0786	0.0710	0.0805
no. of parameters (N_{par})	202	146	84
R_1 [$I > 2\sigma(I)$]	0.0570	0.0464	0.0467
wR_2 (all data)	0.1625	0.0993	0.1338
GOF on F^2 (S)	1.024	0.989	1.100

$R_{int} = \Sigma|F_o^2 - F_o^2(\text{mean})|/\Sigma F_o^2$, $R_1 = \Sigma||F_o| - |F_c||/\Sigma|F_o|$,
w$R_2 = [\Sigma w(F_o^2 - F_c^2)^2/\Sigma w(F_o^2)^2]^{1/2}$, $S = [\Sigma w(F_o^2 - F_c^2)^2/(N_{ref} - N_{par})]^{1/2}$

Computational Details

The quantum chemical DFT calculations have been performed with the TURBOMOLE 7.3 suite of programs.[11] The structures are fully optimized at the TPSS-D3/def2-TZVP + COSMO(toluene) level of theory, which combines the TPSS meta-GGA density functional[12] with the BJ-damped DFT-D3 dispersion correction[13] and the def2-TZVP basis set,[14] using the Conductor-like Screening Model (COSMO) continuum solvation model[15] for toluene solvent (dielectric constant ε = 2.38 and solvent diameter R_{solv} = 3.48 Å). The density-fitting RI-J approach[14a, 16] is used to accelerate the geometry optimization and numerical harmonic frequency calculations[17] in solution. The optimized structures are characterized by frequency analysis to identify the nature of located stationary points (no imaginary frequency for true minima and only one imaginary frequency for transition state) and to provide thermal corrections (at 298.15 K and 1 atm) according to the modified ideal gas–rigid rotor–harmonic oscillator model.[18] This choice of dispersion-corrected meta-GGA functional makes the efficient exploration of all potential reaction paths possible.

The final solvation free energies in toluene are computed with the COSMO-RS solvation model[19] (parameter file: BP_TZVP_C30_1601.ctd) using the COSMOtherm program package[20] on the above TPSS-D3 optimized structures, and corrected by +1.89 kcal/mol to

account for higher reference solute concentration of 1 mol/L usually used in solution. To check the effects of the chosen DFT functional on the reaction energies and barriers, single-point calculations at the meta-GGA TPSS-D3[12] and hybrid-meta-GGA PW6B95-D3[21] levels are performed using a larger def2-QZVP basis set.[14b, 22] The final reaction Gibbs free energies (ΔG) are determined from the electronic single-point energies plus TPSS-D3 thermal corrections and COSMO-RS solvation free energies. The computed reaction free energies from both DFT functionals are in good mutual agreement with a standard deviation of only 1.7 kcal/mol, despite about 1.9 kcal/mol higher barriers are expected at the PW6B95-D3 level. In our discussion, higher-level PW6B95-D3 Gibbs free energies (in kcal/mol, at 298.15 K and 1 mol/L concentration) will be used in our discussion unless specified otherwise. The applied DFT methods in combination with the large AO basis set provide usually accurate electronic energies leading to errors for chemical energies (including barriers) on the order of typically 1–2 kcal/mol. This has been tested thoroughly for the huge data base GMTKN55[23] which is the common standard in the field of DFT benchmarking.

To help experimental ^{11}B NMR assignment, nuclear magnetic shielding constants for various boron-containing complexes are also computed using the GIAO (Gauge Including Atomic Orbital) method[24] at the TPSS/def2-QZVP level; the final ^{11}B NMR chemical shifts are computed using the experimentally known ^{11}B NMR signal of $[HB(C_6F_5)_2]_2$ at 18.0 ppm[25] (DFT-computed shielding 89.0 ppm) in C_6D_6 solution as reference. We also note that DFT computed dipole of compound **8** is 3.18 D at the TPSS-D3/def2-TZVP + COSMO level, along the apical BH bond (positive charge on the H end); in gas-phase, it is 2.13 and 2.23 D at the TPSS-D3/def2-QZVP and PW6B95-D3/def2-QZVP level, respectively.

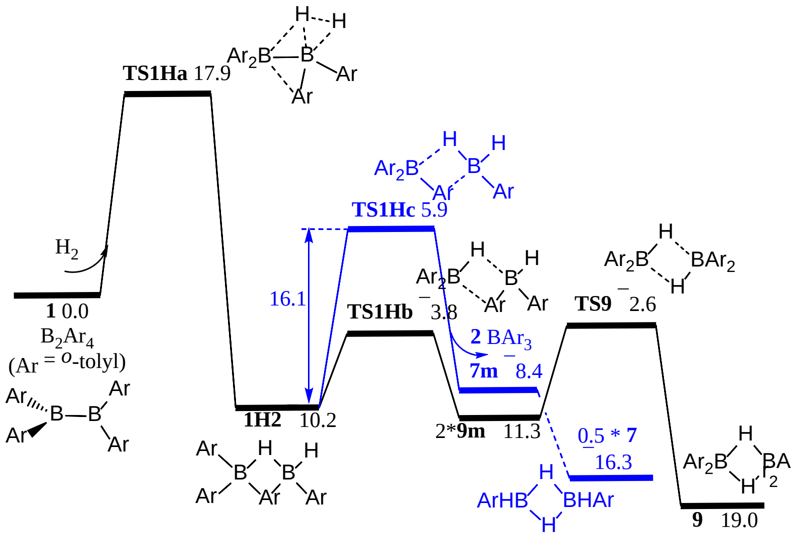

Figure S34. Gibbs free energy paths (in kcal/mol, at 298 K and 1M concentration) for the reaction of H_2 and B_2(o-tolyl)$_4$ (**1**, where Ar = *o*-tolyl) in toluene solution, computed at the PW6B95-D3/def2-QZVP + COSMO-RS // TPSS-D3/def2-TZVP + COSMO level of theory.

As shown in Figure S34 above, the reaction of H_2 + B_2(o-tolyl)$_4$ is rate-limited by the initial HH/BB addition (via transition structure **TS1Ha**) over a low barrier of 17.9 kcal/mol. From the H/Ar bridged diborane(6) **1H2**, two faster competing BH/BC cleavages (via **TS1Hb** and **TS1Hc**) may lead to the main product **9** and kinetically less favorable **2** + 0.5***7** products, respectively. Moreover, the eliminated reactive monomeric boranes **7m** and **9m** may react with **1** *via* H/Ar exchange, followed by further oligomerization as side reactions that may account for minor unidentified products. The DFT computed reaction paths as well as computed ^{11}B NMR chemical shifts (see Table S3) support our new assignment of ^{11}B signals at 18.6 and 28.5 ppm to the main products [H_2B(o-tolyl)]$_2$ **7** and [HB(o-tolyl)$_2$]$_2$ **9**, respectively.

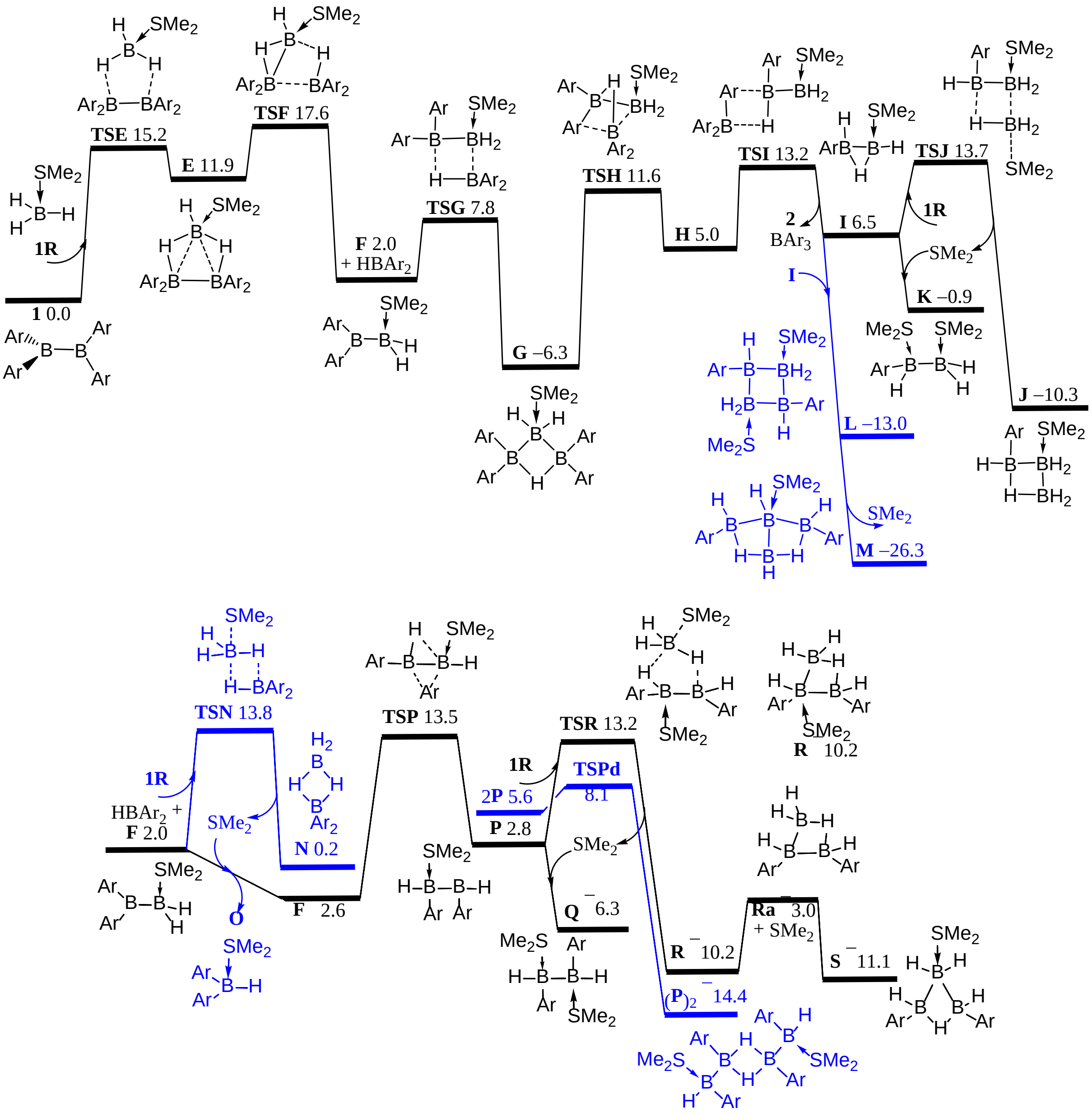

Figure S35. Gibbs free energy paths (in kcal/mol, at 298 K and 1M concentration) for the reaction of neat $BH_3.SMe_2$ (**1R**) and B_2(*o*-tolyl)$_4$ (**1**, where Ar = *o*-tolyl) in toluene solution, computed at the PW6B95-D3/def2-QZVP + COSMO-RS // TPSS-D3/def2-TZVP + COSMO level of theory.

The reaction of **1** with neat $BH_3{\cdot}SMe_2$, in the total absence of THF, afforded no trace of **8**. Instead, ^{11}B NMR data reveals a mixture of **2** in addition to two new strong signals at 2.3 and –22.6 ppm (see Figure S24). DFT calculations suggest that the strong signals at 2.3 and –22.6 ppm are very likely due to the symmetric adduct **S** (Figure S35) with δ_{calc} = 6.8, 6.8, –23.4 ppm; asymmetric adduct **J** is also possible with δ_{calc} = 2.0, 1.4, –24.3 ppm. Both adducts can be readily formed from rapid trapping of intermediate diborane(4) adducts with reactant **1R**

$BH_3 \cdot SMe_2$. Dimerization of diborane(4) adducts **I** and **P** may lead to higher aggregates as minor products observed by ^{11}B NMR spectroscopy. Both cyclic triborane(7) adducts **S** and **J** show only moderate kinetic stability (prevented by barriers of 24.0 and 24.3 kcal/mol via **TSJ** and **TSR**) with respect to back-dissociation into transient diborane(4) adducts **P** and **I**, respectively.

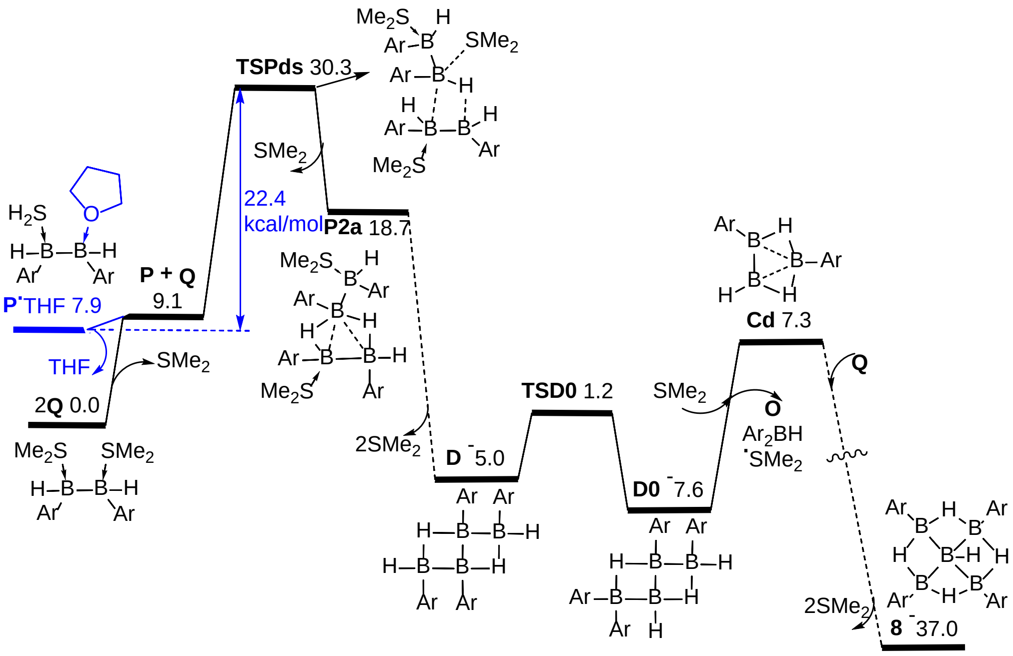

Figure S36. Proposed Gibbs free energy paths (in kcal/mol, at 298 K and 1M concentration) for the THF-promoted formation of **8** B_5H_5(*o*-tolyl)$_4$ in toluene solution, computed at the PW6B95-D3/def2-QZVP + COSMO-RS // TPSS-D3/def2-TZVP + COSMO level of theory. (Ar = *o*-tolyl).

Though the precise mechanism for the formation of **8** B_5H_5(*o*-tolyl)$_4$ is unclear, it is very likely formed via the oligomerization of intermediate diborane(4) adduct **P** (Figure S36). The dimerization of doubly SMe_2-stabilized diborane **Q** is prevented by a high barrier of 30.3 kcal/mol (via **TSQd**); when partially stabilized by concentrated THF into **P**·THF, the barrier can be effectively reduced to about 22.4 kcal/mol, making further dimerization into **D** (and release of SMe_2) possible. Followed by rapid aryl/H exchange (**TSD0**) and Ar_2BH elimination with SMe_2 (or THF), final BH/BB coupling with **P** or **Q** may eventually lead to the highly stable pentaborane(9) product **8**. Similar reaction path can also be expected for the oligomerization of **I** and **K** (see Table S2 for detailed energies).

Table S2. TPSS-D3/def2-TZVP + COSMO computed imaginary frequency (ImF), zero-point energies (ZPE), gas-phase enthalpic (Hc) and Gibbs free-energy (Gc) corrections; the COSMO-RS computed solvation enthalpic (Hsol) and Gibbs free-energy (Gsol) corrections in TOLUENE solution; TPSS-D3/def2-QZVP and PW6B95-D3/def2-QZVP single-point energies (TPSS-D3 and PW6B95-D3); the total PW6B95-D3 free energies G_P; the relative electronic energies (ΔE_T and ΔE_P) and Gibbs free-energies (ΔG_T and ΔG_P) at the TPSS-D3 and PW6B95-D3 levels. See also Figure S28-S30 for labelings of most structures.

Reactions	ImF cm^{-1}	ZPE kcal /mol	Hc kcal /mol	Gc kcal /mol	Hsol kcal /mol	Gsol kcal /mol	TPSS-D3 Eh	PW6B95-D3 Eh	G_P Eh	ΔE_T kcal /mol	ΔE_P kcal /mol	*ΔG_P kcal /mol*	ΔG_T kcal /mol
*Low-lying Conformers of **1** B_2Ar_4 (Ar = o-tolyl group)*													
**1**	0.0	298.03	316.16	264.74	-27.16	-19.72	-1134.34189	-1135.59417	-1135.20071	0.00	0.00	*0.00*	0.00
**1a**	0.0	298.24	316.48	264.35	-27.55	-19.95	-1134.33955	-1135.58970	-1135.19723	1.47	2.80	*2.19*	0.85
**1b**	0.0	297.51	315.67	264.94	-26.09	-18.97	-1134.34115	-1135.59023	-1135.19524	0.47	2.47	*3.43*	1.43
*Reaction of **1** + HBcat*													
**1** + HBcat	0.0	361.96	384.33	310.27	-37.10	-26.26	-1541.63935	-1543.32785	-1542.86924	0.00	0.00	*0.00*	0.00
**TSA**	-217.6	360.80	383.52	323.01	-33.44	-24.83	-1541.63234	-1543.31607	-1542.83789	4.40	7.39	*19.67*	16.68
**A0**	0.0	363.63	385.69	327.26	-32.54	-24.30	-1541.63962	-1543.32371	-1542.83790	-0.17	2.60	*19.66*	16.89
**TSAb**	-156.2	361.54	383.87	324.56	-31.97	-23.67	-1541.62176	-1543.30437	-1542.82186	11.04	14.74	*29.73*	26.03
**TSAc**	-145.1	360.90	383.36	323.50	-33.42	-24.93	-1541.62617	-1543.31295	-1542.83413	8.27	9.35	*22.03*	20.94
**A + 4**	0.0	363.76	385.30	311.65	-39.78	-28.16	-1541.63454	-1543.32407	-1542.86627	3.02	2.37	*1.86*	2.51
**(A)**$_2$ **+ 4**	0.0	364.02	385.85	319.80	-34.50	-25.08	-1541.66421	-1543.35365	-1542.87946	-15.60	-16.19	*-6.41*	-5.83
*Dimerization of **A** via BH/BB addition*													
**2** (or BAr_3)	0.0	222.14	235.37	194.22	-20.90	-14.88	-838.32874	-839.25162	-838.96280				
2*(**1** + HBcat - **4**)	0.0	458.80	487.74	401.85	-40.64	-28.83	-1727.55469	-1729.45720	-1728.85672	0.00	0.00	*0.00*	0.00
2A	0.0	462.41	489.68	404.62	-46.00	-32.62	-1727.54507	-1729.44963	-1728.85078	6.04	4.75	*3.73*	0.00
**TSAd**	-72.7	459.27	487.47	416.40	-37.17	-27.87	-1727.56335	-1729.46383	-1728.84165	-5.44	-4.16	*9.46*	8.18
**(A)**$_2$	0.0	462.93	490.80	420.92	-35.44	-26.46	-1727.60442	-1729.50879	-1728.87716	-31.21	-32.37	*-12.83*	-11.66
**Ad0** + $HBAr_2$	0.0	461.86	489.43	404.45	-43.86	-31.13	-1727.56188	-1729.46588	-1728.86493	-4.51	-5.45	*-5.15*	-4.22
**Ad** + **2**	0.0	462.40	489.93	404.83	-45.50	-32.47	-1727.57584	-1729.48477	-1728.88534	-13.27	-17.30	*-17.96*	-13.93
**Ad1** + **2**	0.0	460.02	488.16	402.08	-44.13	-31.32	-1727.55105	-1729.45381	-1728.85694	2.28	2.13	*-0.14*	0.02

**2** + 0.5(**Ad1**)$_2$	0.0	462.22	490.21	412.39	-41.37	-29.96	-1727.58249	-1729.48646	-1728.87249	-17.45	-18.36	*-9.89*	-8.98
(**A**)$_{2h}$	0.0	465.12	491.94	425.16	-36.98	-27.83	-1727.61606	-1729.52037	-1728.88417	-38.51	-39.64	*-17.22*	-16.09
**Ah** + **7m**	0.0	462.24	489.67	406.13	-41.22	-29.28	-1727.55992	-1729.46290	-1728.85633	-3.28	-3.58	*0.25*	0.54
**Ah** + 0.5*7	0.0	463.70	491.02	414.26	-40.27	-29.20	-1727.58336	-1729.48705	-1728.86890	-17.99	-18.73	*-7.64*	-6.90
*kinetically less favorable dimerization of **A** via double BH/BB*													
2**A**	0.0	462.41	489.68	404.62	-46.00	-32.62	-1727.54507	-1729.44963	-1728.85078	6.04	4.75	*3.73*	0.00
**TSAdh**	-68.4	459.95	488.30	417.10	-36.83	-27.56	-1727.55183	-1729.44787	-1728.82409	1.80	5.85	*20.48*	16.42
(**A**)$_{2h}$	0.0	465.12	491.94	425.16	-36.98	-27.83	-1727.61606	-1729.52037	-1728.88417	-38.51	-39.64	*-17.22*	-16.09
*Isomers of **Ad**: cyclic structure is preferred.*													
**Ad1**	0.0	237.88	252.79	207.86	-23.23	-16.44	-889.22231	-890.20219	-889.89414	0.00	0.00	*0.00*	0.00
**Ad2**	0.0	239.25	253.73	209.41	-24.23	-17.11	-889.23033	-890.21136	-889.90191	-5.03	-5.75	*-4.88*	-4.15
**Ad**	0.0	240.26	254.56	210.61	-24.60	-17.59	-889.24710	-890.23315	-889.92254	-15.55	-19.43	*-17.82*	-13.95
*Reaction of **A** + HBcat*													
**1** + 2HBcat - **4**	0.0	293.33	312.04	246.46	-30.25	-20.95	-1271.07480	-1272.46228	-1272.09689	0.00	0.00	*0.00*	0.00
**A** + HBcat	0.0	295.14	313.01	247.84	-32.93	-22.85	-1271.06999	-1272.45850	-1272.09392	3.02	2.37	*1.86*	2.51
**TSB0**	-49.3	293.31	311.97	259.04	-29.53	-21.61	-1271.07378	-1272.45816	-1272.07678	0.64	2.59	*12.62*	10.67
**B0**	0.0	295.79	313.93	262.57	-30.08	-22.07	-1271.07721	-1272.46370	-1272.07743	-1.51	-0.89	*12.21*	11.59
**TSB**	-29.7	293.05	311.70	258.80	-30.39	-22.34	-1271.06776	-1272.45189	-1272.07206	4.42	6.52	*15.58*	13.48
**B + 4**	0.0	294.14	312.29	245.95	-33.93	-23.62	-1271.06606	-1272.45521	-1272.09487	5.49	4.44	*1.26*	2.31
**TSC + 4**	-264.2	293.12	311.08	244.60	-33.46	-23.30	-1271.05448	-1272.44504	-1272.08636	12.75	10.82	*6.61*	8.54
**C + 4**	0.0	295.48	313.07	247.74	-34.43	-23.91	-1271.07212	-1272.46220	-1272.09949	1.68	0.05	*-1.63*	0.00
0.5(**B**)$_2$ + **4**	0.0	295.83	313.66	255.24	-31.68	-22.72	-1271.10589	-1272.49569	-1272.12063	-22.53	-23.34	*-16.76*	-15.94
0.5(**C0**)$_2$ + **4**	0.0	297.11	314.50	257.12	-31.00	-22.26	-1271.11741	-1272.50856	-1272.12977	-26.74	-29.04	*-20.63*	-18.33
Dimerization of C via BH/BB addition													
2**C**	0.0	325.84	345.24	276.79	-35.31	-24.13	-1186.42023	-1187.72590	-1187.31723	0.00	0.00	*0.00*	0.00
**TSCd**	-295.5	323.32	343.74	287.49	-28.50	-20.80	-1186.43340	-1187.73501	-1187.30700	-8.26	-5.72	*6.42*	3.87
(**C**)$_2$	0.0	327.50	346.80	293.58	-29.71	-21.78	-1186.49393	-1187.80101	-1187.36485	-46.25	-47.13	*-29.88*	-28.99
**Ad** + H_2BAr	0.0	327.53	346.75	278.76	-34.33	-23.71	-1186.44847	-1187.75871	-1187.34623	-17.72	-20.59	*-18.20*	-15.33
**Ad** + 0.5*7	0.0	328.98	348.10	286.88	-33.38	-23.62	-1186.47191	-1187.78286	-1187.35881	-32.43	-35.74	*-26.09*	-22.77

**Cd** + $HBAr_2$	0.0	327.62	346.70	278.59	-35.59	-24.61	-1186.45104	-1187.76170	-1187.35093	-19.33	-22.47	*-21.14*	-18.01
**Cd0** + $HBAr_2$	0.0	327.49	346.64	278.49	-34.69	-23.97	-1186.45196	-1187.76441	-1187.35279	-19.91	-24.17	*-22.31*	-18.05
*Pentaborane(9) $B_5H_5Ar_4$ formation: Barrierless BH/BB coupling of **C** and **Cd***													
**C** + **Cd**	0.0	335.40	355.30	285.48	-37.18	-25.65	-1211.89482	-1213.23405	-1212.81397	0.00	0.00	*0.00*	0.00
**TS8a**	-475.8	334.03	354.17	298.36	-28.86	-20.37	-1211.97089	-1213.30761	-1212.86160	-47.74	-46.16	*-29.89*	-31.47
**C**·**Cd**	0.0	337.77	357.31	303.50	-30.92	-22.50	-1211.98028	-1213.31763	-1212.86682	-53.63	-52.44	*-33.17*	-34.35
**TS8**	-371.7	334.36	354.66	298.91	-30.97	-22.55	-1211.97634	-1213.31390	-1212.87047	-51.16	-50.10	*-35.46*	-36.51
**8**	0.0	338.97	358.18	305.84	-31.51	-22.92	-1212.02152	-1213.36454	-1212.91065	-79.51	-81.88	*-60.67*	-58.30
*low-lying isomers of (**C**)$_2$*													
2**C**	0.0	325.84	345.24	276.79	-35.31	-24.13	-1186.42023	-1187.72590	-1187.31723	0.00	0.00	*0.00*	0.00
**D**	0.0	329.10	348.09	295.56	-28.44	-20.83	-1186.51082	-1187.81861	-1187.37779	-56.84	-58.18	*-38.00*	-36.66
**TSD0**	-112.8	326.18	345.60	291.77	-29.47	-21.40	-1186.49475	-1187.80173	-1187.36786	-46.76	-47.58	*-31.77*	-30.94
**D0**	0.0	328.81	347.80	295.12	-29.76	-21.64	-1186.51187	-1187.82071	-1187.38189	-57.50	-59.50	*-40.57*	-38.58
**Da**	0.0	328.62	347.67	294.88	-30.10	-22.05	-1186.50260	-1187.81153	-1187.37374	-51.69	-53.74	*-35.46*	-33.41
*Reaction of **1** (B_2Ar_4) and **1R** ($BH_3{\cdot}SMe_2$) to form **2** (BAr_3)*													
**1** + **1R**	0.0	365.83	388.39	314.67	-38.76	-27.16	-1639.14140	-1640.79566	-1640.33147	0.00	0.00	*0.00*	0.00
**1**·**1R**	0.0	366.38	389.16	329.12	-31.68	-23.34	-1639.15430	-1640.80569	-1640.31538	-8.09	-6.29	*10.10*	8.30
**TSE**	-58.1	365.44	388.19	327.81	-34.05	-25.40	-1639.14302	-1640.79226	-1640.30732	-1.01	2.13	*15.15*	12.01
**E**	0.0	367.74	390.00	330.96	-36.96	-27.90	-1639.14628	-1640.79847	-1640.31251	-3.06	-1.77	*11.89*	10.60
**TSF**	-27.0	366.93	388.99	330.12	-35.26	-26.43	-1639.13885	-1640.79043	-1640.30346	1.60	3.28	*17.58*	15.90
**F** + $HBAr_2$	0.0	365.95	388.22	313.79	-37.94	-26.46	-1639.13760	-1640.79224	-1640.32834	2.39	2.14	*1.96*	2.21
**TSG**	-27.2	365.53	388.26	327.87	-31.00	-22.85	-1639.15828	-1640.80816	-1640.31906	-10.59	-7.84	*7.78*	5.04
**G**	0.0	367.89	390.02	331.53	-32.08	-23.87	-1639.17980	-1640.83478	-1640.34149	-24.09	-24.55	*-6.29*	-5.83
**TSH**	-30.4	364.32	387.11	326.45	-31.20	-22.71	-1639.14618	-1640.80010	-1640.31305	-2.99	-2.78	*11.56*	11.35
**TSHa**	-67.3	365.46	387.93	328.28	-32.53	-23.85	-1639.14372	-1640.79368	-1640.30554	-1.46	1.24	*16.27*	13.57
**H**	0.0	367.98	390.01	331.73	-31.95	-23.48	-1639.16495	-1640.81765	-1640.32342	-14.78	-13.80	*5.05*	4.07
**TSI**	-6.2	365.16	387.72	327.83	-32.54	-23.84	-1639.14786	-1640.79785	-1640.31040	-4.05	-1.37	*13.22*	10.54
**I** + **2**	0.0	366.35	388.38	314.63	-40.60	-28.79	-1639.12730	-1640.78261	-1640.32106	8.85	8.19	*6.53*	7.19
*…further trapping **I** with **1R** ($BH_3{\cdot}SMe_2$)*													

**I** + **1R**	0.0	212.02	225.23	170.35	-31.29	-21.35	-1305.59808	-1306.73249	-1306.48902	0.00	0.00	0.00	0.00
**I**·**1R**	0.0	212.13	225.98	183.25	-24.15	-16.83	-1305.62080	-1306.75042	-1306.48221	-14.26	-11.25	4.27	1.27
**TSJ**	-334.1	211.33	225.44	181.93	-24.80	-17.29	-1305.61294	-1306.74293	-1306.47755	-9.33	-6.55	7.19	4.42
**J** + SMe_2	0.0	212.67	226.03	171.71	-27.38	-18.34	-1305.62971	-1306.76629	-1306.51586	-19.85	-21.21	-16.84	-15.48
*…and trapping **I** with SMe_2: high-lying “HB” species HB·SMe_2 and **Ka** ArH_2B-$BH(SMe_2)_2$*													
**I** + SMe_2	0.0	191.74	203.91	151.37	-26.07	-17.42	-1278.92093	-1280.03220	-1279.81271	0.00	0.00	0.00	0.00
**TSK**	-52.2	189.47	203.05	160.06	-21.90	-15.08	-1278.93849	-1280.04793	-1279.81388	-11.02	-9.87	-0.73	-1.88
**K**	0.0	192.19	204.60	164.71	-22.67	-16.08	-1278.95574	-1280.06437	-1279.82450	-21.84	-20.19	-7.40	-9.06
**Ka**	0.0	191.31	203.99	163.57	-25.87	-18.85	-1278.93349	-1280.04095	-1279.80731	-7.88	-5.50	3.39	1.01
HB·SMe_2 + **7m**·SMe_2	0.0	190.03	202.79	148.84	-29.16	-19.56	-1278.83582	-1279.94022	-1279.72817	53.41	57.72	53.05	48.74
*facile and exergonic BH/BB dimerization of **I** (ArHBHBH·SMe_2)*													
2**I**	0.0	288.42	306.01	240.82	-39.40	-27.81	-1601.59712	-1603.06199	-1602.71652	0.00	0.00	0.00	0.00
**TSIdh**	-181.5	284.40	304.15	248.73	-31.09	-21.98	-1601.61745	-1603.07625	-1602.71188	-12.76	-8.95	2.91	-0.90
$(\mathbf{I})_{2h}$	0.0	288.89	307.49	255.19	-29.16	-20.53	-1601.66364	-1603.12586	-1602.74890	-41.74	-40.08	-20.32	-21.98
**TSId**	-297.9	286.67	305.40	252.86	-28.58	-20.26	-1601.63580	-1603.09577	-1602.72210	-24.27	-21.20	-3.50	-6.58
$(\mathbf{I})_2$	0.0	289.15	307.35	256.36	-27.96	-19.88	-1601.67594	-1603.13788	-1602.75801	-49.46	-47.62	-26.04	-27.88
**L** + 2SMe_2	0.0	287.83	305.79	230.00	-31.46	-20.04	-1601.63583	-1603.10303	-1602.75940	-24.29	-25.75	-26.91	-25.45
**M** + SMe_2	0.0	289.80	307.58	244.34	-30.55	-20.72	-1601.67536	-1603.14162	-1602.77923	-49.09	-49.97	-39.35	-38.48
*Competing formation of **P** (HArBBArH·SMe_2) instead of **I***													
*…SMe_2 release from **1R** (BH_3·SMe_2) to trap initially formed $HBAr_2$ (**9m**)*													
**1R** + $HBAr_2$	0.0	222.95	236.25	181.44	-27.66	-18.46	-1072.56585	-1073.59209	-1073.32634	0.00	0.00	0.00	0.00
**TSN**	-368.7	222.38	236.29	193.05	-21.95	-15.30	-1072.57071	-1073.59373	-1073.30746	-3.05	-1.03	11.85	9.83
**N** + SMe_2	0.0	223.33	236.82	182.40	-23.47	-15.12	-1072.57360	-1073.60169	-1073.32910	-4.86	-6.03	-1.73	-0.56
$HBAr_2$ + SMe_2	0.0	202.67	214.92	162.88	-22.43	-14.54	-1045.88871	-1046.89180	-1046.64938	0.00	0.00	0.00	0.00
**O**	0.0	203.44	215.72	176.04	-21.11	-15.06	-1045.91445	-1046.91616	-1046.65661	-16.15	-15.29	-4.54	-5.41
*…isomerization of remaining **F** (Ar_2BBH_2·SMe_2) nto **P** (HArBBArH·SMe_2) , followed by SMe_2 trapping*													
**F**	0.0	210.81	224.20	182.28	-21.87	-15.44	-1071.37126	-1072.40165	-1072.13276	0.00	0.00	0.00	0.00
**TSP**	-79.8	210.06	223.12	181.87	-24.05	-17.35	-1071.34348	-1072.37242	-1072.10722	17.43	18.34	16.03	15.12
**P**	0.0	211.45	224.60	183.09	-22.48	-15.94	-1071.36345	-1072.39361	-1072.12422	4.90	5.05	5.36	5.21

**P** + SMe_2	0.0	258.98	275.50	214.46	-28.84	-19.45	-1549.48583	-1550.89480	-1550.57801	0.00	0.00	*0.00*	0.00
**Q**	0.0	259.36	276.13	227.58	-23.08	-16.28	-1549.52430	-1550.93222	-1550.59249	-24.14	-23.48	*-9.08*	-9.75
*Trapping **P** with reactant **1R** ($BH_3{\cdot}SMe_2$) to form S via SMe_2 migration*												
**P** + **1R**	0.0	279.26	296.83	233.03	-34.07	-23.38	-1576.16297	-1577.59510	-1577.25498	0.00	0.00	*0.00*	0.00
**TSR**	-310.7	278.63	297.11	244.56	-27.18	-19.34	-1576.17183	-1577.60022	-1577.23829	-5.56	-3.21	*10.47*	8.12
**S** + SMe_2	0.0	280.58	297.79	235.58	-32.70	-22.61	-1576.18644	-1577.62248	-1577.27707	-14.73	-17.19	*-13.86*	-11.41
**R**	0.0	232.24	246.48	203.14	-24.34	-17.55	-1098.06417	-1099.12056	-1098.82181	0.00	0.00	*0.00*	0.00
**Ra** + SMe_2	0.0	231.70	245.72	190.04	-24.89	-16.31	-1098.03706	-1099.09316	-1098.81027	17.01	17.20	*7.24*	7.06
**S**	0.0	233.05	246.89	204.21	-26.33	-19.09	-1098.06407	-1099.12129	-1098.82327	0.06	-0.45	*-0.92*	-0.40
Dimerization of P via BH/BH addition												
2**P**	0.0	422.90	449.19	366.18	-44.95	-31.88	-2142.72691	-2144.78721	-2144.24844	0.00	0.00	*0.00*	0.00
**TSPdh**	-183.7	419.42	447.45	376.16	-34.15	-24.97	-2142.74939	-2144.80710	-2144.24444	-14.11	-12.48	*2.51*	0.88
(**P**)$_{2h}$	0.0	424.05	450.76	383.12	-33.10	-24.32	-2142.79056	-2144.85503	-2144.28024	-39.94	-42.56	*-19.96*	-17.34
*Highly exergonic **Ad** + **Q** coupling to form pentaborane(9) **8a5** ($Ar_5B_5H_4$)*												
**Ad** + **Q**	0.0	499.62	530.68	438.18	-47.68	-33.87	-2438.77140	-2441.16537	-2440.51503	0.00	0.00	*0.00*	0.00
**8a5** + 2SMe_2	0.0	501.83	531.91	432.03	-48.64	-33.62	-2438.82867	-2441.22870	-2440.58476	-35.94	-39.74	*-43.76*	-39.96
*Reaction of **Ad** and **1R** ($BH_3{\cdot}SMe_2$): formal H/Ar exchange*												
**Ad** + **1R**	0.0	308.06	326.78	260.54	-36.19	-25.03	-1394.04661	-1395.43464	-1395.05330	0.00	0.00	*0.00*	0.00
**Cd** + $ArBH_2.SMe_2$	0.0	308.18	326.69	259.74	-35.16	-24.36	-1394.04362	-1395.43369	-1395.05257	1.88	0.59	*0.45*	1.74
*Highly exergonic Cd + **Q** coupling to form **8** ($Ar_4B_5H_5$)*												
**Cd** + **Q**	0.0	431.71	458.75	374.56	-41.70	-29.23	-2168.20992	-2170.30603	-2169.74970	0.00	0.00	*0.00*	0.00
**8** + 2SMe_2	0.0	434.02	459.98	368.59	-44.23	-29.95	-2168.26626	-2170.36694	-2169.81824	-35.36	-38.22	*-43.01*	-40.15
*Trapping of **P** and **I** with less coordinating THF*												
**P** + THF	0.0	283.69	300.47	238.37	-29.81	-16.94	-1303.96729	-1305.23576	-1304.87988	0.00	0.00	0.00	0.00
**P**·THF	0.0	285.61	302.15	254.07	-24.86	-17.78	-1303.99391	-1305.26135	-1304.88180	-16.70	-16.06	-1.20	-1.84
**I**	0.0	216.45	228.88	175.69	-27.04	-14.91	-1033.40240	-1034.37315	-1034.11392	0.00	0.00	*0.00*	0.00
**I**·THF	0.0	217.85	230.34	190.31	-22.23	-15.81	-1033.43358	-1034.40192	-1034.12083	-19.56	-18.05	*-4.33*	-5.84
*Reaction of **1** (Ar_2BBAr_2) and H_2: major **9** ($(HBAr_2)_2$) and minor **2** (Ar_3B) and **7** ($(H_2BAr)_2$) formation*												
**1** + H_2	0.0	304.38	324.59	263.88	-27.88	-18.54	-1135.52248	-1136.77011	-1136.37311	0.00	0.00	*0.00*	0.00

**TS1Ha**	-481.6	307.88	326.25	274.40	-26.92	-19.57	-1135.50750	-1136.75375	-1136.34466	9.40	10.26	*17.86*	16.99
**1Ha**	0.0	312.13	329.71	279.92	-28.06	-20.59	-1135.55840	-1136.80559	-1136.38932	-22.54	-22.27	*-10.17*	-10.44
**TS1Hb**	-54.0	309.25	327.63	275.63	-26.76	-19.44	-1135.54579	-1136.79043	-1136.37915	-14.63	-12.75	*-3.79*	-5.67
2HBAr$_2$ (**9m**)	0.0	310.28	328.04	263.01	-32.14	-22.04	-1135.53268	-1136.78119	-1136.39116	-6.40	-6.95	*-11.32*	-10.76
**TS9**	-99.3	308.35	326.94	274.48	-26.90	-19.60	-1135.54017	-1136.78643	-1136.37724	-11.10	-10.24	*-2.59*	-3.45
**9**	0.0	311.98	329.89	279.53	-26.55	-19.30	-1135.57313	-1136.82114	-1136.40343	-31.78	-32.02	*-19.02*	-18.78
**TS1Hc**	-162.1	308.75	327.21	275.11	-27.53	-20.07	-1135.52986	-1136.77321	-1136.36377	-4.63	-1.95	*5.87*	3.18
**2** + H$_2$BAr (or **7m**)	0.0	309.41	327.57	262.38	-30.63	-21.00	-1135.53011	-1136.77717	-1136.38650	-4.79	-4.43	*-8.40*	-8.75
**2** + 0.5***7**	0.0	310.87	328.92	270.50	-29.68	-20.92	-1135.55356	-1136.80133	-1136.39907	-19.50	-19.59	*-16.29*	-16.20
*Reaction of **2** (Ar$_3$B) + HBcat: inefficient due to steric hindrance (Ar = o-tolyl)*													
**2** + HBcat	0.0	286.07	303.54	239.76	-30.83	-21.42	-1245.62620	-1246.98530	-1246.63133	0.00	0.00	*0.00*	0.00
**TS2Ha**	-126.2	285.95	303.42	253.24	-28.30	-20.85	-1245.60921	-1246.96568	-1246.59233	10.66	12.31	*24.47*	22.82
**2Ha**	0.0	287.69	304.95	255.49	-28.96	-21.39	-1245.60993	-1246.96703	-1246.59096	10.21	11.46	*25.33*	24.08
**TS2Hb**	-176.8	285.98	303.33	253.48	-28.90	-21.32	-1245.60739	-1246.96397	-1246.59099	11.80	13.39	*25.32*	23.73
**4** + HBAr$_2$ (or **9m**)	0.0	287.70	304.47	240.85	-32.84	-22.87	-1245.62834	-1246.98985	-1246.63645	-1.35	-2.86	*-3.22*	-1.71
**4** + 0.5***9**	0.0	288.55	305.40	249.11	-30.05	-21.50	-1245.64857	-1247.00982	-1246.64259	-14.04	-15.39	*-7.06*	-5.71
*...facile reaction of less bulky **2p** (Ph$_3$B) and HBcat to form **4p** Ph$_2$BBcat and **9p** (HBPh$_2$)$_2$*													
**2p** + HBcat	0.0	235.55	249.79	192.55	-29.90	-20.72	-1127.60187	-1128.83785	-1128.55800	0.00	0.00	*0.00*	0.00
**TS2p**	-63.9	234.57	249.15	204.29	-27.71	-20.39	-1127.58829	-1128.81993	-1128.52385	8.52	11.25	*21.43*	18.70
**4p** + 0.5***9p**	0.0	237.30	251.30	200.88	-29.09	-20.77	-1127.61875	-1128.85545	-1128.56391	-10.59	-11.04	*-3.71*	-3.26
*Dimerization of **Q** via SMe$_2$ release into **P** (HArBBArH·SMe$_2$): high barrier of (9.1 +21.2)=30.3 kcal/mol at **TSPds***													
**P** + **Q**	0.0	470.81	500.73	410.67	-45.55	-32.22	-2620.88775	-2623.32583	-2622.71671	0.00	0.00	*0.00*	0.00
**TSPds**	-79.0	469.78	501.01	423.96	-38.59	-28.59	-2620.88200	-2623.31601	-2622.68293	3.61	6.16	*21.20*	18.64
**P2a** + SMe$_2$	40.9	471.41	501.64	414.08	-45.14	-32.45	-2620.87773	-2623.31646	-2622.70228	6.29	5.88	*9.05*	9.46
**P2m** + 2SMe$_2$	0.0	472.23	501.73	402.69	-47.19	-32.35	-2620.88390	-2623.32686	-2622.72765	2.42	-0.65	*-6.86*	-3.80
**D** + 3SMe$_2$	0.0	471.68	500.79	389.68	-47.53	-31.37	-2620.87793	-2623.32221	-2622.73917	6.16	2.27	*-14.10*	-10.20
*Dimerization of **K** via SMe$_2$ release into **I** (HArBBH$_2$·SMe$_2$): moderate barrier of (7.4 +15.7)=23.1 kcal/mol at **TSIds***													
**I** + **K**	0.0	336.40	357.60	285.12	-42.38	-29.99	-2079.75431	-2081.59536	-2081.18276	0.00	0.00	*0.00*	0.00
**TSIds**	-151.8	333.56	356.68	295.09	-34.33	-24.86	-2079.75751	-2081.59136	-2081.15770	-2.01	2.51	*15.72*	11.20

**I2a**	0.0	336.61	358.28	287.53	-36.69	-25.60	-2079.78120	-2081.62281	-2081.19937	-16.87	-17.22	*-10.42*	-10.07
(**I**)$_2$ + SMe_2	0.0	336.68	358.25	287.73	-34.32	-23.39	-2079.79831	-2081.63908	-2081.21181	-27.62	-27.43	*-18.23*	-18.41

Table S3. DFT computed ^{11}B chemical shifts (δ_{Calc} in ppm) at the GIAO TPSS-D3/def2-QZVP // TPSS-D3/def2-TZVP + COSMO level, as well as some available experimental data (δ_{Exp}) for comparison. Tentative assignment for some experimentally observed ^{11}B signals are shown in red. See Table S4 for optimized structures along with atomic order numbers.

Name	atom	δ_{Calc}	δ_{Exp}	Name	atom	δ_{Calc}	δ_{Exp}
**1**	1	85.6	88.6				
**1**	2	87.7	88.6	BH_3·THF	2	0.8	-1.1
**1R**	1	-19.1	-20.3	**Cd**	1	23.1	
**2**	1	73.0	72.6	**Cd**	18	26.8	
**4**	11	37.0	32.6	**Cd**	33	39.0	
**7**	1	21.3	21.3	**D0**	1	13.9	
**7**	2	21.3	21.3	**D0**	2	12.7	
**7m**	1	70.9		**D0**	3	26.5	
**7m**·SMe_2	1	-5.6	-8.3	**D0**	5	6.9	
**7m**·THF	2	9.3	5	**G**	60	-16.9	
**8a5**	3 (BC)	-37.3		**G**	31	18.5	
**8a5**	6	-8.0		**G**	1	19.1	
**8a5**	1	-8.2		HBcat	1	32.8	
**8a5**	4	-5.9		$HOBAr_2$	1	52.2	45
**8a5**	2	-3.7		**J**	4	-24.3	-22.6
**8a**	3 (BC)	-39.4		**J**	3	1.4	2.3
**8a**	6	-15.7		**J**	1	1.9	
**8a**	4	-4.7		**Ka**	1	-25.6	
**8a**	2	-7.9		**Ka**	16	3.1	
**8a**	1 BH	-0.9		**K**	16	-14.7	
**8**	3 (BH)	-44.3	-46.8	**K**	1	-2.8	
**8**	2	-5.6	-4.6	**M**	2	-30.5	-30.5
**8**	4	-5.6	-4.6	**M**	1	-1.8	-2.6
**8**	6	-5.6	-4.6	**M**	6	4.1	4.9
**8**	1	-5.6	-4.6	**M**	21	4.1	4.9
**8H**	3 (BH)	-55.3	-53.1	**Mt**	2	-8.9	
**8H**	6	-13.5	-13.4	**Mt**	1	0.8	
**8H**	2	-13.5		**Mt**	21	4.4	
**8H**	4	-13.5		**Mt**	6	6.4	
**8H**	1	-13.5		**N**	1	41.2	
**9**	2	28.7	28.5	**N**	2	8.4	
**9**	1	28.7	28.5	**O**	1	-1.5	-0.3
**9m**	1	64.2		**Q**	17	-1.0	
**9m**·THF	2	9.9	8.9	**Q**	1	-1.1	
B_2H_6	1	21.4	16.6	**S**	4	-23.4	-22.6
B_2H_6	2	21.4	16.6	**S**	1	6.8	2.3

BH_3	1	87.9	86.0	**S**	3	6.8

Table S4. TPSS-D3/def2-TZVP + COSMO optimized Cartesian coordinates (in Å) in toluene solution. Each structure is labeled by the specific name (See also Figure S28-S30 and Table S2), followed by the number of atoms, the total energy (in hartrees), and the detailed atomic coordinates (in double-column text list).

1·1R : complex of B_2(*o*-tolyl)$_4$ and $BH_3·SMe_2$
71
Energy = -1639.077947417

B	0.7724758	0.2281957	0.3037948
B	-0.7235266	-0.0833095	-0.4812388
C	0.5239835	1.4161365	1.3563556
C	2.1537570	0.0623474	-0.4833857
C	-1.6224417	-1.3325204	-0.1469968
C	-1.3188503	1.0219170	-1.4429789
C	0.0844549	1.1602706	2.6684775
C	0.6340708	2.7747618	0.9591495
C	2.1325476	-0.4784356	-1.7828371
C	3.4258331	0.3821087	0.0619125
C	-2.0898222	-2.2467291	-1.1260101
C	-2.0020735	-1.5545784	1.1913581
C	-2.3585401	1.8706350	-0.9842281
C	-0.8210689	1.2407236	-2.7408215
C	-0.2573864	2.1774798	3.5587480
H	0.0163759	0.1285345	3.0069562
C	0.2798747	3.7889959	1.8568115
C	3.2842571	-0.6899986	-2.5410819
H	1.1733703	-0.7628558	-2.2075649
C	4.5814461	0.1577034	-0.6976143
C	-2.9070273	-3.3152031	-0.7405362
C	-2.8460682	-2.6016616	1.5596585
H	-1.6432176	-0.8744954	1.9580831
C	-2.8377829	2.8882341	-1.8171294
C	-1.3280275	2.2383683	-3.5731321
H	-0.0120299	0.6206090	-3.1151098
C	-0.1719167	3.5066924	3.1464781
H	-0.5916584	1.9317742	4.5638530
H	0.3648804	4.8251042	1.5335065
C	4.5228771	-0.3675660	-1.9903474
H	3.2154066	-1.1114810	-3.5405832
H	5.5500546	0.3984969	-0.2633762
H	-3.2417967	-4.0202977	-1.4988621
C	-3.3019088	-3.4915763	0.5872174
H	-3.1312997	-2.7298264	2.6005265
C	-2.3393432	3.0764327	-3.1063925
H	-3.6223911	3.5434506	-1.4429548
H	-0.9216565	2.3679971	-4.5730496
H	-0.4462443	4.3149127	3.8196140
H	5.4379663	-0.5306318	-2.5539467
H	-3.9482230	-4.3221485	0.8582547
H	-2.7339619	3.8705799	-3.7348388
B	1.2861981	-1.9544676	1.6052424
H	0.5612034	-2.4639598	2.4146303
H	0.7582603	-0.8567106	1.2954484
H	2.4379377	-1.8052875	1.8915929
S	1.1903879	-3.0017739	-0.0306914
C	2.8988571	-3.5692095	-0.2403704
H	3.4811723	-2.6772886	-0.4785040
H	3.2530501	-4.0273302	0.6840208
H	2.9293970	-4.2759868	-1.0710318
C	0.3643521	-4.5203260	0.5115921
H	-0.6618340	-4.2324862	0.7463380
H	0.3783241	-5.2326981	-0.3147114
H	0.8648763	-4.9247148	1.3923401
C	-2.9470896	1.7116584	0.3982153
H	-3.4739814	0.7552195	0.4933735
H	-2.1675487	1.7312424	1.1678898
H	-3.6570176	2.5164955	0.6108256
C	-1.6862981	-2.1119389	-2.5740625
H	-2.0455346	-1.1690437	-2.9991829
H	-0.5942430	-2.1187803	-2.6796846
H	-2.0867341	-2.9370886	-3.1700275
C	1.1645322	3.1615974	-0.4001607
H	0.6338176	2.6443654	-1.2033927
H	1.0668950	4.2399474	-0.5586478
H	2.2245569	2.8953870	-0.4959440
C	3.5791601	0.9501376	1.4509995
H	3.2027502	1.9778132	1.5046552
H	3.0066273	0.3707044	2.1807590
H	4.6322223	0.9535726	1.7491291

1a : first higher conformer of B_2(*o*-tolyl)$_4$
58
Energy = -1134.280177914

B	-0.0000001	0.0001174	0.8384621
B	0.0000522	0.0000967	-0.8399295

C	-0.8071308	-1.1288641	1.5610860
C	0.8071214	1.1290578	1.5611838
C	-1.1292317	0.8074851	-1.5619051
C	1.1292918	-0.8074121	-1.5618652
C	-2.0923733	-1.4344953	1.0656318
C	-0.3156412	-1.8834244	2.6589934
C	2.0923316	1.4348025	1.0657250
C	0.3156614	1.8834944	2.6591960
C	-1.8842731	0.3167984	-2.6597724
C	-1.4348160	2.0923257	-1.0654162
C	1.8843966	-0.3168418	-2.6597491
C	1.4346634	-2.0923205	-1.0653993
C	-2.9000562	-2.4047277	1.6558526
H	-2.4702631	-0.8750605	0.2123888
C	-1.1200059	-2.8823320	3.2159497
C	2.8999606	2.4051108	1.6558958
H	2.4702970	0.8752868	0.2125723
C	1.1199891	2.8824319	3.2161661
C	-2.8833278	1.1216157	-3.2157716
C	-2.4052998	2.9003363	-1.6545403
H	-0.8757016	2.4691271	-0.2114814
C	2.8833829	-1.1217799	-3.2157120
C	2.4051120	-2.9004257	-1.6544684
H	0.8752445	-2.4691940	-0.2116934
C	-2.4071222	-3.1371272	2.7354189
H	-3.8959193	-2.6000981	1.2670222
H	-0.7347547	-3.4663027	4.0494199
C	2.4070879	3.1373100	2.7356252
H	3.8956525	2.6008079	1.2667986
H	0.7346919	3.4664118	4.0496073
H	-3.4675810	0.7369761	-4.0493275
C	-3.1379247	2.4083217	-2.7342412
H	-2.6007695	3.8957685	-1.2646372
C	3.1378075	-2.4085231	-2.7341767
H	3.4677225	-0.7372018	-4.0492347
H	2.6005192	-3.8958419	-1.2644941
H	-3.0144182	-3.9109662	3.1976534
H	3.0144462	3.9109963	3.1980313
H	-3.9114835	3.0162238	-3.1961284
H	3.9112832	-3.0165320	-3.1960603
C	-1.0680077	1.6496595	3.2192569
H	-1.8395809	1.8062520	2.4561262
H	-1.1834205	0.6211365	3.5759467
H	-1.2678661	2.3305011	4.0515186
C	1.0681565	-1.6498625	3.2188526
H	1.8395862	-1.8065178	2.4555892
H	1.1837938	-0.6213878	3.5756149
H	1.2680382	-2.3308063	4.0510253
C	-1.6512898	-1.0666995	-3.2205648
H	-0.6203914	-1.1857940	-3.5689251
H	-1.8173800	-1.8388851	-2.4599849
H	-2.3262835	-1.2624480	-4.0585375
C	1.6514531	1.0666070	-3.2206836
H	0.6205555	1.1856919	-3.5690484
H	1.8175660	1.8388685	-2.4601849
H	2.3264488	1.2622525	-4.0586777

1b : second higher conformer of **1** B_2(*o*-tolyl)$_4$
58
Energy = -1134.282995910

B	0.8510003	0.0002486	0.0001585
B	-0.8511428	-0.0000539	0.0003177
C	1.6568636	0.5998667	-1.2105633
C	2.8578424	-0.0487797	-1.5829761
C	1.1872312	1.6406018	-2.0528612
C	1.6575285	-0.5994112	1.2103697
C	3.5571475	0.2814426	-2.7396367
H	3.2273585	-0.8549353	-0.9544332
C	1.9097224	1.9819606	-3.2037907
C	-0.0473575	2.4425924	-1.7308352
C	2.8588890	0.0489450	1.5820210
C	1.1882100	-1.6401109	2.0528874
C	-1.6571727	-0.6008463	-1.2096853
C	-1.6575123	0.6004370	1.2101916
C	3.0802773	1.3109637	-3.5540575
H	4.4633298	-0.2565911	-3.0058167
H	1.5424144	2.7847217	-3.8403470
H	-0.6372565	2.6398552	-2.6313051
H	0.2226542	3.4097033	-1.2885270
H	-0.6931586	1.9317524	-1.0090006
C	3.5591347	-0.2818179	2.7379421
H	3.2278488	0.8554298	0.9535739
C	1.9114889	-1.9818356	3.2032116
C	-0.0470788	-2.4414199	1.7318560
C	-1.1865181	-1.6404672	-2.0527753
C	-2.8596954	0.0457478	-1.5806490
C	-1.1872090	1.6400508	2.0535122
C	-2.8602919	-0.0460452	1.5804784
H	3.6159208	1.5898176	-4.4580432
C	3.0826678	-1.3114096	3.5525024
H	4.4657925	0.2557744	3.0033913
H	1.5442565	-2.7843383	3.8401381
H	-0.6932311	-1.9302078	1.0105921
H	-0.6364174	-2.6383544	2.6327606
H	0.2220668	-3.4086570	1.2892983
C	0.0473897	-2.4431948	-1.7299789

C	-1.9082924	-1.9811207	-3.2043648
C	-3.5586953	-0.2842006	-2.7375707
H	-3.2310101	0.8497640	-0.9503873
C	-1.9105350	1.9819659	3.2037490
C	0.0486825	2.4406728	1.7330352
C	-3.5609879	0.2853604	2.7359295
H	-3.2299155	-0.8517841	0.9514542
H	3.6192584	-1.5909570	4.4557143
H	0.6383111	-2.6398926	-2.6298943
H	-0.2235116	-3.4105980	-1.2888489
H	0.6925579	-1.9332382	-1.0069518
H	-1.5405616	-2.7834594	-3.8412068
C	-3.0790650	-1.3104360	-3.5545093
H	-4.4674719	0.2506358	-3.0013196
C	-3.0822418	1.3122181	3.5525932
H	-1.5434246	2.7846858	3.8404743
H	-0.2197414	3.4088209	1.2920275
H	0.6940333	1.9299133	1.0107530
H	0.6385799	2.6357361	2.6339842
H	-4.4704654	-0.2487586	2.9987115
H	-3.6118643	-1.5858923	-4.4612115
H	-3.6166327	1.5891295	4.4579183

1H2 : H/Ar-bridged H_2 adduct of B_2(*o*-tolyl)$_4$
60
Energy = -1135.500538721

B	-0.7808996	-0.8322582	0.9431817
C	-0.4382688	-2.3828241	0.7942127
C	-1.4637688	-0.0159681	-0.3750210
B	0.1170453	0.6025414	0.1364701
C	-1.0073121	-3.2106936	-0.1858183
C	0.3851460	-3.0027249	1.7698801
C	-2.6871102	0.6733017	-0.0747241
C	-1.3246230	-0.5572945	-1.6849441
C	1.3334179	0.2435133	-0.8429842
C	0.0654585	2.0940613	0.7155638
C	-0.7544133	-4.5820034	-0.2473885
H	-1.6800474	-2.7769200	-0.9204399
C	0.6440400	-4.3760681	1.6981067
C	-3.6553313	0.8054881	-1.0747126
C	-2.2950326	-0.4132878	-2.6610122
H	-0.4010387	-1.0720449	-1.9230873
C	2.2111283	-0.7936388	-0.4799006
C	1.6162659	0.9475056	-2.0408070
C	0.9400276	2.4834503	1.7592185
C	-0.7579535	3.0851947	0.1580198
C	0.0881897	-5.1693668	0.6941249
H	-1.2153172	-5.1850497	-1.0254121
H	1.2879057	-4.8300852	2.4487977
C	-3.4676567	0.2808051	-2.3513679
H	-4.5779226	1.3312144	-0.8440917
H	-2.1427633	-0.8260088	-3.6531563
C	3.3291133	-1.1357469	-1.2402712
H	2.0047782	-1.3637131	0.4201265
C	2.7357242	0.5932926	-2.8034737
C	0.9455581	3.8124516	2.2037829
C	-0.7473907	4.4057234	0.6019031
H	-1.4373114	2.8139729	-0.6454721
H	0.3019326	-6.2342556	0.6575819
H	-4.2375571	0.4143802	-3.1061174
H	3.9794558	-1.9452945	-0.9196945
C	3.5954934	-0.4346234	-2.4139998
H	2.9336448	1.1366511	-3.7252981
C	0.1112914	4.7745993	1.6367781
H	1.6215516	4.0932133	3.0089351
H	-1.4043710	5.1395349	0.1425117
H	4.4570522	-0.6866631	-3.0266293
H	0.1338938	5.7991715	1.9984362
H	-1.3821804	-0.5475606	1.9366996
H	0.3425311	-0.1855731	1.1771178
C	1.8690801	1.4930894	2.4185485
H	1.3088025	0.7614533	3.0127708
H	2.4485998	0.9331653	1.6758420
H	2.5678895	2.0012463	3.0888695
C	0.7313233	2.0608762	-2.5422007
H	0.8307158	2.9564735	-1.9194745
H	0.9921417	2.3265168	-3.5707789
H	-0.3252646	1.7741131	-2.5185629
C	-3.0189063	1.2134559	1.2891428
H	-2.1324326	1.5240649	1.8403924
H	-3.7037260	2.0615139	1.1999097
H	-3.5225042	0.4352617	1.8768069
C	0.9894675	-2.2014514	2.8971245
H	1.7594371	-1.5088726	2.5337923
H	0.2296181	-1.5927828	3.4012178
H	1.4576108	-2.8568355	3.6367445

1R : the reactant of $BH_3{\cdot}SMe_2$ adduct
13
Energy = -504.7874657820

B	0.0094109	1.7795862	0.3138524
H	0.0092462	1.5734828	1.5073440
H	-0.9997551	2.3038949	-0.1029226
H	1.0230176	2.2940042	-0.1044355
S	0.0002098	0.0250061	-0.5388830
C	1.3867642	-0.8375770	0.2444821

H	2.2981925	-0.3525361	-0.1082165
H	1.3024848	-0.7442587	1.3289410
H	1.3738340	-1.8834424	-0.0662901
C	-1.3954360	-0.8230364	0.2442007
H	-1.3105881	-0.7304045	1.3286706
H	-2.3016701	-0.3286639	-0.1089190
H	-1.3933497	-1.8690541	-0.0663543

1 : the lowest diborane(4) B_2(*o*-tolyl)$_4$
58
Energy = -1134.283690067

B	0.7580817	-0.4784603	0.1237415
B	-0.8263848	0.0802102	0.0425100
C	1.8830609	-0.0719076	-0.8927551
C	3.2028296	0.0459983	-0.4042169
C	1.6457573	0.2822481	-2.2489752
C	1.1738436	-1.2099366	1.4607413
C	4.2533646	0.4978612	-1.1980283
H	3.3953288	-0.2179377	0.6325682
C	2.7125398	0.7106296	-3.0458394
C	0.2697038	0.2035853	-2.8607556
C	0.8039338	-0.6012170	2.6810227
C	1.9255773	-2.4120752	1.5267144
C	-2.0858890	-0.6122153	-0.5658947
C	-0.9903918	1.5021752	0.7094532
C	4.0055560	0.8255160	-2.5314492
H	5.2548507	0.5881924	-0.7859136
H	2.5264387	0.9665855	-4.0866255
H	-0.1635366	-0.7967975	-2.7673063
H	0.3013615	0.4684092	-3.9215830
H	-0.4243797	0.8943521	-2.3652801
C	1.1750615	-1.1236585	3.9171325
H	0.2383376	0.3259701	2.6515324
C	2.2719512	-2.9402992	2.7782483
C	2.3377947	-3.1850776	0.2947332
C	-2.1199074	-1.9860517	-0.9228783
C	-3.2261333	0.1697049	-0.8490468
C	-1.8705619	1.7605917	1.7918640
C	-0.2110991	2.5672916	0.2161077
H	4.8136158	1.1701246	-3.1715871
C	1.9111909	-2.3084932	3.9668372
H	0.8883903	-0.6142133	4.8334942
H	2.8462088	-3.8642360	2.8167587
H	2.3011439	-2.5689186	-0.6052684
H	1.6757698	-4.0462954	0.1399757
H	3.3551784	-3.5746399	0.4059158
C	-0.9334012	-2.8790318	-0.6804165
C	-3.2717859	-2.5212730	-1.5081879
C	-4.3615346	-0.3640053	-1.4515174
H	-3.2048350	1.2268831	-0.5945760
C	-1.9509550	3.0562977	2.3138170
C	-2.7017964	0.6622637	2.4178294
C	-0.3304380	3.8626503	0.7185062
H	0.5010595	2.3732651	-0.5831510
H	2.2031012	-2.7370647	4.9220476
H	-1.0677225	-3.8555778	-1.1543406
H	-0.7570221	-3.0339195	0.3894550
H	-0.0188643	-2.4290636	-1.0905580
H	-3.2941241	-3.5767806	-1.7704755
C	-4.3854518	-1.7222197	-1.7744528
H	-5.2199238	0.2669604	-1.6657915
C	-1.2046901	4.1076756	1.7772464
H	-2.6113725	3.2444450	3.1581841
H	-3.0062852	0.9416005	3.4311227
H	-2.1495498	-0.2814672	2.4713366
H	-3.6090774	0.4690007	1.8342830
H	0.2697491	4.6677478	0.3027502
H	-5.2653013	-2.1592991	-2.2396477
H	-1.2951263	5.1065085	2.1960054

2Ha : BC/HB adduct of B(O-tolyl)$_3$ and HBcat
57
Energy = -1245.549109103

B	-0.8650394	0.0910880	0.1723140
C	0.1983916	-0.0005742	-1.1960733
C	-1.6439275	-1.2327368	0.6397245
C	0.3067282	-1.1924090	-1.9887013
C	0.4650583	1.2436720	-1.8391414
C	-1.2127711	-1.8822901	1.8116334
C	-2.8016564	-1.7423786	0.0046060
C	0.6331015	-1.0676951	-3.3438054
C	0.8135181	1.3365573	-3.1719980
H	0.4040870	2.1459204	-1.2401512
C	-1.8708289	-2.9911501	2.3419893
H	-0.3239535	-1.5197748	2.3228218
C	-3.4549883	-2.8600857	0.5388169
C	0.8844321	0.1674714	-3.9355946
H	0.7085692	-1.9710945	-3.9432376
H	1.0172000	2.3040037	-3.6198975
C	-3.0020712	-3.4888973	1.6982286
H	-1.4992621	-3.4616224	3.2486045
H	-4.3370589	-3.2429500	0.0293168
H	1.1464575	0.2165447	-4.9888025
H	-3.5279797	-4.3547800	2.0917206
B	0.9995217	0.0398325	0.3704331
H	-0.0160857	0.1693004	1.1846784

O	1.7316360	-1.1482386	0.7142526
O	1.8398933	1.1916049	0.5838137
C	3.0067144	-0.7152078	1.0052295
C	3.0688512	0.6809013	0.9272713
C	4.2413195	1.3728362	1.1792212
C	5.3694726	0.6063884	1.5208501
C	5.3060104	-0.7875247	1.5992017
C	4.1103622	-1.4803247	1.3392226
H	4.2838993	2.4554637	1.1179337
H	6.3081577	1.1120140	1.7270556
H	6.1954821	-1.3505760	1.8656575
H	4.0528826	-2.5624080	1.3981506
C	-1.6284721	1.4956509	0.3908455
C	-1.6390489	1.9350574	1.7336415
C	-2.3412105	2.2768297	-0.5481758
C	-2.3038722	3.0816851	2.1529286
H	-1.1044277	1.3487431	2.4794466
C	-3.0143395	3.4325171	-0.1135341
C	-3.0034410	3.8428034	1.2146645
H	-2.2763514	3.3778160	3.1982884
H	-3.5612368	4.0197433	-0.8492661
H	-3.5335372	4.7430977	1.5142733
C	-2.4347441	1.9759534	-2.0292824
H	-1.8919044	2.7327914	-2.6078841
H	-2.0292992	1.0028084	-2.2944382
H	-3.4806228	2.0114051	-2.3551173
C	-3.3500294	-1.1206291	-1.2524129
H	-3.7161383	-0.1074013	-1.0579561
H	-2.5801472	-1.0434979	-2.0287668
H	-4.1780794	-1.7127003	-1.6520186
C	0.1125499	-2.5814117	-1.4495702
H	0.6006050	-2.7069267	-0.4821338
H	-0.9503265	-2.7991238	-1.2988450
H	0.5136635	-3.3109901	-2.1581741

2 : monoborane(3) B(*o*-tolyl)$_3$
43
Energy = -838.2857309082

B	-0.0055302	-0.0019622	-0.0603859
C	-1.5467489	0.2722377	-0.0492592
C	0.5179328	-1.4714146	-0.2003264
C	0.9980256	1.1977824	-0.1502240
C	-2.0320607	1.2818286	-0.9048505
C	-2.4846631	-0.4595948	0.7232055
C	-0.1208221	-2.2829556	-1.1616142
C	1.6125985	-2.0182731	0.5151350
C	2.0540742	1.0905406	-1.0792092
C	0.8832087	2.3974501	0.5981634
C	-3.3935394	1.5495598	-1.0363144
H	-1.3168184	1.8610708	-1.4852281
C	-3.8459875	-0.1581625	0.6107056
C	-2.0480075	-1.5231011	1.7054482
C	0.3219690	-3.5711893	-1.4535234
H	-0.9772491	-1.8799495	-1.6982187
C	2.0334379	-3.3230556	0.2314383
C	2.2778952	-1.2635847	1.6416118
H	2.1621858	0.1642376	-1.6391470
C	2.9489764	2.1342493	-1.3060499
C	1.8091681	3.4251603	0.3874787
C	-0.1807406	2.5828726	1.6571183
C	-4.3053153	0.8258602	-0.2678236
H	-3.7382339	2.3223990	-1.7181313
H	-4.5607240	-0.7036099	1.2236426
H	-2.8886526	-1.8366326	2.3313187
H	-1.6558588	-2.4046395	1.1878907
H	-1.2504733	-1.1584062	2.3622931
C	1.4078235	-4.0938838	-0.7493808
H	-0.1786509	-4.1644959	-2.2140569
H	2.8683704	-3.7419285	0.7897036
H	2.3688783	-0.1986616	1.4144467
H	3.2726531	-1.6678536	1.8513615
H	1.6847918	-1.3511043	2.5616947
H	3.7408156	2.0289146	-2.0428280
C	2.8257329	3.3085408	-0.5628013
H	1.7363078	4.3327826	0.9833199
H	-1.1403456	2.8646584	1.2096768
H	0.1109456	3.3718471	2.3571085
H	-0.3518575	1.6631991	2.2246160
H	-5.3701858	1.0311108	-0.3414743
H	1.7627026	-5.1003054	-0.9559261
H	3.5254591	4.1272262	-0.7098635

4 : product (*o*-tolyl)Bcat
27
Energy = -677.8304404543

C	1.7546992	-0.1052183	0.0034648
C	2.4078454	1.1450772	0.0031326
C	2.5418601	-1.2856902	-0.0004789
C	3.7961101	1.2466073	-0.0001978
H	1.8069366	2.0506949	0.0040240
C	3.9364690	-1.1659978	-0.0053879
C	4.5635715	0.0807982	-0.0049173
H	4.2747989	2.2216292	0.0012504
H	4.5417055	-2.0695754	-0.0103760
H	5.6487440	0.1394282	-0.0077110
B	0.2126449	-0.0949031	0.0055420

O -0.5388566 1.0944286 0.0064589
O -0.6392626 -1.2091735 0.0033258
C -1.8642942 0.6975877 0.0022520
C -1.9254712 -0.6959941 0.0012234
C -3.1282106 -1.3813248 -0.0013584
C -4.2909034 -0.5957614 -0.0038723
C -4.2296723 0.8032642 -0.0026630
C -3.0032113 1.4849007 0.0006471
H -3.1675372 -2.4655201 -0.0013475
H -5.2593744 -1.0864360 -0.0077986
H -5.1517191 1.3765212 -0.0050385
H -2.9482435 2.5684449 0.0016756
C 1.9250475 -2.6632423 -0.0004711
H 1.2917663 -2.8151001 0.8799941
H 1.2854625 -2.8123604 -0.8768348
H 2.7043340 -3.4301094 -0.0044517

7m·SMe_2 : adduct of **7m** (*o*-tolyl)BH_2 and SMe_2
26
Energy = -775.3303508915
B -1.4745273 0.8430923 1.1857602
H -2.5713088 0.5081293 1.5770430
H -1.5072940 1.6052161 0.2431217
S -0.8141668 -0.8515239 0.3753369
C -1.9215741 -1.0813081 -1.0385758
H -1.9770049 -0.1501880 -1.6058201
H -1.5465339 -1.9001909 -1.6544358
H -2.9013794 -1.3359761 -0.6314657
C 0.7001799 -0.3273439 -0.4661930
H 0.4806897 0.5369969 -1.0962463
H 1.4103039 -0.0532674 0.3156960
H 1.0880554 -1.1607631 -1.0537710
C -0.4107084 1.2439060 2.3130439
C 0.5255213 2.2603085 2.0475532
C -0.3499576 0.6159210 3.5831074
C 1.4862057 2.6588905 2.9798585
H 0.4915738 2.7556150 1.0785731
C 0.6149263 1.0180499 4.5156661
C 1.5310685 2.0313924 4.2246350
H 2.1894331 3.4524219 2.7383786
H 0.6492034 0.5277526 5.4872175
H 2.2695645 2.3271317 4.9657747
C -1.3150633 -0.4870902 3.9508724
H -2.3531738 -0.1437009 3.8753257
H -1.2189173 -1.3449635 3.2743902
H -1.1371329 -0.8377026 4.9720857

7m·THF : adduct of **7m** (*o*-tolyl)BH_2 and THF
30
Energy = -529.8116664501
H 0.2708301 0.3011575 -1.7973403
B 0.1285692 0.1236573 -0.6000867
C -1.1830615 -0.7214254 -0.2410546
C -1.9779469 -1.2466769 -1.2752629
C -1.5786337 -1.0020526 1.0916630
C -3.1114418 -2.0259938 -1.0306651
H -1.6927409 -1.0358385 -2.3048407
C -2.7187938 -1.7787411 1.3350107
C -3.4853924 -2.2922449 0.2864878
H -3.6973375 -2.4194234 -1.8581314
H -3.0083584 -1.9891186 2.3634948
H -4.3644363 -2.8958077 0.4994180
H 1.1460264 -0.2615446 -0.0594774
C 1.1237646 2.4976586 -0.0106660
O -0.0609737 1.6008361 0.0209126
C -1.2631274 2.3563027 -0.3824574
C -0.9228987 3.7749988 0.0278234
C 0.5653844 3.8776362 -0.3516378
H 1.5641529 2.4342737 0.9854517
H 1.8108163 2.0971650 -0.7566773
H -1.3836024 2.2408114 -1.4644752
H -2.0996582 1.8977384 0.1428736
H -1.5415122 4.5070686 -0.4958007
H -1.0625736 3.9026607 1.1057800
H 0.6667478 4.0731444 -1.4230048
H 1.0870238 4.6659770 0.1950201
C -0.7907586 -0.4631825 2.2633213
H 0.2860064 -0.5774771 2.0973019
H -0.9737427 0.6092270 2.4050778
H -1.0646257 -0.9762215 3.1905248

7m : monoborane(3) (*o*-tolyl)BH_2
17
Energy = -297.1864430824
B -1.6097317 1.8321973 -0.0006173
C -0.4510471 0.8260633 0.0001516
H -2.7560110 1.4910130 -0.0015273
H -1.3588802 3.0043794 -0.0004566
C 0.8718577 1.3308049 0.0006045
C -0.6312461 -0.5894381 0.0000261
C 1.9841137 0.4948482 0.0001392
H 1.0110641 2.4095482 0.0016660
C 0.4937716 -1.4179525 -0.0004040
C -2.0046611 -1.2165819 0.0004850
C 1.7874532 -0.8876464 -0.0004535
H 2.9887732 0.9078129 0.0001349

H	0.3605384	-2.4973709	-0.0003239
H	-2.5811801	-0.9118179	-0.8793606
H	-1.9276787	-2.3074544	0.0001709
H	-2.5802426	-0.9123074	0.8811221
H	2.6421267	-1.5593176	-0.0008669

7 : BH/BH diborane(6) [(*o*-tolyl)BH_2]$_2$
34
Energy = -594.4196234947

B	-0.6850792	-0.4544353	0.3617306
B	0.6841447	0.4491365	-0.3578796
C	-2.1748443	0.0081088	0.1806868
H	-0.3977422	-1.4415666	0.9656763
H	0.0535811	0.5303356	0.8166319
H	-0.0522952	-0.5380538	-0.8112722
C	2.1752201	-0.0097463	-0.1785059
H	0.3943911	1.4356630	-0.9614978
C	-2.5139566	1.3735557	0.1838898
C	-3.2154885	-0.9416814	0.0287074
C	3.2142588	0.9423900	-0.0303654
C	2.5168499	-1.3745840	-0.1789792
C	-3.8325689	1.8061929	0.0469006
H	-1.7262147	2.1140431	0.3048839
C	-4.5352165	-0.4975743	-0.1147707
C	-2.9214973	-2.4234731	0.0000788
C	4.5349165	0.5009924	0.1129311
C	2.9178862	2.4238083	-0.0074483
C	3.8364073	-1.8044907	-0.0425859
H	1.7303951	-2.1167983	-0.2978624
C	-4.8489994	0.8627061	-0.1047982
H	-4.0642710	2.8677102	0.0597067
H	-5.3297615	-1.2309329	-0.2352586
H	-2.4248497	-2.7466187	0.9222320
H	-3.8433844	-3.0003135	-0.1152495
H	-2.2534423	-2.6818542	-0.8301601
C	4.8512595	-0.8587269	0.1054311
H	5.3282306	1.2360768	0.2309838
H	3.8389758	3.0026085	0.1043488
H	2.4195199	2.7424219	-0.9302517
H	2.2504963	2.6844250	0.8226087
H	4.0700016	-2.8656233	-0.0526039
H	-5.8823598	1.1816348	-0.2127260
H	5.8852972	-1.1754865	0.2132469

8a5 : pentaborane(9) (*o*-tolyl)$_5B_5H_4$
79
Energy = -1482.507162549

B	0.0061780	-1.3051041	-0.1863520
B	1.3285971	-0.0475658	-0.1929926
B	0.0629739	-0.0431692	0.9592610
B	-1.2436812	0.0115240	-0.1611867
H	-0.9079543	-0.9002829	-1.0918809
B	0.0729748	1.2629656	-0.1375226
H	0.9525691	-0.9811163	-1.0911844
H	-0.9215992	0.9679123	-1.0291923
H	0.9319672	0.8982361	-1.0857440
C	2.8902372	0.0637150	-0.0965747
C	3.4244493	1.3193406	0.2471136
C	3.7793923	-1.0152857	-0.2999956
H	2.7475338	2.1547836	0.4098792
C	4.7950050	1.5152102	0.4031514
C	5.1544356	-0.8069525	-0.1360443
C	3.2796410	-2.3864475	-0.6808109
H	5.1785454	2.4951850	0.6740896
C	5.6668603	0.4423008	0.2143922
H	5.8337685	-1.6433622	-0.2861126
H	2.5811546	-2.7841462	0.0637033
H	4.1102440	-3.0905095	-0.7789956
H	2.7438671	-2.3641094	-1.6378059
H	6.7383742	0.5754148	0.3374655
C	-2.7846729	-0.0812530	0.1179072
C	-3.2914169	-1.3243624	0.5401216
C	-3.6793537	1.0074873	0.0041244
H	-2.6099728	-2.1656793	0.6378859
C	-4.6379550	-1.5016379	0.8491837
C	-5.0304492	0.8174292	0.3220348
C	-3.2116418	2.3716671	-0.4393082
H	-4.9992755	-2.4733278	1.1751877
C	-5.5143970	-0.4212455	0.7424300
H	-5.7132735	1.6600462	0.2359006
H	-2.4904936	2.7981086	0.2665484
H	-4.0548660	3.0624203	-0.5243444
H	-2.7127888	2.3272953	-1.4153026
H	-6.5673849	-0.5403176	0.9835278
C	-0.0941940	-2.8605361	0.0022551
C	-0.1235320	-3.3904635	1.3027923
C	-0.1769989	-3.7482252	-1.0953230
H	-0.0876627	-2.7083861	2.1477364
C	-0.2123344	-4.7635872	1.5289186
C	-0.2624675	-5.1243505	-0.8579250
C	-0.1887694	-3.2294905	-2.5123460
H	-0.2325402	-5.1500430	2.5443534
C	-0.2759197	-5.6344877	0.4414060
H	-0.3219736	-5.8057403	-1.7038206
H	-0.2163719	-4.0518627	-3.2319886
H	0.6982131	-2.6213517	-2.7283427

H	-1.0643700	-2.5931701	-2.6941749
H	-0.3413184	-6.7075629	0.6010486
C	0.1634844	2.8122795	0.0997380
C	0.1570346	3.3094433	1.4133897
C	0.2714382	3.7272387	-0.9731706
H	0.0832502	2.6079229	2.2391848
C	0.2431335	4.6765469	1.6747518
C	0.3554935	5.0968025	-0.7000047
C	0.3054715	3.2444999	-2.4026282
H	0.2345171	5.0382095	2.6995376
C	0.3401880	5.5741609	0.6115913
H	0.4351260	5.7987619	-1.5272639
H	0.3354858	4.0851384	-3.1007956
H	-0.5745647	2.6355300	-2.6442254
H	1.1887044	2.6201835	-2.5902400
H	0.4069055	6.6425564	0.7997552
C	-0.0700355	-0.0459866	2.5337934
C	-1.2536864	0.4661954	3.0988341
C	0.9168221	-0.5424942	3.4187020
C	-1.4722410	0.4990508	4.4749034
H	-2.0269850	0.8460457	2.4369027
C	0.6847869	-0.5152493	4.8009289
C	-0.4942496	0.0022292	5.3362585
H	-2.4004272	0.9045567	4.8697855
H	1.4490117	-0.9098729	5.4680431
H	-0.6464120	0.0140331	6.4126239
C	2.2262094	-1.0957178	2.9155780
H	2.0745140	-1.7718129	2.0679382
H	2.8862075	-0.2954065	2.5648674
H	2.7459265	-1.6456092	3.7060978

8a : (o-tolyl)$_4$B$_5$H$_5$ with apical B–C bond
66
Energy = -1211.958134428

B	0.0130865	-1.1980099	-0.1924819
B	1.3374668	0.0432554	-0.1384979
B	0.0307810	0.0749288	0.9610718
B	-1.2324623	0.1321109	-0.1777451
H	-0.9113612	-0.8288256	-1.0907026
B	0.0947199	1.3492208	-0.1662649
H	0.9723500	-0.8383897	-1.0755170
H	-0.8240966	1.0553911	-1.0870952
H	1.0559071	1.0247894	-1.0346274
C	2.8792415	-0.0359156	0.1459956
C	3.3438303	0.2560896	1.4393777
C	3.8167635	-0.4142199	-0.8433789
H	2.6233050	0.5194011	2.2087234
C	4.6995707	0.1905359	1.7597361
C	5.1744675	-0.4812014	-0.5103378
C	3.3701815	-0.7638685	-2.2425064
H	5.0347524	0.4209670	2.7674410
C	5.6189957	-0.1796090	0.7782047
H	5.8934417	-0.7728153	-1.2728819
H	2.7681032	-1.6823684	-2.2479086
H	4.2290216	-0.9258352	-2.8993391
H	2.7526324	0.0309190	-2.6778315
H	6.6787110	-0.2367742	1.0127029
C	-2.7848526	0.2321036	0.0215285
C	-3.2983116	0.2431691	1.3290037
C	-3.6862886	0.3085565	-1.0648093
H	-2.6058210	0.1831218	2.1643168
C	-4.6697046	0.3192236	1.5715631
C	-5.0601863	0.3838627	-0.8104617
C	-3.1853901	0.3084287	-2.4885858
H	-5.0450200	0.3231374	2.5914645
C	-5.5542485	0.3878266	0.4951795
H	-5.7524937	0.4392608	-1.6477939
H	-2.5487088	1.1796188	-2.6877857
H	-4.0168731	0.3300672	-3.1980103
H	-2.5830478	-0.5834485	-2.7021597
H	-6.6257569	0.4445400	0.6683684
C	0.1038672	-2.7537675	-0.0047198
C	1.3699326	-3.2880262	0.2998284
C	-0.9975783	-3.6352052	-0.0871389
H	2.2216479	-2.6161404	0.3788139
C	1.5574146	-4.6498926	0.5238972
C	-0.7965386	-5.0025819	0.1417239
C	-2.3848256	-3.1334672	-0.3999158
H	2.5464553	-5.0327405	0.7606601
C	0.4649039	-5.5142834	0.4455783
H	-1.6489849	-5.6757268	0.0799152
H	-3.0932117	-3.9636087	-0.4668783
H	-2.4097214	-2.5913312	-1.3530046
H	-2.7416264	-2.4391720	0.3684456
H	0.5921676	-6.5794994	0.6192287
C	-0.0089950	-0.0500713	2.5348161
C	-0.2191230	-1.3207539	3.1018342
C	0.1454401	1.0457660	3.4182930
C	-0.2741525	-1.5225636	4.4800765
H	-0.3401945	-2.1745720	2.4412109
C	0.0922357	0.8332990	4.8024536
C	-0.1157560	-0.4365598	5.3403757
H	-0.4384737	-2.5208871	4.8783100
H	0.2150545	1.6851419	5.4688269
H	-0.1539286	-0.5734589	6.4181838
C	0.3590127	2.4495014	2.9076405

H	1.2261202	2.5079090	2.2397499
H	-0.5052476	2.7971079	2.3298961
H	0.5176063	3.1432458	3.7386263
H	0.1133648	2.5238287	-0.0376739

8H : pentaborane(9) B_5H_9
14
Energy = -129.7761756316

B	0.0499513	-1.2671779	-0.1388380
B	1.2672133	0.0499291	-0.1387655
B	-0.0000153	-0.0000088	0.9890434
B	-1.2671850	-0.0498874	-0.1388502
H	-0.9140548	-0.9897260	-1.0332122
B	-0.0499296	1.2672166	-0.1387800
H	0.9892195	-0.9135148	-1.0336764
H	-0.0000550	-0.0000418	2.1735607
H	-0.9890787	0.9135414	-1.0337405
H	0.9138449	0.9894930	-1.0333191
H	2.4420073	0.0974422	-0.0027537
H	0.0976374	-2.4419574	-0.0027645
H	-2.4419820	-0.0973244	-0.0028299
H	-0.0975701	2.4420138	-0.0028166

8 : (*o*-tolyl)$_4B_5H_5$ with apical B–H bond
66
Energy = -1211.958769629

B	-0.0313818	-1.2857618	-0.2081721
B	1.2857151	-0.0313803	-0.2082541
B	0.0000040	0.0000043	0.8964088
B	-1.2857126	0.0313835	-0.2082477
H	-0.9338677	-0.9386944	-1.1166524
B	0.0313842	1.2857649	-0.2081785
H	0.9391973	-0.9345281	-1.1162729
H	0.0000054	0.0000078	2.0801995
H	-0.9392178	0.9345463	-1.1162604
H	0.9338813	0.9387089	-1.1166515
C	2.8298423	0.0121039	0.0520528
C	3.3032460	0.9457912	0.9914632
C	3.7633848	-0.8217460	-0.6053337
H	2.5866816	1.5841476	1.5038697
C	4.6608679	1.0697113	1.2814963
C	5.1234110	-0.6977269	-0.2956688
C	3.3260686	-1.8423488	-1.6295835
H	5.0003077	1.8026035	2.0083954
C	5.5760057	0.2385073	0.6348064
H	5.8397578	-1.3437719	-0.7986369
H	2.6566847	-2.5908511	-1.1887470
H	4.1897367	-2.3663819	-2.0476898
H	2.7859280	-1.3691609	-2.4592314
H	6.6378981	0.3165054	0.8527520
C	-2.8298386	-0.0121013	0.0520664
C	-3.3032406	-0.9458104	0.9914560
C	-3.7633844	0.8217494	-0.6053146
H	-2.5866707	-1.5841442	1.5038829
C	-4.6608677	-1.0697800	1.2814434
C	-5.1234167	0.6976791	-0.2956967
C	-3.3260617	1.8424296	-1.6294847
H	-5.0003085	-1.8027078	2.0083057
C	-5.5760111	-0.2385897	0.6347439
H	-5.8397685	1.3437096	-0.7986764
H	-2.6567197	2.5909267	-1.1885754
H	-4.1897306	2.3664602	-2.0475924
H	-2.7858729	1.3693106	-2.4591405
H	-6.6379075	-0.3166226	0.8526580
C	0.0119798	-2.8298336	0.0524669
C	0.9443425	-3.3029022	0.9933675
C	-0.8220324	-3.7634543	-0.6045976
H	1.5844857	-2.5864911	1.5037612
C	1.0649247	-4.6600140	1.2871632
C	-0.7016377	-5.1228766	-0.2908926
C	-1.8379140	-3.3270228	-1.6339009
H	1.7962280	-4.9991344	2.0158117
C	0.2322465	-5.5750088	0.6421614
H	-1.3488358	-5.8391072	-0.7925353
H	-2.3623090	-4.1908159	-2.0513059
H	-1.3604936	-2.7904658	-2.4634502
H	-2.5865436	-2.6546462	-1.1978605
H	0.3073005	-6.6364316	0.8634058
C	-0.0119758	2.8298384	0.0524508
C	-0.9443322	3.3029137	0.9933543
C	0.8220287	3.7634548	-0.6046295
H	-1.5844690	2.5865058	1.5037608
C	-1.0649168	4.6600283	1.2871361
C	0.7016315	5.1228801	-0.2909387
C	1.8379023	3.3270156	-1.6339375
H	-1.7962150	4.9991540	2.0157874
C	-0.2322475	5.5750191	0.6421172
H	1.3488232	5.8391074	-0.7925942
H	2.3622901	4.1908059	-2.0513572
H	1.3604760	2.7904474	-2.4634761
H	2.5865385	2.6546467	-1.1978966
H	-0.3073044	6.6364443	0.8633496

9m·THF : THF adduct of (*o*-tolyl)$_2$BH
43
Energy = -800.3548008079

H	-0.2770323	-0.1927990	-2.0737138
B	-0.0341135	-0.1635144	-0.8841353
C	-1.2380857	-0.7837941	-0.0186545
C	-1.6848053	-2.1024015	-0.2912387
C	-1.8868339	-0.0942375	1.0189561
C	-2.7259575	-2.6640710	0.4573101
C	-2.9267080	-0.6551586	1.7656038
H	-1.5794292	0.9204256	1.2625685
C	-3.3520126	-1.9518174	1.4829142
H	-3.0521336	-3.6780173	0.2309689
H	-3.3987629	-0.0827483	2.5607801
H	-4.1617643	-2.4049498	2.0493239
C	1.4382686	-0.7176024	-0.5360588
C	1.6433005	-1.5640965	0.5661217
C	2.5709443	-0.3757195	-1.3163960
C	2.9008592	-2.0701928	0.9013532
H	0.7836860	-1.8354391	1.1765617
C	3.8321968	-0.8825718	-0.9772074
C	4.0056015	-1.7239220	0.1237597
H	3.0174106	-2.7293090	1.7585917
H	4.6925563	-0.6174193	-1.5897914
H	4.9944132	-2.1047763	0.3678546
C	0.7372248	2.0300029	0.5444870
O	0.0544796	1.4314427	-0.6246774
C	-0.9489270	2.3511949	-1.1871160
C	-0.5426596	3.6985473	-0.6154977
C	-0.0202367	3.3283504	0.7846604
H	0.6840584	1.3073496	1.3580227
H	1.7772124	2.1848740	0.2495512
H	-0.8704639	2.2614780	-2.2703876
H	-1.9337575	2.0194990	-0.8470920
H	0.2546834	4.1436646	-1.2183313
H	-1.3851371	4.3921576	-0.5800945
H	0.6286869	4.0965114	1.2102134
H	-0.8578605	3.1621431	1.4690492
C	-1.0380306	-2.9181847	-1.3861548
H	-1.0390166	-2.3711059	-2.3362164
H	0.0121491	-3.1278764	-1.1503003
H	-1.5587123	-3.8703631	-1.5269207
C	2.4488194	0.5386842	-2.5134458
H	1.6427748	0.2092155	-3.1776651
H	2.2039855	1.5638186	-2.2100463
H	3.3854098	0.5687321	-3.0785819

9m : monoborane(3) (*o*-tolyl)$_2$BH
30
Energy = -567.7378265756

B	-0.0832100	0.4207963	0.7794533
C	1.3510408	0.1043472	0.2798675
C	-1.3625217	0.0652311	-0.0229095
H	-0.2091792	0.9685914	1.8388786
C	2.4424400	-0.0934788	1.1723852
C	1.6203025	0.0882645	-1.1073407
C	-2.5731643	0.8028343	0.1075242
C	-1.3696088	-1.0746831	-0.8581879
C	2.2416271	-0.1076607	2.6690936
C	3.7232051	-0.3085260	0.6547774
C	2.9045168	-0.1045834	-1.6115006
H	0.7995025	0.2565089	-1.7998886
C	-2.6497976	2.0424730	0.9665731
C	-3.7082661	0.3907642	-0.5969805
C	-2.5130800	-1.4895563	-1.5370675
H	-0.4571263	-1.6579146	-0.9524532
H	1.7674185	0.8183723	3.0124495
H	3.1986223	-0.2215093	3.1860707
H	1.5883405	-0.9322452	2.9759980
H	4.5520538	-0.4724459	1.3400440
C	3.9599946	-0.3103897	-0.7223272
H	3.0815388	-0.0972336	-2.6834808
H	-3.6704466	2.4347405	0.9886205
H	-1.9900405	2.8312656	0.5876220
H	-2.3345472	1.8308637	1.9942231
H	-4.6275266	0.9652134	-0.5051763
C	-3.6868409	-0.7458890	-1.4093597
H	-2.4906062	-2.3795791	-2.1598809
H	4.9680424	-0.4695317	-1.0965120
H	-4.5871183	-1.0488402	-1.9377438

9 : BH/BH diborane(6) [(*o*-tolyl)$_2$BH]$_2$
60
Energy = -1135.513916830

B	-0.1024521	-0.9050175	-0.1576373
B	0.1025575	0.9051023	-0.1577834
H	-0.0000847	0.0001405	0.8148604
H	-0.0001224	-0.0000422	-1.1223008
C	1.1917392	-1.8010917	-0.2728100
C	-1.5505797	-1.5376306	-0.1823599
C	1.4318241	-2.8853049	0.6060130
C	2.1308359	-1.5440039	-1.2863567
C	-2.5601186	-1.3275156	0.7843803
C	-1.8329457	-2.3819847	-1.2714676
C	-1.1917047	1.8010802	-0.2728745
C	1.5506357	1.5377520	-0.1827872
C	0.4838070	-3.1955193	1.7393372
C	2.5849083	-3.6600879	0.4409248
C	3.2729097	-2.3271700	-1.4489939

H	1.9658303	-0.7060565	-1.9594908
C	-2.3335275	-0.4658761	2.0026169
C	-3.8044063	-1.9480056	0.6170854
C	-3.0796061	-2.9827891	-1.4367172
H	-1.0530337	-2.5680220	-2.0077169
C	-1.4318404	2.8851841	0.6060633
C	-2.1306168	1.5442650	-1.2866560
C	2.5602757	1.3278854	0.7838957
C	1.8329377	2.3816915	-1.2722310
H	0.3846601	-2.3379286	2.4167201
H	0.8415170	-4.0460043	2.3262438
H	-0.5199605	-3.4246741	1.3660654
H	2.7690948	-4.4855234	1.1255022
C	3.5008023	-3.3919199	-0.5783175
H	3.9802293	-2.1039962	-2.2431799
H	-1.4042013	-0.7303815	2.5197962
H	-3.1566143	-0.5783651	2.7137956
H	-2.2681783	0.5925426	1.7256298
H	-4.5755189	-1.7905939	1.3684886
C	-4.0738812	-2.7613621	-0.4835876
H	-3.2699861	-3.6225199	-2.2943940
C	-0.4856492	3.1931666	1.7415181
C	-2.5840298	3.6610759	0.4399154
C	-3.2718138	2.3284965	-1.4503067
H	-1.9664988	0.7052703	-1.9587124
C	2.3334894	0.4673368	2.0028706
C	3.8047267	1.9478707	0.6159991
C	3.0797262	2.9820611	-1.4380127
H	1.0528787	2.5676935	-2.0083374
H	4.3877931	-4.0106394	-0.6870947
H	-5.0504297	-3.2268162	-0.5889040
H	-0.3890282	2.3349014	2.4184154
H	-0.8433332	4.0436823	2.3283950
H	0.5191603	3.4212117	1.3703884
H	-2.7669881	4.4879944	1.1230294
C	-3.4999508	3.3929235	-0.5792979
H	-3.9778464	2.1070126	-2.2461077
H	2.2676189	-0.5912792	1.7267551
H	1.4043714	0.7326908	2.5199932
H	3.1567386	0.5800423	2.7138307
H	4.5760496	1.7903603	1.3671701
C	4.0741802	2.7606604	-0.4850877
H	3.2700673	3.6214413	-2.2959593
H	-4.3875667	4.0108780	-0.6872976
H	5.0508818	3.2256784	-0.5908950

A0 : BH/BB adduct of HBcat and $B_2(o\text{-tolyl})_4$
72
Energy = -1541.561920867

B	1.0600918	0.8914491	-0.3165329
C	1.6693852	0.9891886	-1.7935193
C	2.0794974	1.2114526	0.8875203
B	-0.0342081	-0.6979066	-0.0896667
H	0.2861368	1.9693347	-0.3179739
C	2.7117114	0.1120668	-2.1417132
C	1.2608990	1.9362644	-2.7650531
C	2.1434946	2.5397810	1.3542023
C	2.9825423	0.2800487	1.4419852
C	0.0434230	-1.5593016	-1.3945661
C	-0.6658047	-1.2508622	1.2325965
B	-0.6048395	1.1481957	0.1748272
C	3.3092643	0.1185258	-3.3985492
H	3.0502491	-0.6192770	-1.4163051
C	1.8718719	1.9388332	-4.0271834
C	0.1698048	2.9524269	-2.5101204
C	3.0581004	2.9465706	2.3215431
H	1.4542074	3.2778158	0.9484220
C	3.9054480	0.6969259	2.4130548
C	2.9683360	-1.1774169	1.0575690
C	0.4712674	-2.9113303	-1.4193759
C	-0.3675127	-0.9802073	-2.6176039
C	-1.8376866	-2.0576909	1.2298884
C	-0.0879409	-0.9274078	2.4800892
O	-0.9713237	1.6132947	1.4667588
O	-1.7831032	0.9177902	-0.6109739
C	2.8813716	1.0369716	-4.3564414
H	4.0991034	-0.5917280	-3.6289245
H	1.5421369	2.6672945	-4.7653673
H	0.0618988	3.6163585	-3.3722492
H	0.3890986	3.5804433	-1.6372542
H	-0.8025772	2.4763671	-2.3335568
C	3.9518373	2.0160684	2.8553458
H	3.0729262	3.9805218	2.6564318
H	4.5914721	-0.0354912	2.8345888
H	2.1880000	-1.3861850	0.3191754
H	2.7701280	-1.8131121	1.9279585
H	3.9271917	-1.4928836	0.6298886
C	0.9571045	-3.6655689	-0.2020097
C	0.4830069	-3.6048491	-2.6395050
C	-0.3842167	-1.6882982	-3.8091846
H	-0.7099047	0.0472783	-2.6199034
C	-2.3522506	-2.5136136	2.4498836
C	-2.5726471	-2.4456426	-0.0315368
C	-0.5866156	-1.4230966	3.6777208
H	0.7718125	-0.2664967	2.4994614
C	-2.3340046	1.4391828	1.5331989

C	-2.8290707	1.0354131	0.2901879
H	3.3310083	1.0571829	-5.3458102
H	4.6731927	2.3139613	3.6117047
H	2.0497668	-3.7639703	-0.2214369
H	0.5439757	-4.6799059	-0.1975735
H	0.6815556	-3.1767204	0.7320692
H	0.8258217	-4.6377040	-2.6487263
C	0.0585459	-3.0137139	-3.8242904
H	-0.7244518	-1.2087661	-4.7227869
C	-1.7324601	-2.2211165	3.6623502
H	-3.2621798	-3.1095846	2.4435560
H	-3.6114983	-2.6942670	0.2047076
H	-2.1125768	-3.3245230	-0.4979656
H	-2.5620163	-1.6491894	-0.7776229
H	-0.1030847	-1.1709648	4.6175550
C	-3.1662712	1.6084672	2.6264914
C	-4.1721495	0.7851519	0.0717497
H	0.0738886	-3.5798027	-4.7520973
H	-2.1520691	-2.5983889	4.5913130
C	-4.5301625	1.3551317	2.4211466
H	-2.7772818	1.9097033	3.5930115
C	-5.0233228	0.9550787	1.1734034
H	-4.5422426	0.4657045	-0.8964428
H	-5.2184708	1.4684566	3.2532780
H	-6.0857576	0.7660614	1.0545008

(**A**)$_{2h}$: BB/BB dimer of H(*o*-tolyl)BB(*o*-tolyl)$_2$
90
Energy = -1727.525611013

B	0.1636208	-0.4470576	-0.5916640
B	1.5007226	0.4322759	0.3449103
B	-1.3294004	0.4574391	-0.2294804
H	-0.4911570	0.4157436	-1.3136373
B	-0.0685057	-0.0738450	1.0224326
H	0.6877393	0.9413423	1.3248810
C	2.8016884	-0.2116780	1.0002758
C	2.9180905	-0.3889648	2.3902618
C	3.8851792	-0.6553591	0.1960569
H	2.1138797	-0.0503696	3.0326157
C	4.0243096	-0.9911761	2.9860225
C	4.9880239	-1.2703113	0.8039926
H	4.0643278	-1.1067831	4.0661830
C	5.0701928	-1.4446974	2.1846007
H	5.8003605	-1.6216823	0.1705355
H	5.9399823	-1.9252603	2.6249702
C	-2.7045641	-0.2667044	-0.5775381
C	-2.8719150	-1.0040280	-1.7630811
C	-3.8077315	-0.2138446	0.3156862
H	-2.0534342	-1.0533048	-2.4712464
C	-4.0475189	-1.6860095	-2.0701004
C	-4.9814668	-0.9129210	0.0030675
H	-4.1252780	-2.2441461	-2.9997008
C	-5.1141665	-1.6474999	-1.1742521
H	-5.8097097	-0.8773413	0.7082821
H	-6.0385626	-2.1780890	-1.3872989
C	0.4920562	-1.6743091	-1.4794244
C	1.1522745	-2.8529261	-1.0349447
C	0.2792375	-1.5289179	-2.8739827
C	1.5324484	-3.8172340	-1.9763507
C	0.6820927	-2.4831692	-3.7973290
H	2.0254705	-4.7219406	-1.6268769
C	1.3081033	-3.6457224	-3.3403950
H	0.5116955	-2.3270473	-4.8585257
H	1.6246704	-4.4112977	-4.0434915
C	-0.5022212	-0.7678591	2.3385136
C	-1.2918777	-1.9479579	2.4195836
C	-0.2463835	-0.0666350	3.5444195
C	-1.7536244	-2.3744319	3.6708396
C	-0.7291649	-0.4899396	4.7745026
H	-2.3472784	-3.2845129	3.7257146
C	-1.4846830	-1.6636249	4.8381603
H	-0.5216367	0.0838559	5.6729508
H	-1.8667978	-2.0194018	5.7910897
H	0.3247843	0.8583175	3.4990036
H	-0.1907908	-0.6175329	-3.2376929
C	-3.7744039	0.5544750	1.6149960
H	-2.8673865	0.3446252	2.1883896
H	-3.8001386	1.6347993	1.4362599
H	-4.6373459	0.2891891	2.2330631
C	3.9015407	-0.5140003	-1.3070731
H	2.9650008	-0.8544320	-1.7574129
H	4.0413526	0.5307893	-1.6045792
H	4.7186780	-1.1036123	-1.7333529
C	1.4824743	-3.1246573	0.4109137
H	2.5674626	-3.1937562	0.5428212
H	1.1153846	-2.3480574	1.0797165
H	1.0514179	-4.0811893	0.7267570
C	-1.6751548	-2.7744085	1.2183020
H	-2.7646970	-2.8012966	1.1112218
H	-1.2589921	-2.3858694	0.2903834
H	-1.3313783	-3.8075900	1.3413000
C	1.7191249	1.7461237	-0.5714607
C	2.0147505	2.9664256	0.0901765
C	1.6871814	1.7538433	-1.9729383
C	2.2074471	4.1277931	-0.6671753
C	1.8994109	2.9124298	-2.7181132

H	1.5023898	0.8236515	-2.5037458
C	2.1460512	4.1144528	-2.0594305
H	2.4126604	5.0606012	-0.1460753
H	1.8611775	2.8745666	-3.8036510
H	2.2978239	5.0321457	-2.6214424
C	-1.3900828	2.0509844	0.0361632
C	-1.5736444	2.8975544	-1.0880286
C	-1.3242688	2.6504475	1.3016545
C	-1.6282498	4.2837402	-0.9010231
C	-1.3986456	4.0309739	1.4784115
H	-1.2233592	2.0206307	2.1815795
C	-1.5359772	4.8572721	0.3657792
H	-1.7486112	4.9221823	-1.7737687
H	-1.3387063	4.4545314	2.4775415
H	-1.5793697	5.9370788	0.4807131
C	2.1384182	3.0498572	1.5908270
H	1.1831389	2.8321715	2.0837240
H	2.8742314	2.3311521	1.9677717
H	2.4424173	4.0548283	1.8963773
C	-1.7220346	2.3470740	-2.4841388
H	-2.5334676	1.6129492	-2.5359386
H	-1.9318610	3.1514323	-3.1945965
H	-0.8039203	1.8473796	-2.8165029

(**A**)$_2$: BH/BB dimer of H(*o*-tolyl)BB(*o*-tolyl)$_2$
90
Energy = -1727.512962129

B	0.0405050	-0.0512976	-1.4918637
B	-0.6597126	1.1296612	-2.4896699
B	1.4765992	-0.6853100	-0.9521287
H	1.0619104	-0.7361111	-2.1875699
B	1.4903275	0.9972410	-0.5315486
H	2.4757569	0.1076147	-0.9362617
C	1.6025400	1.1555112	1.0447894
C	0.4694035	1.3900117	1.8395957
C	2.8596554	1.0713741	1.6885517
C	0.5505565	1.5259272	3.2240100
H	-0.4980134	1.5065537	1.3592216
C	2.9329697	1.2015501	3.0809755
C	1.7919579	1.4219050	3.8517317
H	-0.3474066	1.7133649	3.8068329
H	3.9042381	1.1294024	3.5661044
H	1.8734013	1.5186384	4.9311175
C	-1.0588027	-1.1315694	-1.0254837
C	-1.6539371	-2.0816303	-1.8855414
C	-1.4749825	-1.1095288	0.3194877
C	-2.6577837	-2.9244406	-1.3843958
C	-2.4791777	-1.9419362	0.8020820
H	-0.9817156	-0.4337544	1.0098899
H	-3.1038317	-3.6579120	-2.0539055
C	-3.0860189	-2.8545173	-0.0619880
H	-2.7708794	-1.8923337	1.8478978
H	-3.8680493	-3.5193307	0.2953228
C	1.8044876	-2.0386033	-0.2417231
C	2.2943173	-3.1393912	-0.9822636
C	1.6328005	-2.1834468	1.1454832
C	2.5781891	-4.3397387	-0.3216117
C	1.9208335	-3.3835806	1.7927008
H	1.2604073	-1.3440365	1.7235323
H	2.9548966	-5.1831386	-0.8965731
C	2.3925699	-4.4692234	1.0552902
H	1.7741391	-3.4709396	2.8660135
H	2.6189440	-5.4115673	1.5474155
C	-1.4334795	2.3462179	-1.8777017
C	-1.4354818	3.6338239	-2.4759908
C	-2.1746413	2.1689360	-0.6923050
C	-2.1422578	4.6720794	-1.8596903
C	-2.9075260	3.2009949	-0.1100507
H	-2.2000929	1.1839354	-0.2354497
C	-2.8810776	4.4669653	-0.6935964
H	-2.1133124	5.6641271	-2.3059182
H	-3.4791637	3.0231212	0.7969696
H	-3.4254668	5.2927806	-0.2431028
C	-0.7230280	0.8573870	-4.0378981
C	0.4441645	0.5432298	-4.7569817
C	-1.9529056	0.8771753	-4.7470369
C	0.4371977	0.3204585	-6.1312171
H	1.3879136	0.4914673	-4.2243718
C	-1.9543302	0.6147128	-6.1217630
C	-0.7746252	0.3560660	-6.8206485
H	1.3640633	0.1031861	-6.6554425
H	-2.9035253	0.6102063	-6.6540177
H	-0.8064631	0.1668704	-7.8904284
C	1.9297804	2.2058814	-1.4803415
C	1.6906399	3.5755241	-1.1908727
C	2.7213568	1.9158261	-2.6111081
C	2.2158178	4.5573118	-2.0412218
C	3.2331501	2.8991447	-3.4530257
H	2.9709057	0.8778571	-2.8240109
C	2.9754844	4.2377463	-3.1648620
H	2.0135492	5.6016129	-1.8134822
H	3.8358033	2.6217364	-4.3133656
H	3.3653976	5.0260555	-3.8030227
C	-0.6914266	3.9319713	-3.7564109
H	0.2689305	3.4150679	-3.8017631
H	-1.2751265	3.6124996	-4.6283296

H -0.5098133 5.0070710 -3.8469129
C -3.2777819 1.1258065 -4.0608968
H -3.3627699 0.5593130 -3.1281603
H -3.4035599 2.1831859 -3.8030163
H -4.1034391 0.8356179 -4.7175590
C -1.2348357 -2.2737727 -3.3244198
H -2.0023535 -1.9076819 -4.0157268
H -0.3137698 -1.7420511 -3.5650291
H -1.0853940 -3.3387160 -3.5343986
C 2.5295296 -3.0348951 -2.4713086
H 1.5889919 -2.8952838 -3.0161868
H 3.1750710 -2.1821502 -2.7156736
H 3.0074228 -3.9401857 -2.8557695
C 0.9057022 4.0384988 0.0108396
H -0.0348553 3.4937432 0.1154054
H 1.4755420 3.8871836 0.9340768
H 0.6744834 5.1033064 -0.0809512
C 4.1237451 0.8177389 0.9023903
H 4.2007106 1.4911997 0.0408779
H 5.0080828 0.9578335 1.5301450
H 4.1485739 -0.2112295 0.5186564

Ad1 : first open triborane(5) (*o*-tolyl)$_3$B$_3$H$_2$
47
Energy = -889.1770090760
B -0.9899026 -1.5710576 -2.0926023
B -1.2141733 -0.0478579 -2.7662803
B 0.6125612 -1.8182719 -1.8861791
C -2.1645679 -2.4927345 -1.6350578
C -1.4460239 0.9968302 -1.6100397
C -1.0536618 0.3048983 -4.2735885
H 1.2492084 -2.4074019 -2.7203174
H 1.2574394 -1.3337444 -0.9959189
C -3.4737837 -2.4341706 -2.1878861
C -1.9263541 -3.4204167 -0.5953492
C -0.4949506 2.0173606 -1.3540022
C -2.5636108 0.8996419 -0.7607849
C -0.2538010 -0.5500237 -5.0641368
C -1.6554777 1.4277758 -4.9088210
C -4.4689156 -3.2798933 -1.6866536
C -3.8153875 -1.4974245 -3.3183455
C -2.9318334 -4.2382276 -0.0861230
H -0.9265377 -3.4822573 -0.1726231
C -0.7145459 2.9121786 -0.3028066
C 0.7549096 2.1371388 -2.1931941
C -2.7869266 1.8170283 0.2657068
H -3.2800749 0.0961472 -0.9127435
C 0.0008813 -0.2999776 -6.4102728
H 0.1868003 -1.4279498 -4.5982928
C -1.4185829 1.6494216 -6.2687899
C -2.5614351 2.3840781 -4.1705996
H -5.4664754 -3.2377671 -2.1178957
C -4.2101786 -4.1683934 -0.6408882
H -4.8849179 -1.5309027 -3.5442783
H -3.5610073 -0.4593457 -3.0660331
H -3.2595506 -1.7443861 -4.2286271
H -2.7197456 -4.9298622 0.7244603
C -1.8576559 2.8306346 0.4958461
H 0.0213113 3.6907317 -0.1111108
H 1.3417955 1.2095404 -2.1681471
H 0.5121660 2.3277511 -3.2445767
H 1.3917003 2.9495896 -1.8321043
H -3.6710708 1.7292370 0.8916370
C -0.5895387 0.8087409 -7.0157720
H 0.6363294 -0.9692445 -6.9836199
H -1.8880608 2.5029345 -6.7532603
H -3.3366688 1.8563340 -3.6060419
H -2.0013763 2.9835595 -3.4450282
H -3.0486312 3.0651598 -4.8742738
H -5.0061681 -4.8056194 -0.2646856
H -2.0112190 3.5459094 1.2994978
H -0.4195147 1.0146606 -8.0693311

Ad2 : second open triborane(5) (*o*-tolyl)$_3$B$_3$H$_2$
47
Energy = -889.1853231749
B 0.4582983 0.0536659 -0.0251368
B -0.4384741 -1.2974749 -0.3469487
B -0.5452047 1.3677227 0.2057930
C 2.0171631 0.1611122 -0.0526926
C -1.1141987 -2.2836163 0.6442994
C -1.0753814 2.2443547 -0.9590655
H -0.7113451 -1.4708712 -1.5125957
H -0.9297830 1.6165649 1.3186589
C 2.8586150 -0.9562784 -0.3123217
C 2.6337592 1.4110516 0.1792569
C -0.8431076 -2.2838591 2.0410637
C -2.0472056 -3.2218804 0.1494000
C -0.6992009 1.9178605 -2.2830447
C -1.9167788 3.3786682 -0.7672288
C 4.2471772 -0.7841431 -0.3292171
C 2.2853260 -2.3253401 -0.5726932
C 4.0163765 1.5727433 0.1664626
H 1.9998699 2.2742059 0.3709508
C -1.5101726 -3.1905026 2.8707549
C 0.1729227 -1.3454026 2.6393224

C	-2.7103642	-4.1177619	0.9839986
H	-2.2515058	-3.2319655	-0.9187980
C	-1.1249614	2.6550235	-3.3835584
H	-0.0540936	1.0561107	-2.4410414
C	-2.3355236	4.1158158	-1.8797175
C	-2.3706056	3.8130080	0.6065254
H	4.8875894	-1.6396530	-0.5318052
C	4.8255353	0.4642660	-0.0899624
H	3.0753575	-3.0485988	-0.7952802
H	1.7245768	-2.6934244	0.2948957
H	1.5916231	-2.3089249	-1.4230736
H	4.4600792	2.5475604	0.3492268
C	-2.4373420	-4.0980737	2.3532259
H	-1.3016038	-3.1892985	3.9383453
H	0.1415295	-1.3748891	3.7324599
H	1.1891020	-1.6096305	2.3230661
H	-0.0025025	-0.3108032	2.3207137
H	-3.4301846	-4.8229821	0.5775039
C	-1.9492612	3.7625232	-3.1751927
H	-0.8202453	2.3764790	-4.3884650
H	-2.9774918	4.9816080	-1.7327099
H	-2.9229499	3.0149047	1.1133585
H	-1.5186226	4.0669758	1.2460916
H	-3.0194516	4.6905622	0.5347967
H	5.9073353	0.5692406	-0.1065766
H	-2.9433724	-4.7907782	3.0209038
H	-2.2930637	4.3544002	-4.0196622

Ad : cyclic triborane(5) (*o*-tolyl)$_3$B$_3$H$_2$
47
Energy = -889.2028189505

B	-0.5222133	0.8046568	0.2549867
B	-1.9457191	0.2034569	0.9671659
H	-1.2657185	1.2715952	1.2848478
B	-0.7114827	-0.7626921	0.3571391
H	-1.4531622	-0.9145543	1.4608341
C	-3.4272324	0.3990635	1.3890912
C	-3.8860161	1.6773324	1.7630808
C	-4.3551291	-0.6729435	1.3532893
H	-3.1796227	2.5042399	1.8018995
C	-5.2202689	1.9089840	2.0895369
C	-5.6918746	-0.4262274	1.6826281
H	-5.5490965	2.9044015	2.3746401
C	-6.1278445	0.8492074	2.0481311
H	-6.4039036	-1.2479354	1.6491345
H	-7.1726197	1.0130905	2.2979925
C	0.0080614	2.0349119	-0.5202998
C	1.2987779	2.0558703	-1.1071692
C	-0.8187657	3.1700118	-0.6834707
C	1.6974958	3.1827480	-1.8408425
C	-0.4124332	4.2795662	-1.4152374
H	2.6873731	3.1958463	-2.2924200
C	0.8579250	4.2825471	-2.0000067
H	-1.0737321	5.1337088	-1.5319186
H	1.1935544	5.1422124	-2.5741119
C	-0.4439772	-2.1534037	-0.2673197
C	-0.2785324	-2.2929265	-1.6687099
C	-0.3455943	-3.3064505	0.5352659
C	-0.0128127	-3.5565516	-2.2060861
C	-0.0702009	-4.5587721	-0.0118169
H	0.1054295	-3.6568045	-3.2832590
C	0.0987700	-4.6852155	-1.3913246
H	0.0068160	-5.4298793	0.6335654
H	0.3092897	-5.6558402	-1.8326725
H	-0.4805737	-3.2180345	1.6116112
H	-1.8093597	3.1674216	-0.2335153
C	-3.9198532	-2.0624289	0.9594637
H	-3.3880544	-2.0565966	0.0011354
H	-3.2293612	-2.4888397	1.6982748
H	-4.7799533	-2.7322921	0.8772774
C	2.2751286	0.9142665	-0.9470834
H	3.1491795	1.2391583	-0.3690676
H	1.8182131	0.0683570	-0.4297589
H	2.6434944	0.5750564	-1.9221085
C	-0.4315819	-1.0992629	-2.5775737
H	-1.4347763	-0.6641574	-2.4860204
H	0.2768887	-0.3057347	-2.3163716
H	-0.2700496	-1.3751193	-3.6235644

A·SMe$_2$: SMe$_2$ adduct of **A** HB$_2$(*o*-tolyl)$_3$
54
Energy = -1341.869921656

B	0.4884409	0.8561962	0.3946737
C	-0.8547251	0.1093043	0.0717794
C	1.8332805	0.2050440	-0.0836976
C	-1.5753436	0.3887979	-1.1023536
C	-1.3937663	-0.8252654	0.9851749
C	2.9845643	0.0373726	0.7215985
C	1.9377898	-0.0869282	-1.4613792
C	-2.7909101	-0.2399775	-1.3798534
H	-1.1916190	1.1274254	-1.8035100
C	-2.6195540	-1.4370114	0.7066994
C	4.1927961	-0.3517583	0.1301423
C	3.1508581	-0.4475196	-2.0448378
H	1.0528517	-0.0014975	-2.0886033
H	-3.3271811	-0.0052222	-2.2960378

C -3.3204155 -1.1532208 -0.4681464
H -3.0318430 -2.1454714 1.4230863
C 4.2885267 -0.5737009 -1.2437608
H 5.0727275 -0.4816472 0.7574880
H 3.2076364 -0.6382729 -3.1133921
H -4.2725589 -1.6388241 -0.6656274
H 5.2404904 -0.8588867 -1.6836805
B 0.3170648 2.4300162 1.0343947
H -0.2441964 3.0684445 0.1587388
S 2.0696298 3.3614810 1.1331249
C 2.5533608 3.4992837 -0.6055916
H 2.8702696 2.5067775 -0.9267988
H 1.6938017 3.8340696 -1.1900043
H 3.3818138 4.2054560 -0.6796069
C 1.5740967 5.0739199 1.4613235
H 0.8148880 5.3694135 0.7344396
H 1.1563914 5.0877321 2.4691274
H 2.4531873 5.7179866 1.4054160
C -0.6494663 -1.1322217 2.2629336
H 0.3497429 -1.5313298 2.0465243
H -0.5137975 -0.2222068 2.8605049
H -1.1845509 -1.8669630 2.8719013
C 2.9010138 0.1985908 2.2185664
H 3.7668609 0.7356058 2.6192896
H 1.9895651 0.7314211 2.5028035
H 2.8654171 -0.7828823 2.7088774
C -0.3791526 2.5516436 2.4803328
C -1.7873680 2.6556783 2.6232804
C 0.3819206 2.5170022 3.6651226
C -2.3561883 2.6934780 3.9034093
C -0.1905507 2.5616724 4.9366826
H 1.4673613 2.4544794 3.5982776
C -1.5767093 2.6416035 5.0595913
H -3.4386151 2.7724460 3.9918465
H 0.4423669 2.5328013 5.8204916
H -2.0465507 2.6718892 6.0394652
C -2.7027277 2.7326003 1.4259318
H -2.3498393 3.4844906 0.7110641
H -2.7362848 1.7809218 0.8862741
H -3.7209051 2.9895799 1.7353972

A·THF : THF adduct of **A** $HB_2(o\text{-tolyl})_3$
58
Energy = -1096.349185378
B 0.6278045 -0.4027676 -0.2197533
C 2.0642036 -0.3009131 0.4145175
C 3.0872440 -1.0133586 -0.2471005
C 4.4265907 -0.9120766 0.1279328
C 4.7712114 -0.1284426 1.2290089
C 3.7714331 0.5483050 1.9311795
C 2.4311329 0.4891690 1.5343564
C 1.3936901 1.2445352 2.3251371
H 0.6059340 0.5786368 2.6934351
H 0.9007273 1.9963546 1.6991096
H 1.8453829 1.7526806 3.1820601
H 4.0366772 1.1407821 2.8047122
H 5.8071511 -0.0524834 1.5491764
H 5.1904680 -1.4575576 -0.4203931
H 2.8199340 -1.6544978 -1.0843285
C 0.1633057 -1.8553476 -0.6133542
C 0.2264609 -2.2909544 -1.9477774
C -0.1979188 -3.5674136 -2.3221234
C -0.7267423 -4.4276240 -1.3598047
C -0.8080588 -4.0061773 -0.0299877
C -0.3608713 -2.7394372 0.3576015
C -0.4676055 -2.2931907 1.7962665
H -1.1422463 -1.4325661 1.8856400
H 0.5121688 -1.9792228 2.1784495
H -0.8460531 -3.0950438 2.4373277
H -1.2265971 -4.6731479 0.7218936
H -1.0778523 -5.4175302 -1.6398435
H -0.1329237 -3.8800893 -3.3615050
H 0.6007616 -1.6120938 -2.7114424
B -0.5024703 0.7800892 -0.6957038
C -1.8720176 0.6748496 0.1460445
C -2.0085959 1.3784087 1.3602009
C -3.1388742 1.2818724 2.1705359
C -4.1911094 0.4527697 1.7817095
C -4.0921781 -0.2435017 0.5778339
C -2.9605435 -0.1474340 -0.2452629
C -2.9544508 -0.9360872 -1.5323619
H -2.4358338 -0.3907644 -2.3265549
H -3.9791100 -1.1530792 -1.8531710
H -2.4281707 -1.8893451 -1.4126668
H -4.9186713 -0.8759950 0.2570669
H -5.0807699 0.3570208 2.3994047
H -3.1947732 1.8462660 3.0986602
H -1.2002453 2.0283704 1.6827572
H -0.6588576 0.6687590 -1.9037802
O 0.0284877 2.3122130 -0.5485476
C -0.9211436 3.3775798 -0.9956562
C -0.1068963 4.2659084 -1.9253466
H -0.4344176 5.3063249 -1.8703816
C 1.3343507 4.0633068 -1.4312944
H 2.0817143 4.3374305 -2.1791110
H 1.5198540 4.6409733 -0.5200797

C 1.3620824 2.5776078 -1.1350805
H 1.4453618 1.9677764 -2.0399048
H 2.0916488 2.2538686 -0.3950271
H -0.2015060 3.9242776 -2.9605607
H -1.7608317 2.8672118 -1.4659095
H -1.2458692 3.8849942 -0.0865572

A : monohydridodibroane(4) $HB_2(o\text{-tolyl})_3$
45
Energy = -863.7285936596
B -0.7853584 -0.8575642 -1.0307037
C -1.9552594 -1.4843185 -0.2303913
B 0.4485012 0.0761923 -0.3980068
C -2.9296864 -2.3484986 -0.8091375
C -2.0839141 -1.1721403 1.1431588
C 0.2329227 1.6278100 -0.5037010
C 1.7296822 -0.5704250 0.2276922
C -3.9607495 -2.8497117 -0.0077422
C -3.1195189 -1.6660417 1.9292079
H -1.3428801 -0.5164373 1.5958413
C -0.1361383 2.1666762 -1.7539062
C 0.3642541 2.5159768 0.5949194
C 3.0421616 -0.0288914 0.1432836
C 1.5568655 -1.7738571 0.9461290
C -4.0622557 -2.5136345 1.3443656
H -4.7010199 -3.5136905 -0.4484288
H -3.1930990 -1.4015644 2.9801724
C -0.3073631 3.5378764 -1.9447250
H -0.2629320 1.4954557 -2.6005080
C 0.1513001 3.8835834 0.3991026
C 4.0940437 -0.6865425 0.7892905
C 2.6059371 -2.3980890 1.6184447
H 0.5634532 -2.2145514 0.9917701
H -4.8779974 -2.9161432 1.9393377
H -0.5657512 3.9247522 -2.9269142
C -0.1656994 4.4015879 -0.8596525
H 0.2420306 4.5581494 1.2482547
C 3.8847887 -1.8486460 1.5363021
H 5.0992197 -0.2775213 0.7100430
H 2.4326971 -3.3111795 2.1814998
H -0.3119186 5.4712175 -0.9848071
H 4.7221298 -2.3280253 2.0366568
C 3.3493550 1.2269801 -0.6397547
H 2.8643517 1.2240537 -1.6199422
H 2.9931557 2.1200962 -0.1142926
H 4.4289701 1.3285066 -0.7849354
C 0.7222125 2.0146616 1.9753245
H 1.7427472 1.6168583 2.0016798
H 0.6499320 2.8214221 2.7103025
H 0.0601671 1.2024387 2.2957840
C -2.8776098 -2.7495408 -2.2641100
H -1.9523933 -3.2874107 -2.4964079
H -3.7226714 -3.3978642 -2.5123471
H -2.9099250 -1.8722806 -2.9186253
H -0.7811076 -1.0078586 -2.2273137

B0 : BH/BC adduct of HBcat and **A**
59
Energy = -1271.015050248
B -0.0893536 -0.1411391 -0.2669633
C 1.3214076 -0.9898319 -0.6075109
B 0.0692973 1.3084329 0.6225459
C 1.5511875 -1.2759153 -1.9763230
C 2.4491719 -0.9533877 0.2708928
C 0.7430217 2.5040152 -0.1416314
C -0.5534677 1.4982203 2.0473380
C 2.8239139 -1.5141904 -2.4744502
H 0.6938174 -1.3194160 -2.6399930
C 3.7166868 -1.2214256 -0.2443550
C 2.0328320 2.3585065 -0.6896231
C 0.0792037 3.7450594 -0.3308264
C -0.0248025 2.3583319 3.0504416
C -1.7382314 0.7941508 2.3501042
C 3.9119266 -1.4955083 -1.6000802
H 2.9678183 -1.7208639 -3.5304224
H 4.5724543 -1.1919422 0.4249722
C 2.6822571 3.4034602 -1.3457622
H 2.5498925 1.4096274 -0.5841000
C 0.7254842 4.7731363 -1.0247345
C -0.6875155 2.4681075 4.2775678
C -2.4163220 0.9481553 3.5566891
H -2.1589181 0.1253940 1.6027213
H 4.9141178 -1.6914224 -1.9705131
H 3.6873938 3.2623144 -1.7344605
C 2.0237238 4.6201850 -1.5162333
H 0.2016085 5.7144342 -1.1788460
C -1.8794516 1.7871200 4.5322382
H -0.2644736 3.1089794 5.0483678
H -3.3395475 0.4059059 3.7410922
H 2.5074696 5.4408622 -2.0393148
H -2.3767418 1.9047807 5.4913811
C 1.2421130 3.1594982 2.8544643
H 2.0346349 2.5742303 2.3792714
H 1.0662144 4.0262927 2.2079004
H 1.6111976 3.5243206 3.8174781
C -1.3240884 3.9806916 0.1823342

H -1.3394063 4.0641499 1.2749233
H -1.7342517 4.9051522 -0.2346258
H -1.9961213 3.1571349 -0.0812633
C 2.3097746 -0.5783042 1.7181209
H 2.0121193 0.4749259 1.8097572
H 1.5411388 -1.1707228 2.2195997
H 3.2590386 -0.7020761 2.2449866
C -0.1108511 -4.0321705 0.7833467
C -0.7103466 -4.0243166 -0.4801273
C -1.1469878 -5.1876724 -1.0895757
C -0.9601039 -6.3830841 -0.3745401
C -0.3619125 -6.3903672 0.8889446
C 0.0787730 -5.2025810 1.4971095
H -1.6109183 -5.1748524 -2.0704528
H -1.2875773 -7.3191075 -0.8167862
H -0.2332362 -7.3316626 1.4146780
H 0.5430728 -5.2001764 2.4778115
O -0.7849756 -2.7317513 -0.9554920
O 0.2181384 -2.7467698 1.1552801
B -0.1120929 -1.9329168 0.0240577
H -0.8331623 -0.9121987 0.4822707
H -0.7345150 -0.0387629 -1.2750908

B_2H_6 : diborane(6)
8
Energy = -53.33049392421
B -0.6953467 -0.5439932 0.0516204
B 0.6928950 0.5350454 -0.0427703
H -0.5109092 -1.7161537 0.1418302
H 0.0059137 0.0717981 0.9812970
H -0.0083449 -0.0807851 -0.9724387
H 0.5084932 1.7072137 -0.1329531
H -1.7908157 -0.0800377 0.0233748
H 1.7883472 0.0710502 -0.0145460

(**B**)$_2$: BH/BH dimer [(*o*-toly)$_2$BBH$_2$]$_2$
64
Energy = -1186.408993684
B 0.4867241 0.0635718 0.6712002
B 2.0746665 -0.1337899 0.1199468
C 2.8471009 1.1340103 -0.3821999
C 2.7159470 -1.5573990 0.1871409
C 2.2443457 1.9339478 -1.3735374
C 4.1211838 1.5228642 0.1061468
C 3.6763304 -2.0696383 -0.7279403
C 2.3043701 -2.3949965 1.2479198
C 2.8877247 3.0434674 -1.9211208
H 1.2598613 1.6611442 -1.7471085
C 4.7376807 2.6627082 -0.4189947
C 4.1845117 -3.3584831 -0.5339644
C 2.8471048 -3.6609783 1.4538198
H 1.5544323 -2.0250561 1.9443334
H 2.4065732 3.6239192 -2.7038314
C 4.1416776 3.4126943 -1.4363960
H 5.7068808 2.9660553 -0.0279568
C 3.7931012 -4.1460450 0.5511668
H 4.9059204 -3.7532554 -1.2464268
H 2.5244741 -4.2686293 2.2948533
H 4.6503318 4.2862589 -1.8355227
H 4.2160938 -5.1389951 0.6794204
C 4.1540054 -1.2799155 -1.9251236
H 3.3243243 -0.8078418 -2.4589542
H 4.8344172 -0.4750603 -1.6253247
H 4.6893361 -1.9329735 -2.6207178
C 4.8177311 0.7397983 1.1957640
H 5.1260434 -0.2491305 0.8380130
H 5.7093076 1.2702076 1.5426324
H 4.1600911 0.5739343 2.0559776
H -0.3374004 -0.8851731 0.2574951
H 0.2663329 0.2041594 1.8419740
B -0.9294047 0.0906782 -0.4024562
B -2.5752429 0.2238913 -0.0327059
C -3.3784385 -1.1267173 0.0819278
C -3.3869592 1.5561934 0.0443073
C -4.3651129 -1.3603380 -0.9002394
C -3.1335141 -2.1438457 1.0331562
C -2.9477448 2.8157417 -0.4544149
C -4.6326195 1.5170361 0.7152082
C -5.0592935 -2.5669353 -0.9756560
H -4.5846863 -0.5773664 -1.6233049
C -3.8614546 -3.3384948 0.9702191
C -3.7533758 3.9455416 -0.2739569
C -5.4108094 2.6530611 0.9167200
H -4.9837472 0.5601671 1.0920168
H -5.8050610 -2.7202002 -1.7513111
C -4.8064275 -3.5630624 -0.0312243
H -3.6872041 -4.1041908 1.7240094
C -4.9676838 3.8758057 0.4111020
H -3.4217717 4.9006139 -0.6755684
H -6.3553653 2.5865474 1.4495939
H -5.3520154 -4.5024555 -0.0633053
H -5.5663354 4.7735525 0.5418658
C -1.6407780 2.9891901 -1.1903464
H -0.8003232 3.0539664 -0.4876115
H -1.4387290 2.1599072 -1.8739601
H -1.6477953 3.9170119 -1.7697746

C	-2.1290437	-1.9572872	2.1487351
H	-2.4968526	-2.3999240	3.0801323
H	-1.1768446	-2.4502296	1.9127453
H	-1.9177082	-0.9009345	2.3349882
H	-0.1571760	1.0465299	0.0779600
H	-0.6453684	-0.0560093	-1.5608748

BH_3·THF : THF adduct of BH_3
17
Energy = -259.2680101440

H	-2.1818067	0.1699767	1.4319513
B	-2.2465917	-0.0033210	0.2277580
H	-2.7069988	-1.0824325	-0.0772396
H	-2.7518807	0.9266252	-0.3631821
C	0.0564729	1.2027975	-0.1502258
O	-0.7415304	-0.0468738	-0.2995243
C	0.0771622	-1.2035705	0.1202848
C	1.4926533	-0.7352528	-0.1497279
C	1.4395253	0.7373060	0.2966752
H	0.0468168	1.6726979	-1.1345242
H	-0.4625895	1.8277264	0.5770666
H	-0.1168607	-1.3810570	1.1829277
H	-0.2654732	-2.0470904	-0.4777719
H	2.2254916	-1.3175786	0.4129985
H	1.7232777	-0.8139273	-1.2165471
H	1.5288276	0.8029450	1.3848417
H	2.2292393	1.3429734	-0.1527298

BH_3 : monoborane(3)
4
Energy = -26.62842989418

B	0.0104825	1.9877825	0.4034786
H	0.0088804	1.4975719	1.4915349
H	-1.0222052	2.2378603	-0.1398272
H	1.0447619	2.2277532	-0.1413480

B·THF : THF adduct of **B** H_2BB(*o*-tolyl)$_2$
45
Energy = -825.8045342277

B	0.3411752	-0.5249294	0.0632821
C	-0.1830354	0.9097259	0.4401867
C	-0.9133959	1.0525334	1.6378642
C	-1.3927425	2.2876332	2.0719605
C	-1.1942397	3.4164207	1.2767015
C	-0.5091949	3.2935684	0.0657064
C	0.0118489	2.0660190	-0.3579209
C	0.7627108	1.9977690	-1.6687546
H	0.3765602	1.2013744	-2.3146218
H	1.8239746	1.7803388	-1.5046636
H	0.6851775	2.9463767	-2.2087267
H	-0.3671693	4.1721747	-0.5611021
H	-1.5782719	4.3844350	1.5883609
H	-1.9347401	2.3653318	3.0111954
H	-1.0964223	0.1697644	2.2460272
C	1.8386969	-0.7376719	-0.3661176
C	2.0855335	-1.5440296	-1.4963139
C	3.3684798	-1.7309717	-2.0124083
C	4.4559973	-1.1469138	-1.3644698
C	4.2432425	-0.3787635	-0.2165771
C	2.9570448	-0.1513552	0.2838132
C	2.7956039	0.7102640	1.5160259
H	2.1620786	0.2273936	2.2672473
H	3.7695921	0.9204102	1.9685047
H	2.3184293	1.6654539	1.2698060
H	5.0955901	0.0620340	0.2973557
H	5.4655411	-1.2938630	-1.7399277
H	3.5191877	-2.3418171	-2.8990176
H	1.2398882	-2.0193741	-1.9869480
B	-0.6550235	-1.8901868	0.1830427
H	-0.4100628	-2.4619740	1.2348866
H	-0.6242781	-2.6838601	-0.7441072
O	-2.2029355	-1.4817856	0.3379223
C	-2.8312783	-0.7604489	-0.7798701
C	-4.2964944	-0.7725489	-0.3966705
H	-4.9448523	-0.5710806	-1.2520111
C	-4.4804686	-2.1981414	0.1598223
H	-5.3162434	-2.2692608	0.8587975
C	-3.1471851	-2.5086564	0.8444767
H	-2.7332466	-3.4844995	0.5860916
H	-3.1615955	-2.3786189	1.9272666
H	-4.6556022	-2.8999170	-0.6600989
H	-4.4876045	-0.0215039	0.3760525
H	-2.3562853	0.2194057	-0.8049011
H	-2.6246274	-1.3162460	-1.7009569

B : dihydridodiborane(4) H_2BB(*o*-tolyl)$_2$
32
Energy = -593.1744569650

B	0.4057258	0.3388879	-0.3549091
B	1.2264263	0.2828611	1.0536316
C	2.5701400	-0.0457743	0.2837425
C	0.9956251	0.5013384	2.5610966
C	2.8166264	-1.3364502	-0.2273057
C	3.5098503	0.9856437	0.0156332
C	1.9482407	0.2444544	3.5891687
C	-0.2678384	1.0156055	2.9376697

C	3.9922682	-1.6281906	-0.9199092
H	2.0874667	-2.1245192	-0.0564767
C	4.6751658	0.6795229	-0.6880780
C	1.6023516	0.5150213	4.9172073
C	-0.5942137	1.2916078	4.2618740
H	-0.9982509	1.2046454	2.1545504
H	4.1702715	-2.6343536	-1.2892546
C	4.9252003	-0.6175700	-1.1477823
H	5.3995773	1.4681172	-0.8808690
C	0.3524419	1.0386818	5.2559030
H	2.3255033	0.3111387	5.7037185
H	-1.5714069	1.6903938	4.5191773
H	5.8410508	-0.8308084	-1.6923350
H	0.1193730	1.2431383	6.2977306
C	3.3183882	-0.3196265	3.3018441
H	3.2578637	-1.2545348	2.7365583
H	3.9181042	0.3718613	2.7004996
H	3.8530114	-0.5112927	4.2363693
C	3.2543144	2.3853021	0.5161462
H	3.2200434	2.4087553	1.6128407
H	4.0350767	3.0745326	0.1834480
H	2.2868190	2.7604322	0.1603292
H	0.0036640	-0.6717077	-0.8652536
H	0.3942823	1.3221369	-1.0443040

(**C**)$_2$: BB/BB dimer of dihydridodiborane(4) **C**
64
Energy = -1186.432775118

B	0.2784762	-0.2878373	-1.4020676
B	0.1335224	1.3787817	-2.0135148
B	1.7957506	-0.7598191	-0.9999397
H	1.4111984	-0.9109644	-2.2098637
B	1.2445243	0.9103587	-0.6892175
H	2.5696258	0.2243256	-0.9587020
C	-0.9863105	-1.1859580	-1.2181674
C	-1.9816276	-1.4002258	-2.1993424
C	-1.1520843	-1.7763907	0.0494698
C	-3.0941725	-2.1870534	-1.8805714
C	-2.2791327	-2.5344927	0.3627812
H	-0.3765439	-1.6372729	0.7982296
H	-3.8521111	-2.3596283	-2.6420855
C	-3.2548249	-2.7446086	-0.6111110
H	-2.3849140	-2.9709741	1.3525288
H	-4.1333783	-3.3449631	-0.3891106
C	2.2964980	-1.9792856	-0.1613231
C	2.0985222	-3.3243647	-0.5570432
C	2.9301796	-1.7336144	1.0717229
C	2.5182003	-4.3564838	0.2907802
C	3.3353014	-2.7698269	1.9118999
H	3.1015838	-0.7043289	1.3824683
H	2.3628551	-5.3877577	-0.0202716
C	3.1270137	-4.0927183	1.5188710
H	3.8151907	-2.5463899	2.8613116
H	3.4416540	-4.9134115	2.1582774
C	-1.1662491	2.2421661	-1.7760774
C	-1.5224805	3.2831170	-2.6683595
C	-2.0294013	1.9678983	-0.7005242
C	-2.7051888	4.0004821	-2.4567104
C	-3.2095825	2.6838025	-0.4986535
H	-1.7701550	1.1686711	-0.0113829
C	-3.5481004	3.7086894	-1.3818108
H	-2.9708417	4.8001644	-3.1456597
H	-3.8573041	2.4452840	0.3411181
H	-4.4626650	4.2787570	-1.2386869
C	1.6956969	2.2544260	-1.6356382
C	1.6787327	3.5396870	-1.0124619
C	2.5817741	2.0535936	-2.7260602
C	2.5104808	4.5432706	-1.5250128
C	3.3866564	3.0615548	-3.2250370
H	2.6197441	1.0657886	-3.1797118
C	3.3459220	4.3214755	-2.6169520
H	2.4987494	5.5214358	-1.0512798
H	4.0392857	2.8765354	-4.0726527
H	3.9718004	5.1276867	-2.9893679
C	-0.6372192	3.6296120	-3.8401349
H	0.3604604	3.9368167	-3.5059000
H	-0.4941228	2.7607120	-4.4938302
H	-1.0690025	4.4420495	-4.4319285
C	-1.8719957	-0.7982661	-3.5784761
H	-1.9985541	0.2899138	-3.5337868
H	-0.8909887	-0.9901891	-4.0266821
H	-2.6399885	-1.2061282	-4.2421545
C	1.4422514	-3.6635588	-1.8729978
H	0.4026586	-3.3166982	-1.8932649
H	1.9598878	-3.1820559	-2.7117532
H	1.4469158	-4.7440742	-2.0434008
C	0.8248768	3.8646068	0.1785938
H	-0.2306651	3.6560836	-0.0195580
H	1.1101774	3.2506402	1.0404923
H	0.9374429	4.9174231	0.4488917
H	0.3313435	1.0217125	-3.1440392
H	1.0504245	1.2195569	0.4414411

C·Cd : BH/BB adduct of **C** and **Cd**
66
Energy = -1211.916937261

B	-0.8082896	0.8043099	-0.3515281
H	-0.3843193	0.5011156	-1.5086200
H	-0.8808211	2.1453173	-0.2721972
B	0.3209794	1.8697655	0.2752734
B	0.8965250	0.5986720	-0.7046595
H	0.3848068	2.2010670	1.4091842
B	0.4138294	3.3588886	-1.3629033
H	1.3627822	1.2575664	-1.7911513
H	-0.3654073	2.9842118	-2.2011674
B	1.5546795	2.2178744	-0.8935264
C	-2.0649979	0.0615189	0.1470524
C	-2.8096247	0.6223196	1.2054412
C	-2.4865659	-1.1776261	-0.4005766
C	-3.9349169	-0.0112624	1.7229504
H	-2.4927824	1.5731615	1.6284311
C	-3.6141352	-1.8073267	0.1374622
C	-4.3369336	-1.2364533	1.1860798
H	-4.4920675	0.4420237	2.5379121
H	-3.9331259	-2.7610196	-0.2768561
H	-5.2123993	-1.7456667	1.5798772
C	1.5852145	-0.7636395	-0.4013379
C	2.5918142	-1.3229595	-1.2260062
C	1.2146614	-1.4479264	0.7723762
C	3.1846069	-2.5317112	-0.8474879
C	1.8168972	-2.6485732	1.1404528
H	0.4386701	-1.0233970	1.4049217
C	2.8071972	-3.1930680	0.3226914
H	3.9580300	-2.9611952	-1.4803241
H	1.5159181	-3.1552767	2.0531241
H	3.2857004	-4.1309908	0.5918954
C	3.0903419	2.4065038	-0.5583728
C	3.9505400	3.0973054	-1.4457059
C	3.6378594	1.8940699	0.6303362
C	5.3024341	3.2521589	-1.1171314
C	4.9844158	2.0588722	0.9550922
H	2.9905015	1.3515610	1.3151887
C	5.8222575	2.7418738	0.0739407
H	5.9577684	3.7824905	-1.8054052
H	5.3769352	1.6533203	1.8841962
H	6.8750503	2.8770447	0.3090067
C	0.3447762	4.8204348	-0.8405265
C	1.1544016	5.2027275	0.2524026
C	-0.4757442	5.8164526	-1.4402421
C	1.1693620	6.5028047	0.7475776
H	1.7899767	4.4499244	0.7121253
C	-0.4494414	7.1221166	-0.9385873
C	0.3606326	7.4688176	0.1451154
H	1.8029688	6.7652801	1.5906253
H	-1.0763639	7.8815261	-1.4013849
H	0.3589884	8.4908454	0.5156221
C	-1.7374899	-1.8334094	-1.5337479
H	-0.7132705	-2.0871848	-1.2359230
H	-2.2393612	-2.7515682	-1.8499906
H	-1.6668823	-1.1674882	-2.4023169
C	3.0465679	-0.6362624	-2.4900890
H	3.5098271	0.3308200	-2.2590740
H	3.7806656	-1.2482065	-3.0209024
H	2.2087107	-0.4453241	-3.1709866
C	3.4301186	3.6608430	-2.7454729
H	2.6064344	4.3617433	-2.5636226
H	4.2197967	4.1869424	-3.2901862
H	3.0354104	2.8691617	-3.3941313
C	-1.3747838	5.5014469	-2.6127163
H	-0.7986694	5.1483194	-3.4750450
H	-2.0887857	4.7088614	-2.3633065
H	-1.9360316	6.3899775	-2.9162865

Cd0 : isomer of cyclic triborane(5) **Cd**
34
Energy = -618.6541711489

B	-0.7691246	1.5331635	-0.2288510
C	-1.7453325	0.3267112	-0.2129740
C	-1.3462397	-0.8987798	-0.7788916
C	-2.1972301	-2.0016325	-0.8045917
C	-3.4717000	-1.8987192	-0.2452734
C	-3.8827493	-0.6933490	0.3264717
C	-3.0429092	0.4258252	0.3471148
C	-3.5219595	1.7140655	0.9697211
H	-3.5627045	2.5206704	0.2261597
H	-2.8477460	2.0462923	1.7679633
H	-4.5231328	1.5960047	1.3931068
H	-4.8760078	-0.6166553	0.7633918
H	-4.1443321	-2.7523346	-0.2519504
H	-1.8670727	-2.9350440	-1.2521664
H	-0.3500334	-0.9825724	-1.2040492
H	1.4199992	2.6537192	-0.1705487
H	-1.3417148	2.6254236	-0.3905328
B	0.0377241	2.9084148	-0.3130483
C	1.8215460	0.3827982	0.2334950
C	1.4301839	-0.6478992	1.1084729
C	2.2803348	-1.7096986	1.4104205
C	3.5442942	-1.7664059	0.8214332
C	3.9468843	-0.7569674	-0.0547972
C	3.1089270	0.3229759	-0.3546122
C	3.5770522	1.3987712	-1.3033763
H	2.8882829	1.5113727	-2.1490368

H	4.5703981	1.1684866	-1.6981161
H	3.6307544	2.3729249	-0.8002200
H	4.9311342	-0.8058377	-0.5155196
H	4.2153893	-2.5924772	1.0412993
H	1.9578635	-2.4886461	2.0958814
H	0.4414584	-0.6106437	1.5572622
B	0.8471491	1.5563784	-0.0544692
H	0.0392440	4.0832411	-0.4636585

Cd : cyclic triborane(5) **Cd** H_3B_3(*o*-tolyl)$_2$
34
Energy = -618.6545025816

B	-0.8162574	0.7018234	-0.5766998
C	-2.1190937	-0.0231945	-0.1514546
C	-2.1515178	-1.4318670	-0.1248181
C	-3.2758108	-2.1302522	0.3076656
C	-4.4031762	-1.4196758	0.7228531
C	-4.3924157	-0.0236094	0.7021738
C	-3.2700179	0.6907527	0.2714318
C	-3.2975324	2.1992155	0.2677179
H	-3.2382510	2.5965329	-0.7535351
H	-2.4474746	2.6131188	0.8228345
H	-4.2205584	2.5717250	0.7195564
H	-5.2728439	0.5248653	1.0292220
H	-5.2885046	-1.9488447	1.0645282
H	-3.2734858	-3.2163804	0.3215834
H	-1.2762366	-1.9886057	-0.4530682
H	-0.0317270	-0.1979695	-1.1337035
H	-1.0991909	1.8027547	-1.2631288
B	0.1039373	2.0758102	-0.8065132
C	2.1526186	-0.0800825	-0.3860105
C	2.1833463	-1.4894328	-0.4465902
C	3.3297657	-2.2163719	-0.1354347
C	4.4824620	-1.5364673	0.2610996
C	4.4759152	-0.1419373	0.3336824
C	3.3343664	0.6001799	0.0135574
C	3.3664759	2.1043139	0.1147797
H	3.0274897	2.5671695	-0.8186505
H	4.3762309	2.4585728	0.3402216
H	2.6947437	2.4608021	0.9052692
H	5.3780056	0.3831006	0.6400282
H	5.3844192	-2.0877474	0.5130479
H	3.3245296	-3.3008762	-0.1989661
H	1.2863256	-2.0250537	-0.7509580
B	0.8736900	0.7018363	-0.7554167
H	0.1242611	3.2355159	-0.5775176

C·THF : THF adduct of diborane(4) **C**
45
Energy = -825.8022365343

B	0.1031153	-0.3141289	-0.2463428
C	-0.0876133	-1.8403958	-0.5455503
C	0.1408141	-2.3064475	-1.8552836
C	0.0489017	-3.6592608	-2.1876327
C	-0.3199068	-4.5794313	-1.2059673
C	-0.5813279	-4.1383255	0.0941563
C	-0.4608300	-2.7884850	0.4413819
C	-0.7759668	-2.3384145	1.8458785
H	-1.6758230	-1.7121759	1.8600258
H	0.0362476	-1.7215154	2.2485982
H	-0.9362429	-3.1938199	2.5094962
H	-0.8816329	-4.8577937	0.8539966
H	-0.4120749	-5.6350568	-1.4494268
H	0.2444519	-3.9896901	-3.2048060
H	0.3969994	-1.5818106	-2.6256064
B	-0.6977767	0.8929368	-1.0770894
C	-1.9164321	0.7093160	-0.0295588
C	-1.8211694	1.2628991	1.2661357
C	-2.8045061	1.0700797	2.2364873
C	-3.9166703	0.2844353	1.9365642
C	-4.0273921	-0.2937086	0.6687716
C	-3.0494944	-0.1030889	-0.3125488
C	-3.2160971	-0.7586109	-1.6590096
H	-3.1446800	-0.0187829	-2.4647970
H	-4.1822467	-1.2680799	-1.7280593
H	-2.4212979	-1.4921186	-1.8361877
H	-4.8951050	-0.9081611	0.4349590
H	-4.6905389	0.1155848	2.6811575
H	-2.6970472	1.5190467	3.2208849
H	-0.9490265	1.8648956	1.5096896
H	-0.9920102	0.8511849	-2.2506728
O	-0.0606057	2.3334344	-0.8229243
C	-0.8743912	3.5227786	-1.2025706
C	0.1014562	4.4516160	-1.9151082
H	-0.1310952	5.5010954	-1.7225647
C	1.4709224	4.0301452	-1.3530110
H	2.3004036	4.3257392	-1.9990947
H	1.6315281	4.4538537	-0.3566831
C	1.3340538	2.5225397	-1.2721613
H	1.4369710	2.0344139	-2.2460398
H	1.9580217	2.0270173	-0.5297946
H	0.0700010	4.2802237	-2.9952470
H	-1.6917317	3.1558638	-1.8236729
H	-1.2592290	3.9289597	-0.2662423
H	0.7208566	-0.0260761	0.7542109

C : diborane(4) H(*o*-tolyl)BB(*o*-tolyl)H
32
Energy = -593.1802661349

B	-0.3729601	-0.7442243	-1.2921366
C	-0.0228862	-1.9290031	-0.3544457
B	0.3697208	0.7444433	-1.2927631
C	-0.5859940	-3.2324136	-0.4823217
C	0.8884734	-1.7039160	0.7045291
C	0.0211146	1.9294358	-0.3548067
C	-0.2234380	-4.2261974	0.4325445
C	1.2390316	-2.6987489	1.6121206
H	1.3188753	-0.7104739	0.8150286
C	-0.8906977	1.7053203	0.7039796
C	0.5864971	3.2319389	-0.4819079
C	0.6760538	-3.9683858	1.4703463
H	-0.6523404	-5.2210143	0.3344933
H	1.9398743	-2.4933568	2.4164081
C	-1.2394682	2.7002001	1.6122093
H	-1.3229228	0.7125993	0.8138143
C	0.2257359	4.2257850	0.4335882
H	0.9351056	-4.7615649	2.1670908
H	-1.9395811	2.4951587	2.4172182
C	-0.6750114	3.9692013	1.4706159
H	0.6577067	5.2194346	0.3371741
H	-0.9345771	4.7631017	2.1663530
C	1.5623290	3.5751164	-1.5821861
H	1.1108247	3.4405231	-2.5707896
H	1.8893306	4.6149255	-1.4922696
H	2.4463353	2.9298789	-1.5444304
C	-1.5610172	-3.5767115	-1.5829664
H	-1.1095900	-3.4406556	-2.5714042
H	-1.8860867	-4.6171874	-1.4937624
H	-2.4462340	-2.9331465	-1.5449602
H	-1.2373455	-0.9203677	-2.1157899
H	1.2331413	0.9203051	-2.1174860

D0 : the lowest tetraborane(8) H_4B_4(*o*-tolyl)$_4$
64
Energy = -1186.450087372

B	-0.3705151	-0.2979937	-0.3827951
B	-0.0145678	1.4667847	-0.0849648
B	1.1102573	-1.1419522	-0.0036149
H	0.6223954	-0.9118144	-1.1873662
B	0.6659599	0.1534627	0.9002830
H	1.8618424	-0.0970155	0.3320668
C	-1.7412481	-1.0431968	-0.3030804
C	-2.8156204	-0.8068369	-1.1936453
C	-1.9091212	-1.9959443	0.7192299
C	-4.0063803	-1.5207616	-1.0268413
C	-3.1009317	-2.6992726	0.8777100
H	-1.0836548	-2.1804975	1.4028590
H	-4.8318621	-1.3390175	-1.7115434
C	-4.1553095	-2.4584261	-0.0030558
H	-3.2064012	-3.4264380	1.6780108
H	-5.0922478	-2.9989902	0.1027157
C	1.7720070	-2.5292509	0.1386084
C	1.4441157	-3.6325547	-0.6902114
C	2.7328411	-2.7080935	1.1553870
C	2.0708565	-4.8622437	-0.4596955
C	3.3523495	-3.9358057	1.3672593
H	2.9918252	-1.8646806	1.7917060
H	1.8149750	-5.7098611	-1.0913632
C	3.0158337	-5.0206237	0.5547284
H	4.0892844	-4.0473088	2.1575790
H	3.4897480	-5.9865590	0.7069351
C	-1.3357133	2.2121848	0.4072829
C	-1.9115034	3.2996439	-0.2964854
C	-1.9824144	1.7748547	1.5764913
C	-3.0806756	3.8970844	0.1919550
C	-3.1437800	2.3756286	2.0589514
H	-1.5602817	0.9360099	2.1244840
C	-3.6969738	3.4481134	1.3604111
H	-3.5185724	4.7264502	-0.3604476
H	-3.6143309	2.0076688	2.9671313
H	-4.6040601	3.9299527	1.7166681
C	1.2297350	2.2537918	-0.7115686
C	1.8613698	3.2859941	0.0215018
C	1.7411271	1.9414543	-1.9827945
C	2.9539276	3.9615473	-0.5377399
C	2.8286807	2.6176176	-2.5321972
H	1.2701027	1.1525563	-2.5683003
C	3.4412568	3.6392181	-1.8044745
H	3.4325865	4.7523895	0.0369498
H	3.1917301	2.3525035	-3.5219927
H	4.2910949	4.1771566	-2.2163010
C	-1.3057473	3.8361310	-1.5728327
H	-0.3680479	4.3681629	-1.3761924
H	-1.0625595	3.0325068	-2.2760373
H	-1.9970724	4.5276858	-2.0639188
C	-2.7072192	0.2074391	-2.3046454
H	-2.6009555	1.2178462	-1.8920218
H	-1.8372607	0.0180106	-2.9441837
H	-3.6002681	0.1909534	-2.9351043
C	0.4319757	-3.5129089	-1.8024776
H	-0.5590416	-3.2534127	-1.4126743
H	0.7144482	-2.7290372	-2.5165189

H	0.3485697	-4.4538925	-2.3523023
C	1.3731848	3.6748820	1.3960967
H	0.4102954	4.1968739	1.3411367
H	1.2132667	2.7913251	2.0242466
H	2.0920904	4.3330933	1.8931630
H	-0.3979770	0.7305427	-1.1907890
H	0.7332736	0.1736019	2.0826849

Da : low-lying tetraborane(8) H_4B_4(*o*-tolyl)$_4$
64
Energy = -1186.441078330

B	-0.4140241	-0.4082941	-0.2925347
B	-0.1666039	1.3241444	-0.5020644
B	1.0432906	-1.1928301	-0.2494782
H	0.3078218	-0.9390359	-1.3128620
B	0.8822554	0.2647006	0.6475962
H	1.9319297	-0.1860651	-0.0275519
C	-1.7415721	-1.1587576	0.0818844
C	-2.9524166	-1.0008460	-0.6307911
C	-1.7456027	-1.9688740	1.2335796
C	-4.1098484	-1.6382887	-0.1696292
C	-2.9052560	-2.5924309	1.6907672
H	-0.8182116	-2.0984689	1.7869836
H	-5.0394356	-1.5092989	-0.7203153
C	-4.0953529	-2.4261498	0.9821304
H	-2.8795404	-3.2040725	2.5888420
H	-5.0091431	-2.9062849	1.3227859
C	1.7681621	-2.5410109	-0.1560063
C	1.2722984	-3.7075322	-0.7966855
C	2.9351517	-2.6381919	0.6310931
C	1.9470479	-4.9171047	-0.6081256
C	3.5893317	-3.8521496	0.8144519
H	3.3296997	-1.7434026	1.1076920
H	1.5700655	-5.8127933	-1.0960515
C	3.0898958	-4.9970072	0.1901324
H	4.4824881	-3.9055806	1.4300572
H	3.5928809	-5.9516853	0.3178219
C	0.5358496	1.7433983	-1.8886290
C	1.4112456	2.8569490	-1.9513760
C	0.2978377	1.0405621	-3.0805923
C	1.9992369	3.2140940	-3.1709040
C	0.8763476	1.4066496	-4.2965604
H	-0.3785199	0.1894440	-3.0638944
C	1.7359089	2.5028510	-4.3422300
H	2.6711034	4.0698319	-3.2020021
H	0.6583491	0.8393051	-5.1978471
H	2.1982831	2.8038972	-5.2787826
C	-1.2612255	2.2281045	0.2369995
C	-2.0037417	3.2735266	-0.3708634
C	-1.5707734	1.9169413	1.5767309
C	-2.9770076	3.9532814	0.3777003
C	-2.5270495	2.6066840	2.3166067
H	-1.0503277	1.0907809	2.0562811
C	-3.2396340	3.6406949	1.7099636
H	-3.5432347	4.7474345	-0.1056559
H	-2.7254637	2.3281009	3.3484818
H	-4.0010712	4.1876823	2.2602266
C	1.7250459	3.6739957	-0.7201536
H	0.8155505	3.9138780	-0.1579302
H	2.3888731	3.1285910	-0.0352906
H	2.2269307	4.6078169	-0.9891578
C	-3.0059253	-0.1696325	-1.8853392
H	-2.4556619	0.7670249	-1.7493118
H	-2.5463670	-0.6984818	-2.7299971
H	-4.0376303	0.0665259	-2.1615600
C	0.0423634	-3.6629758	-1.6668773
H	-0.8315994	-3.3263571	-1.0972487
H	0.1719590	-2.9639497	-2.5026130
H	-0.1737733	-4.6497972	-2.0837544
C	-1.8186520	3.6992922	-1.8104258
H	-0.8611497	4.2080316	-1.9651126
H	-1.8287387	2.8471005	-2.4960273
H	-2.6181804	4.3877190	-2.1010066
H	0.9869333	1.4915356	0.2981195
H	1.0444293	0.2167933	1.8252346

D : H/H bridged BB/BB dimer of **C**
64
Energy = -1186.449650782

B	-0.0119946	0.8421127	0.9838325
B	1.4153078	-0.0746326	1.6144915
B	-1.4115565	0.0731550	1.6399772
H	-0.8276449	1.1967311	2.0117356
B	0.0131762	-0.8351169	0.9768687
H	1.2485220	0.6733080	2.5493484
H	-1.2593962	-0.6340723	2.6051082
H	0.8231358	-1.1854047	2.0099348
C	2.8847397	-0.2414945	1.0885008
C	3.3382046	-1.4296903	0.4880826
C	3.7839307	0.8543965	1.1566897
H	2.6646789	-2.2790154	0.4220875
C	4.6205208	-1.5490855	-0.0443557
C	5.0670363	0.7260396	0.6106668
H	4.9356991	-2.4825978	-0.5034185
C	5.4916283	-0.4608917	0.0124788
H	5.7456546	1.5753973	0.6585276

H	6.4943059	-0.5344234	-0.4005838
C	-2.8764392	0.2361150	1.0937612
C	-3.3306456	1.4180107	0.4822063
C	-3.7711728	-0.8635303	1.1593499
H	-2.6615831	2.2709404	0.4177398
C	-4.6082082	1.5276689	-0.0640478
C	-5.0493759	-0.7454408	0.5995726
H	-4.9234022	2.4571050	-0.5313962
C	-5.4742076	0.4353745	-0.0103969
H	-5.7237310	-1.5983536	0.6455866
H	-6.4728425	0.5010770	-0.4344827
C	0.1573386	2.0631341	0.0447738
C	0.8915517	2.0440593	-1.1691128
C	-0.3597688	3.3022013	0.4911817
C	1.0566180	3.2393235	-1.8810692
C	-0.1736898	4.4818868	-0.2183158
H	1.6081294	3.2181296	-2.8187493
C	0.5380111	4.4472371	-1.4200963
H	-0.5778468	5.4173916	0.1576545
H	0.6889988	5.3571018	-1.9947344
C	-0.1644705	-2.0563519	0.0389290
C	-0.9028944	-2.0346981	-1.1720684
C	0.3502081	-3.2973190	0.4825250
C	-1.0758075	-3.2290649	-1.8836594
C	0.1572961	-4.4760713	-0.2269730
H	-1.6318394	-3.2054223	-2.8186695
C	-0.5594877	-4.4390716	-1.4256112
H	0.5601053	-5.4129802	0.1470782
H	-0.7162623	-5.3483313	-1.9996677
H	0.8961399	-3.3333043	1.4229404
H	-0.9026093	3.3354105	1.4334529
C	-3.3707943	-2.1725402	1.7978525
H	-2.5255463	-2.6298981	1.2716330
H	-3.0599233	-2.0289868	2.8393961
H	-4.2054378	-2.8793257	1.7827338
C	3.3838048	2.1698502	1.7819209
H	2.5493380	2.6301599	1.2410726
H	3.0576739	2.0350715	2.8199360
H	4.2234545	2.8707119	1.7741738
C	1.5122088	0.7923902	-1.7403099
H	2.6052328	0.8649991	-1.7156587
H	1.2280957	-0.1057384	-1.1930997
H	1.2141540	0.6632234	-2.7866790
C	-1.5180399	-0.7792006	-1.7406887
H	-2.6110682	-0.8542201	-1.7355857
H	-1.2451754	0.1135448	-1.1789322
H	-1.2020382	-0.6369841	-2.7802252

E : BB/HBH adduct of **1** and $BH_3 \cdot SMe_2$
71
Energy = -1639.076217510

B	0.3049035	-0.9189627	0.2770388
B	-0.5425567	0.7143273	-0.0349978
C	0.3976764	-1.4697931	1.7940670
C	1.6004350	-1.2491510	-0.6410202
C	-1.3572874	1.4586371	1.1441661
C	0.2471809	1.6338215	-1.1024365
C	1.4412141	-1.0952338	2.6769267
C	-0.5509311	-2.3861124	2.2842429
C	1.8554302	-2.5672409	-1.0999012
C	2.5782601	-0.2822462	-0.9330326
C	-1.0189031	1.3094523	2.4977550
C	-2.4196500	2.3491446	0.8405453
C	0.8642289	2.8686595	-0.7836337
C	0.3352127	1.2083195	-2.4404217
C	1.4888374	-1.6262512	3.9726221
C	-0.4995314	-2.9133132	3.5740212
H	-1.3710876	-2.6919938	1.6372068
C	3.0138575	-2.8482853	-1.8389550
C	3.7338877	-0.5656500	-1.6581561
H	2.4316946	0.7326780	-0.5834646
C	-1.6669371	2.0063126	3.5179782
H	-0.2197948	0.6301220	2.7672452
C	-3.0712341	3.0444071	1.8676907
C	1.5115512	3.6000734	-1.7922345
C	0.9680149	1.9405774	-3.4392057
H	-0.0686036	0.2333278	-2.7080842
C	0.5327462	-2.5324611	4.4302230
H	2.2903757	-1.3099975	4.6381873
H	-1.2611343	-3.6147453	3.9063284
C	3.9524958	-1.8595819	-2.1279951
H	3.1834195	-3.8675173	-2.1823415
H	4.4559355	0.2228284	-1.8564632
H	-1.3619897	1.8625218	4.5521353
C	-2.7017975	2.8852549	3.2036481
H	-3.8787808	3.7284026	1.6113907
C	1.5643314	3.1583106	-3.1124222
H	1.9915395	4.5399177	-1.5245147
H	1.0097750	1.5574094	-4.4556106
H	0.5914987	-2.9287892	5.4407734
H	4.8448427	-2.1010670	-2.7005159
H	-3.2167703	3.4405986	3.9839418
H	2.0733166	3.7494953	-3.8693167
B	-1.3728254	-0.8457430	-0.4361459
H	-0.5220641	-1.8410405	-0.1640038
H	-2.3313317	-1.0765301	0.2328513

H	-1.5429767	0.4168319	-0.8482333
C	0.8922006	3.4392832	0.6163880
H	-0.0869477	3.8272093	0.9168827
H	1.1623394	2.6819885	1.3576415
H	1.6188364	4.2560701	0.6740566
C	0.9126329	-3.7142367	-0.8151834
H	0.5603258	-3.7036135	0.2214569
H	0.0297657	-3.6804210	-1.4682304
H	1.4039325	-4.6735870	-1.0034174
C	2.4874382	-0.0866697	2.2750323
H	3.2315702	-0.5248258	1.6009874
H	2.0335591	0.7518842	1.7358071
H	3.0067693	0.3065355	3.1545944
C	-2.8849295	2.5732756	-0.5793036
H	-3.4177424	1.6930859	-0.9630608
H	-2.0440995	2.7619436	-1.2549959
H	-3.5752533	3.4205061	-0.6330742
S	-1.7298921	-1.6098629	-2.2566783
C	-2.9499889	-0.4567147	-2.9389499
H	-2.4233547	0.4772029	-3.1428308
H	-3.3334049	-0.8840896	-3.8659650
H	-3.7509922	-0.3021286	-2.2148072
C	-2.8075938	-3.0015884	-1.8136779
H	-3.1508796	-3.4712101	-2.7360585
H	-2.2037423	-3.7045606	-1.2382279
H	-3.6410821	-2.6334357	-1.2142928

F : SMe_2 adduct of **B** $H_2BB(\textit{o}\text{-tolyl})_2$
41
Energy = -1071.326906388

B	0.2351234	1.0480579	0.3687197
C	-1.0765923	0.3150235	-0.0934706
C	1.5952303	0.2687344	0.2454775
C	-1.9329789	0.9782696	-0.9960076
C	-1.4292574	-1.0012519	0.3085429
C	2.5810100	0.1822856	1.2577071
C	1.9053557	-0.2588776	-1.0268649
C	-3.0708653	0.3684353	-1.5264525
H	-1.6847695	1.9933991	-1.2949750
C	-2.5937347	-1.5880970	-0.1973405
C	3.8296203	-0.3776852	0.9619862
C	3.1593826	-0.7933777	-1.3182485
H	1.1466632	-0.2267662	-1.8067611
H	-3.6975968	0.9016895	-2.2367933
C	-3.4062237	-0.9219179	-1.1187633
H	-2.8669106	-2.5891995	0.1311579
C	4.1306937	-0.8489317	-0.3164941
H	4.5796226	-0.4445925	1.7482111
H	3.3745782	-1.1724589	-2.3141147
H	-4.2983287	-1.4075851	-1.5060352
H	5.1120177	-1.2671455	-0.5251128
B	0.1381763	2.6902204	0.8104772
H	-0.4980705	3.3843477	0.0405049
H	-0.2505903	2.8396030	1.9551136
S	1.9174599	3.5067459	0.8460931
C	2.4260253	3.4810452	-0.8914650
H	2.6244378	2.4374477	-1.1403295
H	1.6161679	3.8738789	-1.5097135
H	3.3331453	4.0768999	-1.0037306
C	1.5843332	5.2764994	1.0515661
H	0.8373091	5.5894995	0.3193583
H	1.1981396	5.4077829	2.0636923
H	2.5161632	5.8321057	0.9306049
C	-0.5894401	-1.7758706	1.2991501
H	0.3826878	-2.0396511	0.8688375
H	-0.3898729	-1.1891536	2.2022938
H	-1.0966478	-2.6985493	1.5969390
C	2.2706564	0.5997110	2.6747446
H	3.1456452	1.0365307	3.1663344
H	1.4496676	1.3217847	2.7043872
H	1.9595239	-0.2706754	3.2678655

G : BH/BB adduct of $HB(\textit{o}\text{-tolyl})_2$ and **F**
71
Energy = -1639.104434124

B	-0.3022274	-0.9308030	-0.0801123
C	0.5949483	-2.2107228	0.3106926
C	0.7521535	-3.1782246	-0.7059518
C	1.4900449	-4.3445833	-0.5383692
C	2.0950619	-4.5962233	0.6948909
C	1.9136139	-3.6879854	1.7320417
C	1.1629181	-2.5095240	1.5728334
C	0.9750935	-1.6689189	2.8200057
H	0.4810366	-0.7162445	2.6262627
H	1.9375069	-1.4589195	3.2998922
H	0.3663826	-2.2152291	3.5507286
H	2.3571893	-3.8936102	2.7048211
H	2.6846719	-5.4957813	0.8511127
H	1.5901299	-5.0514451	-1.3582398
H	0.2634976	-3.0030115	-1.6614569
C	-1.8522470	-1.2924821	-0.3518990
C	-2.3097460	-1.6486982	-1.6324494
C	-3.6357744	-1.9931732	-1.8951047
C	-4.5657313	-1.9909369	-0.8585174
C	-4.1375583	-1.6627947	0.4268478
C	-2.8046116	-1.3303436	0.6965433

C	-2.4223490	-1.0208988	2.1210160
H	-1.6611320	-1.7179766	2.4851230
H	-2.0153275	-0.0089057	2.2111449
H	-3.2917993	-1.0922867	2.7813074
H	-4.8517095	-1.6653036	1.2484577
H	-5.6068285	-2.2439164	-1.0432619
H	-3.9375149	-2.2512447	-2.9075521
H	-1.6140617	-1.6406748	-2.4665900
H	-0.2126826	-0.0987210	0.9372171
B	0.0350116	0.8323853	0.0041007
C	1.3873391	1.5587643	0.4659396
C	2.3777041	0.8391284	1.1582441
C	3.5856743	1.4075446	1.5611644
C	3.8392352	2.7460710	1.2689263
C	2.8867841	3.4779414	0.5588321
C	1.6737287	2.9117848	0.1423284
C	0.7502391	3.7702910	-0.6897464
H	0.3980001	3.2334583	-1.5762052
H	1.2716209	4.6766334	-1.0128327
H	-0.1442660	4.0673474	-0.1324016
H	3.0941800	4.5145676	0.3005011
H	4.7729059	3.2131443	1.5710173
H	4.3215015	0.8078723	2.0906563
H	2.2070923	-0.2104308	1.3651191
C	-1.3247776	1.6758232	-0.0510357
C	-2.1375089	1.7070074	-1.1938221
C	-3.3471390	2.3968055	-1.2314404
C	-3.7833835	3.0783382	-0.0954516
C	-2.9957290	3.0615160	1.0548371
C	-1.7724601	2.3791284	1.0930163
C	-0.9512672	2.4481073	2.3618625
H	-1.5699892	2.7697506	3.2052448
H	-0.1226480	3.1591410	2.2569874
H	-0.4970543	1.4844090	2.6168210
H	-3.3338084	3.5875101	1.9461164
H	-4.7302021	3.6129316	-0.1006998
H	-3.9520296	2.3893957	-2.1348378
H	-1.8169271	1.1624718	-2.0773468
B	0.5317603	-0.0710125	-1.5378248
H	0.0611281	-0.9182399	-2.2397470
H	0.3975934	0.9873094	-2.0959166
S	2.4567170	-0.4998691	-1.5968365
C	3.2791282	1.0106687	-2.1740724
H	4.2779298	0.7288225	-2.5117040
H	2.7013911	1.4557176	-2.9857076
H	3.3432008	1.6901123	-1.3250959
C	2.5418860	-1.4474354	-3.1438018
H	2.0202604	-0.9061102	-3.9347832
H	2.0645520	-2.4085582	-2.9569444
H	3.5947068	-1.5913846	-3.3927662

H_2 : dihydrogen gas
2
Energy = -1.180112045801

H	0.0278455	-0.0000098	0.0000707
H	0.7721545	0.0000098	-0.0000707

HBcat : Catecholborane $HBO_2C_2Me_4$
14
Energy = -407.2796753878

B	0.0000554	2.3631998	0.0000006
H	-0.0000481	3.5440749	0.0000062
O	0.0000749	1.5817805	1.1521414
O	0.0001920	1.5817803	-1.1521437
C	0.0001079	0.2667091	0.6967232
C	0.0001942	0.2667111	-0.6967286
C	0.0000028	-0.9036406	-1.4353653
C	-0.0001285	-2.0983648	-0.7003008
C	-0.0000246	-2.0983653	0.7002982
C	0.0000087	-0.9036376	1.4353634
H	-0.0000068	-0.8956737	-2.5201064
H	-0.0002132	-3.0445989	-1.2324994
H	0.0000454	-3.0445940	1.2325062
H	-0.0001313	-0.8956770	2.5201048

$HOBAr_2$: $HOB(\textit{o}\text{-tolyl})_2$ (Ar = *o*-tolyl)
31
Energy = -643.0710992343

B	-0.0973158	0.3577273	0.9630263
C	1.3486736	0.1304383	0.3956510
C	-1.3761343	0.0105413	0.1102632
C	2.4932742	-0.1554786	1.1869614
C	1.5215036	0.2504219	-0.9983621
C	-2.5452439	0.8112082	0.0679459
C	-1.3697390	-1.1930433	-0.6223653
C	2.4232732	-0.3183861	2.6877369
C	3.7348463	-0.3113771	0.5585179
C	2.7669954	0.1180401	-1.6101248
H	0.6507715	0.4612109	-1.6144070
C	-2.6460905	2.1293052	0.8056131
C	-3.6403992	0.3869464	-0.6946102
C	-2.4756213	-1.6209226	-1.3551032
H	-0.4727702	-1.8080494	-0.6101037
H	2.2507260	0.6436192	3.1819127
H	3.3597295	-0.7371684	3.0679999
H	1.5994022	-0.9748286	2.9846263

H	4.6057616	-0.5491042	1.1658813
C	3.8810037	-0.1703224	-0.8227910
H	2.8653337	0.2300917	-2.6864122
H	-3.4123063	2.7647595	0.3520147
H	-1.6986206	2.6764673	0.8007163
H	-2.9394996	1.9826338	1.8546599
H	-4.5257442	1.0175635	-0.7439914
C	-3.6175122	-0.8205472	-1.3936865
H	-2.4424952	-2.5604232	-1.8999029
H	4.8602516	-0.2919665	-1.2786194
H	-4.4843374	-1.1273220	-1.9730620
O	-0.2140317	0.8661242	2.2367915
H	-1.1370081	0.9630353	2.5224316

I·1R : B..HB adduct of **I** and $BH_3{\cdot}SMe_2$
41
Energy = -1305.574762761

B	0.1720658	0.3762256	-0.5004667
H	1.2339330	-0.1282507	-0.7697792
B	-0.6965137	-1.9485823	-0.7023670
B	-0.6342927	1.3187081	-1.6957397
H	-0.0081366	-2.1628980	-1.6591788
H	-0.0419789	2.3641393	-1.9153270
H	-1.8265825	1.4957714	-1.5187630
S	-0.6097822	0.3781118	-3.4044659
C	-1.0449975	1.6206748	-4.6531176
C	1.1516383	0.1801537	-3.7691044
H	-2.0906483	1.8825602	-4.4823674
H	-0.4106540	2.4996611	-4.5201421
H	-0.9245812	1.1919299	-5.6501945
H	1.6501963	1.1459767	-3.6617490
H	1.5408531	-0.5249000	-3.0342371
H	1.2667202	-0.2155944	-4.7796283
C	0.0545040	0.9362778	0.9917934
C	1.1703327	1.5000522	1.6615252
C	-1.1767724	0.9444492	1.6755299
C	1.0259841	2.0063871	2.9610878
C	-1.3149155	1.4409653	2.9714941
H	-2.0520934	0.5367684	1.1712118
C	-0.2013159	1.9737379	3.6245532
H	1.8950038	2.4314535	3.4612830
H	-2.2824467	1.4158176	3.4677339
H	-0.2874245	2.3651747	4.6351298
C	2.5193262	1.5798241	0.9849003
H	2.4657275	2.1753565	0.0658779
H	3.2631493	2.0333909	1.6474927
H	2.8759016	0.5864288	0.6867213
H	-0.7295888	-0.7582355	-0.3176289
H	-1.8490368	-2.2888173	-0.7926727
S	0.0235780	-3.0236895	0.7339623
C	1.6285452	-2.2991223	1.1576614
H	2.2665025	-2.4144406	0.2809046
H	1.4912592	-1.2436494	1.3952939
H	2.0376203	-2.8572230	2.0011501
C	-0.9486907	-2.5725179	2.1953262
H	-0.8903858	-1.4941741	2.3507588
H	-1.9759863	-2.8791982	1.9937745
H	-0.5526191	-3.1230165	3.0498341

(I)$_{2h}$: BH/BH dimer of **I** H(*o*-tolyl)$BBH_2{\cdot}SMe_2$
56
Energy = -1601.598521669

B	0.4879805	-0.0558135	-0.4001362
B	-1.8753311	1.7375523	0.4335236
B	1.1312251	-1.6177214	-0.7284467
H	-0.6341505	0.1190779	-1.0773076
B	-1.2447784	0.1724094	0.0968391
H	-2.2190687	1.8784258	1.5941858
H	1.2553509	-1.8396221	-1.9208010
H	-0.1243016	0.0019056	0.7753409
C	1.3631286	1.2510952	-0.5940013
C	2.0485772	1.8766184	0.4724146
C	1.5741217	1.7462963	-1.8923869
C	2.9238446	2.9376690	0.2087977
C	2.4381376	2.8109979	-2.1478467
H	3.4527502	3.4050124	1.0378218
C	3.1267884	3.4065496	-1.0900947
H	2.5794783	3.1681361	-3.1652140
H	3.8137564	4.2291557	-1.2723755
C	-2.1248251	-1.1337515	0.2832694
C	-2.6350747	-1.8984232	-0.7909660
C	-2.5029785	-1.4990611	1.5872611
C	-3.4919076	-2.9756893	-0.5303722
C	-3.3428976	-2.5825926	1.8416567
H	-3.8855772	-3.5510260	-1.3667605
C	-3.8474404	-3.3247840	0.7730953
H	-3.6109162	-2.8389478	2.8639528
H	-4.5135300	-4.1655714	0.9504025
H	-2.1357157	-0.9081556	2.4250675
H	1.0590178	1.2736813	-2.7274994
C	1.8416756	1.4139338	1.8960602
H	2.0620904	0.3449130	2.0077408
H	0.7999080	1.5617529	2.2070784
H	2.4846210	1.9672909	2.5870763
C	-2.2826286	-1.5616967	-2.2217215
H	-1.2067535	-1.6782305	-2.3992713

H	-2.8170692	-2.2140023	-2.9189714
H	-2.5398190	-0.5230520	-2.4655044
S	-3.5213125	1.9738216	-0.5916336
S	2.9631364	-1.7080404	-0.0397551
C	-4.7805767	0.9746884	0.2462160
H	-5.7551273	1.2060932	-0.1873310
H	-4.5233015	-0.0712230	0.0767139
H	-4.7569685	1.1976066	1.3147122
C	-4.0889733	3.6308003	-0.1243475
H	-4.1182469	3.7034132	0.9649628
H	-3.3630620	4.3368002	-0.5302226
H	-5.0737925	3.8091980	-0.5605464
C	3.4826214	-3.3936087	-0.4528542
H	2.8809864	-4.0663435	0.1599699
H	3.2917851	-3.5766119	-1.5124496
H	4.5415868	-3.5114149	-0.2148014
C	3.9955330	-0.7800469	-1.2052905
H	3.7546120	-1.0958163	-2.2224139
H	3.7594320	0.2757943	-1.0718659
H	5.0436429	-0.9717579	-0.9682625
H	-1.2089273	2.6636023	0.0204186
H	0.6121373	-2.5401594	-0.1361350

I2a : BH/BB dimer of **I** H(*o*-tolyl)BBH$_2$·SMe$_2$
56
Energy = -1601.596945493

B	0.0158388	-0.0559361	0.8307896
C	0.1169149	0.9862275	-0.4003568
C	1.4280008	1.1126915	-0.9093060
C	1.7505459	1.9203427	-1.9973914
C	0.7463038	2.6700562	-2.6084920
C	-0.5563705	2.5734958	-2.1240006
C	-0.8933383	1.7398821	-1.0462298
C	-2.3504655	1.6585541	-0.6607318
H	-2.9520816	2.2906709	-1.3213046
H	-2.7107662	0.6277659	-0.7416115
H	-2.5274664	1.9772811	0.3720137
H	-1.3484327	3.1437245	-2.6062996
H	0.9706684	3.3177889	-3.4523279
H	2.7752829	1.9680770	-2.3580214
H	2.2210970	0.5369287	-0.4353437
H	0.4424002	-1.2430408	0.2588169
B	1.1958230	0.1636818	2.0988537
H	0.8270903	0.0293394	3.2455574
S	1.7912667	2.0373523	2.0842725
C	0.2850639	3.0044584	2.3548925
H	-0.2961525	2.9310866	1.4344058
H	-0.2740440	2.5877048	3.1947988
H	0.5603883	4.0429373	2.5461484
C	2.5804426	2.2332613	3.7040818
H	3.4803699	1.6160785	3.6890710
H	1.9011162	1.8853873	4.4848626
H	2.8474576	3.2818792	3.8489986
B	-0.7698816	-1.6188268	0.4189438
C	-1.4737279	-1.8983576	-0.9867636
C	-0.9342740	-1.4253448	-2.1963736
C	-1.5360868	-1.6703215	-3.4319999
C	-2.7111871	-2.4186853	-3.4898886
C	-3.2549724	-2.9231046	-2.3071607
C	-2.6544852	-2.6809732	-1.0646325
C	-3.2817550	-3.2728061	0.1755823
H	-4.1140427	-3.9340755	-0.0849917
H	-2.5478498	-3.8474191	0.7530467
H	-3.6606394	-2.4906728	0.8438808
H	-4.1621267	-3.5244620	-2.3465156
H	-3.1959872	-2.6171581	-4.4428306
H	-1.0883237	-1.2787371	-4.3425404
H	-0.0213641	-0.8373857	-2.1695903
H	-0.6034487	-2.5990970	1.1018684
B	-1.6497681	-0.4802264	1.5353000
H	-1.4176389	0.6836541	1.7692494
S	-1.9106276	-1.3396796	3.2883085
C	-0.5233285	-2.3927090	3.7953964
H	0.4199350	-1.8663692	3.6582931
H	-0.5657578	-3.2883439	3.1768517
H	-0.6927062	-2.6525569	4.8422401
C	-1.8001919	-0.0077984	4.5118819
H	-2.5721000	0.7178719	4.2514472
H	-0.8093525	0.4444041	4.4613789
H	-1.9975050	-0.4270486	5.4998862
H	2.2543976	-0.4096275	1.9148050
H	-2.7544542	-0.5570284	1.0820789

(**I**)$_2$: BB/BB dimer of **I** H(*o*-tolyl)BBH$_2$·SMe$_2$
56
Energy = -1601.611106791

B	0.5575874	0.4598136	0.4503236
H	0.2135339	1.5271223	0.8878637
B	-0.5611759	-0.4622483	-0.4540250
B	0.6161454	0.6853305	-1.4324849
B	-0.6169407	-0.6864009	1.4301450
H	-0.2161089	-1.5283647	-0.8934308
H	1.2016892	1.7232687	-1.2639010
H	-0.2098913	0.8128316	-2.2945549
S	1.9241876	-0.5424034	-2.2705433
H	-1.2070477	-1.7202661	1.2518618

H	0.2066673	-0.8256976	2.2924705
S	-1.9216586	0.5426411	2.2692512
C	1.4471116	-0.4169452	-4.0170193
C	3.4967625	0.3550813	-2.3143651
C	-3.4922737	-0.3576553	2.3237035
C	-1.4357387	0.4272615	4.0139099
H	0.4652367	-0.8843688	-4.1061349
H	1.3925488	0.6322015	-4.3128670
H	2.1782994	-0.9601532	-4.6185996
H	3.3291526	1.3698746	-2.6795693
H	3.8698134	0.3716018	-1.2893028
H	4.1862427	-0.1887785	-2.9626528
H	-4.1820240	0.1897573	2.9686861
H	-3.8666604	-0.3828130	1.2992400
H	-3.3219769	-1.3692267	2.6964997
H	-1.3809824	-0.6200414	4.3161017
H	-0.4527805	0.8939614	4.0947063
H	-2.1628954	0.9753920	4.6159023
C	2.0717963	0.0477067	0.7686082
C	3.0541286	1.0074959	1.1174755
C	2.4838804	-1.2942230	0.6746115
C	4.3790863	0.6033119	1.3356748
C	3.8053112	-1.6916434	0.8828308
H	1.7378882	-2.0457649	0.4231673
C	4.7644518	-0.7332107	1.2126172
H	5.1234114	1.3533827	1.5983017
H	4.0838170	-2.7387678	0.7903036
H	5.8000826	-1.0202948	1.3768197
C	-2.0754668	-0.0494927	-0.7705715
C	-3.0586661	-1.0092199	-1.1172421
C	-2.4866328	1.2927717	-0.6777953
C	-4.3833538	-0.6044346	-1.3358647
C	-3.8081267	1.6906057	-0.8851154
H	-1.7395501	2.0444437	-0.4300300
C	-4.7677509	0.7325113	-1.2143370
H	-5.1281277	-1.3542844	-1.5978148
H	-4.0865323	2.7375905	-0.7907924
H	-5.8030391	1.0202239	-1.3795862
C	2.7076146	2.4714357	1.2470411
H	1.9650202	2.6347763	2.0364800
H	3.5989530	3.0622407	1.4808966
H	2.2668300	2.8554164	0.3199029
C	-2.7132880	-2.4737059	-1.2433773
H	-3.6043241	-3.0639639	-1.4796831
H	-1.9678204	-2.6386897	-2.0296888
H	-2.2764159	-2.8569932	-0.3140467

I·THF : THF adduct of **I** H(*o*-tolyl)BBH_2·SMe_2

41

Energy = -1033.392374502

B	0.5222355	-0.0987751	1.1642767
H	0.9491225	-0.4993813	2.2392872
C	-0.6622077	-0.9993796	0.5668425
C	-0.7643801	-1.2732008	-0.8123152
C	-1.7400256	-1.4261492	1.3853087
C	-1.8656209	-1.9232274	-1.3739446
H	0.0366763	-0.9435927	-1.4720306
C	-2.8438421	-2.0751701	0.8189850
C	-2.9200086	-2.3261437	-0.5538632
H	-1.9038389	-2.1053296	-2.4459154
H	-3.6627611	-2.3857036	1.4667067
H	-3.7890951	-2.8274840	-0.9725926
B	0.1419087	1.5998633	1.2184499
H	-0.6474627	1.8998583	2.0988656
H	1.0810977	2.3778834	1.1929596
S	-0.8024718	2.1508555	-0.4247000
C	-2.5345646	1.6777456	-0.1752969
H	-2.5848350	0.5909193	-0.2420733
H	-2.8496316	2.0132614	0.8150459
H	-3.1387045	2.1381616	-0.9595383
C	-0.9713747	3.9486347	-0.2507252
H	-1.3809420	4.1753992	0.7361964
H	0.0296028	4.3716996	-0.3468973
H	-1.6187190	4.3304044	-1.0432194
C	2.3455216	-1.7431997	0.1685293
O	1.8008240	-0.3757693	0.1608557
C	2.9354841	0.5749262	0.1598083
C	4.1661317	-0.2618093	-0.2109985
C	3.5885541	-1.6089823	-0.6858243
H	2.5648235	-2.0107322	1.2079794
H	1.5609530	-2.3845037	-0.2292117
H	2.6770420	1.3383619	-0.5736886
H	2.9936468	1.0214732	1.1525067
H	4.7669174	0.2292156	-0.9794893
H	4.7951661	-0.4139010	0.6703446
H	3.3145849	-1.5691183	-1.7447189
H	4.2822800	-2.4391899	-0.5342125
C	-1.7233443	-1.1528700	2.8697629
H	-0.8423918	-1.6034172	3.3427686
H	-1.6623920	-0.0756514	3.0649499
H	-2.6230155	-1.5465041	3.3539309

I : adduct H(*o*-tolyl)BBH_2·SMe_2

28

Energy = -800.7721628612

B	0.5130308	-0.5437485	-0.0218018

C	1.8420905	0.2768668	0.2053240
C	2.3152359	0.3296985	1.5352861
C	3.4343839	1.0724271	1.9112811
C	4.1469557	1.7713444	0.9366257
C	3.7194449	1.7195242	-0.3914820
C	2.5793818	0.9994099	-0.7712468
C	2.1690146	1.0100461	-2.2217430
H	2.9254209	1.5076760	-2.8362959
H	2.0214708	-0.0063025	-2.6056948
H	1.2156669	1.5335739	-2.3552303
H	4.2803023	2.2559974	-1.1547993
H	5.0305886	2.3458806	1.2027924
H	3.7563235	1.0919472	2.9495947
H	1.7782727	-0.2354550	2.2950970
B	-0.4964089	-0.5459315	-1.2880670
S	-2.0738153	-1.6115117	-1.2135227
C	-2.0196530	-2.7115201	-2.6605443
H	-2.9084886	-3.3448870	-2.6503979
H	-1.1057524	-3.3071177	-2.6287485
H	-2.0258596	-2.0663229	-3.5400632
C	-1.8955403	-2.8158378	0.1237982
H	-2.7410857	-3.5023379	0.0591012
H	-1.9271565	-2.2609131	1.0607484
H	-0.9458861	-3.3432818	0.0254778
H	-0.5741452	0.0954164	-2.2939771
H	0.2942242	-1.3464373	0.8514553
H	0.5415658	-1.3457450	-1.2748940

J0 : triborane(7) B_3H_6(*o*-tolyl)
23
Energy = -349.3344371084

B	0.6708410	0.1328618	0.1640529
H	1.8010454	-0.2082321	0.2800163
B	-0.4295348	-0.6270847	-0.8891840
B	0.1586150	1.1928066	-1.0634778
H	0.0484277	-1.5204319	-1.5247169
H	1.1740926	1.6136918	-1.5343966
H	-0.8559454	1.5986624	-1.5337029
C	0.2028091	1.5659153	0.7544183
C	1.1727383	2.5160185	1.2079905
C	-1.1514506	1.7311972	1.1482967
C	0.7488677	3.5629880	2.0261443
C	-1.5490921	2.7755903	1.9695321
H	-1.8832231	1.0133582	0.7895712
C	-0.5899938	3.6933996	2.4052311
H	1.4762676	4.2909733	2.3755199
H	-2.5883428	2.8814179	2.2643564
H	-0.8848755	4.5215622	3.0440807
C	2.6251596	2.3961120	0.8460094
H	2.7571924	2.2712599	-0.2332660
H	3.1778566	3.2799884	1.1734530
H	3.0655746	1.5134783	1.3251093
H	-0.1492625	-0.8424362	0.4256526
H	-1.6196851	-0.5360175	-0.8594810

J0·THF : THF adduct of **J0**
36
Energy = -581.9637416417

B	0.3370029	2.1465746	0.2248287
C	1.1955740	0.8097920	0.2211353
C	1.4303680	0.1149152	-0.9787117
C	2.1271962	-1.0925474	-1.0165365
C	2.6136413	-1.6390232	0.1718216
C	2.4017647	-0.9644826	1.3750960
C	1.7035105	0.2501731	1.4192214
C	1.4790928	0.9229092	2.7526287
H	1.9312240	0.3419014	3.5618681
H	0.4087615	1.0337676	2.9647020
H	1.9070638	1.9313652	2.7662618
H	2.7814418	-1.3891777	2.3024319
H	3.1554973	-2.5812959	0.1639644
H	2.2871743	-1.6036319	-1.9625639
H	1.0466635	0.5314862	-1.9081598
H	0.5506700	2.9609723	1.0759733
B	-0.6984515	2.7137938	-1.0428180
H	-1.0649251	3.8512584	-0.9666137
H	-0.8914830	2.1462701	-2.0823904
B	-1.4674468	1.6276428	0.2196971
H	-2.5165162	2.1904609	0.0191816
H	-1.3058324	1.5150556	1.4084719
O	-1.6025088	0.1680586	-0.3333286
C	-2.3633933	-0.1502837	-1.5659100
C	-2.9127511	-1.5459613	-1.3081145
H	-3.0003430	-2.1154724	-2.2355305
C	-1.8924151	-2.1543050	-0.3295041
C	-1.5820897	-0.9892924	0.5885745
H	-0.5885511	-0.9979185	1.0342587
H	-2.3524644	-0.8191383	1.3457184
H	-2.2951658	-3.0051788	0.2236983
H	-0.9871590	-2.4696980	-0.8571393
H	-3.8991493	-1.4872582	-0.8386541
H	-3.1173534	0.6280844	-1.6838386
H	-1.6442543	-0.1121787	-2.3852260
H	0.6077381	2.7557109	-0.9347686

J : SMe_2 adduct of triborane(7) B_3H_6(*o*-tolyl)

32
Energy = -827.4783558270

B	0.6127650	0.3103750	0.0667074
H	1.6801317	-0.1990725	0.2902724
B	-0.5682214	-0.4752051	-0.9467986
B	0.6775363	0.5840644	-1.7666437
H	-0.4170350	-1.5643407	-1.4219834
H	1.0911112	1.7051471	-1.6610641
H	-0.1792538	0.5587367	-2.6191348
S	2.1093531	-0.5218739	-2.5103614
C	2.1293727	-0.0226681	-4.2526547
C	3.6394504	0.2615673	-1.9426254
H	1.2147479	-0.4114994	-4.7016961
H	2.1548049	1.0663333	-4.3188140
H	3.0026867	-0.4688899	-4.7303919
H	3.6131451	1.3258109	-2.1805764
H	3.6909317	0.1087901	-0.8648644
H	4.4780717	-0.2335001	-2.4337519
C	0.1941296	1.5608306	0.9600592
C	1.1311168	2.5548652	1.3365218
C	-1.1235712	1.7010669	1.4310766
C	0.7256233	3.6286433	2.1399408
C	-1.5209979	2.7689738	2.2370027
H	-1.8612657	0.9506070	1.1561515
C	-0.5889475	3.7432585	2.5938198
H	1.4570244	4.3858284	2.4162388
H	-2.5489164	2.8379883	2.5839521
H	-0.8775130	4.5796496	3.2250882
C	2.5683449	2.4846185	0.8813095
H	2.6402643	2.5699226	-0.2091882
H	3.1595613	3.2907175	1.3252231
H	3.0252627	1.5270920	1.1559932
H	-0.2246447	-0.6656737	0.3271597
H	-1.6744230	-0.0210140	-0.9404944

Ka : high-lying adduct H_2(*o*-tolyl)BBH$(SMe_2)_2$
37
Energy = -1278.894912513

B	0.5639583	-0.9724824	0.2011600
C	1.7701795	0.0980218	0.3007207
C	2.3191709	0.4598752	1.5479393
C	3.3413944	1.4017403	1.6930688
C	3.8706125	2.0206341	0.5601896
C	3.3692386	1.6686732	-0.6960937
C	2.3435097	0.7251639	-0.8389787
C	1.8707031	0.3612555	-2.2257668
H	2.4127048	0.9266671	-2.9915043
H	2.0127734	-0.7102646	-2.4154597
H	0.7978543	0.5524727	-2.3398724
H	3.7874953	2.1338960	-1.5886356
H	4.6666685	2.7564743	0.6479878
H	3.7254765	1.6457157	2.6819262
H	1.9246151	-0.0251319	2.4416716
B	-0.9519004	-0.2818657	-0.3273209
S	-2.1878886	-1.6264436	-1.0981395
C	-1.3318210	-1.9317651	-2.6639742
H	-1.7859402	-2.8033560	-3.1372712
H	-0.2733144	-2.0874796	-2.4481193
H	-1.4784333	-1.0450629	-3.2817177
C	-1.8250901	-3.1614169	-0.2083273
H	-2.3074432	-3.9855812	-0.7361485
H	-2.2500006	-3.0555778	0.7911792
H	-0.7421222	-3.2785839	-0.1534098
H	-1.0549856	0.6092689	-1.1405621
S	-1.9757130	0.3361695	1.2243840
C	-0.9117289	1.6878784	1.7840198
H	0.0116404	1.2347117	2.1424057
H	-0.6988939	2.3469231	0.9403431
H	-1.4277502	2.2170042	2.5865075
C	-3.3941726	1.2926858	0.6089181
H	-4.0885101	0.5816825	0.1579033
H	-3.8728307	1.7962080	1.4509558
H	-3.0463195	2.0101827	-0.1365128
H	0.4098735	-1.5217179	1.2967352
H	0.8522937	-1.8506704	-0.6232036

K : adduct Me_2S·H(*o*-tolyl)BBH_2·SMe_2
37
Energy = -1278.911604912

B	0.0539778	0.2685943	-0.1955983
C	-1.4612620	-0.2012311	0.0680069
C	-2.3265449	0.4363827	0.9781080
C	-3.6249367	-0.0122894	1.2324182
C	-4.1045131	-1.1435504	0.5732214
C	-3.2667200	-1.8056004	-0.3276903
C	-1.9658671	-1.3567628	-0.5853982
C	-1.1001229	-2.1236891	-1.5543147
H	-0.1861959	-2.4729076	-1.0595030
H	-0.7780516	-1.4882180	-2.3880630
H	-1.6316228	-2.9913704	-1.9582397
H	-3.6294871	-2.6933114	-0.8445654
H	-5.1116920	-1.5093325	0.7574522
H	-4.2552244	0.5192547	1.9422071
H	-1.9762804	1.3170280	1.5165496
B	1.1643978	-0.4998629	0.9214237
H	0.8994759	-1.6668320	1.1518125

H 1.3913619 0.1207748 1.9484987
S 2.9480138 -0.5907927 0.1180325
C 2.7363794 -1.7314001 -1.2700860
H 3.7022502 -1.9012781 -1.7487022
H 2.0445868 -1.2490201 -1.9615487
H 2.3089207 -2.6659233 -0.9004480
C 3.9360035 -1.6354989 1.2237800
H 4.8962216 -1.8580547 0.7538477
H 4.0909469 -1.0649468 2.1410051
H 3.3787387 -2.5500613 1.4389140
H 0.3602830 0.2450800 -1.3773962
S 0.1005733 2.2139883 0.1750488
C 1.7616749 2.6993810 -0.3468560
H 2.4568374 2.3146981 0.3997487
H 1.9760180 2.2620256 -1.3244147
H 1.8151909 3.7889247 -0.3809799
C -0.8744299 2.9504251 -1.1646207
H -0.7961534 4.0383713 -1.1209755
H -1.9074143 2.6389133 -1.0004543
H -0.5133299 2.5624913 -2.1193985

L : BB/BB dimer of H_2BB(*o*-tolyl)H
38
Energy = -645.3573107625
B 0.0966764 0.8349638 1.1027779
B 1.3819635 -0.2792435 1.7525684
B -1.3848192 0.2857353 1.7510591
H -0.5929409 1.2587818 2.1785112
B -0.0992060 -0.8289603 1.1029779
H 1.5128498 0.4306616 2.7139151
H -1.5173956 -0.4304417 2.7076808
H 0.5950364 -1.2593427 2.1731400
C 0.2454273 1.9845752 0.0760088
C 1.0355038 1.8967673 -1.1022739
C -0.4624530 3.1823733 0.3176372
C 1.0697997 2.9880492 -1.9776211
C -0.4063476 4.2639780 -0.5547043
H 1.6658691 2.9144770 -2.8841605
C 0.3641592 4.1626096 -1.7142847
H -0.9596796 5.1730210 -0.3376146
H 0.4168310 4.9935027 -2.4123975
C -0.2483218 -1.9787195 0.0765978
C -1.0426989 -1.8928751 -1.0989000
C 0.4632321 -3.1748376 0.3159118
C -1.0773396 -2.9843028 -1.9740883
C 0.4067706 -4.2565962 -0.5561586
H -1.6756609 -2.9117896 -2.8792289
C -0.3684477 -4.1573343 -1.7128104

H 0.9636320 -5.1640194 -0.3413439
H -0.4223376 -4.9887706 -2.4101832
H 1.0748118 -3.2498904 1.2120451
H -1.0708977 3.2589569 1.2157851
C 1.8300886 0.6623241 -1.4540936
H 2.6517822 0.5092651 -0.7472570
H 1.2151743 -0.2427645 -1.4268358
H 2.2560493 0.7588708 -2.4563283
C -1.8428502 -0.6610943 -1.4477917
H -2.6736647 -0.5218678 -0.7485601
H -1.2360091 0.2486445 -1.4054726
H -2.2577233 -0.7518576 -2.4552339
H 2.3623121 -0.7546797 1.2752901
H -2.3639572 0.7654384 1.2757339

Mt : THF adduct of **L** B_4H_6(*o*-tolyl)$_2$
51
Energy = -877.9814477672
B 0.7079551 -0.5968297 0.1571822
B 1.3632424 0.4496329 -1.0071963
H 2.1416028 1.3407115 -1.1646277
H 0.2319521 0.0568825 1.1733001
H 1.6331813 -1.2503178 0.8047303
B 0.2967409 1.1233000 0.3103014
C -1.1951153 1.6023105 0.0856894
C -2.2970694 0.7338845 0.1905349
C -3.6008130 1.1502010 -0.0823362
C -3.8342469 2.4687535 -0.4748317
C -2.7582677 3.3525799 -0.5765352
C -1.4487483 2.9434778 -0.2973850
C -0.3157755 3.9329956 -0.4311057
H 0.4628994 3.5598273 -1.1078396
H 0.1726338 4.1070518 0.5352551
H -0.6762148 4.8923974 -0.8138762
H -2.9360878 4.3828670 -0.8796165
H -4.8426436 2.8085715 -0.6964484
H -4.4272987 0.4497947 0.0093770
H -2.1283259 -0.2991338 0.4881657
B 2.4238532 -0.8105557 -0.2220947
H 3.2267118 -0.1809703 0.4068346
H 1.0361578 1.8322037 0.9341137
C 2.9081935 -2.0845146 -1.0277849
C 2.1068525 -3.2299502 -1.1870276
C 4.1614407 -2.0624162 -1.6905679
C 2.5030073 -4.3125340 -1.9726209
H 1.1419455 -3.2717280 -0.6863366
C 4.5493308 -3.1489434 -2.4843914
C 3.7319116 -4.2705993 -2.6328367

H	1.8560366	-5.1808214	-2.0709753
H	5.5093786	-3.1123350	-2.9961461
H	4.0531906	-5.1018651	-3.2551655
C	5.0794979	-0.8686996	-1.5715432
H	4.5695124	0.0598648	-1.8566184
H	5.4173329	-0.7297894	-0.5376720
H	5.9600511	-0.9882359	-2.2097616
H	-0.1006165	-1.3466786	-0.2847666
O	0.7350197	0.0610625	-2.3945373
C	-0.3518597	0.9015964	-2.9588939
C	1.6705330	-0.3491748	-3.4887691
C	-0.3951463	0.4689304	-4.4084500
H	-0.0576648	1.9471054	-2.8323773
H	-1.2370669	0.6813312	-2.3661644
C	1.0948629	0.2886039	-4.7448076
H	1.6516920	-1.4385640	-3.4903801
H	2.6588824	0.0118773	-3.2065966
H	-0.9371550	-0.4762068	-4.5087786
H	-0.8782207	1.2202921	-5.0364729
H	1.2563692	-0.3439660	-5.6198357
H	1.5601894	1.2613933	-4.9270509

M : SMe_2 adduct of **L** B_4H_6(*o*-tolyl)$_2$
47
Energy = -1123.504649678

B	0.8209415	-0.5346818	0.2698519
B	1.3134607	0.4232552	-1.0473340
H	2.0282859	1.3275332	-1.3520825
H	0.3988783	0.1994481	1.2550055
H	1.8407186	-1.0644743	0.8757193
B	0.3327200	1.1765225	0.2907454
C	-1.1904782	1.5587636	0.0870265
C	-2.2298879	0.6141869	0.1701702
C	-3.5600218	0.9455498	-0.0896343
C	-3.8854432	2.2530768	-0.4518335
C	-2.8730894	3.2101865	-0.5392596
C	-1.5367995	2.8870577	-0.2715592
C	-0.4779743	3.9565147	-0.4015984
H	0.3016642	3.6595388	-1.1136323
H	0.0257695	4.1355512	0.5553889
H	-0.9161484	4.8987512	-0.7437988
H	-3.1220403	4.2310261	-0.8232803
H	-4.9156430	2.5274169	-0.6636048
H	-4.3356680	0.1876835	-0.0123969
H	-1.9902856	-0.4111195	0.4428019
B	2.5018044	-0.7272093	-0.2810951
H	3.3454083	-0.0199588	0.1900273
H	1.0695874	1.9768093	0.7912057
C	2.9395412	-2.0674140	-1.0020234
C	2.1566312	-3.2361834	-0.9775489
C	4.1294656	-2.0895044	-1.7743896
C	2.5135643	-4.3864964	-1.6817809
H	1.2395518	-3.2448168	-0.3927359
C	4.4765794	-3.2449510	-2.4857990
C	3.6806840	-4.3910550	-2.4463166
H	1.8830832	-5.2710298	-1.6363226
H	5.3867962	-3.2432429	-3.0826813
H	3.9705426	-5.2762908	-3.0064660
C	5.0161656	-0.8700158	-1.8675623
H	4.4536504	0.0052552	-2.2143524
H	5.4317535	-0.6055054	-0.8881674
H	5.8454903	-1.0435109	-2.5595669
H	0.0196452	-1.3702884	0.0040417
S	0.3466667	-0.2115170	-2.5890511
C	-0.3937082	1.2898539	-3.2850101
H	-1.1744313	1.6046700	-2.5918855
H	0.3731144	2.0610084	-3.3775371
H	-0.8210996	1.0391179	-4.2571063
C	1.6401091	-0.5118472	-3.8233185
H	2.1989267	-1.3854145	-3.4864747
H	1.1558854	-0.7108225	-4.7806234
H	2.2910102	0.3620192	-3.8840083

N : diborane(6) (*o*-tolyl)$_2$BH·BH_3
34
Energy = -594.4205638974

B	0.0000608	-0.0000045	1.2484800
B	0.0005435	0.0001544	3.0466643
H	-0.9756100	0.0528224	2.1948756
H	0.9759853	-0.0570528	2.1943655
C	-0.0151954	-1.3944814	0.5131407
C	0.0148421	1.3943614	0.5129502
C	-0.9104685	-1.6652872	-0.5502121
C	0.8920370	-2.3990829	0.8935937
C	0.9099904	1.6653085	-0.5504761
C	-0.8926187	2.3987474	0.8934012
C	-1.9235326	-0.6440173	-1.0128493
C	-0.8603594	-2.9112722	-1.1859483
C	0.9425681	-3.6324450	0.2451724
H	1.5849404	-2.2054222	1.7095857
C	1.9245755	0.6449741	-1.0118395
C	0.8591751	2.9110661	-1.1866098
C	-0.9427972	3.6324433	0.2456148
H	-1.5865470	2.2043315	1.7083452
H	-2.4868626	-0.2231433	-0.1729199
H	-2.6345647	-1.0960600	-1.7101974

H	-1.4354812	0.1969115	-1.5186945
H	-1.5544407	-3.1165141	-1.9982200
C	0.0591924	-3.8890014	-0.8028019
H	1.6597536	-4.3859763	0.5589111
H	2.4879413	0.2252931	-0.1713357
H	2.6354638	1.0974116	-1.7090820
H	1.4378410	-0.1968674	-1.5174264
H	1.5521330	3.1158804	-1.9999507
C	-0.0600387	3.8888898	-0.8028894
H	-1.6588060	4.3865417	0.5606586
H	0.0797695	-4.8458894	-1.3175565
H	-0.0809408	4.8457094	-1.3177596
H	0.0754012	1.0421365	3.6186704
H	-0.0739508	-1.0404659	3.6212086

O : adduct (*o*-tolyl)$_2$BH·SMe$_2$
39
Energy = -1045.871758617

B	0.2630565	1.7242888	0.3716690
H	-0.7607063	2.2517822	-0.0112576
S	0.0702825	0.0152976	-0.6939388
C	1.1814537	-1.2289405	0.0163820
H	1.0306779	-1.2724874	1.0965135
H	0.9614336	-2.1878358	-0.4556964
H	2.2047662	-0.9271615	-0.2088412
C	-1.5053921	-0.6382329	-0.0915915
H	-1.4939251	-0.6558054	1.0003092
H	-2.2891487	0.0237981	-0.4606503
H	-1.6390669	-1.6412396	-0.4996636
C	0.2655823	1.3382721	1.9318732
C	1.4623194	0.8769767	2.5121271
C	-0.8675001	1.4478094	2.7761611
C	1.5544728	0.4993786	3.8517171
H	2.3540592	0.8293349	1.8892312
C	-0.7700266	1.0685524	4.1228528
C	0.4237217	0.5895089	4.6637262
H	2.4984666	0.1454307	4.2591990
H	-1.6468902	1.1613456	4.7615088
H	0.4709708	0.3022839	5.7112788
C	1.5807716	2.4870662	-0.1474495
C	1.8377992	3.7939042	0.3444607
C	2.5099868	1.9547037	-1.0561623
C	2.9759852	4.4921091	-0.0746246
C	3.6478950	2.6527180	-1.4714053
H	2.3466826	0.9637226	-1.4777562
C	3.8860834	3.9319916	-0.9742179
H	3.1496857	5.4960366	0.3094976
H	4.3393150	2.1973099	-2.1763379
H	4.7647954	4.4920275	-1.2841376
C	-2.1768488	2.0239390	2.2849805
H	-2.5878853	1.4629103	1.4405679
H	-2.9202580	2.0263150	3.0881331
H	-2.0421887	3.0548413	1.9377054
C	0.8883092	4.4486486	1.3189224
H	-0.1411175	4.4230243	0.9410957
H	0.8817306	3.9181386	2.2783846
H	1.1672401	5.4912640	1.4997110

(**P**)$_{2h}$: BH/BH dimer of **P**
82
Energy = -2142.692216506

B	0.8918008	0.1626093	-0.0844835
B	-2.1077087	1.0454658	0.3184252
B	2.1089524	-1.0451414	-0.3175879
H	-0.0864652	-0.0314155	-0.9572621
B	-0.8911798	-0.1627980	0.0847666
H	-2.5869887	0.8553957	1.4246676
H	2.5897361	-0.8542405	-1.4230342
H	0.0872751	0.0307923	0.9574313
C	1.3606560	1.6704758	-0.1986053
C	1.5062006	2.5241179	0.9175540
C	1.7783345	2.1462977	-1.4534253
C	2.0421954	3.8050182	0.7437389
C	2.3017517	3.4263245	-1.6215213
H	2.1416714	4.4569826	1.6093023
C	2.4381828	4.2622040	-0.5130786
H	2.6053963	3.7680969	-2.6080885
H	2.8442376	5.2642836	-0.6249248
C	-1.3605457	-1.6704836	0.1984783
C	-1.5068301	-2.5235033	-0.9180817
C	-1.7783158	-2.1465871	1.4531496
C	-2.0439451	-3.8040017	-0.7448169
C	-2.3028088	-3.4262543	1.6206772
H	-2.1440686	-4.4554789	-1.6106742
C	-2.4402035	-4.2614263	0.5118328
H	-2.6066336	-3.7682687	2.6071081
H	-2.8472822	-5.2631395	0.6232519
H	-1.6963945	-1.4910274	2.3176260
H	1.6971739	1.4902103	-2.3175736
C	1.0978639	2.0640499	2.2960681
H	1.6116221	1.1352577	2.5757997
H	0.0200325	1.8682564	2.3373828
H	1.3329139	2.8225923	3.0485254
C	-1.0979467	-2.0631998	-2.2963490
H	-0.0197780	-1.8692880	-2.3376761
H	-1.3344692	-2.8207797	-3.0493181

H	-1.6101200	-1.1333162	-2.5753374
S	-3.6660626	0.5336078	-0.8523771
S	3.6659361	-0.5338487	0.8552915
C	-4.5537244	-0.7699381	0.0407332
H	-5.5302784	-0.8962712	-0.4308857
H	-3.9653300	-1.6829528	-0.0456815
H	-4.6506542	-0.4801207	1.0885762
C	-4.8192121	1.9061435	-0.5840283
H	-4.9440871	2.0634323	0.4893316
H	-4.3724125	2.7908886	-1.0385750
H	-5.7688237	1.6660324	-1.0654632
C	4.8190377	-1.9065796	0.5877043
H	4.3714904	-2.7913848	1.0414013
H	4.9449901	-2.0633766	-0.4855938
H	5.7681807	-1.6669154	1.0702927
C	4.5542175	0.7697031	-0.0371372
H	4.6517583	0.4800100	-1.0849608
H	3.9657009	1.6826766	0.0489268
H	5.5304803	0.8960914	0.4350644
C	-1.8410283	2.6084482	0.0788116
C	-1.9366716	3.4631946	1.1936614
C	-1.4676324	3.1987797	-1.1529883
C	-1.6367059	4.8237766	1.1287895
H	-2.2404905	3.0338435	2.1464991
C	-1.1696893	4.5646503	-1.2162715
C	-1.2441271	5.3799470	-0.0876518
H	-1.7045694	5.4429531	2.0206003
H	-0.8552836	4.9913930	-2.1669795
H	-0.9904576	6.4348445	-0.1583178
C	1.8416085	-2.6082306	-0.0796611
C	1.9366838	-3.4619240	-1.1953422
C	1.4682250	-3.1996348	1.1516370
C	1.6359570	-4.8223970	-1.1318051
H	2.2408046	-3.0318468	-2.1477664
C	1.1694637	-4.5653878	1.2135810
C	1.2431864	-5.3795743	0.0841208
H	1.7033487	-5.4406869	-2.0242669
H	0.8549953	-4.9929371	2.1639058
H	0.9890023	-6.4344137	0.1537358
C	-1.4024431	2.3841761	-2.4217138
H	-2.3999141	2.2502432	-2.8625960
H	-0.7738655	2.8742974	-3.1710640
H	-1.0009813	1.3850698	-2.2317572
C	1.4041984	-2.3863964	2.4212986
H	0.7740096	-2.8759251	3.1696979
H	2.4016996	-2.2554062	2.8629979
H	1.0051569	-1.3861342	2.2322781

P2a : BH/BB dimer of **P**
82
Energy = -2142.664376432

B	0.1333534	0.4118580	0.4338241
C	0.1986582	1.6702008	-0.5911596
C	1.1567255	2.6569543	-0.2651857
C	1.3063306	3.8636176	-0.9416713
C	0.4720852	4.1398838	-2.0235754
C	-0.4698678	3.1872563	-2.3927913
C	-0.6232221	1.9670117	-1.7115140
C	-1.6908469	1.0579838	-2.2622141
H	-2.6404166	1.5978432	-2.3453853
H	-1.4159575	0.7168774	-3.2683208
H	-1.8588932	0.1837174	-1.6446090
H	-1.1253464	3.3869064	-3.2390384
H	0.5554378	5.0753081	-2.5711985
H	2.0644295	4.5755639	-0.6250183
H	1.8320628	2.4740346	0.5623060
H	0.1800412	-0.7415224	-0.3076709
B	1.4395026	-0.0664206	1.5017845
C	2.6407251	-0.8185700	0.7273428
C	3.3570334	-0.1509824	-0.2851944
C	4.3826313	-0.7567963	-1.0114872
C	4.7228501	-2.0816366	-0.7393329
C	4.0273410	-2.7715169	0.2543893
C	2.9999475	-2.1646942	0.9906133
C	2.2885925	-2.9842197	2.0430130
H	2.3596721	-2.5185753	3.0323980
H	1.2213704	-3.0697518	1.8102048
H	2.7153431	-3.9908251	2.1024170
H	4.2863470	-3.8078371	0.4668313
H	5.5170145	-2.5755397	-1.2942643
H	4.9054320	-0.2027616	-1.7881364
H	3.0898675	0.8772933	-0.5196026
H	1.0228986	-0.6866985	2.4460829
S	2.3985147	1.3794877	2.4921604
C	1.1079772	2.2355684	3.4262747
H	0.5252231	2.8104203	2.7045630
H	0.4760248	1.4977464	3.9238557
H	1.5678546	2.9045374	4.1551858
C	3.1984040	0.4441173	3.8201736
H	3.9216769	-0.2128716	3.3337406
H	2.4456267	-0.1487094	4.3435150
H	3.7069650	1.1336429	4.4961267
B	-0.8789228	-1.1942286	0.2656683
C	-1.5079262	-2.0798543	-0.9177643
C	-0.9945958	-2.0260786	-2.2272422
C	-1.4402552	-2.8603151	-3.2512337

C	-2.4274815	-3.8088965	-2.9850788
C	-2.9193825	-3.9232960	-1.6845584
C	-2.4654827	-3.0921376	-0.6515877
C	-2.9782192	-3.3344144	0.7464990
H	-3.6503732	-4.1977291	0.7735842
H	-2.1442735	-3.5216948	1.4351470
H	-3.5207870	-2.4704821	1.1447941
H	-3.6638903	-4.6847220	-1.4565452
H	-2.7941912	-4.4654540	-3.7703733
H	-1.0149950	-2.7730157	-4.2485517
H	-0.2110597	-1.3053631	-2.4494220
H	-0.4359139	-1.9304915	1.1144770
B	-1.6723989	0.1887313	1.0867435
C	-2.9877103	0.9237497	0.5350952
C	-4.0007211	0.1518554	-0.0632434
C	-5.1746211	0.7095571	-0.5646106
C	-5.3668021	2.0883853	-0.4771161
C	-4.3837041	2.8772025	0.1191244
C	-3.2038420	2.3216675	0.6349115
C	-2.1961358	3.2563599	1.2626172
H	-1.9246440	2.9392499	2.2753210
H	-1.2722293	3.2942189	0.6765366
H	-2.6026170	4.2700070	1.3268409
H	-4.5319449	3.9531328	0.1906037
H	-6.2715842	2.5474115	-0.8678039
H	-5.9260209	0.0765600	-1.0303505
H	-3.8389023	-0.9154628	-0.1764915
H	-0.8468368	1.0328039	1.4728790
S	-2.3452036	-0.5798859	2.7745949
C	-1.0982094	-1.3660898	3.8272207
H	-0.3702908	-0.6348924	4.1746090
H	-0.6053787	-2.1241005	3.2196602
H	-1.6304421	-1.8225520	4.6640483
C	-2.8277971	0.8090340	3.8321608
H	-3.5393717	1.4037168	3.2578618
H	-1.9550573	1.4062059	4.1012713
H	-3.3096809	0.4057567	4.7242740

P·THF : THF adduct of **P**
54
Energy = -1303.933334727

B	-0.6986313	-0.7560359	0.5117877
C	-2.1913680	-0.3131645	0.1476053
C	-2.6161762	-0.2052667	-1.1921915
C	-3.8692159	0.2971357	-1.5466785
C	-4.7503912	0.7153098	-0.5479257
C	-4.3588692	0.6135756	0.7897032
C	-3.1028991	0.1094622	1.1484646
C	-2.7152011	0.0555925	2.6062886
H	-3.5248775	0.4263138	3.2432398
H	-2.4677276	-0.9688409	2.9083100
H	-1.8172474	0.6561694	2.7932067
H	-5.0411490	0.9397846	1.5735659
H	-5.7280011	1.1162797	-0.8036975
H	-4.1525657	0.3715049	-2.5943045
H	-1.9325461	-0.5087722	-1.9833443
H	-0.6098022	-1.3867672	1.5542140
B	0.4298459	0.5954371	0.5096512
C	1.9744718	0.3117197	0.1888031
C	2.5230346	0.3827329	-1.1076914
C	3.8466183	0.0422272	-1.3916531
C	4.6790947	-0.3970956	-0.3619590
C	4.1659282	-0.4759649	0.9350815
C	2.8415604	-0.1261582	1.2232443
C	2.3348260	-0.2428195	2.6402184
H	2.0445686	0.7391682	3.0335218
H	1.4344530	-0.8667808	2.6824353
H	3.0973277	-0.6723430	3.2981169
H	4.8084219	-0.8185515	1.7453716
H	5.7112570	-0.6738659	-0.5620390
H	4.2207768	0.1108423	-2.4108017
H	1.8857661	0.6928649	-1.9367313
H	0.2655948	1.2221956	1.5429455
S	-0.1285031	2.0213244	-0.8132287
C	-1.5504741	2.8425970	-0.0470402
H	-2.4382587	2.2486775	-0.2572622
H	-1.3783986	2.9082424	1.0294133
H	-1.6413420	3.8351011	-0.4929438
C	1.0994043	3.3235246	-0.5032146
H	2.0574431	2.9664770	-0.8801758
H	1.1617228	3.4945839	0.5738445
H	0.7949745	4.2291315	-1.0315273
C	1.0122545	-2.5576879	-0.4647059
C	-1.3408407	-3.0593565	-0.6488053
C	0.8399646	-3.9789836	-0.9625731
H	1.2810158	-2.5123452	0.5943814
H	1.6884082	-1.9361001	-1.0455476
C	-0.5511328	-4.3395310	-0.4250014
H	-1.7949359	-3.0058353	-1.6396690
H	-2.0828616	-2.8421174	0.1177079
H	0.8547432	-4.0104058	-2.0571868
H	1.6282143	-4.6340904	-0.5834897
H	-1.0077854	-5.1863526	-0.9426474
H	-0.4948055	-4.5716703	0.6431306
O	-0.3348100	-1.9553133	-0.5970948

P : adduct H(*o*-tolyl)BB(*o*-tolyl)H·SMe_2
41
Energy = -1071.319007786

B	-0.5832839	0.2239953	1.2543629
C	-1.9242910	-0.0723781	0.5161084
C	-1.9549844	-0.0924539	-0.8956331
C	-3.1122971	-0.3969053	-1.6092595
C	-4.2968632	-0.6478854	-0.9148135
C	-4.3026491	-0.6101719	0.4810782
C	-3.1370208	-0.3426649	1.2073409
C	-3.1925259	-0.3031736	2.7151099
H	-4.1332058	-0.7215810	3.0845999
H	-2.3575598	-0.8629497	3.1514409
H	-3.1134633	0.7256044	3.0884680
H	-5.2290182	-0.8034233	1.0184586
H	-5.2132507	-0.8704596	-1.4552451
H	-3.0949165	-0.4272739	-2.6954674
H	-1.0381309	0.1117990	-1.4428584
H	-0.3525961	-0.4514950	2.2291226
B	0.6255041	1.3320702	0.8346009
C	1.8707607	0.5100278	0.2197492
C	1.9725128	0.2123333	-1.1524745
C	3.0356565	-0.5171342	-1.6871233
C	4.0361361	-0.9932953	-0.8403630
C	3.9522332	-0.7268179	0.5279576
C	2.8921028	0.0110834	1.0691803
C	2.8588747	0.2745046	2.5549820
H	2.9204160	1.3500454	2.7625619
H	1.9206046	-0.0754832	2.9994328
H	3.6917731	-0.2251685	3.0592481
H	4.7286578	-1.0979662	1.1950403
H	4.8705007	-1.5666153	-1.2362113
H	3.0774092	-0.7151802	-2.7555256
H	1.2003131	0.5686439	-1.8333571
H	0.9310759	2.0696512	1.7515887
S	-0.0037334	2.6098264	-0.5321196
C	-1.3128637	3.4831458	0.3593805
H	-2.1408568	2.7790605	0.4581228
H	-0.9423371	3.7776311	1.3433430
H	-1.6228830	4.3508414	-0.2247038
C	1.3006642	3.8666385	-0.5378688
H	2.1839527	3.3900075	-0.9664844
H	1.5002163	4.1770009	0.4896432
H	0.9887798	4.7088409	-1.1577001

Q : adduct Me_2S·H(*o*-tolyl)BB(*o*-tolyl)H·SMe_2
50
Energy = -1549.459065576

B	-0.5095048	-0.7181392	0.5554464
C	-2.0434147	-0.4368276	0.1925586
C	-2.5060546	-0.3479832	-1.1360736
C	-3.8101668	0.0322313	-1.4592282
C	-4.7081169	0.3431551	-0.4378916
C	-4.2804651	0.2589431	0.8901990
C	-2.9747036	-0.1247210	1.2174098
C	-2.5536043	-0.1620800	2.6662972
H	-3.3656160	0.1713001	3.3207081
H	-2.2566098	-1.1749875	2.9634272
H	-1.6789445	0.4775765	2.8325300
H	-4.9749736	0.5040181	1.6927317
H	-5.7257348	0.6487956	-0.6679615
H	-4.1185550	0.0939398	-2.5004720
H	-1.8159931	-0.5609814	-1.9529365
H	-0.3431384	-1.3697275	1.5691396
B	0.5088427	0.7189824	0.5530304
C	2.0428701	0.4375118	0.1906318
C	2.5060294	0.3486652	-1.1378361
C	3.8101988	-0.0318742	-1.4603869
C	4.7074556	-0.3436765	-0.4387009
C	4.2795444	-0.2585465	0.8892498
C	2.9739098	0.1259936	1.2159183
C	2.5528775	0.1657707	2.6647525
H	2.2562459	1.1792674	2.9602160
H	1.6780040	-0.4732781	2.8321334
H	3.3648061	-0.1668176	3.3196791
H	4.9736950	-0.5036207	1.6920832
H	5.7247355	-0.6507082	-0.6684036
H	4.1193049	-0.0927600	-2.5014645
H	1.8164558	0.5621166	-1.9549533
H	0.3425810	1.3739340	1.5645570
S	-0.1510169	2.0231504	-0.8204870
C	-1.5841532	2.8038058	-0.0348078
H	-2.4197050	2.1084234	-0.1063736
H	-1.3420574	3.0051359	1.0109891
H	-1.8066316	3.7263019	-0.5745955
C	1.0290023	3.3916189	-0.6493159
H	1.9843313	3.0418333	-1.0415335
H	1.1271688	3.6402207	0.4097410
H	0.6705147	4.2463127	-1.2262942
S	0.1495929	-2.0260932	-0.8145817
C	1.5841500	-2.8032950	-0.0281922
H	2.4188837	-2.1072982	-0.1026709
H	1.3431879	-3.0017052	1.0184139
H	1.8069728	-3.7271378	-0.5655377
C	-1.0286844	-3.3954533	-0.6380023
H	-0.6701188	-4.2513947	-1.2130870

H -1.9848779 -3.0477421 -1.0298385
H -1.1252322 -3.6412127 0.4218679

Ra : cyclic triborane(7) B_3H_5(*o*-tolyl)$_2$
36
Energy = -619.8830052202
B 0.7241696 -0.9361458 -0.2763586
H 1.8923372 -0.9007434 -0.4825971
B -0.3257084 -1.3221377 -1.5704497
B -0.3114451 0.4025861 -0.7484417
H 0.2477929 -1.4933458 -2.6067534
H -1.4844691 0.5569516 -0.6233160
C 0.1492389 -0.4042667 1.1086932
C 0.9628694 0.3559503 2.0056851
C -1.1294135 -0.8306486 1.5524858
C 0.4759584 0.6279109 3.2864559
C -1.5906079 -0.5557703 2.8318407
H -1.7534390 -1.3930163 0.8635942
C -0.7804296 0.1801982 3.7004836
H 1.0932585 1.1999172 3.9744328
H -2.5697861 -0.9003570 3.1497414
H -1.1277584 0.4103959 4.7039847
C 2.3243969 0.8536129 1.6125289
H 2.2657285 1.4956376 0.7264278
H 2.7781056 1.4184791 2.4306819
H 2.9841109 0.0187055 1.3519339
H 0.2437145 -2.1016298 -0.6449632
H -1.4764149 -1.6353481 -1.5054338
C 0.5576490 1.6560403 -1.1001777
C 1.6359615 1.5449291 -2.0001231
C 0.2437997 2.9414568 -0.5832132
C 2.3765630 2.6550615 -2.4027109
H 1.8859904 0.5641948 -2.3961389
C 1.0041785 4.0455883 -0.9801461
C 2.0617323 3.9111660 -1.8832217
H 3.1960986 2.5410222 -3.1070902
H 0.7658197 5.0269575 -0.5755225
H 2.6367603 4.7855206 -2.1768532
C -0.8743416 3.1202272 0.4119563
H -0.9984690 4.1738721 0.6771382
H -1.8222517 2.7443396 0.0096369
H -0.6778638 2.5520297 1.3286415

R : SMe_2 adduct of **Ra**
45
Energy = -1098.018805596
B 0.4611748 -0.9432088 -0.1917804
H 1.5090692 -1.3437869 -0.6266378
B -0.9538246 -1.1966104 -1.2321664
B -0.1271920 0.4142555 -1.3240098
H -0.8298281 -1.8811667 -2.2057837
H -1.0424917 0.2093850 -2.1161899
S 1.4213362 0.6309995 -2.4997863
C 1.4424083 -0.8244794 -3.5735960
C 0.8592541 1.9022038 -3.6637737
H 1.6450819 -1.6831218 -2.9330025
H 0.4759425 -0.9326405 -4.0667549
H 2.2464316 -0.6919760 -4.2987491
H -0.0616796 1.5718708 -4.1474603
H 0.6786667 2.7999060 -3.0704301
H 1.6492921 2.0825914 -4.3939611
C 0.4270142 -0.4796170 1.3316810
C 1.6125308 -0.2928739 2.0801021
C -0.7949443 -0.1946137 1.9648675
C 1.5344723 0.1643920 3.4032950
C -0.8680349 0.2578400 3.2801801
H -1.7163838 -0.3136897 1.3982818
C 0.3088586 0.4406934 4.0077671
H 2.4551094 0.3119419 3.9654736
H -1.8327289 0.4778748 3.7308760
H 0.2743943 0.7982009 5.0339784
C 2.9776723 -0.5508227 1.4818502
H 3.1484262 0.0645934 0.5906652
H 3.7646297 -0.3241913 2.2078737
H 3.0892521 -1.5944844 1.1671405
H -0.3037462 -1.9676760 -0.2927997
H -2.0565602 -1.0934766 -0.7782060
C -0.3315604 1.7940687 -0.5509978
C 0.7651323 2.3894031 0.0976792
C -1.5782068 2.4581856 -0.4791592
C 0.6584535 3.5848562 0.8036530
H 1.7302654 1.8889219 0.0650642
C -1.6802814 3.6575616 0.2396663
C -0.5786420 4.2238450 0.8795342
H 1.5285603 4.0052099 1.3010885
H -2.6462280 4.1564136 0.2934233
H -0.6872387 5.1542048 1.4312309
C -2.8189007 1.9023085 -1.1368650
H -3.6495533 2.6090500 -1.0515574
H -2.6521754 1.6950792 -2.2004088
H -3.1260177 0.9567112 -0.6757698

SMe_2 : dimethyl sulfide
9
Energy = -478.1072166427
S 0.0005290 0.1063091 -0.4769751

C 1.3861905 -0.8250838 0.2454461
H 2.3054567 -0.3626518 -0.1212441
H 1.3602221 -0.7652012 1.3368823
H 1.3533959 -1.8702203 -0.0739525
C -1.3947271 -0.8113889 0.2444955
H -1.3658909 -0.7562304 1.3361033
H -2.3091280 -0.3365721 -0.1185224
H -1.3750392 -1.8555526 -0.0791581

S : symmetric isomer of adduct **R**
45
Energy = -1098.020844864
B 0.5503590 0.3386366 0.0323319
H 1.5982685 -0.2097125 0.2671856
B -0.6305163 -0.4042305 -1.0420893
B 0.6918463 0.6205486 -1.7955966
H -0.4155483 -1.4966577 -1.4921731
H 1.1068900 1.7418418 -1.7101827
H -0.1227070 0.5704107 -2.6856540
S 2.1208433 -0.5278868 -2.4657274
C 2.1965380 -0.0794094 -4.2202834
C 3.6545987 0.2363485 -1.8824966
H 1.2859508 -0.4629991 -4.6823833
H 2.2458646 1.0067145 -4.3165113
H 3.0726084 -0.5560931 -4.6626962
H 3.6581621 1.2951146 -2.1452293
H 3.6800514 0.1070814 -0.8005828
H 4.4917426 -0.2892546 -2.3438602
C 0.1649899 1.5738941 0.9613417
C 1.1011899 2.5851441 1.2923464
C -1.1159977 1.6682748 1.5353468
C 0.7245826 3.6385252 2.1362197
C -1.4847394 2.7167262 2.3784884
H -1.8510255 0.9008414 1.3093592
C -0.5595191 3.7163670 2.6769140
H 1.4563267 4.4079658 2.3765502
H -2.4871395 2.7513465 2.7981781
H -0.8283356 4.5432380 3.3296088
C -2.1289337 0.1275200 -1.0994968
C -3.1830485 -0.6737500 -0.5978619
C -2.4556024 1.3913134 -1.6158046
C -4.4992016 -0.1964989 -0.6340261
C -3.7682925 1.8626975 -1.6497474
H -1.6578509 2.0288477 -1.9894363
C -4.7987616 1.0628703 -1.1558295
H -5.3001147 -0.8225991 -0.2448179
H -3.9844192 2.8480181 -2.0551170
H -5.8270905 1.4151070 -1.1729003
C 2.5127062 2.5501569 0.7594258
H 2.5281800 2.6483877 -0.3319650
H 3.1115570 3.3617379 1.1833021
H 3.0007963 1.5984223 0.9997379
C -2.8984697 -2.0279271 0.0074842
H -3.8249233 -2.5302122 0.3008811
H -2.3583721 -2.6730679 -0.6952972
H -2.2659914 -1.9377857 0.9007457
H -0.3060335 -0.6204830 0.2512347

THF : tetrahydrofuran
13
Energy = -232.5930405661
C 0.0452644 1.1709607 -0.1594499
O -0.7806749 -0.0244195 -0.1294478
C 0.0591201 -1.1808250 0.1207265
C 1.4907325 -0.7219016 -0.1468747
C 1.4351077 0.7425209 0.3145056
H 0.0712945 1.5500704 -1.1900411
H -0.4191871 1.9251678 0.4841122
H -0.0682549 -1.4970126 1.1660515
H -0.2756271 -1.9880190 -0.5379167
H 2.2296314 -1.3175788 0.3960616
H 1.7155364 -0.7769400 -1.2179052
H 1.5013235 0.7977742 1.4067596
H 2.2287464 1.3612983 -0.1128381

TS1Ha : transition structure, HH/BB of H_2 to **1**
60
Energy = -1135.448737660
B -0.7978073 -0.7534089 0.6492742
C -0.4561573 -2.3001418 0.7827288
C -1.8455314 -0.1799929 -0.4298680
B 0.3390279 0.5064707 0.1738651
C -0.8540527 -3.1951130 -0.2235900
C 0.2499252 -2.8296046 1.8919343
C -3.0900212 0.4209609 -0.0957171
C -1.5273614 -0.3201924 -1.7958380
C 1.4740975 0.0996741 -0.8272389
C 0.2217987 1.9890857 0.6965285
C -0.5558033 -4.5574699 -0.1661146
H -1.4104668 -2.8155194 -1.0767011
C 0.5412440 -4.1971864 1.9460156
C -3.9415809 0.8460750 -1.1200764
C -2.3859200 0.1111708 -2.8072956
H -0.5817078 -0.7789500 -2.0685699
C 2.1955787 -1.0835222 -0.5730381
C 1.8812345 0.9067705 -1.9242216

C	1.2775508	2.5259294	1.4797726
C	-0.8842341	2.8188193	0.4330263
C	0.1492899	-5.0643217	0.9246711
H	-0.8749507	-5.2178791	-0.9686193
H	1.0887724	-4.5860599	2.8024947
C	-3.5984913	0.7046067	-2.4669976
H	-4.8958623	1.2964308	-0.8563173
H	-2.1044605	-0.0139254	-3.8490953
C	3.3037963	-1.4545995	-1.3322332
H	1.8821399	-1.7213353	0.2464965
C	2.9724775	0.5047150	-2.7008605
C	1.1768550	3.8295355	1.9784711
C	-0.9644242	4.1224793	0.9206477
H	-1.6864755	2.4438061	-0.1937430
H	0.3910016	-6.1223206	0.9840777
H	-4.2790978	1.0522397	-3.2394060
H	3.8483156	-2.3650872	-1.0967089
C	3.6916313	-0.6561603	-2.4069836
H	3.2675706	1.1161680	-3.5511367
C	0.0687816	4.6323430	1.7054768
H	1.9882598	4.2212018	2.5889711
H	-1.8309700	4.7355221	0.6861021
H	4.5426709	-0.9366241	-3.0223237
H	0.0175071	5.6441768	2.0987047
H	-1.4131021	-0.4845304	1.7954420
H	-0.6149738	-0.0213291	1.6857930
C	2.5211886	1.7207362	1.7791074
H	2.2781674	0.7136937	2.1372238
H	3.1331347	1.5926001	0.8789922
H	3.1301859	2.2146175	2.5417623
C	1.1519788	2.1746533	-2.2970346
H	1.3409061	2.9686824	-1.5663401
H	1.4774375	2.5281494	-3.2799356
H	0.0686903	2.0182219	-2.3237524
C	-3.5274226	0.6018352	1.3361298
H	-2.8925479	1.3242926	1.8632086
H	-4.5577007	0.9647083	1.3828992
H	-3.4757104	-0.3455286	1.8892428
C	0.7052301	-1.9311044	3.0139533
H	1.3184275	-1.1040599	2.6320014
H	-0.1446947	-1.4808547	3.5438652
H	1.3008832	-2.4818626	3.7466464

TS1Hb : TS, BH/BC of two **9m** HB(o-tolyl)$_2$
60
Energy = -1135.485972790

B	-1.2495046	-0.8898827	1.0312767
C	-0.8811654	-2.4086049	0.9242150
C	-1.7916097	-0.0572079	-0.1858541
B	0.7993816	0.6091539	0.2400645
C	-1.4542173	-3.2079306	-0.0863801
C	-0.0398156	-3.0516797	1.8737481
C	-2.6999726	1.0235716	-0.0054815
C	-1.3963408	-0.3663340	-1.5075610
C	1.6872451	-0.0550808	-0.8486323
C	0.4950189	2.0948866	0.6125013
C	-1.2038218	-4.5753718	-0.1869455
H	-2.1287696	-2.7446493	-0.8011406
C	0.2233826	-4.4201673	1.7519206
C	-3.2132681	1.6844526	-1.1252202
C	-1.8802794	0.3291485	-2.6143683
H	-0.6783778	-1.1657237	-1.6603723
C	2.0879928	-1.3777756	-0.5458457
C	2.2126876	0.5349800	-2.0330870
C	0.8540558	2.5530415	1.9071790
C	-0.2017556	2.9790164	-0.2286498
C	-0.3480779	-5.1825557	0.7316400
H	-1.6694308	-5.1609597	-0.9752633
H	0.8811909	-4.8986966	2.4745619
C	-2.8112895	1.3487163	-2.4199089
H	-3.9173126	2.5007280	-0.9801775
H	-1.5414152	0.0740091	-3.6145426
C	2.9722796	-2.0983354	-1.3423213
H	1.6783276	-1.8474028	0.3420940
C	3.1032753	-0.1980577	-2.8239444
C	0.5351560	3.8596160	2.2922704
C	-0.5455524	4.2667728	0.1758028
H	-0.5220272	2.6355992	-1.2057049
H	-0.1329574	-6.2458491	0.6633247
H	-3.2114544	1.8957908	-3.2695953
H	3.2530438	-3.1127405	-1.0731583
C	3.4868693	-1.4990617	-2.4909266
H	3.5006290	0.2594981	-3.7273014
C	-0.1651628	4.7150332	1.4404637
H	0.8297684	4.2055608	3.2810046
H	-1.1068678	4.9148383	-0.4920876
H	4.1794519	-2.0390646	-3.1313718
H	-0.4154737	5.7214102	1.7661042
H	-1.3757172	-0.4277000	2.1280394
H	0.5022638	-0.1770172	1.1294314
C	1.5813347	1.6499033	2.8754386
H	0.9590675	0.7865692	3.1403159
H	2.5095885	1.2562928	2.4450443
H	1.8333402	2.1852762	3.7952841
C	1.8227092	1.9136525	-2.4959336
H	2.0360350	2.6718157	-1.7356163

H 2.3573990 2.1753647 -3.4133187
H 0.7489487 1.9610075 -2.7045159
C -3.1226887 1.4878042 1.3663523
H -2.2546100 1.6002245 2.0216193
H -3.6365528 2.4507828 1.2994297
H -3.8012357 0.7704196 1.8435042
C 0.6098433 -2.2806455 2.9950188
H 1.3842684 -1.6026096 2.6132039
H -0.1195312 -1.6558648 3.5209818
H 1.0807776 -2.9584221 3.7125320

TS1Hc : TS, BH/BC addition of **7m** and **2**
60
Energy = -1135.471234287
B -0.7526842 -1.0598984 1.4417370
C -0.3864299 -2.5193378 1.0004388
C -1.4377352 0.2409665 -0.3573731
B -0.0145609 0.8754633 -0.0619154
C -1.1212003 -3.2554373 0.0559709
C 0.6836703 -3.1767822 1.6632325
C -2.6780091 0.6556653 0.2295605
C -1.5085354 -0.5358313 -1.5481270
C 1.2311439 0.3395939 -0.8915499
C 0.1159478 2.2979579 0.6073113
C -0.7869258 -4.5643953 -0.2866131
H -1.9641948 -2.7818917 -0.4370415
C 1.0038453 -4.4960975 1.3218827
C -3.8791720 0.3369366 -0.4162102
C -2.7059401 -0.8308307 -2.1790937
H -0.5754163 -0.8808367 -1.9829518
C 2.0305289 -0.7427973 -0.4943474
C 1.5502201 0.9764417 -2.1188615
C 1.1303995 2.6749997 1.5220344
C -0.7619272 3.3010218 0.1492188
C 0.2901386 -5.1876518 0.3438186
H -1.3655131 -5.0958320 -1.0375571
H 1.8272167 -4.9874935 1.8361252
C -3.9011220 -0.3790978 -1.6098611
H -4.8169178 0.6570695 0.0319866
H -2.7172533 -1.4066419 -3.0997428
C 3.1146586 -1.1875776 -1.2504582
H 1.7938814 -1.2582631 0.4270172
C 2.6357747 0.5188537 -2.8754473
C 1.2136477 4.0075663 1.9458734
C -0.6541400 4.6283936 0.5553793
H -1.5421995 3.0310805 -0.5587170
H 0.5652219 -6.2073182 0.0868533
H -4.8511940 -0.5968172 -2.0904475
H 3.7060095 -2.0312351 -0.9039013
C 3.4239982 -0.5508453 -2.4504474
H 2.8605065 1.0117249 -3.8194681
C 0.3393913 4.9836981 1.4678228
H 1.9878451 4.2840240 2.6588376
H -1.3413211 5.3760169 0.1681638
H 4.2623478 -0.8858810 -3.0557063
H 0.4360074 6.0125666 1.8046546
H -1.7782445 -0.8481211 2.0101436
H 0.1477852 -0.2874915 1.6907704
C 2.1009204 1.6697421 2.0897160
H 1.5964174 0.9823769 2.7799921
H 2.5454344 1.0580004 1.2987603
H 2.9022276 2.1710386 2.6401308
C 0.7288842 2.1298274 -2.6464590
H 0.8995773 3.0404601 -2.0614776
H 0.9826970 2.3398495 -3.6896001
H -0.3451852 1.9145562 -2.5937557
C -2.7810258 1.3954338 1.5403713
H -1.8086466 1.5968087 1.9850634
H -3.3048473 2.3478157 1.4004715
H -3.3709459 0.7984558 2.2457482
C 1.4613559 -2.4954485 2.7678305
H 2.0140810 -1.6214252 2.4009908
H 0.7975206 -2.1373066 3.5627680
H 2.1823099 -3.1860190 3.2143454

TS2Ha : TS, BH/BC addition of HBcat and **2**
57
Energy = -1245.547994016
B -0.9059507 0.0526772 0.1315923
C 0.1546440 -0.0364289 -1.1325759
C -1.7244140 -1.2415146 0.6281080
C 0.4628664 -1.2386145 -1.8431539
C 0.5800823 1.1874707 -1.7096192
C -1.3704075 -1.9044962 1.8167118
C -2.8696597 -1.7111655 -0.0584345
C 1.1237128 -1.1460541 -3.0744865
C 1.2496849 1.2578617 -2.9201829
H 0.3796973 2.1032999 -1.1626334
C -2.0900398 -2.9909867 2.3141287
H -0.4960739 -1.5754618 2.3742521
C -3.5862940 -2.8037378 0.4434948
C 1.5146765 0.0766186 -3.6161072
H 1.3479513 -2.0637385 -3.6126215
H 1.5593816 2.2186040 -3.3203365
C -3.2085348 -3.4485478 1.6212576
H -1.7747083 -3.4743129 3.2352342

H	-4.4577376	-3.1544975	-0.1059642
H	2.0336927	0.1046590	-4.5703061
H	-3.7817604	-4.2949689	1.9901805
B	0.9814205	0.0588052	0.7642683
H	-0.1137997	0.1147486	1.3366182
O	1.7467880	-1.1108901	0.9519360
O	1.7786834	1.2183513	0.8800637
C	3.0565728	-0.6631000	0.9220655
C	3.0755273	0.7342686	0.8786252
C	4.2588277	1.4508853	0.8494468
C	5.4451763	0.6999632	0.8635581
C	5.4256202	-0.6983885	0.9075734
C	4.2181956	-1.4142040	0.9392979
H	4.2670738	2.5351533	0.8140367
H	6.3982004	1.2194585	0.8366887
H	6.3634876	-1.2453701	0.9143317
H	4.1944264	-2.4982743	0.9719576
C	-1.6745427	1.4511090	0.3940305
C	-1.8103806	1.8186994	1.7513453
C	-2.3059494	2.2812948	-0.5627190
C	-2.5069089	2.9464058	2.1693886
H	-1.3556257	1.1866433	2.5131787
C	-3.0158473	3.4158632	-0.1295298
C	-3.1199419	3.7589783	1.2133574
H	-2.5749006	3.1861509	3.2272363
H	-3.5012625	4.0388429	-0.8787503
H	-3.6752428	4.6444182	1.5113858
C	-2.2861155	2.0465898	-2.0593041
H	-1.6594689	2.7963383	-2.5574974
H	-1.9056727	1.0655566	-2.3333286
H	-3.2979321	2.1501119	-2.4671803
C	-3.3340293	-1.0656034	-1.3383113
H	-3.6884850	-0.0457177	-1.1542622
H	-2.5197480	-0.9980235	-2.0691626
H	-4.1512532	-1.6348951	-1.7899436
C	0.1362479	-2.6175407	-1.3346475
H	0.4655681	-2.7480778	-0.3014011
H	-0.9418938	-2.8058078	-1.3480414
H	0.6246005	-3.3684664	-1.9618326

TS2Hb : TS, BH/BC addition of **9m** and **4**
57
Energy = -1245.546513858

B	-0.6575926	0.0319368	0.4463541
C	-0.0757716	0.2623799	-1.4865759
C	-0.8866946	-1.4710567	0.9129267
C	-0.3643072	-0.8365545	-2.3535431
C	-0.4081368	1.5752669	-1.9295534
C	0.1203727	-1.9939200	1.7536801
C	-2.0281995	-2.2836453	0.6945565
C	-1.0287341	-0.5790940	-3.5602081
C	-0.9995537	1.8089497	-3.1560304
H	-0.1729475	2.4102885	-1.2777281
C	0.0269284	-3.2453932	2.3578224
H	1.0085972	-1.3969883	1.9391024
C	-2.1082315	-3.5468132	1.2932594
C	-1.3326917	0.7173449	-3.9668359
H	-1.2764929	-1.4155765	-4.2084864
H	-1.2251170	2.8217363	-3.4746208
C	-1.0968830	-4.0347216	2.1200671
H	0.8271289	-3.6012551	3.0012741
H	-2.9877453	-4.1587120	1.1040977
H	-1.8260681	0.8789929	-4.9211949
H	-1.1882172	-5.0190417	2.5717852
B	1.1573653	0.2610776	-0.4510077
H	0.4736933	0.3243416	0.8961042
O	2.0291057	-0.8693078	-0.3485040
O	1.9322604	1.4712943	-0.3181028
C	3.2405164	-0.3510782	0.0563282
C	3.1802197	1.0476104	0.0754042
C	4.2751263	1.8181192	0.4299981
C	5.4510533	1.1295012	0.7751254
C	5.5094349	-0.2670703	0.7570277
C	4.3932664	-1.0397203	0.3919339
H	4.2243017	2.9022170	0.4415234
H	6.3302602	1.6979055	1.0639507
H	6.4328070	-0.7683222	1.0316916
H	4.4298791	-2.1242348	0.3737979
C	-1.6019078	1.2370930	0.8964020
C	-1.1777953	1.8056442	2.1204818
C	-2.7997188	1.7353489	0.3340464
C	-1.8953689	2.7950133	2.7849900
H	-0.2498842	1.4513803	2.5653972
C	-3.5152554	2.7364273	1.0123758
C	-3.0840298	3.2649641	2.2246476
H	-1.5267596	3.1989949	3.7242080
H	-4.4348720	3.1092186	0.5645966
H	-3.6628443	4.0397498	2.7206856
C	-3.3686480	1.2938213	-0.9951513
H	-3.4017192	2.1408504	-1.6888768
H	-2.7847167	0.5037513	-1.4618035
H	-4.3980024	0.9366783	-0.8743136
C	-3.1665295	-1.8418654	-0.1820943
H	-3.6633669	-0.9633980	0.2404499
H	-2.8155615	-1.5675269	-1.1824726
H	-3.9086266	-2.6383448	-0.2865973

C 0.0831459 -2.2421901 -2.0736287
H -0.0060528 -2.5048409 -1.0187144
H -0.4857010 -2.9518161 -2.6803216
H 1.1445078 -2.3401120 -2.3326652

TS7 : TS, BH/BH dimerization of **7m**
34
Energy = -594.3792496521
B -0.9230577 -0.3759449 0.9982259
B 0.9019805 0.5402027 -0.9048757
C -2.3439972 -0.0445577 0.5117241
H -0.5232763 -1.4924577 1.1384640
H -0.2117013 0.5349109 1.3410454
H 0.0901630 -0.3110615 -1.1743116
C 2.3023746 0.1000778 -0.4554433
H 0.5931078 1.6819297 -1.0689673
C -2.7557700 1.3080053 0.5235854
C -3.2722174 -1.0268027 0.0611675
C 3.3487427 1.0129821 -0.1342566
C 2.5711981 -1.2854991 -0.3612464
C -4.0301722 1.6976587 0.1228797
H -2.0454712 2.0599393 0.8596534
C -4.5471893 -0.6236071 -0.3442850
C -2.9066928 -2.4902259 -0.0022466
C 4.5932420 0.5112171 0.2551920
C 3.1425937 2.5074630 -0.1938853
C 3.8148460 -1.7734163 0.0268534
H 1.7716930 -1.9824910 -0.6011721
C -4.9279731 0.7211797 -0.3132700
H -4.3226891 2.7436015 0.1445287
H -5.2570854 -1.3709550 -0.6915839
H -2.6212002 -2.8721298 0.9836218
H -3.7494573 -3.0815007 -0.3710785
H -2.0528175 -2.6553947 -0.6684634
C 4.8290962 -0.8644306 0.3355743
H 5.3939678 1.2046427 0.5020489
H 4.0640587 3.0330052 0.0716449
H 2.8377724 2.8280080 -1.1954716
H 2.3543537 2.8249019 0.4974113
H 3.9963508 -2.8425034 0.0902712
H -5.9276468 1.0037042 -0.6333428
H 5.8082692 -1.2239404 0.6413762

TS9 : TS, BH/BH dimerization of **9m**
60
Energy = -1135.480867505
B -0.9977401 -0.6825297 -0.5525646
B 0.6560641 1.0432799 0.4043575
H -0.1311010 0.4346170 1.1040770
H -0.5840811 0.3264789 -1.0970062
C -0.0388904 -1.9092395 -0.5743186
C -2.5190072 -0.7903583 -0.2382209
C -0.0165267 -2.9553728 0.3812897
C 0.8630639 -1.9783574 -1.6586893
C -3.3658650 0.2727688 0.1770703
C -3.1188826 -2.0424868 -0.5056077
C 0.3368839 2.5396858 0.0639757
C 2.0553178 0.4236477 0.1335104
C -0.8889083 -2.9499766 1.6169858
C 0.8828480 -4.0154359 0.2110017
C 1.7287180 -3.0511280 -1.8353231
H 0.8845795 -1.1553677 -2.3689954
C -2.8191997 1.6403628 0.4820483
C -4.7375010 0.0403176 0.3277503
C -4.4886881 -2.2545594 -0.3795884
H -2.4852532 -2.8606299 -0.8380599
C 0.3055795 3.4645366 1.1347992
C 0.0989031 3.0183537 -1.2350602
C 2.6410352 -0.5791897 0.9499723
C 2.7556470 0.8358656 -1.0195343
H -1.2495743 -1.9473122 1.8573821
H -0.3301405 -3.3359759 2.4759847
H -1.7698139 -3.5895398 1.4845934
H 0.9209930 -4.8018302 0.9624046
C 1.7401098 -4.0756337 -0.8866747
H 2.4122463 -3.0739358 -2.6791969
H -2.1231503 1.6102401 1.3278425
H -3.6253020 2.3381038 0.7254895
H -2.2537186 2.0403269 -0.3649180
H -5.3762256 0.8556848 0.6604792
C -5.3024937 -1.2053935 0.0496672
H -4.9166317 -3.2268795 -0.6079526
C 0.5656245 2.9911985 2.5459925
C 0.0373940 4.8128716 0.8791029
C -0.1752362 4.3647290 -1.4807319
H 0.1031723 2.3201811 -2.0693017
C 2.0260881 -0.9961379 2.2608724
C 3.8509765 -1.1599879 0.5571581
C 3.9493454 0.2379949 -1.4105840
H 2.3324149 1.6263823 -1.6340486
H 2.4307471 -4.9088558 -0.9884697
H -6.3732433 -1.3524835 0.1649399
H -0.1713431 2.2361968 2.8502135
H 0.5195174 3.8189924 3.2593230
H 1.5547978 2.5234723 2.6318153
H 0.0147574 5.5147027 1.7106914

C	-0.2054554	5.2677896	-0.4183648
H	-0.3670306	4.7036531	-2.4955007
H	0.9371023	-0.9295057	2.2341415
H	2.3708828	-0.3389031	3.0702253
H	2.3118221	-2.0197544	2.5181019
H	4.2898894	-1.9385511	1.1771922
C	4.4948008	-0.7734510	-0.6176153
H	4.4511769	0.5550614	-2.3208220
H	-0.4178573	6.3188176	-0.5958150
H	5.4246563	-1.2556126	-0.9085778

TSAb : TS, BH/BB addition of HBcat and **1**

72

Energy = -1541.544071130

B	1.0592914	0.5162371	0.5269386
C	2.4754711	0.8239688	-0.1689279
C	1.1135836	0.5094588	2.1322337
B	0.0409376	-0.7087284	-0.3496695
H	0.5709740	1.9564980	0.2656275
C	3.6112517	0.3583147	0.5240305
C	2.6943212	1.4686419	-1.4151294
C	0.9616860	1.6993564	2.8700026
C	1.3907102	-0.6709610	2.8619050
C	0.2381323	-0.9435728	-1.8950030
C	-0.9738399	-1.6152857	0.4190860
B	-0.6254055	1.6913992	0.1503865
C	4.8997177	0.4452327	0.0027085
H	3.4764284	-0.0979979	1.5001291
C	3.9969022	1.5657543	-1.9225361
C	1.5822136	2.1187408	-2.1957430
C	1.0263148	1.7352361	4.2602803
H	0.7805399	2.6321205	2.3404717
C	1.4609468	-0.6279494	4.2616636
C	1.6483360	-2.0012104	2.1911650
C	1.4199898	-1.3572553	-2.5496583
C	-0.8997762	-0.6983483	-2.6969322
C	-1.3107381	-2.9503684	0.0279332
C	-1.6124134	-1.1048725	1.5736580
O	-1.5001490	1.9513078	1.2083399
O	-1.2945106	1.7156992	-1.0741886
C	5.0957312	1.0453722	-1.2405193
H	5.7424177	0.0516511	0.5652815
H	4.1461180	2.0606916	-2.8799954
H	1.9563054	2.4933152	-3.1526625
H	1.1654819	2.9744102	-1.6467269
H	0.7600935	1.4281274	-2.3936756
C	1.2700847	0.5567383	4.9671513
H	0.8931234	2.6767476	4.7870809
H	1.6766960	-1.5472719	4.8036083
H	1.7678911	-1.8950444	1.1096362
H	0.8212559	-2.6997281	2.3594667
H	2.5629144	-2.4609092	2.5830555
C	2.6275291	-1.8612220	-1.7973322
C	1.4536544	-1.4096609	-3.9499647
C	-0.8548261	-0.7462413	-4.0860754
H	-1.8410558	-0.4588026	-2.2114945
C	-2.2703912	-3.6556353	0.7606437
C	-0.6370542	-3.6884548	-1.1061516
C	-2.5866887	-1.8075555	2.2741981
H	-1.3237925	-0.1283931	1.9369122
C	-2.7555204	2.0234040	0.6289964
C	-2.6306739	1.8855498	-0.7537552
H	6.0916678	1.1224106	-1.6689856
H	1.3239995	0.5630775	6.0527535
H	3.5095332	-1.2383555	-1.9796496
H	2.8681340	-2.8830517	-2.1164223
H	2.4538797	-1.8794854	-0.7205768
H	2.3716906	-1.7294681	-4.4399687
C	0.3417324	-1.0853572	-4.7221767
H	-1.7473880	-0.5309325	-4.6682170
C	-2.9227707	-3.0943237	1.8600935
H	-2.5092848	-4.6749597	0.4648876
H	-0.9082929	-4.7478186	-1.0692806
H	0.4524118	-3.6102445	-1.0468524
H	-0.9305228	-3.2909179	-2.0817814
H	-3.0630571	-1.3631739	3.1436327
C	-3.9824273	2.1970240	1.2453841
C	-3.7230346	1.9132519	-1.6036548
H	0.3980099	-1.1265988	-5.8068921
H	-3.6715903	-3.6705136	2.3976097
C	-5.0979083	2.2277891	0.3972478
H	-4.0735817	2.3004076	2.3210463
C	-4.9717611	2.0900127	-0.9920081
H	-3.6169870	1.8059986	-2.6775501
H	-6.0847406	2.3598284	0.8297180
H	-5.8625579	2.1186485	-1.6116295

TSAc : TS, BH/BC addition of HBcat and **1**

72

Energy = -1541.549024340

B	0.1710915	0.0881070	0.3574012
C	1.3859049	0.6460285	-1.1138585
C	0.7339166	0.3924373	1.8206558
B	-0.5595125	-1.4450340	0.0945035
H	-0.7157966	0.9314968	0.1332916
C	2.6886728	0.7172361	-0.5484964

C	1.2418285	0.0791153	-2.4215631
C	0.1828059	1.4687467	2.5432281
C	1.6112605	-0.4693527	2.5213643
C	-0.6078658	-2.3544752	-1.1816531
C	-1.5247395	-1.8475527	1.2755354
B	0.3540580	1.8147091	-0.7040645
C	3.8032532	0.2418319	-1.2170934
H	2.7924771	1.1646008	0.4348533
C	2.3852014	-0.3814771	-3.0799983
C	-0.0661466	0.0458829	-3.1608691
C	0.4835490	1.7088471	3.8814433
H	-0.5166169	2.1325580	2.0434852
C	1.9262986	-0.2162375	3.8618557
C	2.1939621	-1.6820568	1.8554010
C	0.4625142	-2.9819071	-1.8612692
C	-1.9026531	-2.4814363	-1.7330513
C	-1.6528251	-3.1694517	1.7826738
C	-2.3384917	-0.8472450	1.8464791
O	-0.6215790	2.3252532	-1.6222877
O	0.8339384	2.8863154	0.1068144
C	3.6466311	-0.3084209	-2.4947995
H	4.7857583	0.3009790	-0.7594024
H	2.2782436	-0.8117217	-4.0723483
H	-0.1087268	-0.8349271	-3.8067147
H	-0.1536173	0.9413201	-3.7883997
H	-0.9229120	0.0347483	-2.4900690
C	1.3743719	0.8660439	4.5458430
H	0.0247948	2.5457995	4.4017615
H	2.6032612	-0.8925423	4.3801681
H	1.4049906	-2.2570664	1.3543959
H	2.6865146	-2.3393621	2.5776715
H	2.9232200	-1.4114145	1.0829786
C	1.8458160	-3.0165401	-1.2709653
C	0.2174898	-3.6541762	-3.0645318
C	-2.1306987	-3.1258772	-2.9464603
H	-2.7441645	-2.0395837	-1.2033682
C	-2.5633600	-3.4235839	2.8146960
C	-0.8089657	-4.3156780	1.2746158
C	-3.2707186	-1.1192311	2.8447163
H	-2.2459394	0.1724132	1.4827438
C	-0.8279633	3.6329574	-1.2445757
C	0.0431671	3.9695437	-0.2042196
H	4.5122165	-0.6828513	-3.0341824
H	1.6266646	1.0394205	5.5889225
H	2.5914935	-3.2783390	-2.0264513
H	1.9054455	-3.7624370	-0.4678120
H	2.1132573	-2.0507578	-0.8381424
H	1.0487975	-4.1366080	-3.5747682
C	-1.0598345	-3.7141578	-3.6213120
H	-3.1362533	-3.1784429	-3.3555239
C	-3.3775305	-2.4182489	3.3390139
H	-2.6408445	-4.4345540	3.2097896
H	-1.0626262	-5.2400200	1.8015648
H	0.2598833	-4.1268542	1.4269766
H	-0.9562833	-4.4737371	0.2023851
H	-3.8936951	-0.3233883	3.2442089
C	-1.7274141	4.5454622	-1.7694041
C	0.0542165	5.2313662	0.3645789
H	-1.2191190	-4.2281238	-4.5655599
H	-4.0847970	-2.6502795	4.1311556
C	-1.7259537	5.8303384	-1.2003643
H	-2.4001273	4.2791851	-2.5782268
C	-0.8564464	6.1654147	-0.1575128
H	0.7325622	5.4828735	1.1732301
H	-2.4178171	6.5764064	-1.5798218
H	-0.8837868	7.1673126	0.2604804

TSAdh : TS, BB/BB dimerization of **A**
90
Energy = -1727.461434137

B	0.6272428	-0.1817899	-1.2182323
B	1.6153134	0.2476866	0.0915329
B	-1.4732718	0.3138640	-0.0777001
H	-0.5204227	0.3792282	-1.1874698
B	-0.4918997	-0.2153870	1.2024554
H	0.6548302	0.3277402	1.1838786
C	2.8083119	-0.5696697	0.7581007
C	2.7181147	-1.1716453	2.0231870
C	4.0206047	-0.7356974	0.0388711
H	1.8031223	-1.0649944	2.5967422
C	3.7643498	-1.9120978	2.5760217
C	5.0632117	-1.4830504	0.5992624
H	3.6495382	-2.3604632	3.5601442
C	4.9484577	-2.0724577	1.8593791
H	5.9841241	-1.6039163	0.0310018
H	5.7737611	-2.6482522	2.2710478
C	-2.6512243	-0.5355332	-0.7455952
C	-2.5596125	-1.1319333	-2.0126975
C	-3.8477979	-0.7485876	-0.0107121
H	-1.6617979	-0.9882246	-2.6019724
C	-3.5816383	-1.9157677	-2.5503479
C	-4.8664296	-1.5390976	-0.5558104
H	-3.4612432	-2.3592320	-3.5361001
C	-4.7464627	-2.1264122	-1.8162328
H	-5.7728044	-1.6972342	0.0265521
H	-5.5514756	-2.7399763	-2.2133514

C 0.7768620 -1.0096921 -2.5153772
C 1.2008396 -2.3619842 -2.4743331
C 0.4030007 -0.4590371 -3.7641630
C 1.2416844 -3.0951512 -3.6693948
C 0.4831344 -1.1877185 -4.9418042
H 1.5442176 -4.1398027 -3.6330207
C 0.9031908 -2.5214596 -4.8908491
H 0.2032375 -0.7338670 -5.8882569
H 0.9491293 -3.1148303 -5.8001368
C -0.7100696 -1.0529215 2.4793286
C -1.2614175 -2.3585190 2.4321770
C -0.4171116 -0.4897166 3.7430120
C -1.5289670 -3.0267528 3.6330747
C -0.7209441 -1.1519223 4.9258114
H -1.9314440 -4.0370926 3.5959730
C -1.2847935 -2.4299192 4.8681139
H -0.5090928 -0.6861762 5.8840709
H -1.5119870 -2.9683212 5.7843954
H 0.0406270 0.4952056 3.7912051
H 0.0373213 0.5641888 -3.7996971
C -4.0569034 -0.1387481 1.3547698
H -3.2201487 -0.3618360 2.0261603
H -4.1328243 0.9528090 1.2943100
H -4.9734888 -0.5217225 1.8140970
C 4.2125491 -0.1298436 -1.3305251
H 3.4038470 -0.4204212 -2.0122402
H 4.2112158 0.9650817 -1.2849603
H 5.1611305 -0.4539509 -1.7695272
C 1.5427110 -3.0690090 -1.1850783
H 2.5631938 -3.4670745 -1.2186433
H 1.4709538 -2.4096215 -0.3193974
H 0.8665531 -3.9171438 -1.0270074
C -1.4867399 -3.0782847 1.1252406
H -2.5298411 -3.3942513 1.0178292
H -1.2380965 -2.4510999 0.2671404
H -0.8618014 -3.9783676 1.0799233
C 1.7563415 1.8378797 -0.1701912
C 1.8289159 2.8204754 0.8460430
C 1.7762344 2.2792667 -1.5081631
C 1.8911372 4.1726725 0.4921076
C 1.8440155 3.6284637 -1.8543741
H 1.7804361 1.5411896 -2.3124636
C 1.8913323 4.5836034 -0.8420113
H 1.9423086 4.9194799 1.2818652
H 1.8577236 3.9262976 -2.8994684
H 1.9362685 5.6420619 -1.0846976
C -1.6926076 1.8859225 0.1825715
C -1.8651570 2.8520273 -0.8373818
C -1.7523695 2.3276813 1.5181607
C -2.0752479 4.1911148 -0.4901055
C -1.9731764 3.6614839 1.8581188
H -1.6603496 1.5973356 2.3229485
C -2.1299227 4.6024753 0.8423753
H -2.2017102 4.9253780 -1.2833845
H -2.0190871 3.9609580 2.9020587
H -2.2974856 5.6494002 1.0821863
C 1.8157112 2.4404954 2.3022964
H 0.8220622 2.0752701 2.5901054
H 2.5317813 1.6393295 2.5147387
H 2.0439419 3.3019797 2.9362959
C -1.8029354 2.4668125 -2.2910129
H -2.4644355 1.6212864 -2.5088823
H -2.0745999 3.3081950 -2.9350059
H -0.7838832 2.1604160 -2.5568139

TSAd : TS, BH/BB dimerization of two A
90
Energy = -1727.472495438
B 0.0065461 0.2060162 -1.5918673
B -0.4188691 1.3454077 -2.7505597
B 2.0521372 -0.5911797 -0.6862933
H 1.1479238 -0.2719730 -1.8544505
B 1.9099401 0.7817758 0.3275240
H 2.9868018 -0.4020626 -1.4376977
C 2.3900824 0.6927061 1.8262501
C 1.5388507 0.3422103 2.8880535
C 3.7495606 0.9539096 2.1234225
C 1.9953204 0.2664014 4.2035700
H 0.4916829 0.1304863 2.6875991
C 4.1996091 0.8682646 3.4454759
C 3.3347717 0.5306569 4.4875273
H 1.3061133 0.0012940 5.0013751
H 5.2474345 1.0719901 3.6584714
H 3.7046443 0.4727156 5.5078322
C -0.9549885 -0.5922710 -0.6647721
C -1.4819331 -1.8354621 -1.0764666
C -1.2968436 -0.1046176 0.6087674
C -2.3171541 -2.5458682 -0.2071876
C -2.1117549 -0.8300141 1.4739721
H -0.8973968 0.8521327 0.9353010
H -2.7121947 -3.5083225 -0.5257099
C -2.6283936 -2.0590513 1.0607073
H -2.3411441 -0.4397049 2.4618642
H -3.2670503 -2.6357515 1.7240674
C 1.7680829 -2.1108487 -0.3709442
C 2.0411825 -3.1299965 -1.3207256

C	1.2240676	-2.5031583	0.8647327
C	1.7579144	-4.4649784	-1.0091815
C	0.9472212	-3.8338798	1.1713487
H	1.0061050	-1.7397091	1.6026427
H	1.9609938	-5.2364320	-1.7498921
C	1.2126903	-4.8229007	0.2252557
H	0.5154384	-4.0949902	2.1342528
H	0.9963521	-5.8663546	0.4417018
C	-1.0863982	2.7288316	-2.5245319
C	-1.1204480	3.7805418	-3.4823426
C	-1.7399624	2.9413331	-1.2929582
C	-1.7965660	4.9636829	-3.1727180
C	-2.4424700	4.1100301	-1.0093888
H	-1.7126426	2.1504432	-0.5507918
C	-2.4649985	5.1306410	-1.9571636
H	-1.8016216	5.7732581	-3.8996008
H	-2.9463581	4.2310782	-0.0543530
H	-2.9895030	6.0603990	-1.7518067
C	-0.2694757	0.6470278	-4.1513761
C	1.0022175	0.2434160	-4.6029494
C	-1.3998240	0.3266306	-4.9450414
C	1.1754462	-0.3980384	-5.8285405
H	1.8749326	0.4534569	-3.9895022
C	-1.2158941	-0.3456733	-6.1556423
C	0.0587602	-0.6977334	-6.6076619
H	2.1710021	-0.6788565	-6.1612503
H	-2.0873212	-0.6009537	-6.7553191
H	0.1739333	-1.2158207	-7.5559329
C	1.6259165	2.1950165	-0.3082588
C	1.0695150	3.3345462	0.3322402
C	2.1427998	2.3765588	-1.6188539
C	1.0271584	4.5550528	-0.3527388
C	2.1520157	3.6090977	-2.2606200
H	2.6003818	1.5250559	-2.1119227
C	1.5693624	4.7059323	-1.6276723
H	0.5608575	5.4095985	0.1311977
H	2.5916924	3.7081263	-3.2487375
H	1.5288709	5.6717407	-2.1227905
C	-0.4254770	3.6793703	-4.8181746
H	0.5882612	3.2823731	-4.7151679
H	-0.9604993	3.0053113	-5.4965572
H	-0.3697061	4.6636156	-5.2926371
C	-2.7949442	0.6834956	-4.4877158
H	-2.9780179	0.3473583	-3.4605465
H	-2.9489239	1.7688568	-4.4962865
H	-3.5460383	0.2254403	-5.1374918
C	-1.1591935	-2.4001835	-2.4378923
H	-2.0115880	-2.2975844	-3.1207028
H	-0.3139414	-1.8793441	-2.8992037
H	-0.9080503	-3.4626047	-2.3696306
C	2.6114722	-2.8045222	-2.6805564
H	1.9628933	-2.1157666	-3.2346749
H	3.5874919	-2.3125854	-2.5962066
H	2.7314116	-3.7131791	-3.2783228
C	0.5097860	3.2942108	1.7323897
H	-0.1618112	2.4462584	1.8904154
H	1.3137118	3.1955723	2.4696001
H	-0.0451240	4.2119908	1.9460250
C	4.7156387	1.3160908	1.0189621
H	4.3690298	2.1948021	0.4621320
H	5.7101470	1.5333913	1.4192987
H	4.8103980	0.4974030	0.2947062

TSAt : TS, aryl-shift of adduct **A**·THF
58
Energy = -1096.323451883

B	-0.4146782	-0.1941329	-0.2446257
C	0.0719376	-1.4655839	0.6560087
C	0.2806609	-2.6428737	-0.1028358
C	0.6828386	-3.8560338	0.4428460
C	0.8939467	-3.9458324	1.8224416
C	0.6624391	-2.8214189	2.6063974
C	0.2446309	-1.5930760	2.0571714
C	-0.0118277	-0.4787347	3.0516472
H	-0.5941811	-0.8604643	3.8988443
H	-0.5519103	0.3536877	2.6061055
H	0.9273391	-0.0843415	3.4610169
H	0.7939282	-2.8885630	3.6861043
H	1.2168864	-4.8786704	2.2785086
H	0.8341729	-4.7230270	-0.1968662
H	0.1162928	-2.5821718	-1.1781025
C	-1.9156201	-0.4785670	-0.8064042
C	-2.0874821	-0.8915426	-2.1406940
C	-3.3316055	-1.2144613	-2.6870074
C	-4.4722554	-1.1292974	-1.8895832
C	-4.3361264	-0.7356412	-0.5570199
C	-3.0855239	-0.4228633	-0.0085123
C	-3.0111040	-0.0274636	1.4443040
H	-2.4930680	-0.7978064	2.0267308
H	-4.0106532	0.1102790	1.8695006
H	-2.4432437	0.9006451	1.5749346
H	-5.2208582	-0.6684174	0.0752169
H	-5.4533088	-1.3673086	-2.2941634
H	-3.4092442	-1.5243821	-3.7272615
H	-1.2050322	-0.9528361	-2.7762824
B	0.6135650	0.4045380	-1.4231735

C -0.0301813 1.3508617 0.2813681
C 1.1357490 1.5116478 1.0723914
C 1.5995957 2.7408455 1.5228249
C 0.9024861 3.9001144 1.1740057
C -0.2276166 3.7932554 0.3662820
C -0.6967773 2.5558383 -0.1006319
C -1.9067030 2.5626842 -0.9998894
H -1.7409204 1.9578639 -1.8955879
H -2.1496825 3.5871054 -1.2992427
H -2.7796141 2.1307771 -0.4989070
H -0.7606263 4.6956700 0.0726292
H 1.2426989 4.8750767 1.5131239
H 2.4922076 2.7965064 2.1408850
H 1.6849735 0.6176613 1.3540072
H 0.3338747 1.1770713 -2.2947990
O 2.0933691 0.2528484 -1.4649233
C 2.9854200 1.3935155 -1.8857533
C 4.3113749 1.0515790 -1.2365288
H 4.3461093 1.4416999 -0.2151163
C 4.3147087 -0.4869245 -1.2261511
H 4.5359408 -0.8797698 -2.2229164
H 5.0370381 -0.8991564 -0.5189666
C 2.8968865 -0.8340594 -0.8202330
H 2.5014029 -1.7674539 -1.2116885
H 2.7153572 -0.7690023 0.2521517
H 5.1467165 1.4693415 -1.8019997
H 3.0038327 1.3688889 -2.9757134
H 2.5042640 2.2966997 -1.5153591

TSA : TS, BH/BC addition of HBcat and **1**
72
Energy = -1541.553870907
B -0.1372588 0.0809628 -0.2663370
C 1.1777882 -0.8343837 -0.5772305
C -1.0623355 0.4975811 -1.5075704
B 0.0725553 1.4041360 0.8013058
C 1.3677695 -1.3755220 -1.8661844
C 2.2431311 -0.9709312 0.3577682
C -2.4775741 0.4716098 -1.5606260
C -0.3853055 1.0992354 -2.5884526
C 0.8309451 2.5343556 -0.0018557
C -0.4798117 1.6220410 2.2530345
C 2.5534633 -1.9969199 -2.2451016
H 0.5510217 -1.3103849 -2.5781725
C 3.4280342 -1.5991922 -0.0320829
C -3.1385812 0.9938124 -2.6815365
C -1.0523901 1.6352495 -3.6865147
H 0.6983691 1.1678780 -2.5521242
C 2.1822788 2.4197012 -0.3722520
C 0.1048355 3.6542860 -0.4803774
C -0.0326962 2.6480776 3.1396312
C -1.4688941 0.7417137 2.7438310
C 3.5942547 -2.1033855 -1.3231955
H 2.6626169 -2.3992814 -3.2483533
H 4.2353764 -1.6961221 0.6901437
C -2.4441309 1.5736008 -3.7412056
H -4.2260414 0.9587552 -2.7081494
H -0.4873748 2.0997466 -4.4906183
C 2.8193960 3.3881893 -1.1484990
H 2.7549400 1.5545642 -0.0511796
C 0.7484265 4.6094102 -1.2719687
C -0.5990753 2.7484925 4.4160812
C -2.0401901 0.8620040 4.0070124
H -1.8096293 -0.0705294 2.1106695
H 4.5272557 -2.5869313 -1.6002181
H -2.9847341 1.9815223 -4.5914860
H 3.8692592 3.2723999 -1.4058201
C 2.0991241 4.4914126 -1.6034168
H 0.1760627 5.4597049 -1.6381591
C -1.6005495 1.8801017 4.8512201
H -0.2439463 3.5286314 5.0859558
H -2.8082980 0.1657876 4.3316942
H 2.5787871 5.2493826 -2.2172990
H -2.0228412 1.9933229 5.8462754
C 1.0576362 3.6327162 2.7888398
H 1.9527353 3.1312000 2.4091123
H 0.7376059 4.3274649 2.0060779
H 1.3326063 4.2143903 3.6736587
C -1.3653907 3.8065155 -0.1738740
H -1.5547148 3.7682821 0.9052092
H -1.7502138 4.7546920 -0.5603884
H -1.9399928 2.9924059 -0.6324410
C -3.3241253 -0.0679015 -0.4326066
H -3.0831434 0.4278647 0.5147232
H -4.3848342 0.1037624 -0.6366001
H -3.1832136 -1.1451999 -0.2900041
C 2.1200989 -0.4704075 1.7724227
H 2.0698122 0.6271917 1.8087144
H 1.2143699 -0.8462579 2.2559329
H 2.9857626 -0.7731422 2.3686652
C -0.1667415 -4.0198323 0.6134325
C -0.8140215 -3.9374554 -0.6219496
C -1.0192024 -5.0487874 -1.4200685
C -0.5405039 -6.2699678 -0.9211435
C 0.1067511 -6.3521440 0.3174556
C 0.3067278 -5.2174146 1.1186639

H	-1.5211687	-4.9769782	-2.3790368
H	-0.6741661	-7.1701871	-1.5131735
H	0.4652907	-7.3149362	0.6685271
H	0.8080518	-5.2736685	2.0790338
O	-1.1858308	-2.6241106	-0.8626681
O	-0.1095729	-2.7594901	1.1897431
B	-0.6323028	-1.9112687	0.2062426
H	-1.0680536	-0.8104851	0.5506461

TSB0 : TS, BH/BC addition of HBcat and **B**
59
Energy = -1271.010801199

B	-0.0774293	0.0425520	-0.1818522
C	1.2171508	-0.8345679	-0.5290307
B	0.0697567	1.4004610	0.8145406
C	1.3733572	-1.3122655	-1.8457034
C	2.2940451	-1.0175800	0.3796542
C	0.8041819	2.5227727	-0.0204245
C	-0.4894826	1.6581004	2.2541603
C	2.5466135	-1.9286422	-2.2739953
H	0.5453479	-1.1939944	-2.5396358
C	3.4629515	-1.6448232	-0.0567695
C	2.1603155	2.4255130	-0.3791635
C	0.0659886	3.6339341	-0.5015960
C	0.0010812	2.6570008	3.1457131
C	-1.5690799	0.8663124	2.7030983
C	3.5974097	-2.0947324	-1.3724140
H	2.6392949	-2.2794486	-3.2981015
H	4.2836228	-1.7827624	0.6437323
C	2.7941228	3.4125587	-1.1340989
H	2.7385215	1.5643459	-0.0565715
C	0.7059582	4.6059392	-1.2769145
C	-0.5966048	2.8086029	4.4021172
C	-2.1752537	1.0449311	3.9436670
H	-1.9686458	0.0996577	2.0457309
H	4.5180051	-2.5795822	-1.6864640
H	3.8493527	3.3162887	-1.3767522
C	2.0646129	4.5116168	-1.5855333
H	0.1283390	5.4540226	-1.6401395
C	-1.6790971	2.0242131	4.8028945
H	-0.2044425	3.5620733	5.0819100
H	-3.0154233	0.4229124	4.2396619
H	2.5432591	5.2847933	-2.1808166
H	-2.1246884	2.1749966	5.7827202
C	1.1697602	3.5549736	2.8135202
H	2.0353864	2.9847633	2.4626680
H	0.9191999	4.2608379	2.0151009
H	1.4662634	4.1267887	3.6977480
C	-1.4070186	3.7701460	-0.1932010
H	-1.5909029	3.7343328	0.8865533
H	-1.8039339	4.7126483	-0.5811763
H	-1.9823939	2.9493934	-0.6407817
C	2.1954990	-0.5480997	1.8078642
H	2.1391996	0.5482858	1.8644421
H	1.2976328	-0.9362283	2.2975602
H	3.0706045	-0.8602234	2.3850072
C	-0.1596175	-4.0681826	0.6393807
C	-0.8418695	-3.9619342	-0.5740815
C	-1.0325327	-5.0480085	-1.4094641
C	-0.4979583	-6.2684420	-0.9700984
C	0.1860663	-6.3745587	0.2471596
C	0.3693830	-5.2654852	1.0863814
H	-1.5627641	-4.9584136	-2.3514870
H	-0.6171915	-7.1501457	-1.5921696
H	0.5868659	-7.3368136	0.5506041
H	0.8987184	-5.3396054	2.0302526
O	-1.2568584	-2.6473204	-0.7554735
O	-0.1264357	-2.8246664	1.2587275
B	-0.7196598	-1.9653883	0.3351164
H	-1.0631688	-0.8404983	0.6643677
H	-0.8438854	0.1576841	-1.1035545

TSB : TS, BH/BC addition of **B** and **4**
59
Energy = -1271.006197904

B	-0.0810895	0.2032258	-0.2418220
C	-0.6527820	-1.7524195	-0.4686684
B	1.2227275	0.0910953	0.8707526
C	-0.0940192	-2.1430056	-1.7191941
C	-0.4042457	-2.5802693	0.6776233
C	2.6483251	-0.0717093	0.2239074
C	1.0458480	0.3348952	2.4066358
C	0.6599458	-3.2964274	-1.8531259
H	-0.2905341	-1.5195952	-2.5848736
C	0.3773103	-3.7269528	0.5178424
C	3.1390174	-1.3000330	-0.2442287
C	3.4524579	1.0796396	0.0399451
C	1.9749025	-0.0437338	3.4192371
C	-0.1008283	1.0554929	2.8144626
C	0.8915597	-4.0925155	-0.7266905
H	1.0683679	-3.5766131	-2.8186102
H	0.5742731	-4.3531167	1.3838844
C	4.3927042	-1.4113317	-0.8458412
H	2.5367587	-2.1940691	-0.1197356
C	4.7010151	0.9629641	-0.5801005
C	1.7200806	0.3073199	4.7493948

C	-0.3289167	1.4254150	4.1367272
H	-0.8258725	1.3387421	2.0568753
H	1.4834655	-4.9991908	-0.8154493
H	4.7482146	-2.3816828	-1.1844651
C	5.1815770	-0.2737093	-1.0161188
H	5.3071086	1.8567610	-0.7183860
C	0.5896443	1.0418431	5.1130206
H	2.4248044	-0.0007583	5.5189818
H	-1.2158574	1.9928066	4.4052938
H	6.1577778	-0.3440497	-1.4889780
H	0.4274028	1.3044184	6.1552904
C	3.2247054	-0.8398647	3.1274521
H	3.0078952	-1.7319325	2.5330445
H	3.9467220	-0.2507106	2.5522026
H	3.6995929	-1.1511276	4.0626900
C	2.9608741	2.4299747	0.5084849
H	2.7945008	2.4356998	1.5923988
H	3.6785986	3.2193903	0.2663122
H	2.0015607	2.6864002	0.0393631
C	-1.0202559	-2.3041631	2.0189801
H	-1.1203031	-1.2369779	2.2149157
H	-2.0272493	-2.7379908	2.0533665
H	-0.4240366	-2.7608461	2.8134186
C	-4.0772865	-0.3018639	-0.1740195
C	-3.7753324	-0.0180745	-1.5090667
C	-4.7063306	0.5392923	-2.3690036
C	-5.9748532	0.8090557	-1.8300749
C	-6.2771259	0.5240013	-0.4940274
C	-5.3248124	-0.0442797	0.3676003
H	-4.4662591	0.7564346	-3.4046781
H	-6.7358716	1.2493419	-2.4672350
H	-7.2686237	0.7467168	-0.1115515
H	-5.5526911	-0.2695571	1.4042112
O	-2.4731671	-0.3912706	-1.7780843
O	-2.9748673	-0.8612092	0.4424582
B	-1.9501062	-0.8351672	-0.5361445
H	-1.1508592	0.6709991	0.1282906
H	0.1482376	0.4825115	-1.3847395

TSCd : TS, BH/BB dimerization of **C**
64
Energy = -1186.371635897

B	0.1419582	0.0004518	-1.6181834
B	-0.2673856	1.2458178	-2.6542675
B	2.0568754	-0.5567043	-0.6702335
H	1.2724264	-0.5224865	-1.8372876
B	1.9178033	0.9092580	0.1724679
H	3.0683131	-0.3694489	-1.3283567
C	-0.8845775	-0.8863629	-0.8486400
C	-1.4029260	-2.0594982	-1.4361875
C	-1.2846229	-0.5480073	0.4558197
C	-2.2782588	-2.8647832	-0.7001042
C	-2.1586160	-1.3560252	1.1787522
H	-0.8811291	0.3465831	0.9283070
H	-2.6623631	-3.7783282	-1.1510275
C	-2.6548714	-2.5236289	0.5984790
H	-2.4335909	-1.0843979	2.1946664
H	-3.3259701	-3.1705193	1.1575784
C	1.8764072	-2.0311724	-0.1172667
C	2.2433384	-3.1660316	-0.8843411
C	1.2787930	-2.2573770	1.1351004
C	1.9775324	-4.4509927	-0.3965238
C	1.0190188	-3.5390566	1.6187695
H	1.0028511	-1.4001474	1.7442099
H	2.2536684	-5.3152890	-0.9986306
C	1.3627661	-4.6444481	0.8419827
H	0.5371904	-3.6724477	2.5847151
H	1.1569333	-5.6526127	1.1950046
C	-0.9271477	2.6084442	-2.3553109
C	-0.9936694	3.6698944	-3.3016199
C	-1.6083116	2.7735470	-1.1287334
C	-1.7238276	4.8200658	-2.9884507
C	-2.3555310	3.9106194	-0.8392126
H	-1.5673064	1.9689769	-0.4000355
C	-2.4079305	4.9422785	-1.7767406
H	-1.7624541	5.6353383	-3.7083901
H	-2.8860020	3.9962714	0.1058243
H	-2.9780539	5.8442706	-1.5665855
C	1.8198020	2.3176795	-0.4708804
C	1.2471977	3.4412839	0.1791174
C	2.4035339	2.5113102	-1.7463296
C	1.2670353	4.6856473	-0.4557252
C	2.4537701	3.7654275	-2.3490636
H	2.8553001	1.6611336	-2.2526873
C	1.8715867	4.8560554	-1.7030413
H	0.8021289	5.5388916	0.0337853
H	2.9431653	3.8914630	-3.3115476
H	1.8804283	5.8379042	-2.1697040
C	-0.2948309	3.5806865	-4.6343715
H	0.7802568	3.4169335	-4.5044960
H	-0.6804295	2.7401787	-5.2243669
H	-0.4370817	4.5006526	-5.2097337
C	-1.0122471	-2.4410525	-2.8441610
H	-1.6933844	-1.9873636	-3.5764918
H	0.0016232	-2.0956044	-3.0824706
H	-1.0425848	-3.5258671	-2.9855949

C 2.9113390 -3.0158913 -2.2319270
H 2.3054299 -2.4178881 -2.9232884
H 3.8761555 -2.5016250 -2.1374200
H 3.0858865 -3.9936096 -2.6923706
C 0.6167970 3.3030956 1.5425219
H -0.1836441 2.5546707 1.5414613
H 1.3602382 2.9761735 2.2809677
H 0.1896657 4.2532131 1.8764794
H -0.1353716 0.9299259 -3.8152491
H 2.1437552 0.8664885 1.3586515

TSC : TS, aryl-shift of **B** forming **C**
32
Energy = -593.1624347250
B 0.7297784 2.4082881 -0.1637882
C 1.3428656 0.4656875 -0.0688400
C -1.4886353 0.4306510 0.0956894
C 2.0310263 0.2093686 -1.2733578
C 1.9282251 0.0313288 1.1562859
C -1.5246914 -0.9672674 0.2581559
C -2.7165835 1.1397436 0.0442841
C 3.2563930 -0.4566707 -1.2874116
H 1.5879914 0.5373113 -2.2097728
C 3.1398876 -0.6597919 1.1212701
C -2.7273647 -1.6603815 0.3847520
H -0.5848610 -1.5143742 0.2874296
C -3.9180737 0.4319891 0.1585525
H 3.7727416 -0.6325047 -2.2267549
C 3.8061982 -0.8986412 -0.0849012
H 3.5806513 -1.0019046 2.0549001
C -3.9302137 -0.9540921 0.3322654
H -2.7284955 -2.7389834 0.5165705
H -4.8596212 0.9754397 0.1163351
H 4.7570473 -1.4247178 -0.0797208
H -4.8776486 -1.4787395 0.4248072
B -0.1240907 1.1537700 0.0162242
H 1.5427940 3.2598498 -0.2170426
H 0.1457547 2.0381065 -1.2389370
C -2.7364354 2.6342566 -0.1468750
H -2.0802117 3.1273211 0.5797708
H -2.3666267 2.9082242 -1.1432728
H -3.7496822 3.0319719 -0.0407450
C 1.2351808 0.2836753 2.4688104
H 0.8428064 1.3062504 2.5110456
H 0.3773098 -0.3899368 2.5890091
H 1.9142534 0.1214327 3.3100962

TSD0 : TS, aryl-shift of **D** forming **D0**
64
Energy = -1186.432859681
B -0.1893664 -0.5998631 -0.3008919
B 0.0610677 1.1995999 -0.3724602
B 1.2275478 -1.4296292 -0.2394319
H 0.6740959 -1.1476701 -1.3633847
B 1.0542005 0.0918847 0.5858018
H 2.1304768 -0.5279371 -0.0359611
C -1.6270883 -1.1920057 -0.2115487
C -2.6919091 -0.9067301 -1.0964095
C -1.8682736 -2.0505395 0.8792141
C -3.9466472 -1.4778141 -0.8550489
C -3.1284908 -2.5936560 1.1207996
H -1.0450818 -2.2920300 1.5475430
H -4.7622451 -1.2635671 -1.5426280
C -4.1747360 -2.3057598 0.2447190
H -3.2873211 -3.2460373 1.9752533
H -5.1619315 -2.7296213 0.4093014
C 1.7813196 -2.8569966 0.0162605
C 1.2780702 -4.0004670 -0.6527180
C 2.8003990 -3.0329252 0.9727530
C 1.7934814 -5.2606941 -0.3297985
C 3.2997405 -4.2945805 1.2894223
H 3.2057149 -2.1609427 1.4828020
H 1.4034419 -6.1358917 -0.8452169
C 2.7917602 -5.4170520 0.6334974
H 4.0821422 -4.4000079 2.0361131
H 3.1737599 -6.4079396 0.8641252
C -1.1110477 2.0943583 0.2205899
C -1.4240731 3.3364271 -0.3824431
C -1.8826087 1.6855258 1.3196308
C -2.4740616 4.1113075 0.1258162
C -2.9269491 2.4604208 1.8227033
H -1.6603535 0.7325334 1.7929959
C -3.2248766 3.6836179 1.2220068
H -2.7051952 5.0647851 -0.3458715
H -3.5047489 2.1119518 2.6752476
H -4.0362291 4.3005945 1.6003644
C 1.6446713 1.7600706 -0.1793203
C 2.0209163 2.7105782 0.8114211
C 2.5012161 1.5947683 -1.2975188
C 3.2024770 3.4451799 0.6284851
C 3.6557323 2.3367498 -1.4673395
H 2.2302584 0.8527152 -2.0461869
C 4.0114974 3.2752597 -0.4898932
H 3.4862449 4.1683406 1.3895120
H 4.2786496 2.1891256 -2.3447351
H 4.9195960 3.8618225 -0.5976150

C	-0.6326447	3.8403565	-1.5652003
H	0.4208585	3.9927464	-1.3014353
H	-0.6451437	3.1138413	-2.3868015
H	-1.0369743	4.7879050	-1.9332211
C	-2.5130818	0.0024073	-2.2865954
H	-2.4057982	1.0435047	-1.9597896
H	-1.6147460	-0.2515541	-2.8594298
H	-3.3769215	-0.0604953	-2.9546502
C	0.2026979	-3.8790506	-1.7037084
H	-0.7142178	-3.4507794	-1.2835840
H	0.5200753	-3.2193417	-2.5212499
H	-0.0369608	-4.8567535	-2.1306895
C	1.1955459	2.9959126	2.0357168
H	0.3475893	3.6435893	1.7846365
H	0.7723418	2.0852971	2.4660042
H	1.8038367	3.5011128	2.7914818
H	-0.0235509	0.9955858	-1.5633443
H	1.1664710	0.1833115	1.7595697

TSE : TS, BB/HBH addition of **1** and **1R**

71

Energy = -1639.070217872

B	0.1469257	-0.7845537	-0.1631853
B	-0.0207715	0.7063272	0.7472782
C	-0.0108041	-2.1690555	0.5918625
C	0.5459653	-0.8136216	-1.7007363
C	-0.3264226	0.6151974	2.3268105
C	0.7988961	2.0260317	0.3284193
C	1.0165227	-3.1459995	0.5867999
C	-1.1618316	-2.4431075	1.3560372
C	0.0552396	-1.7772707	-2.6278755
C	1.4633649	0.1378914	-2.1885213
C	0.3776579	-0.3467025	3.0766416
C	-1.1682905	1.4997549	3.0500683
C	2.1909547	2.0957605	0.5999775
C	0.2266729	3.1370505	-0.3122404
C	0.8565678	-4.3259429	1.3255201
C	-1.3283723	-3.6336410	2.0574974
H	-1.9497224	-1.6959445	1.4001858
C	0.4713289	-1.7274410	-3.9643716
C	1.9030898	0.1531967	-3.5111601
H	1.8408591	0.8915140	-1.5073365
C	0.2761247	-0.4518166	4.4631974
H	1.0267861	-1.0412526	2.5538729
C	-1.2885398	1.3717752	4.4410450
C	2.9288299	3.2195846	0.2118139
C	0.9628774	4.2574865	-0.6987287
H	-0.8413728	3.1332155	-0.5149632
C	-0.3077188	-4.5848306	2.0481806
H	1.6621138	-5.0583542	1.3248117
H	-2.2392749	-3.8093766	2.6245222
C	1.3945109	-0.7809444	-4.4110844
H	0.0695094	-2.4550525	-4.6672133
H	2.6207732	0.9009346	-3.8393394
H	0.8470405	-1.2093103	4.9948913
C	-0.5746078	0.4086512	5.1543247
H	-1.9468309	2.0553057	4.9750636
C	2.3305992	4.3010482	-0.4374323
H	3.9976915	3.2408731	0.4175478
H	0.4696719	5.0866105	-1.2003433
H	-0.4112560	-5.5137148	2.6033711
H	1.7065783	-0.7747310	-5.4524602
H	-0.6808130	0.3370035	6.2341335
H	2.9255804	5.1610025	-0.7335067
B	-2.0621222	0.4771251	-0.4298770
H	-1.7741740	-0.6850645	-0.5787796
H	-3.1677725	0.6246533	0.0166564
H	-1.3207303	1.2128292	0.2603366
C	2.8997853	0.9531730	1.2809600
H	2.6292126	0.8908235	2.3408927
H	2.6113346	-0.0059542	0.8316343
H	3.9861437	1.0587392	1.2050618
C	-0.9136397	-2.8708162	-2.2377316
H	-0.4071490	-3.6630724	-1.6758595
H	-1.7165388	-2.5042462	-1.5906466
H	-1.3591287	-3.3199431	-3.1313363
C	2.3009983	-2.9539561	-0.1894447
H	2.1266841	-3.0115482	-1.2698128
H	2.7523862	-1.9742449	0.0002905
H	3.0306047	-3.7234693	0.0809252
C	-1.9528466	2.5980771	2.3713250
H	-2.7498297	2.1947221	1.7357040
H	-1.3060653	3.2052013	1.7298389
H	-2.4159916	3.2546530	3.1142551
S	-1.9467425	1.2674073	-2.1974934
C	-3.1652925	2.6116081	-2.1029757
H	-2.7698987	3.3640887	-1.4195512
H	-3.2757239	3.0370268	-3.1012260
H	-4.1115098	2.2194125	-1.7287950
C	-2.8916089	0.0751551	-3.1843911
H	-3.1019504	0.5362732	-4.1501946
H	-2.2489118	-0.7942938	-3.3149250
H	-3.8127415	-0.1887519	-2.6628860

TSF : TS, BH/BB addition of **9m** and **F**

71

Energy = -1639.068248565

B	-0.2204296	-0.8215927	0.4583590
B	-0.5044309	1.0574189	-0.3593961
C	0.4643377	-0.8061048	1.9115918
C	0.1716614	-2.0807363	-0.4801186
C	-0.5817081	2.4019836	0.5436328
C	0.6607067	1.0361178	-1.4717489
C	1.8467494	-1.0132855	2.1401965
C	-0.3625878	-0.6977811	3.0498838
C	-0.3754452	-3.3544863	-0.1578197
C	1.0883201	-2.0453574	-1.5445977
C	-0.5282534	2.4268190	1.9451499
C	-0.7476264	3.6535632	-0.1093815
C	1.9915215	1.4300111	-1.1949162
C	0.3528901	0.7050653	-2.8040240
C	2.3372649	-1.0213403	3.4552268
C	0.1279188	-0.7309141	4.3521086
H	-1.4347700	-0.5746348	2.9069287
C	-0.0489694	-4.4794826	-0.9276693
C	1.4133426	-3.1700120	-2.3021147
H	1.5850478	-1.1168715	-1.7870569
C	-0.6014402	3.6047725	2.6899703
H	-0.3997271	1.4937569	2.4791025
C	-0.8222910	4.8348535	0.6421119
C	2.9524800	1.3907747	-2.2171331
C	1.3037305	0.6887025	-3.8204627
H	-0.6685992	0.4241080	-3.0493508
C	1.4994282	-0.8782252	4.5587009
H	3.4064076	-1.1557229	3.6093551
H	-0.5531498	-0.6377118	5.1941015
C	0.8289657	-4.3989867	-2.0064202
H	-0.4862519	-5.4397756	-0.6604509
H	2.1278471	-3.0797626	-3.1167249
H	-0.5451488	3.5647245	3.7759806
C	-0.7435762	4.8262564	2.0348533
H	-0.9491411	5.7811801	0.1179483
C	2.6266766	1.0155825	-3.5196922
H	3.9786544	1.6672623	-1.9808012
H	1.0195471	0.4115588	-4.8323840
H	1.9109065	-0.8941012	5.5645924
H	1.0672041	-5.2858121	-2.5884267
H	-0.8003904	5.7572154	2.5941400
H	3.3931053	0.9914671	-4.2901446
B	-1.7822273	-0.1110632	0.1525316
H	-1.4601372	-1.1668656	0.8528251
H	-2.5199994	0.4894062	0.8693833
H	-1.5365802	1.1075980	-1.0387873
C	2.4136514	1.9429496	0.1621643
H	2.0987219	2.9856240	0.2894471
H	1.9555370	1.3794155	0.9774703
H	3.5025003	1.8983732	0.2679797
C	-1.2898406	-3.5744904	1.0279422
H	-0.9089823	-3.0829964	1.9291529
H	-2.3019845	-3.1876519	0.8538135
H	-1.3923065	-4.6439093	1.2340919
C	2.8340682	-1.2699551	1.0275132
H	2.7598533	-2.3072869	0.6818656
H	2.6569267	-0.6358539	0.1581804
H	3.8565764	-1.1009132	1.3799023
C	-0.8812506	3.7658852	-1.6108419
H	-1.7377812	3.1845382	-1.9768793
H	-0.0006874	3.3743647	-2.1288692
H	-1.0260192	4.8098979	-1.9062761
S	-2.8708215	-1.0570176	-1.2031825
C	-3.5432452	0.2926172	-2.2135350
H	-2.7842099	0.5733199	-2.9427806
H	-4.4238563	-0.0994018	-2.7246660
H	-3.7989702	1.1381018	-1.5728845
C	-4.3587697	-1.4316907	-0.2289589
H	-5.0946624	-1.8771778	-0.8991908
H	-4.0765492	-2.1481417	0.5427961
H	-4.7318454	-0.5120941	0.2237994

TSG : TS, BH/BB of **9m** and **F** forming **G**
71
Energy = -1639.081572667

B	0.5240635	-1.3179357	0.0992340
C	2.0069629	-1.9318737	0.1466861
C	2.4011153	-2.8001530	-0.8935645
C	3.6801532	-3.3426935	-0.9800986
C	4.6212620	-3.0291824	0.0041457
C	4.2525276	-2.1989380	1.0588725
C	2.9613474	-1.6523650	1.1537649
C	2.6381789	-0.8270722	2.3765960
H	1.7967056	-0.1581655	2.1998753
H	3.5046388	-0.2379988	2.6939756
H	2.3612973	-1.4802188	3.2146668
H	4.9800139	-1.9640310	1.8339401
H	5.6294339	-3.4324483	-0.0469454
H	3.9434322	-3.9984912	-1.8062160
H	1.6771532	-3.0476733	-1.6662454
C	-0.6935637	-2.3010085	-0.2028957
C	-0.9752237	-2.7750809	-1.4952953
C	-2.0338829	-3.6437074	-1.7584382
C	-2.8540304	-4.0600345	-0.7107352
C	-2.5946305	-3.6055375	0.5825420

C	-1.5285332	-2.7396111	0.8519367
C	-1.2869894	-2.2785547	2.2690468
H	-0.2938494	-2.5803091	2.6241344
H	-1.3297201	-1.1863799	2.3327934
H	-2.0353235	-2.6954053	2.9498563
H	-3.2385697	-3.9196371	1.4020821
H	-3.6934058	-4.7251256	-0.8976154
H	-2.2277026	-3.9772280	-2.7750642
H	-0.3697785	-2.4253654	-2.3299593
H	0.2810854	-0.4497790	0.9083229
B	-0.6455186	1.2815613	-0.3055808
C	-0.2087760	2.5942166	0.4497690
C	0.7977208	2.6081549	1.4308308
C	1.2370998	3.7913683	2.0252137
C	0.6885799	5.0088969	1.6236195
C	-0.3007626	5.0216076	0.6379745
C	-0.7636943	3.8377031	0.0536308
C	-1.8380757	3.8962649	-1.0083169
H	-1.5304011	3.3663516	-1.9185643
H	-2.0677956	4.9318572	-1.2761191
H	-2.7599278	3.4144224	-0.6626479
H	-0.7261583	5.9699802	0.3146898
H	1.0285626	5.9408750	2.0673350
H	2.0093364	3.7621789	2.7897890
H	1.2424307	1.6680944	1.7429569
C	-2.1011692	0.7162443	-0.1848372
C	-2.6228402	0.0133300	-1.2905113
C	-3.9048657	-0.5276317	-1.2996157
C	-4.6990563	-0.4028206	-0.1618757
C	-4.2061386	0.2729442	0.9555666
C	-2.9303310	0.8461378	0.9630080
C	-2.4785613	1.5571676	2.2177887
H	-3.1926943	1.3835875	3.0286860
H	-2.3974869	2.6380389	2.0579486
H	-1.4917776	1.2169251	2.5457648
H	-4.8248458	0.3529855	1.8473601
H	-5.6940479	-0.8400261	-0.1346865
H	-4.2660966	-1.0701718	-2.1684592
H	-1.9925614	-0.1086787	-2.1665954
B	0.4482175	0.5506323	-1.3925926
H	0.6084072	-0.6981705	-1.2141694
H	0.2470693	0.5838076	-2.5845243
S	2.2737141	1.1899577	-1.1223903
C	2.2006592	2.8379230	-1.8759099
H	3.2146221	3.2391071	-1.9078302
H	1.7764888	2.7592318	-2.8784255
H	1.5677965	3.4500677	-1.2328398
C	3.2468316	0.3167226	-2.3777981
H	2.7374586	0.3805179	-3.3409040
H	3.3213755	-0.7202140	-2.0466312
H	4.2374710	0.7725444	-2.4220467

TSHa : high TS, BH/BC addition of **9m** and **F**
71
Energy = -1639.067821907

B	0.9233787	0.0796802	-0.3217658
B	-1.0187534	-0.3395094	0.4761994
C	1.4562428	1.4465555	-0.9416916
C	1.9029423	-1.1528583	-0.3204087
C	0.0771882	0.2103377	1.5824729
C	-2.2170608	0.7160680	0.2816777
C	1.8858951	2.5899811	-0.2505405
C	1.6009824	1.4601630	-2.3592477
C	1.5195055	-2.3132260	-1.0174824
C	3.1920384	-1.1305504	0.2685959
C	0.6896304	-0.6219043	2.5773337
C	0.0173629	1.6024306	1.8639909
C	-2.6435030	1.4242685	-0.8629632
C	-2.9980683	0.8584990	1.4498011
C	2.3831542	3.7207181	-0.8976128
H	1.8418643	2.5956899	0.8322928
C	2.0911809	2.6037798	-3.0019063
C	2.3533101	-3.4232276	-1.1363302
H	0.5343906	-2.3398575	-1.4706011
C	4.0144458	-2.2578477	0.1654615
C	1.1685078	-0.0398825	3.7570681
C	0.4983057	2.1640590	3.0386084
H	-0.4583891	2.2441987	1.1306400
C	-3.7842954	2.2399540	-0.7958833
C	-4.1292498	1.6669639	1.5104431
H	-2.6944712	0.3178589	2.3441781
C	2.4765311	3.7365482	-2.2873572
H	2.6955921	4.5834259	-0.3143575
H	2.1736882	2.5970874	-4.0872218
C	3.6087684	-3.3981939	-0.5300824
H	2.0223470	-4.3022880	-1.6835232
H	4.9922428	-2.2414663	0.6430663
H	1.6190812	-0.6831868	4.5092924
C	1.0761305	1.3301934	3.9967112
H	0.4201562	3.2340706	3.2095076
C	-4.5284050	2.3722654	0.3737980
H	-4.0887268	2.7838822	-1.6886923
H	-4.6908302	1.7488886	2.4382469
H	2.8506376	4.6133315	-2.8096638
H	4.2689135	-4.2596201	-0.5941298
H	1.4536053	1.7431836	4.9285666

H	-5.4057413	3.0141108	0.3967401
B	-1.5789723	-1.9766338	0.5282213
H	-2.2107565	-2.1035065	1.5650225
H	-0.1914076	-0.2403300	-0.8394559
H	-0.8860080	-2.9401314	0.3000128
S	-3.0567340	-2.1950319	-0.7647678
C	-2.2239138	-2.6552960	-2.3081307
H	-1.6779219	-1.7789267	-2.6599442
H	-1.5310411	-3.4754867	-2.1078521
H	-2.9718365	-2.9437456	-3.0485820
C	-3.7162565	-3.8251581	-0.3228170
H	-4.2548417	-3.7046410	0.6181473
H	-4.3978009	-4.1592646	-1.1075056
H	-2.8844977	-4.5219352	-0.1977727
C	-1.9039787	1.3499438	-2.1747251
H	-2.4289108	1.9169573	-2.9495788
H	-0.8896261	1.7513572	-2.0858458
H	-1.8117283	0.3171454	-2.5276849
C	0.7740838	-2.1171488	2.4697489
H	0.9605864	-2.4485527	1.4497769
H	1.5601025	-2.4998022	3.1277625
H	-0.1795497	-2.5626202	2.7789511
C	1.2816244	0.2518302	-3.2072149
H	0.3276308	-0.2057603	-2.9257573
H	1.2327081	0.5229886	-4.2658878
H	2.0474275	-0.5228808	-3.0851058
C	3.6967275	0.0772625	1.0170163
H	2.9356885	0.4733648	1.6954712
H	4.5878738	-0.1719894	1.6011585
H	3.9569569	0.8845960	0.3224653

TSH : TS, BH/BC addition of **9m** and **F**
71
Energy = -1639.069033024

B	-1.2805520	-0.8319821	0.6086417
C	-1.2500035	-2.2999429	0.0898958
C	-1.9813772	-2.6947531	-1.0484922
C	-1.8706471	-3.9731142	-1.5883213
C	-1.0495996	-4.9131809	-0.9622508
C	-0.3555377	-4.5620051	0.1954464
C	-0.4345669	-3.2695571	0.7275468
C	0.3352974	-2.9585000	1.9919461
H	-0.1030428	-2.1264769	2.5481054
H	1.3730395	-2.6944792	1.7552107
H	0.3579080	-3.8313995	2.6522938
H	0.2596141	-5.3051563	0.6989680
H	-0.9620120	-5.9193449	-1.3637809
H	-2.4259797	-4.2382298	-2.4838513
H	-2.6266663	-1.9760215	-1.5433206
C	-2.5461062	0.0512587	0.8107433
C	-3.4646505	0.3193578	-0.2224588
C	-4.5512757	1.1694709	-0.0365326
C	-4.7638728	1.7554339	1.2109766
C	-3.8821263	1.4861875	2.2574160
C	-2.7761433	0.6494451	2.0774530
C	-1.8768520	0.3490963	3.2534989
H	-2.1131287	-0.6311161	3.6876194
H	-0.8228707	0.3215153	2.9544162
H	-1.9945678	1.0981746	4.0417685
H	-4.0480785	1.9398368	3.2325603
H	-5.6071306	2.4228250	1.3684020
H	-5.2190117	1.3850623	-0.8661059
H	-3.2963804	-0.0951741	-1.2107652
H	-0.2314890	-0.3693020	0.9781398
B	0.9659136	0.7443306	-0.6869874
C	2.0800217	1.1486765	0.3573244
C	2.3046978	0.4664991	1.5670279
C	3.3884386	0.7597047	2.3959488
C	4.3012337	1.7444494	2.0194750
C	4.1059102	2.4290233	0.8176542
C	3.0127775	2.1532147	-0.0105932
C	2.8390804	2.9167148	-1.3030944
H	2.7257734	2.2328341	-2.1530516
H	3.6972874	3.5681550	-1.4940897
H	1.9345066	3.5364671	-1.2761224
H	4.8168423	3.1953563	0.5129424
H	5.1568576	1.9760713	2.6483521
H	3.5229068	0.2135022	3.3265378
H	1.6136598	-0.3153811	1.8688235
C	-0.2512600	1.6818585	-1.0273568
C	-0.8654751	1.4935925	-2.2841247
C	-1.9102811	2.2940448	-2.7387764
C	-2.3997537	3.3014234	-1.9085104
C	-1.8331785	3.4963694	-0.6484112
C	-0.7640444	2.7171397	-0.1966913
C	-0.2092631	2.9959664	1.1772984
H	-0.8681980	3.6785070	1.7222605
H	0.7876413	3.4478465	1.1205235
H	-0.1005996	2.0766052	1.7566859
H	-2.2381328	4.2657675	0.0054404
H	-3.2319093	3.9229996	-2.2295603
H	-2.3518925	2.1222276	-3.7171028
H	-0.4995354	0.6881219	-2.9157382
B	1.2993583	-0.5810574	-1.7170034
H	0.3712232	-1.2781600	-2.0567572
H	1.9503289	-0.2075490	-2.6812365

S 2.4616490 -1.8765905 -0.8293741
C 4.1393355 -1.2141151 -1.0031973
H 4.8532384 -1.9685630 -0.6676390
H 4.3100209 -0.9431125 -2.0470200
H 4.1923025 -0.3302962 -0.3660202
C 2.5303256 -3.2436891 -2.0158651
H 2.7801597 -2.8546429 -3.0050727
H 1.5344740 -3.6891494 -2.0222388
H 3.2689557 -3.9744334 -1.6800867

TSIdh : TS, BH/BH dimerization of **I**
56
Energy = -1601.553361950
B 1.1822626 -0.3761153 0.3006031
B -1.6489978 1.6460552 0.6493072
B 1.6750431 -1.8624214 -0.3877987
H -0.5643148 0.0165456 -0.9696534
B -1.3513062 0.1028838 -0.0384731
H -2.1528176 1.6812499 1.7587125
H 1.7361548 -1.8252278 -1.6056611
H 0.3558612 -0.3447228 1.1995205
C 1.8913222 0.9847144 -0.0036438
C 2.2995205 1.8719656 1.0228571
C 2.1610303 1.3498890 -1.3368715
C 2.9679939 3.0556483 0.6913453
C 2.7906479 2.5492546 -1.6622866
H 3.2895791 3.7246075 1.4879050
C 3.2091595 3.4026825 -0.6393605
H 2.9676485 2.8121105 -2.7024945
H 3.7183031 4.3342657 -0.8743276
C -2.1880621 -1.1856727 0.2593965
C -2.2892054 -2.2889033 -0.6325758
C -2.8880435 -1.2676147 1.4821689
C -3.0675423 -3.3961088 -0.2746179
C -3.6402586 -2.3831095 1.8422175
H -3.1480517 -4.2331589 -0.9658149
C -3.7334170 -3.4540159 0.9514930
H -4.1551635 -2.4170568 2.7992322
H -4.3239657 -4.3309501 1.2060848
H -2.8209930 -0.4228942 2.1632304
H 1.8566348 0.6713527 -2.1308635
C 2.0159517 1.5488585 2.4701486
H 2.5273629 0.6332277 2.7911861
H 0.9414530 1.3837190 2.6148073
H 2.3388714 2.3634957 3.1257022
C -1.5893906 -2.2954862 -1.9706418
H -0.5060675 -2.2084481 -1.8380366
H -1.8020557 -3.2242033 -2.5092038
H -1.9084261 -1.4550960 -2.5967995
S -3.0107058 2.4390151 -0.4990641
S 3.5063699 -2.2124519 0.1908992
C -4.5270231 1.5098447 -0.1518857
H -5.3685650 2.0068289 -0.6375175
H -4.3889147 0.5085927 -0.5623019
H -4.6693007 1.4509845 0.9291138
C -3.4233721 4.0096126 0.3079930
H -3.6864368 3.8142671 1.3495911
H -2.5269089 4.6291666 0.2557095
H -4.2458422 4.4871148 -0.2283208
C 3.9506589 -3.7340205 -0.6892147
H 3.3084410 -4.5216316 -0.2929183
H 3.7644504 -3.5948498 -1.7560330
H 4.9994509 -3.9670296 -0.4950520
C 4.5711422 -1.0333289 -0.6797329
H 4.3267243 -1.0460832 -1.7435640
H 4.3644946 -0.0474726 -0.2630606
H 5.6119353 -1.3128129 -0.5069445
H -0.7485493 2.4473390 0.5126312
H 1.1201172 -2.8622345 0.0188067

TSIds : TS, dimerization of **K** via SMe_2 release
65
Energy = -2079.678260316
B 0.2277994 0.7084958 -0.2595114
C -1.0131692 1.4181739 -0.9462680
B 1.5595649 1.3643628 0.5984308
H 0.3949255 -0.5062318 -0.6017745
H -1.0148579 0.4328269 1.5692885
S 1.4416197 0.6382078 -2.8161054
C -0.9252948 2.8241001 -1.0454815
C -2.1782853 0.8196738 -1.4977660
H 1.8604343 0.7620038 1.5990306
S 1.3281775 3.2021232 1.2622122
H 2.5438443 1.5420672 -0.0954471
B -0.9324040 -0.7836706 1.5208000
C 3.0268820 -0.1642881 -2.4539184
C 1.9948013 2.3564474 -2.9781227
C -1.9119121 3.6215661 -1.6204854
H -0.0293690 3.3178166 -0.6816154
C -3.1763082 1.6290071 -2.0586839
C -2.3910884 -0.6691464 -1.5541821
C -0.0906114 3.1557605 2.3841407
C 2.6800364 3.3620927 2.4556484
B 0.3009394 -1.4217545 0.4477850
S -0.5621480 -1.2951835 3.3695087
H -2.0442645 -1.2640068 1.4181453

H	2.8030249	-1.1979531	-2.1815265
H	3.6815536	-0.1430401	-3.3287109
H	3.4978046	0.3452149	-1.6092111
H	2.5167396	2.6554205	-2.0648995
H	1.0991844	2.9665983	-3.1099563
H	2.6490237	2.4686375	-3.8462848
C	-3.0616000	3.0164695	-2.1240758
H	-1.7835039	4.6999481	-1.6692872
H	-4.0677245	1.1491728	-2.4589072
H	-3.4595769	-0.9000319	-1.6195023
H	-1.9080963	-1.0893151	-2.4441345
H	-1.9733770	-1.1812322	-0.6885941
H	-0.9655124	2.9341578	1.7713197
H	0.0586684	2.3656020	3.1210188
H	-0.1954445	4.1271477	2.8700123
H	3.6072755	3.3277974	1.8814888
H	2.6433082	2.5258423	3.1567686
H	2.5922475	4.3195664	2.9722928
C	0.1182717	-2.7126036	-0.4969993
H	1.4240031	-1.3915166	0.9071552
C	1.0493540	-0.6420173	3.8668633
C	-1.6549385	-0.2437664	4.3602017
H	-3.8563502	3.6120449	-2.5663582
C	0.2034199	-2.6563248	-1.8999657
C	-0.0471235	-4.0014119	0.0724405
H	1.0872728	0.4326514	3.6913021
H	1.7922287	-1.1389090	3.2424772
H	1.2095342	-0.8780425	4.9203461
H	-2.6758917	-0.5220625	4.0944792
H	-1.4804091	0.8051629	4.1137567
H	-1.4762494	-0.4357878	5.4200362
C	0.1134431	-3.7847519	-2.7162297
H	0.3322475	-1.6870550	-2.3768480
C	-0.1352538	-5.1348281	-0.7458290
C	-0.1225514	-4.1723894	1.5691773
C	-0.0612863	-5.0408462	-2.1369929
H	0.1815299	-3.6808904	-3.7970106
H	-0.2616942	-6.1107852	-0.2787933
H	-0.1144695	-5.2310012	1.8483672
H	0.7218185	-3.6785516	2.0665714
H	-1.0338063	-3.7169690	1.9721924
H	-0.1334615	-5.9336631	-2.7533097

TSId : TS, BB/BB dimerization of **I**
56
Energy = -1601.571485532

B	0.9160207	0.3215568	1.1929725
H	1.0001299	1.1420010	2.0916915
B	-0.5063522	-0.2087232	-0.7230067
B	0.4675497	1.1929409	-0.6889650
B	-0.5264238	-0.6280857	1.4887376
H	0.0255974	-1.2156477	-1.1124361
H	1.1282510	1.4494435	0.3542757
H	-0.0046107	2.2795170	-0.9212733
S	1.8713091	0.8596467	-1.9753991
H	-1.1280274	-1.3382686	0.7219817
H	-0.2110546	-1.3385244	2.4199008
S	-1.9026265	0.5831863	2.1601918
C	0.8662829	0.7619460	-3.4816006
C	2.7245565	2.4329319	-2.2879358
C	-3.4138263	-0.4109423	2.2573178
C	-1.4701317	0.7156178	3.9144404
H	0.2547769	-0.1364699	-3.3847814
H	0.2315619	1.6470143	-3.5546514
H	1.5317716	0.6783555	-4.3421509
H	1.9871168	3.2243305	-2.4346065
H	3.3365375	2.6428572	-1.4096330
H	3.3635295	2.3206690	-3.1660459
H	-4.1869407	0.1600508	2.7750784
H	-3.7109741	-0.6029379	1.2242542
H	-3.2000267	-1.3476486	2.7758829
H	-1.4390530	-0.2810532	4.3580665
H	-0.4810750	1.1754193	3.9497463
H	-2.2016258	1.3523641	4.4147769
C	2.2723304	-0.4990091	0.9548856
C	3.5477314	0.1093255	1.0761386
C	2.2385736	-1.8579454	0.6005989
C	4.7080631	-0.6350924	0.8255273
C	3.3958568	-2.6008164	0.3609119
H	1.2694823	-2.3402736	0.4989731
C	4.6420047	-1.9828341	0.4678535
H	5.6790843	-0.1502841	0.9153376
H	3.3265965	-3.6511644	0.0864101
H	5.5550336	-2.5424215	0.2783493
C	-2.0665213	-0.0913576	-1.0424523
C	-2.8595431	-1.2262754	-1.3673847
C	-2.7210067	1.1524793	-0.9834004
C	-4.2326617	-1.0781975	-1.5965608
C	-4.0928709	1.2951319	-1.2040637
H	-2.1297516	2.0328249	-0.7467829
C	-4.8557935	0.1697878	-1.5110742
H	-4.8269286	-1.9574973	-1.8397830
H	-4.5606844	2.2744455	-1.1355848
H	-5.9255983	0.2571907	-1.6848104
C	3.6841780	1.5570866	1.4860165
H	3.3400334	1.7083512	2.5161979

H	4.7254769	1.8870996	1.4177688
H	3.0714325	2.2181177	0.8615848
C	-2.2533919	-2.6056696	-1.4476682
H	-3.0198342	-3.3550385	-1.6688164
H	-1.4800531	-2.6569401	-2.2217599
H	-1.7639269	-2.8737929	-0.5031315

TSI : TS, BH/BC addition of **I** and **2**
71
Energy = -1639.072249467

B	1.0374698	0.0996879	-0.0633409
B	-1.0402829	-0.3799883	0.1555208
C	1.5613255	1.3894412	-0.8790958
C	1.9423646	-1.1898193	-0.3472680
C	0.4346081	0.3683233	1.4410178
C	-2.1956293	0.7094696	0.1664466
C	2.0643028	2.5136317	-0.1972528
C	1.6817561	1.3949609	-2.2946047
C	1.5022884	-2.2743914	-1.1216291
C	3.2759097	-1.2282082	0.1306652
C	0.6547215	-0.4841665	2.5685835
C	-0.0240161	1.6872385	1.7158226
C	-2.5779132	1.5367487	-0.9152630
C	-2.9682126	0.7825953	1.3445949
C	2.6118371	3.6168396	-0.8501929
H	2.0404639	2.5229540	0.8886716
C	2.2128772	2.5160822	-2.9466564
C	2.3139896	-3.3700029	-1.4087163
H	0.4857252	-2.2612800	-1.5008907
C	4.0873373	-2.3330950	-0.1575700
C	0.4473069	0.0199009	3.8597197
C	-0.2311432	2.1692958	2.9958374
H	-0.2486670	2.3306072	0.8733082
C	-3.6763666	2.3983237	-0.7769955
C	-4.0438864	1.6528777	1.4818419
H	-2.6912282	0.1448286	2.1800880
C	2.6721114	3.6274380	-2.2422480
H	2.9859129	4.4594285	-0.2739659
H	2.2660054	2.5106501	-4.0337750
C	3.6198400	-3.4034795	-0.9195314
H	1.9297536	-4.1908516	-2.0095779
H	5.1048604	-2.3526419	0.2292198
H	0.6174485	-0.6377122	4.7088334
C	0.0190499	1.3255796	4.0814073
H	-0.5914010	3.1815790	3.1510577
C	-4.4040737	2.4713417	0.4090225
H	-3.9585754	3.0277353	-1.6189105
H	-4.5984149	1.6932672	2.4161695
H	3.0812415	4.4824165	-2.7742065
H	4.2689420	-4.2499030	-1.1294655
H	-0.1367554	1.6781832	5.0977157
H	-5.2447164	3.1555137	0.4926859
B	-1.4748412	-1.9829266	0.6179566
H	-1.9256499	-2.0363446	1.7452623
H	-0.2340548	-0.2475012	-0.8272247
H	-0.7724721	-2.9303101	0.3541673
S	-3.1080547	-2.3408426	-0.4344748
C	-2.4752914	-2.5997200	-2.1122081
H	-2.1660956	-1.6251610	-2.4930537
H	-1.6215222	-3.2787443	-2.0729022
H	-3.2707627	-3.0036865	-2.7401542
C	-3.5155244	-4.0537765	-0.0073967
H	-3.8864317	-4.0431093	1.0187353
H	-4.2920620	-4.4136806	-0.6848684
H	-2.6152647	-4.6663658	-0.0778114
C	-1.8298691	1.5289133	-2.2251380
H	-2.3042733	2.1989053	-2.9480330
H	-0.7921992	1.8505197	-2.0890114
H	-1.7990651	0.5283260	-2.6701034
C	1.1118397	-1.9144947	2.4714401
H	0.8803682	-2.3606520	1.5082822
H	2.1979175	-1.9750030	2.6188091
H	0.6368692	-2.5038385	3.2621248
C	1.2736991	0.2210529	-3.1505644
H	0.3465762	-0.2410287	-2.8017229
H	1.1333337	0.5336838	-4.1897269
H	2.0407459	-0.5607088	-3.1271301
C	3.8580421	-0.1000472	0.9500028
H	3.1268627	0.3101989	1.6538261
H	4.7297119	-0.4425135	1.5165228
H	4.1754547	0.7286336	0.3056793

TSJ : TS, BH/BB addition of **1R** and **I**
41
Energy = -1305.566597917

B	0.1454814	0.1776295	-0.1349356
H	1.2724468	0.3111014	-0.5625564
B	-0.8094449	-1.4125900	-0.7454444
B	-1.0254323	0.9367496	-1.2189798
H	-0.6735597	-1.5688955	-1.9178983
H	-1.0949012	2.1014292	-0.8746128
H	-2.1273981	0.4807062	-1.4265617
S	-0.2636097	1.0202650	-3.0234006
C	-1.4629343	2.0078366	-3.9575092
C	1.0772417	2.2279245	-2.8776294
H	-2.3537869	1.3895719	-4.0779471

H	-1.7030400	2.9101906	-3.3909679
H	-1.0420558	2.2546701	-4.9340002
H	0.6858631	3.1458836	-2.4349024
H	1.8271993	1.7827520	-2.2230310
H	1.4976302	2.4133812	-3.8673628
C	-0.0453678	0.6110084	1.3958261
C	0.5664221	1.7920619	1.8893518
C	-0.8445578	-0.1164906	2.2961391
C	0.3688222	2.1857477	3.2192501
C	-1.0374688	0.2766445	3.6220837
H	-1.3367154	-1.0212485	1.9465864
C	-0.4254171	1.4387802	4.0918107
H	0.8498755	3.0952462	3.5766544
H	-1.6614987	-0.3211532	4.2829150
H	-0.5619638	1.7611485	5.1211712
C	1.4303309	2.6441841	0.9909775
H	0.8557615	3.0116743	0.1326913
H	1.8333183	3.5053167	1.5333646
H	2.2667350	2.0659728	0.5806183
H	0.2257196	-1.1374884	-0.0441231
H	-1.8482153	-1.5434846	-0.1836456
S	0.0085345	-3.5262566	-0.2786975
C	1.6460127	-3.3531267	-1.0371119
H	1.4953469	-3.3615545	-2.1179798
H	2.0949411	-2.4040140	-0.7319020
H	2.2776516	-4.1929228	-0.7424394
C	0.4654558	-3.4587427	1.4760078
H	0.9472705	-2.5035822	1.6994718
H	-0.4584806	-3.5508442	2.0491871
H	1.1307177	-4.2919796	1.7099290

TSKa : TS, SMe_2 addition to **J** forming **Ka**
37
Energy = -1278.874832705

B	0.2069332	-0.9355438	0.7555929
C	1.7399796	-0.5933241	0.4938188
C	2.4616890	-0.0009930	1.5527630
C	3.7900678	0.4099958	1.4342448
C	4.4632981	0.2166036	0.2270268
C	3.7844255	-0.3850462	-0.8346404
C	2.4453414	-0.7833817	-0.7241730
C	1.7762427	-1.4038279	-1.9264647
H	2.5137355	-1.6806843	-2.6870747
H	1.2029575	-2.2955454	-1.6467766
H	1.0615586	-0.7085779	-2.3836654
H	4.3055223	-0.5507056	-1.7770209
H	5.5014310	0.5197560	0.1137628
H	4.2990137	0.8677425	2.2801839
H	1.9500963	0.1436764	2.5036608
B	-0.9926815	-0.4601844	-0.2478058
S	-2.8319820	-0.9213718	0.1645785
C	-3.0286281	-2.6069923	-0.4866550
H	-4.0356794	-2.9510536	-0.2444480
H	-2.2684053	-3.2560850	-0.0507111
H	-2.9028388	-2.5421127	-1.5684253
C	-2.9078567	-1.2361063	1.9449179
H	-3.9088601	-1.6015712	2.1794858
H	-2.7275450	-0.2784814	2.4350018
H	-2.1361471	-1.9529755	2.2236269
H	-0.9803016	-0.3305276	-1.4398705
S	-1.5852513	2.0738192	-0.0610601
C	0.1557680	2.4361902	-0.4137798
H	0.7200923	2.1622542	0.4786172
H	0.4971253	1.8178774	-1.2471163
H	0.2813436	3.4986913	-0.6343710
C	-2.3288583	2.4613965	-1.6709723
H	-3.3863849	2.1966179	-1.6059382
H	-2.2309984	3.5266177	-1.8935141
H	-1.8479260	1.8634161	-2.4496832
H	-0.0647908	-1.1514021	1.9175429
H	-0.2230260	-1.9291682	0.0442870

TSK : TS, SMe_2 addition to **J** forming **K**
37
Energy = -1278.891661690

B	0.1053928	-0.9787471	-0.0716812
C	-1.4340161	-0.8179731	0.1630020
C	-1.8771926	-0.3550059	1.4202286
C	-3.2128001	-0.0475182	1.6752974
C	-4.1576564	-0.2338786	0.6652747
C	-3.7550099	-0.7214343	-0.5805303
C	-2.4122555	-1.0065302	-0.8520086
C	-2.0429038	-1.5192565	-2.2255381
H	-1.4163831	-2.4142063	-2.1692466
H	-1.4765500	-0.7698922	-2.7927644
H	-2.9432261	-1.7621709	-2.7984192
H	-4.4994436	-0.8758419	-1.3594190
H	-5.2059387	-0.0082209	0.8449541
H	-3.5166346	0.3264780	2.6498032
H	-1.1345785	-0.2132975	2.2012766
B	1.2061614	-1.3883334	1.1514553
H	1.4562369	-2.5852941	1.0860040
H	0.9638403	-1.0486451	2.2934015
S	2.9165895	-0.5149882	0.8252677
C	3.4266804	-1.0820627	-0.8169391
H	4.4316227	-0.7105462	-1.0238906

H	2.7119293	-0.6688625	-1.5283139
H	3.3957028	-2.1732716	-0.8482593
C	4.1151277	-1.4198504	1.8433758
H	5.1207866	-1.0569942	1.6216828
H	3.8627423	-1.2101961	2.8841764
H	4.0249026	-2.4889618	1.6408592
H	0.4573902	-0.9187496	-1.2271359
S	0.6250218	2.2381063	-0.1536229
C	1.6950019	2.4509815	-1.6069595
H	2.7261310	2.4561251	-1.2459829
H	1.5595585	1.6196547	-2.3037827
H	1.4816934	3.3999316	-2.1056041
C	-0.9976521	2.3429114	-0.9660258
H	-1.1387400	3.3277709	-1.4186467
H	-1.7524538	2.1726637	-0.1962359
H	-1.0740986	1.5564281	-1.7190117

TSN : TS, BH/BH of **9m** and **1R**
43
Energy = -1072.524008439

B	-0.9137378	0.1670064	0.0302784
C	-0.4136837	1.3611576	0.9640582
H	-0.5376975	-0.9263884	0.7169703
H	-2.1449648	0.0459762	0.0576103
C	-0.3440061	-0.0726922	-1.4411330
C	-0.3994193	2.6950217	0.4870346
C	0.0055434	1.1401623	2.2864654
B	-1.7384747	-1.3903254	0.8397980
C	-1.2384913	-0.3222237	-2.4953769
C	1.0393526	-0.0600022	-1.7386306
C	0.0379989	3.7282821	1.3254703
C	-0.8584772	3.0299274	-0.9131797
C	0.4425183	2.1725694	3.1176325
H	-0.0135613	0.1272909	2.6824618
H	-2.1972718	-2.0590571	-0.0299718
H	-2.2969292	-1.1317590	1.8572726
S	-0.4979330	-3.2391811	1.5984369
C	-0.8064461	-0.5460033	-3.8026604
H	-2.3054708	-0.3388607	-2.2806897
C	1.4696083	-0.2955137	-3.0509057
C	2.0717764	0.2035307	-0.6673769
C	0.4645253	3.4789635	2.6309743
H	0.0409095	4.7484466	0.9456579
H	-1.8509732	2.6113558	-1.1173559
H	-0.9038574	4.1136412	-1.0576063
H	-0.1819136	2.6086249	-1.6653797
H	0.7622754	1.9579685	4.1343578
C	0.3118492	-3.6199540	0.0199073
C	0.9130753	-2.5833731	2.5293617
C	0.5602256	-0.5335293	-4.0823372
H	-1.5280572	-0.7327610	-4.5942353
H	2.5367205	-0.2928828	-3.2659398
H	2.0446784	1.2485996	-0.3379609
H	1.8911996	-0.4022700	0.2284203
H	3.0788760	-0.0174911	-1.0331465
H	0.8035446	4.2977538	3.2604044
H	-0.4569105	-4.0339313	-0.6343685
H	0.7166824	-2.7067965	-0.4228248
H	1.0995025	-4.3575991	0.1823369
H	1.3477554	-1.7300922	2.0021376
H	0.5366385	-2.2688149	3.5041650
H	1.6605761	-3.3684501	2.6581313
H	0.9179425	-0.7108103	-5.0935307

TSPdh : TS, BH/BH dimerization of **P**
82
Energy = -2142.651672896

B	1.0485117	-0.2848407	0.0296014
B	-1.6530011	1.8568643	0.6209446
B	1.1679748	-1.8974128	-0.5415789
H	-0.3767702	0.4620999	-1.1216167
B	-1.2842309	0.4573526	-0.2984386
H	-2.0946983	1.5378671	1.7136640
H	1.0298837	-1.9512654	-1.7518625
H	0.3020393	-0.0132496	0.9642120
C	2.0756260	0.8398911	-0.3536731
C	2.9166737	1.4414102	0.6126056
C	2.2239019	1.2419008	-1.6948617
C	3.8526189	2.4027221	0.2172582
C	3.1406292	2.2187334	-2.0770268
H	4.4860781	2.8634579	0.9728628
C	3.9672916	2.7998544	-1.1143987
H	3.2116152	2.5233475	-3.1184675
H	4.6879348	3.5635604	-1.3957851
C	-2.2024182	-0.8003002	-0.3567533
C	-2.5666694	-1.3857652	-1.5956655
C	-2.7377989	-1.3551605	0.8215048
C	-3.4596709	-2.4615955	-1.6149641
C	-3.5891712	-2.4556703	0.7954812
H	-3.7443298	-2.8988475	-2.5705733
C	-3.9651884	-3.0019840	-0.4321563
H	-3.9578181	-2.8868234	1.7224205
H	-4.6365869	-3.8562040	-0.4697722
H	-2.4587308	-0.9131615	1.7749697
H	1.5876899	0.7843799	-2.4498261
C	2.8161209	1.0379442	2.0623467

H	3.1395964	0.0001974	2.2104871
H	1.7778147	1.1083104	2.4056848
H	3.4344612	1.6809291	2.6956363
C	-1.9940732	-0.8640473	-2.8924831
H	-0.9009945	-0.9600721	-2.8854790
H	-2.3888672	-1.4223791	-3.7468119
H	-2.2216835	0.1973030	-3.0470707
S	-3.2796222	2.6525460	-0.2092797
S	3.1304289	-2.3642731	-0.3730732
C	-4.6207518	1.5535680	0.3098618
H	-5.5731692	2.0198654	0.0520329
H	-4.4918530	0.6174169	-0.2337645
H	-4.5377697	1.3746796	1.3832517
C	-3.6013608	4.0798527	0.8596872
H	-3.6220619	3.7453867	1.8988874
H	-2.7726381	4.7726455	0.7076904
H	-4.5462274	4.5414102	0.5676183
C	3.1681001	-4.1414253	-0.7274535
H	2.6923007	-4.6421343	0.1161794
H	2.6048896	-4.3383125	-1.6416508
H	4.2084889	-4.4571157	-0.8229309
C	3.8934126	-1.7355414	-1.8891766
H	3.3002123	-2.0600164	-2.7459502
H	3.8876382	-0.6478556	-1.8178843
H	4.9163403	-2.1117474	-1.9455380
C	-0.5508757	3.0149118	0.7539626
C	-0.0624026	3.3064487	2.0409539
C	0.0260057	3.7229484	-0.3300701
C	0.9583662	4.2302164	2.2686674
H	-0.4938618	2.7744859	2.8872124
C	1.0440266	4.6541797	-0.0975025
C	1.5174137	4.9113201	1.1888385
H	1.3194979	4.4095296	3.2790825
H	1.4927894	5.1668183	-0.9462880
H	2.3247598	5.6235175	1.3412793
C	0.3825879	-3.0886259	0.1961647
C	-0.4077764	-3.9536632	-0.5804821
C	0.4142443	-3.3269779	1.5935168
C	-1.1470393	-5.0001035	-0.0291570
H	-0.4539216	-3.7820848	-1.6534927
C	-0.3200464	-4.3843954	2.1433433
C	-1.1004069	-5.2223303	1.3452730
H	-1.7626515	-5.6299487	-0.6669787
H	-0.2828928	-4.5496432	3.2193657
H	-1.6713573	-6.0307265	1.7959455
C	-0.4424024	3.5007512	-1.7483778
H	-1.3833428	4.0301112	-1.9500039
H	0.3023560	3.8604651	-2.4647403
H	-0.6218003	2.4382020	-1.9396780
C	1.2304138	-2.4537526	2.5174513
H	1.1426432	-2.7904722	3.5553892
H	2.2946357	-2.4604388	2.2521663
H	0.8988863	-1.4110666	2.4636739

TSPds : TS, dimerization of **Q** via SMe_2 release
91
Energy = -2620.769857520

B	0.6898916	0.3424582	-0.1003756
C	0.4624099	1.7294299	-0.8196207
C	1.3888993	2.7433989	-0.4844064
C	1.3263097	4.0404629	-0.9844745
C	0.2999979	4.3749032	-1.8670784
C	-0.6091456	3.3921110	-2.2494063
C	-0.5420040	2.0787055	-1.7642554
C	-1.5350761	1.0972088	-2.3220981
H	-2.4855425	1.5954076	-2.5363017
H	-1.1534297	0.6763432	-3.2608427
H	-1.7332243	0.2733748	-1.6427987
H	-1.4055135	3.6447622	-2.9466380
H	0.2125218	5.3847084	-2.2598883
H	2.0657951	4.7786754	-0.6854047
H	2.2089191	2.5000129	0.1840282
H	0.0328800	-0.6418031	-0.5702495
B	1.8308886	-0.1456420	1.0962763
C	3.1942684	-0.8444191	0.5752014
C	4.2455201	-0.0749886	0.0414559
C	5.4561073	-0.6305751	-0.3758114
C	5.6483730	-2.0082381	-0.2718520
C	4.6187050	-2.7988718	0.2404939
C	3.4023529	-2.2438180	0.6633380
C	2.3306361	-3.1618671	1.1980208
H	2.0764371	-2.9084890	2.2342445
H	1.4026199	-3.0642036	0.6250649
H	2.6604243	-4.2052748	1.1637366
H	4.7577242	-3.8762928	0.3141909
H	6.5822669	-2.4636537	-0.5915257
H	6.2388541	0.0063825	-0.7812632
H	4.1126642	1.0017477	-0.0512934
H	1.2913503	-0.7841806	1.9655751
S	2.5796856	1.3492248	2.2181421
C	1.1723505	2.1593627	3.0085406
H	0.6189605	2.6695462	2.2195780
H	0.5377810	1.4072573	3.4770410
H	1.5421949	2.8750715	3.7446187
C	3.2470961	0.4114478	3.6147473
H	4.0668610	-0.1880964	3.2158846

H	2.4661795	-0.2399522	4.0125802
H	3.6155067	1.1050130	4.3723746
S	1.7809949	-0.3497323	-2.6670757
C	2.7622754	-1.8690227	-2.8024693
H	2.1002834	-2.6896288	-2.5171462
H	3.1041013	-2.0143811	-3.8308697
H	3.6079487	-1.8283212	-2.1135555
C	3.0340886	0.8922634	-3.0804723
H	3.9094879	0.7651344	-2.4403009
H	2.5787702	1.8668499	-2.8917068
H	3.3159724	0.8117818	-4.1334515
B	-0.9923773	-1.2216468	0.2560592
C	-1.6390843	-2.1721675	-0.8631821
C	-1.2788693	-2.1528738	-2.2228639
C	-1.8087688	-3.0359057	-3.1642646
C	-2.7373671	-3.9961648	-2.7665146
C	-3.0904355	-4.0669874	-1.4182237
C	-2.5522395	-3.1885977	-0.4705385
C	-2.9416973	-3.3599252	0.9760438
H	-3.5202641	-4.2773228	1.1238596
H	-2.0502086	-3.4058186	1.6143873
H	-3.5449566	-2.5189291	1.3369604
H	-3.7925894	-4.8302226	-1.0849664
H	-3.1668443	-4.6897219	-3.4854518
H	-1.4930695	-2.9719731	-4.2035615
H	-0.5510261	-1.4222542	-2.5618968
H	-0.3202337	-1.8905264	1.0025650
B	-1.6815448	0.1689714	1.0902526
C	-2.9661584	0.9528670	0.5130312
C	-4.0067218	0.2101140	-0.0776548
C	-5.1370084	0.8034056	-0.6372483
C	-5.2545565	2.1938645	-0.6258447
C	-4.2439176	2.9567214	-0.0406707
C	-3.1119887	2.3634528	0.5372937
C	-2.0720760	3.2704520	1.1547783
H	-1.9415546	3.0662804	2.2231339
H	-1.0967252	3.1354016	0.6788176
H	-2.3651447	4.3192069	1.0439528
H	-4.3310629	4.0421899	-0.0315276
H	-6.1200968	2.6810127	-1.0688378
H	-5.9111931	0.1892865	-1.0921827
H	-3.9007312	-0.8704438	-0.1324463
H	-0.7974593	0.9200170	1.4681783
S	-2.3844829	-0.5418621	2.8090046
C	-1.0178728	-1.2999337	3.7230817
H	-0.2113829	-0.5779675	3.8528366
H	-0.6631583	-2.1348198	3.1194019
H	-1.3938821	-1.6479225	4.6866185
C	-2.7050121	0.9018847	3.8552210
H	-3.4579689	1.4959596	3.3346649
H	-1.7889186	1.4801985	3.9848651
H	-3.0928040	0.5631257	4.8176974

TSP : TS, aryl/H shift of adduct **F** forming **P**
41
Energy = -1071.301430323

B	0.1824595	-0.5138037	1.1215179
C	-1.3504940	-0.5058706	0.3020715
C	1.4749873	-0.8298215	0.2392237
C	-1.4021757	-0.2861284	-1.0916203
C	-2.5159687	-1.0392676	0.9274914
C	2.7442364	-0.3882050	0.7034762
C	1.4630181	-1.5699969	-0.9570151
C	-2.5177842	-0.5967214	-1.8614921
H	-0.5223050	0.1148338	-1.5828194
C	-3.6328368	-1.3499523	0.1444632
C	3.9027199	-0.6551628	-0.0370620
C	2.6199801	-1.8358935	-1.6932657
H	0.5149622	-1.9543703	-1.3270723
H	-2.5130967	-0.4222622	-2.9337631
C	-3.6421822	-1.1351657	-1.2344210
H	-4.5147344	-1.7665078	0.6262448
C	3.8518892	-1.3672198	-1.2372446
H	4.8612053	-0.2956673	0.3346653
H	2.5582365	-2.4085109	-2.6161545
H	-4.5269299	-1.3839320	-1.8145006
H	4.7619779	-1.5591518	-1.8002033
B	-0.5962307	0.9162546	1.2180953
H	-1.3946058	1.2902379	2.0314909
H	0.0607142	-1.1977048	2.1115899
S	-0.1623082	2.4970952	0.2787244
C	1.0706399	2.2459500	-1.0233604
H	1.8869027	1.6748263	-0.5808946
H	0.6433157	1.6987257	-1.8631631
H	1.4113908	3.2325814	-1.3411170
C	-1.5757205	3.1223586	-0.6822269
H	-1.8640526	2.3768539	-1.4251222
H	-2.3849338	3.2863464	0.0306012
H	-1.2861700	4.0647353	-1.1505665
C	-2.5696381	-1.3066902	2.4061028
H	-1.9015810	-2.1351464	2.6691330
H	-2.2290659	-0.4380608	2.9783083
H	-3.5865299	-1.5647156	2.7151501
C	2.8560223	0.3800911	1.9978105
H	2.2624842	1.3027844	1.9628440
H	2.4543735	-0.2018793	2.8358254

H 3.8973679 0.6373280 2.2160856

TSR : TS, BH/BB addition of **1R** and **P**
54
Energy = -1576.107018672

B	0.4938845	-0.8301883	-0.2298392
H	1.5539376	-1.2703041	-0.6182848
B	-1.0691619	-1.7975528	-0.9599275
B	-0.1434389	0.4292453	-1.3402458
H	-0.9140918	-2.1387643	-2.0890254
H	-1.0047669	0.1919203	-2.1541440
S	1.4203861	0.7066384	-2.5293523
C	1.4102425	-0.7794805	-3.5605769
C	0.8291292	1.9303579	-3.7290552
H	1.6386574	-1.6141372	-2.8966930
H	0.4175454	-0.9045354	-3.9969430
H	2.1756441	-0.6844692	-4.3324547
H	-0.1193881	1.5883560	-4.1487128
H	0.6810987	2.8571323	-3.1719928
H	1.5800046	2.0721275	-4.5084490
C	0.4620360	-0.4384857	1.3263695
C	1.6292580	-0.3798004	2.1233861
C	-0.7518111	-0.0764106	1.9375056
C	1.5426080	0.0203642	3.4651290
C	-0.8377152	0.3126446	3.2715766
H	-1.6580741	-0.0897236	1.3360021
C	0.3222126	0.3626599	4.0465706
H	2.4525507	0.0651329	4.0624623
H	-1.7982356	0.5883765	3.7012249
H	0.2799028	0.6709444	5.0887661
C	2.9898860	-0.7197595	1.5571028
H	3.2476430	-0.0652526	0.7160085
H	3.7645885	-0.6139906	2.3237814
H	3.0204539	-1.7456749	1.1725368
H	-0.0776114	-2.0166934	-0.1871838
H	-2.0742730	-1.3078358	-0.5551085
S	-1.5312439	-3.8992600	-0.1658215
C	0.0509933	-4.7232612	-0.4868258
H	0.1007383	-4.9072956	-1.5614699
H	0.8741985	-4.0706838	-0.1820998
H	0.0887788	-5.6700867	0.0545236
C	-1.4027082	-3.6505027	1.6270607
H	-0.5176150	-3.0513889	1.8537149
H	-2.2983131	-3.1086759	1.9354175
H	-1.3616864	-4.6202326	2.1263717
C	-0.3462438	1.8397089	-0.5963624
C	0.7335618	2.4588133	0.0606058
C	-1.6018620	2.4884428	-0.5282092
C	0.6074998	3.6634925	0.7493181
H	1.7038597	1.9649237	0.0492823
C	-1.7287930	3.6953221	0.1739561
C	-0.6378009	4.2895644	0.8089554
H	1.4685688	4.1017201	1.2488268
H	-2.7051341	4.1757317	0.2247488
H	-0.7622635	5.2251466	1.3492402
C	-2.8291218	1.8956453	-1.1824778
H	-3.7165177	2.4961393	-0.9567719
H	-2.7149521	1.8447461	-2.2719727
H	-3.0063813	0.8704546	-0.8398412

References

[1] A. Hübner, T. Bernert, I. Sänger, E. Alig, M. Bolte, L. Fink, M. Wagner, H. W. Lerner, *Dalton Trans.* **2010**, *39*, 7528–7533.

[2] D. J. Parks, W. E. Piers, G. P. A. Yap, *Organometallics* **1998**, *17*, 5492–5503.

[3] N. Tsukahara, H. Asakawa, K. H. Lee, Z. Lin, M. Yamashita, *J. Am. Chem. Soc.* **2017**, *139*, 2593–2596.

[4] R. Köster, P. Binger, W. Fenzl, E. R. Wonchoba, G. W. Parshall, in *Inorganic Syntheses* (Ed.: G. W. Parshall), McGraw-Hill, New York, NY, **1974**, pp. 134–136.

[5] R. F. Culmo, Shelton, CT **2013**.

[6] A. Del Grosso, R. G. Pritchard, C. A. Muryn, M. J. Ingleson, *Organometallics* **2010**, *29*, 241–249.

[7] in *Software for the CCD Detector System*, Bruker Analytical X-ray Systems, Madison, WI, **2016**.

[8] in *Software for the CCD Detector System*, Bruker Analytical X-ray Systems, Madison, WI, **1998**.

[9] R. H. Blessing, in *Program for absorption corrections using Siemens CCD based on the method of Robert Blessing, Vol. A51*, Acta Cryst., **1995**, pp. 33–38.

[10] aG. M. Sheldrick, in *Program for Crystal Structure Refinement*, University of Göttingen, Göttingen, Germany, **1997**; bG. M. Sheldrick, in *Program for Crystal Structure Refinement*, University of Göttingen, Göttingen, Germany, **1997**.

[11] V7.3 ed., a development of University of Karlsruhe and Forschungszentrum Karlsruhe GmbH,, 1989-2007, TURBOMOLE GmbH, since 2007, available from http://www.turbomole.com, **2018**.

[12] J. Tao, J. P. Perdew, V. N. Staroverov, G. E. Scuseria, *Phys. Rev. Lett.* **2003**, *91*, 146401.

[13] aS. Grimme, J. Antony, S. Ehrlich, H. Krieg, *J. Chem. Phys.* **2010**, *132*, 154104–154118; bS. Grimme, S. Ehrlich, L. Goerigk, *J. Comput. Chem.* **2011**, *32*, 1456–1465.

[14] aF. Weigend, M. Häser, H. Patzelt, R. Ahlrichs, *Chem. Phys. Lett.* **1998**, *294*, 143–152; bF. Weigend, R. Ahlrichs, *Phys. Chem. Chem. Phys.* **2005**, *7*, 3297–3305.

[15] A. Klamt, G. Schüürmann, *J. Chem. Soc., Perkin Trans. 2* **1993**, 799–805.

[16] aK. Eichkorn, F. Weigend, O. Treutler, R. Ahlrichs, *Theor. Chem. Acc.* **1997**, *97*, 119–124; bF. Weigend, *Phys. Chem. Chem. Phys.* **2006**, *8*, 1057–1065.

[17] P. Deglmann, K. May, F. Furche, R. Ahlrichs, *Chem. Phys. Lett.* **2004**, *384*, 103–107.

[18] S. Grimme, *Chem. Eur. J.* **2012**, *18*, 9955–9964.

[19] F. Eckert, A. Klamt, *AIChE Journal* **2002**, *48*, 369–385.

[20] F. Eckert, A. Klamt, in *Release 16.01*, Version C3.0 ed., COSMOlogic GmbH & Co. KG, Leverkusen, Germany, **2015**.

[21] Y. Zhao, D. G. Truhlar, *J. Phys. Chem. A* **2005**, *109*, 5656–5667.

[22] F. Weigend, F. Furche, R. Ahlrichs, *J. Chem. Phys.* **2003**, *119*, 12753–12762.

[23] L. Goerigk, A. Hansen, C. Bauer, S. Ehrlich, A. Najibi, S. Grimme, *Phys. Chem. Chem. Phys.* **2017**, *19*, 32184–32215.

[24] G. Schreckenbach, T. Ziegler, *J. Phys. Chem.* **1995**, *99*, 606–611.

[25] D. J. Parks, R. E. von H. Spence, W. E. Piers, *Angew. Chem. Int. Ed.* **1995**, *34*, 810–811.
