# Supplementary material for: Reactions of B2(o‐tolyl)4 with Boranes: Assembly of the Pentaborane(9), HB[B(o‐tolyl)(μ‐H)]4
Source: Angew Chem Int Ed Engl. 2021 Mar 4;60(15):8532–6. doi: 10.1002/anie.202101054 (PMC8048642; doi:10.1002/anie.202101054)

checkCIF/PLATON report

Structure factors have been supplied for datablock(s) d20131_a

THIS REPORT IS FOR GUIDANCE ONLY. IF USED AS PART OF A REVIEW PROCEDURE FOR PUBLICATION, IT SHOULD NOT REPLACE THE EXPERTISE OF AN EXPERIENCED CRYSTALLOGRAPHIC REFEREE.

No syntax errors found. CIF dictionary Interpreting this report

Datablock: d20131_a

```
Bond precision:  C-C = 0.0020 A          Wavelength=1.54178

Cell:             a=15.5007(6)      b=15.5007(6)      c=5.1190(3)
                  alpha=90          beta=90           gamma=90
Temperature:      150 K

                Calculated                Reported
Volume          1229.95(12)               1229.95(12)
Space group     P 4/n                     P 4/n
Hall group      -P 4a                     -P 4a
Moiety formula  C28 H33 B5                C28 H33 B5
Sum formula     C28 H33 B5                C28 H33 B5
Mr              423.59                    423.59
Dx,g cm-3       1.144                     1.144
Z               2                         2
Mu (mm-1)       0.447                     0.447
F000            452.0                     452.0
F000'           453.04
h,k,lmax        18,18,6                   18,18,6
Nref            1137                      1129
Tmin,Tmax       0.935,0.982               0.633,0.753
Tmin'           0.935

Correction method= # Reported T Limits: Tmin=0.633 Tmax=0.753
AbsCorr = MULTI-SCAN

Data completeness= 0.993          Theta(max)= 68.327

R(reflections)= 0.0467( 864)      wR2(reflections)= 0.1338( 1129)

S = 1.100                  Npar= 84
```

The following ALERTS were generated. Each ALERT has the format
test-name_ALERT_alert-type_alert-level.
Click on the hyperlinks for more details of the test.

Alert level B

```
PLAT410_ALERT_2_B Short Intra H...H Contact  H2B      ..H2B      .       1.87 Ang.
                                               3/2-y,x,z  =      3_655 Check
```

Author Response: This is the actual distance between the bridging H atoms in the molecule.

Alert level C

```
PLAT303_ALERT_2_C Full Occupancy Atom H2B       with # Connections       2.00 Check
PLAT906_ALERT_3_C Large K Value in the Analysis of Variance ......      2.049 Check
PLAT911_ALERT_3_C Missing FCF Refl Between Thmin & STh/L=    0.600          5 Report
```

Alert level G

```
PLAT912_ALERT_4_G Missing # of FCF Reflections Above STh/L=  0.600          2 Note
PLAT913_ALERT_3_G Missing # of Very Strong Reflections in FCF ....          3 Note
PLAT978_ALERT_2_G Number C-C Bonds with Positive Residual Density.          2 Info
```

0 **ALERT level A** = Most likely a serious problem - resolve or explain
1 **ALERT level B** = A potentially serious problem, consider carefully
3 **ALERT level C** = Check. Ensure it is not caused by an omission or oversight
3 **ALERT level G** = General information/check it is not something unexpected

0 ALERT type 1 CIF construction/syntax error, inconsistent or missing data
3 ALERT type 2 Indicator that the structure model may be wrong or deficient
3 ALERT type 3 Indicator that the structure quality may be low
1 ALERT type 4 Improvement, methodology, query or suggestion
0 ALERT type 5 Informative message, check

It is advisable to attempt to resolve as many as possible of the alerts in all categories. Often the minor alerts point to easily fixed oversights, errors and omissions in your CIF or refinement strategy, so attention to these fine details can be worthwhile. In order to resolve some of the more serious problems it may be necessary to carry out additional measurements or structure refinements. However, the purpose of your study may justify the reported deviations and the more serious of these should normally be commented upon in the discussion or experimental section of a paper or in the "special_details" fields of the CIF. checkCIF was carefully designed to identify outliers and unusual parameters, but every test has its limitations and alerts that are not important in a particular case may appear. Conversely, the absence of alerts does not guarantee there are no aspects of the results needing attention. It is up to the individual to critically assess their own results and, if necessary, seek expert advice.

Publication of your CIF in IUCr journals

A basic structural check has been run on your CIF. These basic checks will be run on all CIFs submitted for publication in IUCr journals (*Acta Crystallographica*, *Journal of Applied Crystallography*, *Journal of Synchrotron Radiation*); however, if you intend to submit to *Acta Crystallographica Section C* or *E* or *IUCrData*, you should make sure that full publication checks are run on the final version of your CIF prior to submission.

Publication of your CIF in other journals

Please refer to the *Notes for Authors* of the relevant journal for any special instructions relating to CIF submission.

PLATON version of 05/12/2020; check.def file version of 05/12/2020

Datablock d20131_a - ellipsoid plot

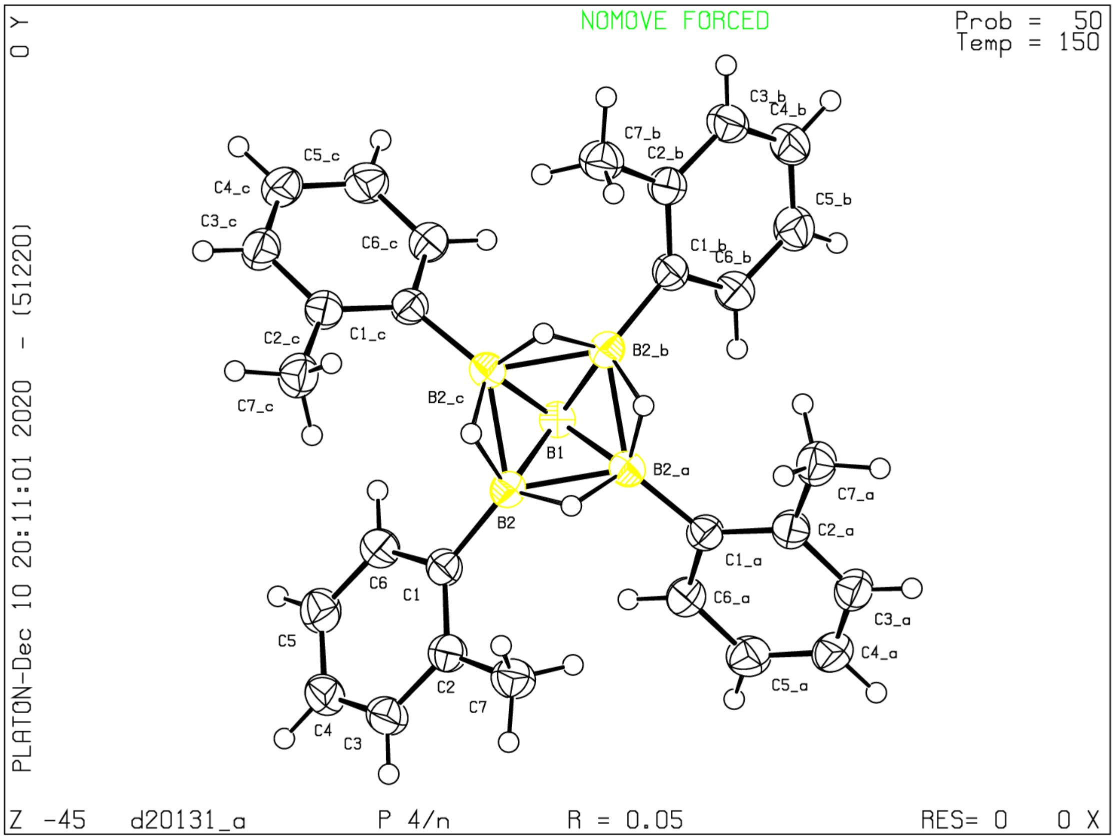

Supplement: Supplementary file 1 — Supplementary [file B5H5(o-tolyl)4-checkcif.pdf › B5H5(o-tolyl)4-checkcif.pdf]
